# Experiences of parents and stakeholders in caring for, and supporting children with special needs in Ghana

**DOI:** 10.1371/journal.pone.0281502

**Published:** 2023-03-03

**Authors:** Joshua Amo-Adjei, Ruth Essuman, Anastasiia Nurzhynska, Antoine Deliege, Geeta Sharma, Iddi Iddrisu, Charity Nikoi

**Affiliations:** 1 Faculty of Social Sciences, Department of Population and Health, University of Cape Coast, Cape Coast, Ghana; 2 Kantar Public, Accra, Ghana; 3 UNICEF Country Office, Accra, Ghana; 4 UNICEF Country Office, Juba, South Sudan; Kasturba Medical College, Manipal Academy of Higher Education, Manipal, INDIA

## Abstract

We studied the caring, parenting, and support services for children with special needs in Ghana. Many of the study participants reported re-adjusting their lives in virtually every domain–social, economic, and emotional to deal with and manage the new realities. How parents navigate this space varied considerably from setting to setting. Regardless of individual and interpersonal resources, community, institutional, and policy circumstances seemed to exacerbate notions of disability. In many instances, parents had a low depth of suspicion about the precursors to disabling events in their children. Parents are constantly pursuing health care, including a cure for their children with disabilities. Views about “otherness” were noted, and these tended to undermine medical interpretations/explanations of disability generally, which in turn affected formal education and health-seeking for children. Institutional arrangements exist to encourage parents to invest in their children regardless of their perceived abilities. However, these do not seem to be sufficient, particularly for health and formal education. Programming and policy implications are highlighted.

## 1 Introduction

Through the interaction of environmental (physical and social) and genetic triggers, many children with different forms of disabilities are born daily [1–4]. Several others, by the same environmental and genetic factors, develop disabilities of diverse kinds every day after birth [5,6]. While many parents accept carrying foetuses with the potential for developmental challenges to term [7,8], many others, for personal and other reasons, terminate pregnancies with risks of development deficiencies [9,10].

Consequently, the conceptual boundaries of disability become puzzling [11]. Nonetheless, there are two broad conceptualisations of disability: incrementalism and reconceptualism. The incrementalists view operates along the medical model, which symbolizes negativity, assuming that there is a deficiency within an individual that must be “fixed”, “cured”, “accommodated” or “endured”. For instance, Mitra [11], aligning with the incrementalists, describes disability as “deprivation in terms of functioning(s) among persons with health deprivations” (p14). The other perspective–advanced by *reconceptualists*, asserts that disability is a social construct, which “resides more in the minds of the beholders than in the bodies of the beheld” [12]. The reconceptualist view faults incrementalists for ignoring the wider social and environmental influences in producing disability perceptions on children with special needs (CwSN) [13]. Put together, disability arises from the extent of interaction among resources, personal and structural environments, and health deprivations.

Since the onset of many disabling conditions among children can be sudden, parents of children with a disability may experience exceptional life circumstances. Parents and caregivers may have to begin adjusting and re-adjusting their priorities, including financial, material, and emotional resources, to navigate the new challenges. At the emotional level, ample evidence illustrates pessimism, hostility, shame, denial, blame, guilt, grief, withdrawal, rejection, helplessness, anger, anxiety, shock, disbelief and self-blame [14,15]. Simply, a section of parents who have CwSNs may have strong negative notions and feelings about themselves (e.g., self-blaming) as well as their children [14,16–18]. In Ghana, there are strong beliefs in witchcraft and magic [19] and perceived to be a cause or consequence of disability [20].

On the other hand, there is a fledgling body of knowledge that demonstrates positive experiences among parents/caregivers of children with different disabilities and levels of severity [21–27]. For instance, Potter [27] study of fathers of children with autism revealed positive recollections on individual qualities, strong emotional bonds with children and father’s personal development on taken-for-granted events in parenting abled children. In a different study focusing on mothers of children with special needs, Kayfitz, Gragg [26] found that having children with disabilities was viewed as empowering as they became more sensitive and conscious of people with disabilities, enriched their tolerance of life events, and deepened beliefs in life’s purpose for all people.

The literature on parenting and social support/services for CwSN is growing, but they are limited in two main respects. First, much of the existing evidence focuses on one or another form of disability, with considerable focus on autism [28–30], cerebral palsy [31], intellectual disabilities [32–35], and hearing impairment [36]. Second, limited attention has been paid to community and institutional support systems (health and education particularly) for parents/caregivers of children with special needs/disabilities. Therefore, this paper seeks to contribute to understanding of experiences of nurturing/parenting children with special needs (CwSNs) in Ghana. To develop deeper insights, we draw qualitative data from parents, community leaders, and social services providers (education, health, nutrition, social welfare, and child protection agencies) of diverse sociocultural and economic backgrounds from the southern, central, and northern parts of Ghana. The rest of the paper is organised as follows: conceptual framework, methods, findings, discussions, and conclusions.

### 1.1 Conceptual framework

**Fig 1:**
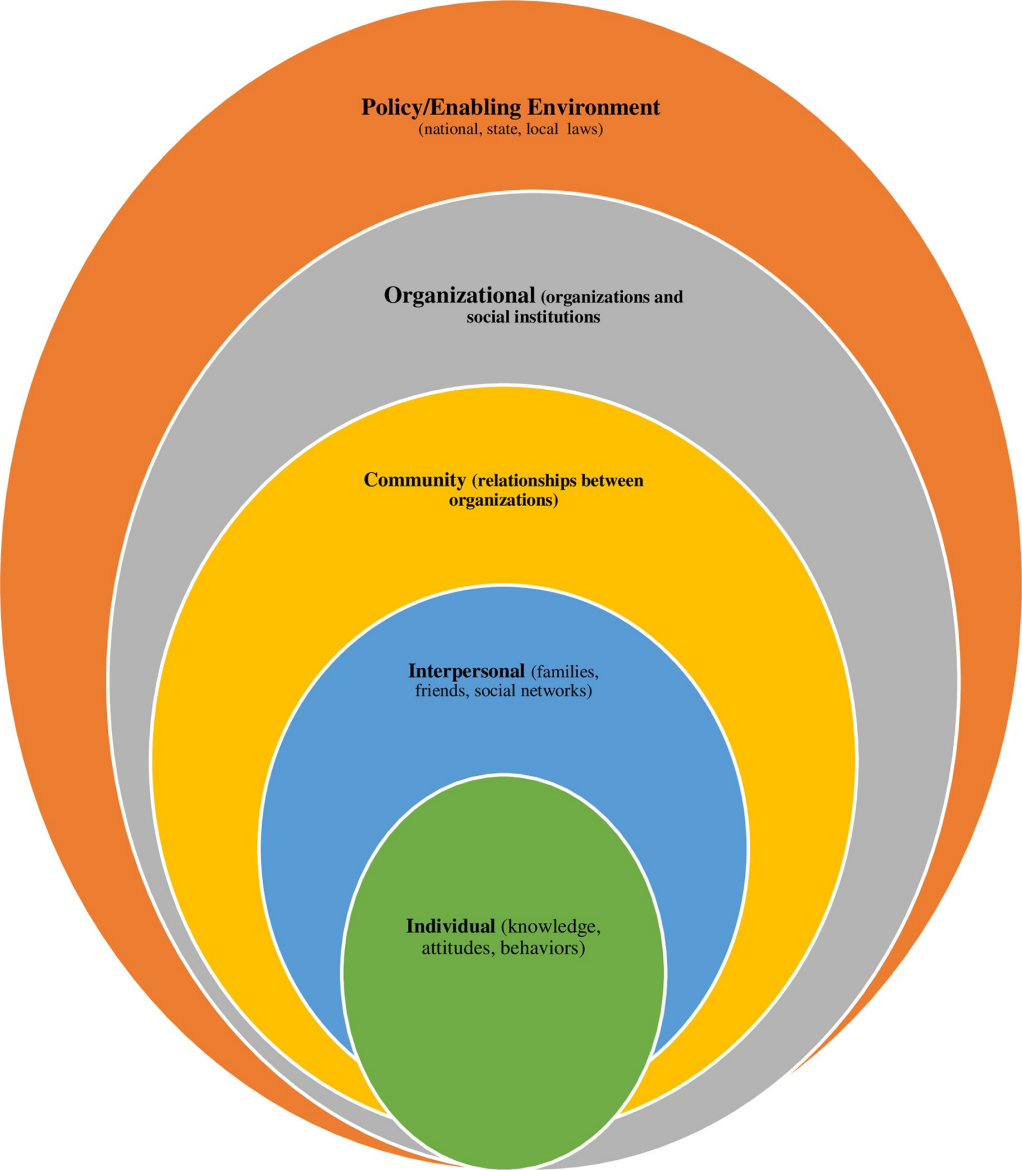
Socio-ecological model.

This work is theorized around the socio-ecological model (SEM). Originally propounded by Bronfenbrenner in 1992 and named ecological systems theory, the framework locates individual behaviour (parenting in this context) in a web of systems that are shaped and influenced by each other. It is anchored on the assumption that individual behaviours are connected to and formed within interactive social and physical environments. The SEM framework is organized into five hierarchical levels–individual, interpersonal, community, organization, and policy/enabling environment (Fig 1).

At the individual level, the drivers of behaviour or actions are primarily on the account of characteristics such as age, gender, educational attainment, economic resources, expectations, and values. These in turn shape knowledge, attitudes, and competencies to handle or manoeuvre within the complexities of life events around health, nutrition, education and health and parenting [37–39]. For instance, child survival rates are higher among children of educated parents, particularly women, than those with no or little education [40] and birth defects are less common in fathers with better education [41] partly because educated fathers are better positioned with resources (knowledge, wider social networks and economic) that advance quality early childhood screening and care services.

The second level of the framework asserts the importance of interpersonal relationships, which comprises formal and informal social networks and support systems that may shape practices and attitudes such as parenting. Studies signal that social network shape childcare in both positive and negative ways. For instance, research in a slum area in Nairobi showed that single mothers extensively relied on kin support for child nurturing [42]. Other studies have also reported mixed findings on the benefits of grandmothers to childcare. Pandey and Littlewood [43] affirmed that combining community resources with grandmothers raising grandchildren enhanced family resiliency, social support and self-efficacy. Some evidence is contrary, though, showing that grandmothers could obstruct parenting practices [44].

The community follows the interpersonal domain. The locus here is concerned with dealings or affairs among and between informal networks that subsist in communities such as religious groupings/associations, hometown unions/associations, community and religious leaders etc. [45–47]. However, at the community level, including school environments and care homes, parenting practices could be constrained, particularly for children with special needs [48].

At the institutional level, parenting practices may be influenced or affected by organizational systems (e.g., in health, education, and social protection/safety). These institutional arrangements may promote or discourage the utilization of very critical resources for childcare and nurturing of CwSNs. Policy and programmatic considerations are at the final stage of the SEM model, which operates at the national and sub-national levels. Enabling a policy environment for parenting is critical to the successes or failures of parenting at the individual level [49]. In more developed welfare states, for instance, parents who consistently show poor parenting qualities (e.g., physical, or sexual violence) may have their children taken to institutional care homes. Anecdotal evidence indicates that parents’ concerns about their children being taken into care homes adapt to proper standards of parenting behaviours [50]. Also, by virtue of a supportive policy environment for parenting, parents with children with special needs may receive helpful services to address limitations in their ability to provide care and support (e.g., financial, material, and emotional resources) [49].

## 2 Methods

### 2.1 Data

Data for this paper was extracted from a larger study (Essuman & Amo-Adjei, 2019) to understand the perspectives of primary (children, parents, and grandparents) and secondary stakeholders on parenting in Ghana. Specifically, it explored the social, economic, cultural, and political contexts of parenting for CwSNs. We utilized multiple qualitative techniques–in-depth interviews, key informant interviews (KII) and observations in the larger study.

### 2.2 Research sites

We purposely sampled eight districts from the Northern (Tolon, Kpandai, Wa West and Lambushhie), Middle (Kwabre East) and Southern (North Tongu, Komenda-Edna-Eguafo-Abirem and Accra Metropolitan) regions of Ghana. In each district, we purposely selected one urban and one rural community, given a total of 16 communities from the eight (8) districts. District capitals were automatically (in many districts, the capital was the only urbanised settlement) selected for the urban sites in each district and one rural town was conveniently selected.

### 2.3 Study population and sampling

A sub-sample of 62 out of 440 is used for this paper. The breakdown of this sub-sample is provided in Table 1.

**Table 1:** Number of participants.

Target group	Number of participants
National Policymakers	4
Domestic Violence Support Unit of Ghana Police Service	4
District Health Directorate (Nutrition and dietetics)	8
Community leaders (e.g., local political leaders, traditional leaders, religious leaders [Pastors and Imams]	20
school teachers (crèche, kindergarten, primary, junior high and secondary schools)	10
Parents/Primary caregivers of children with special needs	16
Total	62

The index child also had to be co-resident with the primary caregiver/parent. The overall criteria for selecting participants were underpinned by the Socio-Ecological Framework, which theorizes/conceptualises norms, behaviours, and practices based on individual, interpersonal, community, institutional and policy (macro) environments within which events occur (caring for and supporting CwSNs in this case).

As a key guiding principle in determining the sample size, we were minded not to generate a too large sample to compromise on depth but at the same time, not too small to affect the information redundancy required for exploratory qualitative studies. The use of sample size over information redundancy is due to the concurrent deployment of moderators across the study sites and the use of multiple interviewers could not have allowed for data saturation. To capture parents with diverse backgrounds (e.g., neighbourhood, child’s type of disability, maternal age, education), field moderators used a structured screening guide to select parents who fit into predetermined criteria.

Twenty fieldworkers, with cultural and linguistic knowledge of the study communities were recruited and trained for five days, inclusive of pre-testing. We pretested the instruments in Madina in Accra, a multi-ethnic settlement that reflects the population and ethnicities of Ghana. It helped us to simulate and adjust the tools for the final study. The fieldwork was conducted in April and May 2019. The Ghana Health Service Ethics Review Committee reviewed and certified the protocol for scientific and ethical clearance (GHS-ERC 011/02/2019). Fieldworkers were also trained adequately in field ethical compliance. Cognizant of interviewing parents who have children with disabilities, we arranged for an on-call counsellor to provide any emotional support to parents /caregivers during the process of the interviews. All research participants gave verbal and written consent after explaining the purpose, selection criteria, and the nature of the questions as well as the duration of the interview to each participant. The research information was explained to participants in a local language they understood. A copy of the information sheet and signed consent form were retained by the participants.

### 2.4 Data analysis

We followed the Consolidated Criteria for Reporting Qualitative Research (COREQ) [51] in analysing the text data. Both deductive and inductive techniques were applied to analyse the data. The deductive approach was framed around the major themes or research objectives and questions (parent nodes). For the next stage, we used inductive techniques to identify child and grandchild nodes through immersive, detailed, and repeated reading of the text. Based on these, we developed a coding framework, which guided the coding of text using the NVivo software. The codes were then reviewed by JAA and RE to check for inconsistencies and to re-align codes where necessary. Thematized accounts of participants are presented under sections and supported by relevant extracts for the interviews.

## 3 Results/Findings

The key themes explored/extracted from the narratives are nature/kinds and timing of disability, community, and traditional notions about children with special needs (CwSN), perceptions and stigmatization of CwSN, social services for CwSN, positive experiences parenting children with disabilities, challenges of parenting CwSN and disciplining of CwSN.

### 3.1 Timing of onset/detection of disability

The types of disabilities parents reported were visual impairment, hearing loss, spinal problems, autism, and walking impairment. Parents gave different accounts of the timing of the onset of disability. In the opinion of some parents/caregivers, the disability condition(s) developed after birth and among this group of parents, the diagnosis was made during routine postnatal clinic visits. A mother with an autistic child recalled:

We went for a check-up when she was 3 years old, and the doctor confirmed she has autism. I was not informed, it was my husband they told, I got to know about it later. (Mother, Osu, Greater Accra).

Some other participants also shared that they observed the disabilities in children immediately after birth. Some mothers recalled that at birth, they observed certain unusual physical signs that were concerning. However, the depth of suspicion was generally low. Consequently, the diagnosis was confirmed after the conditions had deteriorated and became conspicuous that medical treatment was sought. One participant narrated:

As I said, it started from birth. His eyes were red as though there was blood on his eyes from birth. So, we used to drop breast milk on his eyes and as we do that, the blood disappears. It is up to this age that we are realizing that he may probably be suffering from an eye problem. (Mother, Mape, North Tongu, Volta Region)

Our data also shows stories about the sudden onset of disabilities among some children. This group of parents reported that the condition of their children resulted from sudden illness episodes. For example, the mother of a child with cerebral palsy indicated that the condition of her child was detected after a severe malaria illness. She asserted:

It was after birth, she was 10 months when she had severe malaria, and that led her to be deformed because now she can sit unless you hold her, when you hold her sometimes, she sits a little, then she becomes tired if you are not there to hold her, she falls off, she can’t walk she can’t stand, she can’t talk but she hears when you call her, she turns and looks at you. (Mother, Lambushie, Upper West)

### 3.2 Perceived notions about CwSN

Parents with CwSN explained community notions about such children with two descriptors areas: *sympathy* and *abnormality*. Of sympathy, some parents narrated that people in their communities appeared to care and sympathize with their “predicament”. According to them, sympathy manifested in supportive encouragement on what they could do to improve their parenting skills. An important support they received from relatives and friends was encouraging them (parents/caregivers) to provide equal educational opportunities for CwSN comparable to opportunities for children without special needs. A participant remarked:

People always tell me to send him to school for proper education. I think they like him even when they see other children trying to bully and disturb him, they try to intervene by chasing away such people. (Mother, Lambushie, Upper West)

Narratives about abnormality were in the context of children’s physical appearances and the cause of the disability. In terms of appearance, it was primarily about people being scared/afraid or simply unwilling to get close to CwSN for being physically different from others. This view was connected to people’s perceptions about the cause of disability. In communities where children with special needs were viewed as originating from the gods or because of a curse invoked on one of the parents or both, people (usually non-household/family members) avoided having any physical contact with such children. Views of abnormality were also linked to sexual infidelity on the part of one of the parents, especially a mother. One mother narrated her experience in the following:

I’ve heard people say that when I was pregnant, I had sex with another man who isn’t my husband, and the result is what has happened to my child. (Mother, Battor, North Tongu, Volta Region)

Among parents/caregivers in communities where the cause of disability was regarded as a curse, out of frustration, some had resorted to consulting spiritual and traditional healers for a cure. For other parents, the reason for consulting traditional healers was aimed at exonerating themselves from accusations of witchcraft (as the cause of disability) and curses (consequences for offending others). However, participants who pursued this course admitted that they had not succeeded in getting a “cure” for their children. One participant recalled:

Some people have attributed the disability of my child to witches, who have changed the destiny of the child, so they could hurt me. I made attempts by visiting them with the child, but it yielded nothing; I didn’t see the result I wanted to see. (Mother, Battor, North Tongu, Volta Region)

### 3.3 Positive parenting stories

This section explores what parents considered they are doing well in their parenting of children with disabilities. Two practices emerged repeatedly: early stimulation and education and regular health assessment. On early stimulation, participants discussed deliberate efforts to enrol their CwSN in the formal school system. Parents expressed the hope that investing in the human capital development of their CwSN as early as possible, would certainly add value to their future life opportunities. To the best that they could, these parents indicated that they did not want their CwSN to be a burden on others or the state. Accordingly, they indicated formal education offered the best possible route to this vision. One parent narrated:

I’m very focused on my child’s schooling and I want to make sure that my child goes to school so she makes use of her knowledge so she can become someone great in the future. When we are in the house there is a slate [learning board] that I help her to write on. I also sing for the child and sometimes play different games to stimulate her mind at home. (Mother, Mamponteng, Kwabre East, Ashanti Region)

Another practice commonly mentioned by all parents was regular health seeking. Parents recounted the sustained efforts they have been making to ensure that the health of their children is maintained or improved. Some parents expressed the view that they were seeking health care regularly, per adventure, the child may fully recover from the disability. Some of the parents reported combining both traditional and orthodox treatment. From the narratives, we note, however, that caregivers who resort to traditional healing did so because they lacked the financial means to rely exclusively on orthodox treatment. One caregiver illustrated:

I am taking him around looking for medicine (traditional). Whenever I get money, I take him to the hospital also, hoping that he could regain full recovery [health]. (Mother, Wa West, Upper West)

### 3.4 Challenges in parenting CwSN

We again elicited from parents some of their difficulties raising CwSNs. Parents/caregivers recounted varied challenges of raising/nurturing CwSNs. The data showed that childcare-occupational conflicts, and social and emotional dissonances were prevalent in the narratives. Noteworthy, however, was a case of a parent who expressed deep regrets about the decision to keep the pregnancy rather than have an abortion.

For some parents, especially mothers, the nature of the disabilities their children suffered required considerable assistance and support in undertaking basic functional roles. Eventually, some parents whose children were highly dependent in terms of functional activities deserted their careers to get more care time for their CwSNs. A mother’s experience is excerpted in the following:

I was a seamstress apprentice, but her condition made me stop. I am currently a petty trader which gives me some small income and gets more time for her. I initially decided that after birth, I would solicit my parents’ support for complementary care and return to pursue higher education. Unfortunately, I am still home because of the child’s condition. (Mother, Battor, North Tongu, Volta Region)

We heard stories from parents suggesting that the disabilities of their children had taken a huge toll on their social relations, including marital life. Some parents with CwSNs had lost friends who did not want to get close to them because of their children’s disability situation. A mother recalls her experience in the textbox below:

Hmm! A normal child should start crawling at least after 7 months, but I did not see any sign of that with my child. I planned to kill her, but her grandmother asked me not to kill her because it was for a reason God gave him to me. His grandmother encouraged me for that thought to fade away.

Yes, my husband nearly ended the marriage because of that. Because he was thinking that there is no such thing in his family. Therefore, his family had to meet with my family, and they advised him that there is no trace of such illness in either family. If he should leave the marriage, who is he expecting to take care of such a child for him? That is why my husband stayed in the marriage. It is difficult because you must feed, bathe and do almost everything for her. She cannot do anything for himself, she is 8 years old now, a normal child her age should be able to at least sweep but she cannot do anything. She still wears diapers at her age.

Most of my friends have left me because of her. In addition, I cannot leave her behind and go out for any social gathering. Those situations have thought me a life lesson and made me trust in God. Because I did not know that my child could walk one day. It is about finance because sometimes what to eat is very difficult; It is only my husband that works, I would not know what would have happened if I were not staying with my mother. I sometimes give my children food and go to bed with a hungry belly sometimes. (Mother, Osu, Accra)

We also heard accounts of parents’ deep frustrations arising from the limited opportunities for adequate health care services for their children. Connected to this is the high cost of seeking health care for CwSNs. According to many of the parents, the cost of health is beyond their financial means, forcing them to rely on over-the-counter medications instead of taking their children to the hospital. A frustrated parent gave the following account:

As I said, it is because of the financial problems that I usually take him to the pharmacy or drug store instead of the hospital when he is not well. I feel like I am not able to do exactly what I must do to him because of financial problems. I go through a lot. (Mother, Mape, North Tongu, Volta Region)

Due to the challenges of raising CwSNs, some parents expressed strong regrets for the decision to give at all. One mother, for instance, indicated that she was advised to abort the pregnancy due to abnormalities that had been detected during antenatal care. According to this participant, she declined the advice. She now regrets not opting for abortion because the child has serious abnormalities. She recounted:

It’s very difficult for me to talk about my child; my eyes are usually filled with tears when I talk about it. She is my first-born and I love her. When I was pregnant, I was asked to abort her, but I insisted on delivering her for her to become whatever she ought to be. I have finally given birth and it’s 1yr 7months yet still my child could not sit nor do anything. I am a young lady of only 28yrs. I must work to cater for her and her education. Who do I have to take her for me while I go to work? It is burdensome for me; anytime I think about it, tears fill my eyes, and I don’t want to even talk about it because it’s giving me headaches. She cannot see and cannot talk. (Mother, Mape, North Tongu, Volta Region)

### 3.5 Social services for children with special needs

In this section, we aimed to understand the social services available to support parents with CwSN. The first item explored was the capacity of community leaders to help parents traverse parenting children with special needs. All but one of the nine community-level participants indicated that they had received some form of training from the various NGOs operating in their communities. The content of the training focused on referral services (e.g., counselling, financial aid, and educational opportunities) for parents. Churches and NGOs were frequently mentioned as the organizers of the training programmes. The narratives below throw more light on these reports:

This year, we are giving special training to our children ministry teachers to know how to treat or teach such children (disabled). (Community Leader, Abirem, KEEA, Central Region)

Yes, but I said PAN-AFRICA has come here some time ago; assembled the community members and educated us on how to manage such children with special needs in our homes. (Mother, Tolon, North Tongu, Volta Region)

Yes, just like I said this child and family life issue, I think either the beginning of this year or the later part of last year the disability group came and told they were, we had one day just interaction and they were trying to let us understand how children with disability can be sent to the formal system and that they are also trying, some teachers will be trained so that they will be in the normal school where these children will go and they will have the skills trying to also impact knowledge onto these children. (Community Leader, Lambushie, Upper West)

However, at the community level, participants point to the lack of any structured or formal support services or programmes that support children with disabilities. Any support that CwSN received from community members was often at the individual/personal level of community members. An assembly in Elmina commented:

There is nothing like that here. There was a guy who was deformed in one leg and couldn’t walk well, with the consent of his parents the church pushed him into employment. Yes, they do especially the children who cannot walk well or have a hearing impairment but with the hearing impairment when it’s noticed early, the child is made to sit in the front seats only so that they can get special attention from their teachers. (Community Leader, Lambushie, Upper West)

At the district level, our data showed two main forms of support services that parents who have CwSN received. The first was community sensitization programmes conducted by the Department of Social Welfare from time to time. These sensitization programmes are often aimed at highlighting the rights of CwSN as any other children foremost. Again, these programmes are also used to sensitize and point people to the potential and capabilities of CwSNs. Another service the Social Welfare staff offered to parents who have CwSNs was counselling support to navigate the emotional stresses occasioned by disability. An officer of the Department of Social Welfare described typical support for parents with a CwSN in the following:

We visited a child with a disability; she didn’t know what to do because of the new experience; she needed to now guide him and a host of other support interventions. We counsel them on the potentials of such children, especially how sharp their other senses are. We let parents know about the many career options open to children with special needs of different kinds. Our approach is to make parents appreciate that the Department shares in their parent pursuits. (Department of Social Welfare, North Tongu)

The Department of Social Welfare also served as the conduit for the transfer of mandatory 10% of the District Assemblies Common Fund earmarked for people with disabilities in each district. Ghana runs a decentralised governance setup broken into two main levels: central and local governments. The central government represents the national while district assemblies are at the local level. The district assemblies receive budgetary support from the national government to fund development projects and social services. The legal framework obliges district assemblies to support persons with disabilities, which is channelled through the Department of Social Welfare. Beneficiaries may elect to use the funds for health, educational and job creation activities.

A participant of the Ghana Education Service (GES) corroborated the accounts from the Social Welfare Department, asserting that their support services focused on letting parents who have children with special needs identify educational/schooling opportunities that CwSNs could exploit with limited to no constraints.

We do talk to them. The special education officer also goes around to talk to parents when there are PTA meetings. That is where the special education officer will also go and have a talk with those people. (Official, Ghana Education Service, Kpandai, North Region)

Further analysis of the data shows that while educational officers conducted outreach activities to encourage parents/caregivers to enrol CwSN in the formal education system, accounts of teachers showed that many schools did not have disability-friendly facilities. Besides, the teacher participants revealed a lack of capacity to screen and identify students with special needs. They relied on information from parents/caregivers, or the nature of disability/special need was conspicuous. Many teachers narrated that both pre-service and in-service capacity-building opportunities for in-school level screening of special needs were not available in the schools they worked in. To offset these limitations, the Ghana Education Service has instituted a collaborative intervention with the Ghana Health Service to conduct disability screening for in-school pupils at the beginning of every academic year (my first day at school). This is envisaged to improve teachers’ support to children with special needs.

## 4 Discussion

We set out to develop insights into the landscape/environment for raising/parenting CwSN needs in Ghana. Given that CwSNs and their caregivers encounter diverse experiences, it is very useful to develop a better understanding to allow programmes and policies appropriately target children with special needs. Overall, the findings reveal multiplicities of experiences regarding the parenting of CwSNs. These experiences highlight challenging systems and infrastructure for supporting parents who have children with special needs. The accounts of participants hinged on the timing of disability, social and health services for the detection of disabling conditions, community perspectives on CwSNs, positive narratives of parents on parenting CwSNs, challenges and educational and health services for children with disabilities. These are discussed within the socioecological framework, highlighting the relevant connections with participants’ voices.

Firstly, almost all our participating parents were clear that they did not have adequate knowledge about the timing/onset of their children’s disabilities and for those who did, the depth of suspicion seemed palpably low. At the individual, inter-relational and community levels, the capacity to detect signs of disability or potentially disabling conditions may not be sufficient. Parents and caregivers may adopt a wait-and-see attitude at the onset of potentially disabling conditions. However, by integrating early screening programmes into health and other social services, adverse events may be detected early for interventions [52]. Also, the capacity of health systems to early detect risks of disability is strongly connected with the state of public health infrastructure. In settings where these facilities and services fall short, it is more difficult for parents to receive/obtain such services [53,54]. Where it is possible and there is no known medical intervention to rectify and remedy the same, pregnancy termination may be suggested. However, this is subject to parental consent. But as one of our cases demonstrates, even under medical advice, parents reserve the right to accept or reject counselling and subsequent termination of the pregnancy. Research documents a nuanced basis for which people reject or accept medical recommendations. More than four decades ago, Becker and Maiman [55] underscored health beliefs (personal estimation of vulnerability, seriousness of a medical condition, and faith in the efficacy of intervention), perceptions of psychological and cost of recommended action, provider-patient relationship and social influence or social support systems. Cameron [56] affirmed the earlier assertions with similar research findings.

The notions of community members toward CwSNs were mixed with positive and negative narratives. On the positive, some parents recalled being encouraged by interpersonal/family networks to invest in the capabilities of their children without being distracted by the state of the children. Parents’ accounts show that this is an area many thought they were deliberate and faring well. The positive stories were connected with a certain consciousness of the innate potentials of all children, regardless of their (dis)ability status. Stories in this light are gratifying within the rights perspective of disability. Rather than approaching disability with neglect and viewing it as an obstruction, recognising the value and potential of CwSNs is a positive step towards inclusion and equality. These notions reverberate strongly in recent international development discourse which asserts the human rights and capabilities of differently-abled persons [57]. Investing in the development capabilities of persons/children with special needs reckons positively with justice demands associated with disability [58].

Unfortunately, parents with CwSNs recounted many negative notions and expressions towards them and their children. Parents (especially mothers) were viewed either as the “cause” (often due to supernatural punishment for an offence) or as children being agents of disruption to their parents. Whichever way one views these misconceived interpretations of causes of disability in children, it signifies engrained norms of seeing CwSNs as different from other children. Such views about CwSNs attune with Foucault’s [59] “othering”. By describing CwSNs as “other”, it voids any value persons described in that sense. In some Ghanaian communities, projecting CwSNs as “others” has in certain instances, led to the killing of infants with disabilities [60].

Where parents are alleged to be the cause, images of stigmatization and discrimination to themselves as well as the child may be enduring scars [61,62] to the extent that the incidence of a child with a disability has strained some interpersonal relationships, including marriages, with women as the victims of accusations. The gendered interface of parenting burden and attribution of misfortune weighs heavily on women as observed in our findings. It reflects the embedded extent of patriarchy in these environments with women as prime suspects of the cause of misfortune due to witchcraft, either exercised directly or indirectly through the kin of women [63,64]. Under these circumstances, women who have children with special needs may be threatened with divorce or abandoned entirely by their male partners. Even though these beliefs and practices are decimating, the fact of it emerging in our relatively small sample is indicative of the existing scale of these beliefs. Amidst these circumstances, it is noteworthy that the Social Welfare Department, through routine community outreaches, is reaching out to parents with CwSNs about the rights and prospective value-addition regardless of disability. In doing so, educating parents to bridge the gaps between psychosocial and medical beliefs of disability will be valuable.

Howbeit positive these realizations are at the individual and interpersonal level, community, institutional and policy/macro level systems are critical to translating individual/interpersonal visions for CwSNs into tangible outcomes. In developing the capabilities of CwSNs, education (especially, formal) is indispensable. It provides considerable opportunities for increased access to employment, social and other services as well as improved appreciation and demand for their rights. For parents and the community, continuous education on the medical and physiological causes of disability must be provided. This will improve early suspicion, diagnosis, treatment seeking and acceptance of children with disabilities. This can bolster parents’ attachment and positive parenting experiences.

Although the findings contribute to stakeholders’ perspectives on nurturing CwSN in Ghana, there are some limitations of the paper that must be highlighted. First, almost all the parents interviewed were mothers because a major selection criterion was being the primary caregiver. Given the gendered nature of the childcare economy in Ghana, the use of primary caregivers as selection criteria disadvantaged fathers and relegates their perspectives on caring for CwSNs. This, notwithstanding, the perspectives of mothers offered an in-depth understanding of the salient issues since they are directly responsible for the day-to-day care of the CwSNs.

## 5 Concluding remarks

This paper provided insights into the parenting of CwSNs in selected Ghanaian communities with evidence from parents, community leaders, and social services providers. The findings highlight the apparent lack of or limited health and social care services to drive early identification of disabilities or disability-causing risks for children. We show that parents are cognizant of the special needs of CwSNs but the spaces (economic, social, and emotional) for raising CwSNs do not seem optimal. In consequence, women/mothers often bore the brunt of giving birth to CwSNs to the extent of straining social relations including marriage. Social services providers, particularly the Department for Social Welfare, must double-up current efforts to educate parents and community members alike about the need for building and investing in the human capital of CwSN as much as they do for all other children. To help in the early detection of potential birth defects, the Child Health Department of the Ministry of Health is called on to institutionalise a care model for the 4Ds–birth defects, diseases of childhood, deficiencies, and development delays. The social welfare and other child-focused departments can come in to provide synergized services and support.

## Supporting information

S1 File (PDF)
