# Supplementary material for: Experiences of parents and stakeholders in caring for, and supporting children with special needs in Ghana
Source: PLoS One. 2023 Mar 3;18(3):e0281502. doi: 10.1371/journal.pone.0281502 (PMC9983829; doi:10.1371/journal.pone.0281502)
Supplement: S1 File — (PDF) [file pone.0281502.s001.pdf]

M: **ok, madam, thank you so much for allowing us this time to have our conversation with you. We are about starting our discussion. Please can you describe to us the general work of your institution?**

R: thank you. The work of my institution is, one; to monitor and supervise at the KG level, that is, from age 0 to 8; and also to give reports to my director at the end of every term. Also to report issues, my observations, challenges in the schools.

M: **so that is the general task.**

R: yes

M: **so what is your role?**

R: my role is to visit the schools, sits in the classrooms, see to it that they teach and also advise teachers on how to handle kindergarten pupils and how to go about their lessons; the use of TLMs and other things to help the children. You know at the KG level, at that age they need some special care and our system we don't have teachers who have gone for that kind of training. So it is my duty to organize in-service for the teachers on how to handle the KG people.

M: **ok, please in relation to children and adolescents, what role does your department play?**

R: as I earlier on mentioned my this thing is from 0 to 8

M: **so for the adolescent you don't do any direct job with them**

R: no

M: please so tell us about some of the services you provide to parents, caregivers and children.

R: with parents, I think if there will be some kind of education, to educate them to understand how they are going to handle their kids, you know, some parents are there, in our homes, children they have problems on the part of their learning and we don't understand. Maybe we have some who have fast learners and others are very slow and in our local homes when we see such a thing we say, "oh, this child is brain dead, doesn't know anything, is stupid, the is in KG 1 and this is in KG 2", and then that child is not able to say the numerals and the alphabets, and a child in that same class can do that. Some too can identify the colors and other things and some too cannot and when it comes down to it they will say this child is block headed, and it is not so. We don't have any child who is block headed.

M: so what training do you give to the parents with regards to this issue?

R: since I reported as the coordinator, we haven't had any. Maybe God willing next academic year...we had a workshop on such issues. There are certain times we have to invite the parents to our classrooms to also teach them music and dance.

M: you teach the parents?

R: they teach the children.

M: you invite some of the parents to teach the children?

R: yes, that is what they are now bringing in. So we will be organizing workshops, next academic year.

M: so what are some of the workshops you want to organize for some of the parents? Can you mention some of the topics that you will be discussing?

R: like, just as I mentioned, you know sometimes they are good at music and dancing

M: the cultural dance?

R: yes, we will invite them to come to the classroom then teach the children; because at that age they want activities.

M: do you also have programs to sensitize parents as to how to handle violent children?

R: [00:07:08.05 inaudible]

M: **based on your experience working in this community, tell us about your observations on how adult to parent child relationships are in this community.**

R: I don't come from this place.

M: **and you don't live in the community too?**

R: no, I don't live here

M: **so you have not heard anything as to how the parents treat their kids?**

R: with that, some children because of peer group influence, some children will not go to school; they are in town doing things that are not good; like taking drugs. Some parents have conflicts with children who have joined their peer groups who are drug addicts and let me use this word, excuse me, some prostitution; you know, our girls in the JHS, at their level, they need one or two things to keep themselves neat and if their parents are not able to provide, they go in for boyfriends that can help them. For example, some are there, their parents cannot provide them with pants, I think that way they go after boys to get what they need to keep themselves neat. And the boys too, these days, they say "me I am a guy", when they take the weed, cocaine, tramol, they behave in a way, you know, at that age they admire some of these things and you see that they join that group and then they also become addict to those things, through that they have conflicts with their parents. And even in our schools we have some challenges. When they are taking in those things they look at the teacher like some small boy.

M: **ok, thank you madam. Our next question is, what intervention do you have in streamlining or aligning parent/guardian child relationships that are inconsistent with child welfare environment?**

R: hmm, the girl child coordinator, there was a case they reported to her and she made a follow up to the parents. That was about teenage pregnancy. At the end of the day they counselled them. You know, when it happens that way, when they are pregnant and that kind of this thing they don't go back to school, so the coordinator has to go back and counsel them to continue with the school till...

M: **do you work hand in hand with the girl child coordinator?**

R: yes, we even attended a workshop together, that was, last march concerning girl child.

M: **ok, so on the same question, what intervention do you have in streamlining or aligning parent/guardian child relationships that is, parents who have children with disability that are inconsistent with child welfare environment?**

R: on the part of disability they refer to the parapathehic officer, they handle such cases.

M: ok, in the first place what are some of the bad practices that are done against children with disability?

R: the stigma; and then when we are building our schools we don't build access, like big, big stairs where they can access into their classrooms; and they don't also have for the blind, the white stick and the cripple, they use the wheel chair or clutches, they don't have such things to help them to move.

M: so what is your office doing about it?

R: as for my office, there are a lot of challenges so they refer to the district assembly to help.

M: please how do you assess the type of coordination that exists among child and adolescent related institutions like the department of social welfare, ministry of gender and social protection, division of family health and the others, the DOVVSU, what is the coordination that exists?

R: when was that, by then I was still in the classroom, and there was an accident that occurred at Ahwiean, a student there who was selling pure water and a car knocked her down. The whole leg was condemned and then girl child coordinator took the matter up and the case was referred to the social welfare and then they took the matter up. They took care of everything. It looks like the legs were amputated and the woman did very well. They even donated an amount to her which I have forgotten. So we work hand in hand.

M: so do you share information?

R: yes

M: do you normally work to discuss issues?

R: since I resumed work here, there has been nothing like that but as a teacher I sue to hear and the girl child coordinator too and I are friends so I use to get information from her; and when there are such issues they report to the social welfare and the DOVVSU.

M: ok, madam please, could you tell me something about the core issues in monitoring and supervising children related organizations in terms of compliance to national or international regulations and rules?

R: you know, when you re to go out on monitoring and supervision, you need some funds, but we don't have; and there are some places [00:18:06.12 inaudible], we don't have the means so it makes it very difficult.

M: I can't hear you.

R: I said monitoring and supervision you need means of transport or you need funds to take trotro or taxi to the places that you are to do your monitoring and there is nothing like that, we don't have so it makes the work difficult. Even though we are always willing to do but we don't have the means. So there are certain places we cannot go.

M: why?

R: the means

M: the place is not accessible or what?

R: yes, it is not easy. At times to what you yourself you are to us to do one or two things, we sacrifice...and then you have to get it on regular basis, we don't get so it makes the work difficult.

M: please can you describe what makes children unsafe in this community?

R: we have financial problem in this community. If you look along the road, there are these children selling sachet water, child labor. Some carry heavy load from the farm at their age. It is a problem. And at times when you go to the school, what they will even eat, no uniform, no sandals [inaudible [00:20:16.12]. Even they don't have exercise books, pens, pencils to write with. The teachers will do their best by inviting the parents to come there to explain things to them, "when I go home I will give her money to buy it", but they don't do it.

M: what about social issues that happen in this community that doesn't make the community safe for children?

R: some of the things are drug addicts and then some of the parents too are drunkards; and they end the children to buy alcohol for them and they do when they buy they try to taste it and by doing so the child too will become addicted to it and it will come to a stage, the child cannot do without taking the alcohol or the drug.

M: any other issue?

R: and there are a lot of these games something, the gambling, they have to pay money before they are allowed to play and if they don't get the money they have to steal.

When you go round our community you will see those things, I don't know the name, some kind of game they put in money to also get something out of it.

M: **any other issue?**

R: teenage pregnancy too in the community because some of the girls the parents cannot provide their needs; and so they have to also go out to look for boyfriends to have affair with them before they can provide them. And if they are doing so and they don't take any preventive measures, at the end of it all, they become pregnant and it becomes a problem for the family and the community. Just as I said earlier on, even petty, petty things like pad and panties parents cannot provide and it looks as if it is a challenge, "you see this my friend the daughter she is this, she is doing this for the mother and you are here, you cannot do anything", and the girl is not working, she is still schooling, how is she going to get that money to I mean, maybe the mother want a funeral cloth, and the friend's daughter has been able to go out, take a boyfriend somewhere, get the money to do that for the mother; so that girl too will be compelled or forced to go out to also have a boyfriend to get something to help herself and the mother and the siblings.

M: **ok, please do children with disability feel safe in this community?**

R: they are not all that safe. Some are locked up in the rooms because parents feel that "if I take him or her out, they will say you have given birth to such a thing", they don't even consider them human beings. The mother feels that she will be stigmatized because of that they lock them in the room and the poor child will just be there suffering. There are a lot of challenges. This time we have inclusive education but they don't do it.

M: **is it the parents who don't do it or the school?**

R: the parents don't take them to school. Some of them can perform very well but because they are locked up in their rooms, how are they going to learn? They cannot get access to education.

M: **please can you tell me your perspectives on rehabilitation services for children in this district? Are there rehabilitation centers in this community?**

R: I learnt there is one in Jamasi.

M: **so what can you say about the rehabilitation services?**

R: they make the disabled independent. They help them to learn a lot of handiworks so that they can also do something to help themselves and their families. The government and the NGOs also help.

M: do they have care homes where children who are undernourished and staff like that go to? You said there is one at Jamasi

R: but I have never been there but I have heard that there is something like that over there.

M: please what your view on formal and informal child protection services available in this community? So let's talk about the formal one first.

R: the formal services we have social welfare, we have DOVVSU, at education service, we have the early childhood coordinator and the we have the guidance and counselling unit, and then parapathethic officer. So when there are issues concerning disabled, they refer...

M: this one is not only disabled, all children.

R: they refer to any of these services

M: if there is an issue between a child and a parent or a child and an adult, or even a child to child issue...

R: they refer to the officer [00:29:30.05 inaudible] to see the director. If the case is concerning social welfare, they refer it to them.

M: then what about the informal services?

R: we have assemblymen and women and then the [00:29:50.20 inaudible] they refer to the chief and then the...

M: ok, what type of case or cases would you use the formal system to resolve?

R: if a child goes to school and gets a problem, where the child is wounded or the child is beaten by a teacher, when the teacher beats a child and the child is wounded, the case would be referred to the circuit supervisor...if the child is in KG, then the circuit supervisor will also refer it to the early child coordinator and the coordinator will go to the director, then the director, then the director will sit with them and [00:31:06.11 inaudible].

M: **how about the informal structure? What issues would you use the informal structure to resolve?**

R: informal structure, when the case is [00:31:30.21] we call on the assemblyman.

M: **so what kind of case?**

R: if there is a child who is attending school and at a point she becomes pregnant, you know, when a child becomes pregnant she feels shy to go to school or the parents will even say, "don't go because of your pregnancy", such cases we report to the assemblyman or woman, then they sit down with the parents and talk to them. You know this time when you are pregnant doesn't mean you can't go to school, you can go to school and give birth, after birth you continue your education. And then those too who have been smoking and those kinds of things, we also report to the assemblyman and the chief [00:32:57.27 inaudible] and see how best...

M: **ok, please can you tell us about a case you used the formal system to resolve since you assumed office?**

R: since I resumed office there has been no case like that. I resumed in January this year, 2019 so this is the fifth month in office and I don't have any case like that.

M: **how about the informal structures? We also want to know about a case that your outfit adopted the informal scheme to resolve?**

R: I can't think of any.

M: **do you have any comments to add to it?**

R: this thing you are doing is a nice program. We pray and hope that God should continue give you the strength and the financial support so that you can help the little ones. Some of them actually, they are having problems.

M: **ok, thank you very much madam. I really appreciate your time, your patience to answer these questions. Thank you.**

M: **Ok sir, please how old are you?**

R: 32.

M: **And then how many years have you been working in this community?**

R: Me I work in Kpandai.

M: **Ok, but you work here too.**

R: No here we don't have any clinic here.

M: **Ok, but you come from here and work in Kpandai.**

R: And work in Kpandai.

M: **Ok. So how many years have you been working as a Health…?**

R: From 2012

M: **2012 to now**.

R: 2012/2013, I should say is 2013.

M: **Ok, so your level of education.**

R: My level of education now, I completed what do you call it Kpenbi HAS; Health Assistant, Clinicals at Kpenbi. But at currently I am schooling at University of Health and Allied Sciences. I am doing a degree course there.

M: **Ok. So what do you do in the hospital?**

R: Mmm. In the hospital, as a Health Assistant Clinical Nurse.

M: **Ok. So what are some of the…**

R: What we do is that, we assist; ok we assist the degree nurses. That I what I always do, we assist the degree nurses in the hospital.

M: **Ok.**

R: Ok, that is what, our work is. If there is anything that we cannot do; then we call on our superior.

M: **Ok.**

R: Hmmm. But because of the lack of nurses too, sometimes we also do them on our own.

M: **Ok.**

R: But mostly now that the degree and diploma nurses are coming.

M: **So what are some of the activities or practices that you have in that facility about mother and children?**

R: In the facility there is a nutritionist ok, because in case they bring the child, and there is, you have to look at the cardinal signs. To see that, may be this child is malnourished. So you have to call a nutritionist. So that they will come then we have to give them do the CD; and then this is what the nutritionists, they do, then they do the calculations they just give us they go. Ok plus the measuring cup; that you have to give the children.

M: **And what about the mothers, is there anything?**

R: Hmmm, mothers too we give them education on how to take care of the children, because sometimes the way they expose the children to the environment, we talk to them and then; we also educate them on exclusive breastfeeding.

M: **Ok so from your professional perspective or from your professional view of things, what is your view about the health status of the children, you normally attend to?**

R: Hmmm, some of them to be sincere after the treatment and then the feeding by the mothers, you see that they are fine. But except that a few when they go home ok, when they go home because mostly is very difficult for the follow up. Hmmm.

M: **So what mostly bring them, what conditions are they mostly inside before they come for you people to advise them and give them the treatment, when they bring them the children in what condition are they mostly in?**

R: Hmmm when they come, most of them like this they are malnourished.

M: **Ok.**

R: Others too have, what do call it abnormalities. That they cannot work, one that I witnessed, because our facility here cannot take them so they have to refer them to Tamale Teaching Hospital for further management.

M: **Ok. So why do you think that most of the children are malnourished, what are the reasons, what causes the malnourishment?**

R: Hmmm, nutrition, it is the feeding.

M: **Hmmm.**

R: Because they don't get the proper feeding. Then some of them they may have you know these our local foods, they have them: But how they will blend them together so that they will be able to get the necessary nutrient.

M: **Ok.**

R: is another factor.

M: **So what about the children with disability you were talking about, what causes most of the disabilities and those problems?**

R: Aah, the disability?

M: **Yes.**

R: Hmmm. Some of them, because, related to these genetic factors. Some of them are because of these genetic factors. (Noise from background); but others too they delay them, especially those who have those either malaria or some of them have infections. So when they delay them, instead of first reporting them to the hospital; they said they want to try it on the, all the time you see that is disability.

M: **What are the major health related problems of children from 0 to 19 you attend to in your facility there, or where you work?**

R: Hmmm, mostly you know, this our locality the major one is complicated malaria. That you can talk about. Complicated malaria because of the environment, mosquitoes, some of them they don't clear the environment. And some of them too they don't sleep under treated mosquito nets. Mostly, majority of them it is malaria that affect them.

M: Ok. And what are the major; tell us tell me about any form of engagements you have with the parents of the children with peculiar health concerns? The ones you have mentioned malnourishment and the disability those kind, the peculiar, peculiar health conditions. What engagements do you people, the health workers, or especially you and the parents engaged; what engagement or form of engagement do you and the parents of the children of the children with have?

R: Especially related to the malnutrition ok, erh someone, there was a one that came. I have realised that one of the reasons why the child was malnourished, the parents after the delivery, they lose the parent.

M: Hmmm.

R: The mother was not there. So what happened was that, it was the grandmother, are you getting it that was feeding the child. You know this our area.

M: Hmmm.

R: So, when they were doing that, then the child started having this malnourished thing. So when they came what we told them, we asked, when we asked them, you know initially they denied. But later on when we probed further we realised that it was the grandmother that was feeding the child and they were, you know children of that age, a day old or one week old. Then you will be feeding them, are you getting me, their immune system is very weak. So what we advise them, is that, they shouldn't. In case they have those health problems, they should report to the hospital. And what also we did was that anytime some of them this formula (noise from background). You know this food supplement; we also educate them especially about hygiene. They have to keep the bowls or those equipment for the food very clean, so that if not, you will don't waste your money to buy those supplements and come and be infected. So these are some of the things we told them. But we also told them that in case of anything, we give them days that they have to visit. But sometimes most at times it is the public health nurse. They normally visit them.

M: Do you organise any seminars for parents or any other interventions, programmes for them, the mothers?

R: No, no. I can't remember, no.

M: Ok. Can you describe to me some typical interventions you provide for the parents whose children have special need? Those with disability and all those critical conditions, do you people have any typical intervention you know that oh; this is what we do for this category of people. This is what we do for this category of parents with this kind of children, in the hospital everybody knows that we do this for mothers with special children. We do this for this; in your facility do you have those kinds of typical interventions.

R: No I don't think so. We don't have…

M: So mothers who have children with special needs, what do you do to them?

R: Like, that is what initially I said.

M: Hmmm.

R: Like some of them who have psychiatric problems, are you getting me, we refer them to the psychiatric unit.

M: Ok.

R: We refer them to the psychiatric unit, and they will take care of them. Then we have the psychiatric nurses who are also there. They give them the medication and then they ask them to come. And as they come we also monitor them to see whether there are changes. If they see that they can also refer them. But actually they have been called, there is one man June-July; he has been doing well.

M: Ok.

R: He visits them, he normally comes here. He brought so many of the cases here; the adults, who are having that psychiatric problems. So he comes here, sometimes we have to bring the fellow. Then we give them the drugs. I can see that the woman at the back. So they are doing well.

M: Ok. So what do you think health workers like you need to support parents in nurturing and raising their children?

R: Yes what we need is, we need if not workshop. Ok, then we should be allowed like we have the time we will serve; that oh after this number of years we have to further. Are you getting me?

M: Yes.

R: So that you can also acquire knowledge, more knowledge to calm whatever we you have learn some of the things that we lacking here. When you calm I think that it will help the community.

M: So…

R: Nurses, there are so many nurses there. They need like what we are saying, specialised people. Ok that if you come to paediatric, currently there is one who is doing (noisy) but I don't know I am hearing that there incubators and so many things at the hospital there, that he has not come. But I heard that he will come. Because when he comes it will help us because in our facility, you know most of the big facilities the units, they separate the units. Sometimes when the mother delivers, the mother unit is separated from, unless may be they want to breastfeed. But our place is not like that. When they are sick we can look at may be 1 week old or 1 day old with someone who is 5 years in line.

M: Hmmm.

R: If there is any infection, may be cross infection. Are you getting me?

M: Yes.

R: So, we need people who are specialised then we also need what is the, how do they call the name? Erh, they have to give us more buildings. Are you getting it? Because the space is very short, but the EP currently, they have a maternity ward.

M: Hmmm.

R: We need more infrastructures.

M: Ok, erm so how do you assess your capacity, you yourself? How do you assess your capacity in delivering child welfare services?

R: How do I assess…

M: Yeah, your capacity. Your ability to deliver the welfare of children services as you take care of the children. The services you give to the children, you yourself your capacity, how do you assess it?

R: Hmmm, yeah to be sincere, I think we are doing great. But like I also said, we are not 100%.

M: So what are the factors that make you think that you are doing well?

R: Yeah because the reason why I said we are doing well is that when they come, any treatment that we give them. Are you getting me? When they go, they don't come back with the same condition. Are you getting me? Just that a few, like I said a few, that when you tell them what they are supposed to do; they don't follow and then come back with the same situation.

M: What are some of the things you always tell them to do; examples of those things?

R: Like initially I said like, exclusive breastfeeding like this. Are you getting it? That is what they are supposed to do, you tell the mother that she has to breastfeed the child except exclusively. But some of them who do that, they will come and testify that this is my child. This I have done and what the nurse have told me, I have benefited. The child from maybe has not suffered from it. Some of them you see that when you compare them there is much difference. Very vast difference; you will see this one will be having weight, and the other has not been practising, that is exclusive breastfeeding; diarrhoea, some of them start vomiting (Noise). If you look at the weight, the child will lose weight.

M: Ok. So when you look at a child on a typical day, how are you able to identify that he or she is malnourished or not? This child is counting or wasting; how are you able to identify that?

R: You look at, immediately they bring the child you look at the head. Ok.

M: Hmmm.

R: You can see that this one, sometimes you have to look at the skin. Some of them even have oedema. Then they are picky, are you getting me? Then we know that this child is malnourished. These are some of the cardinals we use to identify.

M: Ok, which area do you think you need much improvements or you people needs much improvements in delivering child welfare services?

R: Hmmm, please come again.

M: Which areas, you've just told me you able to identify malnourished children…

R: Ok.

M: and all those things, or your abilities; which areas do you need more improvements, to improve upon your service?

R: Like I have told you, like I was talking about; I was talking about areas do we need, is it more training or…

M: You've just told me some capabilities you able to use to identify children who are malnourished and other sicknesses; those are your capabilities as a professional. So as a professional, in skill and you think you need more improvements, of which improvements?

R: Ok.

M: Additional…

R: I think erh, what is the name? The food supplement…

M: Hmmmm.

R: Is like if, plumping nut like this, is a very long time I have seen it. But when we came 2013, it was there. Are you getting it, is a very long time. You understand it, so I think if they can provide more of that. That is what you have to use to take appetite. So I think it will be very good (noisy background).

M: Ok, so the plumping nut, you've been talking about the plumping nut, you said need more plumping nut, what other thing apart from that (noisy background)?

R: That is all. (Taking some moments before he clarifies); like we also need what is the name prescribers.

M: To describe what?

R: You see some of them when they come, like I only use malnourish, anything, because it could be a condition; anything that has led to that; so because of this we have to get a prescriber, the person who will prescribe the drugs. So as we are managing with this, we can also be treating. This malnourish like this as we are managing, we can also be treating alongside the feeding.

M: Ok. Thank you very much for your time. I don't know like all that we have said, oh ok, do have anything you have to say or any additional comments, anything, ok?

R: What I will say is that, I think we thank you very much. We just hope that what you have asked, the information I have given you; it should be able to benefit us. Because most at times they just come like this and then they go back and then we don't know what they are gone to say; then the positives that is bringing; are you getting it? Erheh, like is good; so that it will help all of us. Because health is wealth.

M: Yeah.

R: If you not strong, then you can't do anything.

M: **We also want to thank you very much for the time you have had for us, we appreciate with small credit, listen…**

R: For.

M: **For you.**

R: No.

M: **(Laughing). No it's ok.**

R: Erh.

M: **We are just appreciating you people for time wasted for us. Thank you very much.**

M: **Interview ended at 4:52.**

M: **Good evening sir!**

R: Good evening

M: **Please can you describe to me the general work of your institution?**

R: We take the lead in integrating the vulnerable, the disadvantage, and excluded in the main stream of development at the district level. We do justice administration, community care, adult education, and many more. We work with people in their communities to make sure that the vulnerable is main stream into the development agenda.

M: **Please what is your role as director in this institution?**

R: I have officers under me. They report to me and I also report to the DCE through the Co Coordinating Director. I make input onto the composite budget into the Assembly , take critical decision on the vulnerable and anything on government policies concerning gender, children and those who are not privileged in the society, we support them. We do adult education too.

M: **What role does your department play in relation to children and adults?**

R: Like I said, anything concerning children we are responsible. Example: Child Abuse, child marriage child neglect and the rest. If there is any case even if the child is in conflict with the law, we have to go to court to defend him and he will not be mixed with the adults in cell or prison.

M: **Can you tell me some of the services you provide to parents care takers and children?**

R: we provide many services. Example: If the father is not responsible, we go in to make sure the father become responsible.

M: **How do you do that?**

R: Normally it happens when there is divorced or separation and the man don't want to take responsibilities of the child, we usually look at the man's income and work out portion of it for the child and forced.

M: **Do you offer counselling to those that drop out of school?**

R: Yes, we offer guidance and counselling too. Certain time we met them in group to discuss some of the dangers of life, this is guidance. And when a child has a peculiar from, we them individually to find out and suggest solution.

M: What about the rehabilitation of abuse children?

R: Though we don't have rehabilitation center, but we do it. We have care givers in the communities that handle abuse children. We can't be present in the communities all the time and some of the issues occur at night or weekends. When it happened our care givers take control of the situation before we are informed.

M: What about counseling for violence parents?

R: We usually invite such parents with a letter and schedule a date for the discussion of the issues.

M: Do you prosecute offender's parents or children?

R: We do but that is the last resort. A case like paternity, we try to get the man to accept the pregnancy even if it involves going for DNA test.

M: What about stigmatization of children with disabilities?

R: That one we do general education in the community.

M: Base on your experience working in this community, can you tell me your observation on issues concerning adults and children are resolved? Parent - adult or child -child relationship?

R: Some parents have good relationship with their children but majority want to take decision for their children. Only few cases children are involved in decision making.

M: Can you give an example?

R: If you are buying a Christmas dress for your child, some parents will go and buy any dress they want. Few will ask the child what he or she wants.

M: Do you think the relationship is cordial or hustle?

R: We have less cordial and more hustle relationship

M: What intervention do you have to streamline or align parents or guardian in child relationship especially those that are inconsistent with the child welfare and environment?

R: We have a district child protection committee that is responsible for the child welfare. They sit on those cases. We also have community child protection committee. They respond to Child abuses in the community level and report to us.

M: What about intervention of parents or guardian child relationship?

R: It is the same. The system take care of all those concerns.

M: What about parents who have disabilities?

R: When there are issues we intervene but when there is no issue we give general sensitization.

M: How do you asses the type of coordination that exist among the child and adolescent related institution?

R: There is cordial relationship however from where I sit there may be an information gap. Some people don't know how to seek redress and others may not know where to get certain information.

M: Are the Department of social welfare, Ministry of Gender and Social Protection, DOVSU, and you come together share information?

R: Mostly and NGO bring us together. We don't have DOVSU in the district but we have it in the Regional. When they want to carry out their program at the district level, they pass through us. Most of these activities are supported by NGOs like UNICEF, Plan Ghana

M: That is mean you have clearly defined role and tasks?

R: Yes

M: Does is mean dovsu, ministry of gender and social welfare all have their roles and activities?

R: Yes

M: What about reporting?

R: I said earlier that I report to the DCE through the district coordination director and copy my regional director.

M: Can you tell me the core issues in monitoring or supervising children related organisation in terms of compliance to national or international regulations and rules?

R: We have many NGOs that supposed to register with us but some of them don't register.

M: Why?

R: They have various reasons. Some of them don't want us to know their operation and others think the procedure is difficult. To register, you need to bring a certificate of incorporation, commencement of business and your constitution and give us a profile of your organization. What expect to see in your profile is the vision, mission, objectives, goals, program areas, and board of directors, staff and any other useful information. We will conduct social investigation of the NGO and give you a respond. We will give you a form to fill. You write an application and addressed it to or the director. The NGO should have a logo of the NGOs

M: Are they some of the issues that make it difficult for me to monitor or supervised

R: we will not be able to do effective monitoring because some of them don't register with us.

M: Is there any other things you want to add?

R: They don't want to share some of the things they do

M: Why do you say so?

R: Some of them have dubious things they do

M: In this district what makes children unsafe?

R: In loop meant and some cases female genital mutilation and the last one is child marriage.

M: How do you handle all those things?

R: As for in loop meant, when we get you we stopped it and rescue the child and we do sensitization

M: Please can you tell me the rate of child marriage, female genital mutilation and in loop meant

R: Female genital mutilation is about 3%, child marriage is about 25% and in loop meant is about 2%

M: A part from these issues can you also talk about perspective on rehabilitation for children in this district?

R: What do you mean?

M: Do you have rehabilitation center for children in the district?

R: We don't have rehabilitation center but we have rehabilitation services

M: How do you do that?

R: When we have teenage pregnancy issue, we interact and council her on is of continuing her education or learning a trade. I even have a picture of a girl reported to me, she said she doesn't want to go back to school.

M: Did you ask why?

R: We investigated it and realized even if she is sent back, it will not yield good result. We assigned her to the guidance and counseling's coordinator of the GES because she was in school. We gave her a sewing machine through the assembly woman to go and learn. If she wanted to go back to school, we will have allowed her to go. We took money from the man who impregnated her and the father wanted us to give the money to him but we refused to him the money.

M: What about access to therapy, do you sometimes put them on therapy?

R: We have different types of therapies. Mostly if you need any therapy you have go to hospital. We have natural therapy and drugs or machine therapy. The therapy we give is counseling.

M: Do you have a care home in the district?

R: No, we don't.

M: What about probation services?

R: We render probation services, even though we don't have a court, we go to Wa for the services.

M: Do children with disabilities feel safe in this community?

R: They feel safe

M: What is your view of formal law or informal child protection services available in this district?

R: I told you we have district child protection committee, the office of social welfare in community development. We are part of the child protection committee. We selected

traditional rulers, somebody from GES and health to form that child protection committee. We also have community child protection teams. The informal one the family system the extended family system.

M: **What type of case would you use in the formal systems and what are those would you use in the informal structures?**

R: All the cases you can use the formal system and can also use the informal system depending on the gravity of the case, for example if husband and wife having a problem sometimes the issues they raise are minor issues so therefore you can refer them back home to resolve those issues, but some of them too they need some technical advice. There was an instance a couple to table an issue. The woman was saying the father do this and that. He goes to drink and come and make noise in the house. Those things are matters at home.

M: **Is that formal?**

R: That is informal. There are also some issues

M: **Like, can you give me an example**

R: There was an issue like the man is irresponsible and works in a different place. It was like he was running away from the woman. He doesn't come back during weekends. Like he has some advantage. In such a case you will need to know what the woman does that keep the man away from the house? You will also need to find out if there is something keeping the man from coming home. The local people can do that investigation so that you can talk to both of them.

M: **Thank you very much for the interview. Is there anything you may want to add?**

R: No

M: **Thank you very much.**

M: This is Subi I am in his office to have an interview with him the date is 3rd May 2019 and the start time is 04:04pm. Good evening sir.

R: Good evening.

M: Please can you describe to me the general work of your institution?

R: We take the lead in integrating the vulnerable, the disadvantage and the excluded in the mainstream of development at the district level. We do justice administration, community care, adult education and many more. So we work with people in their communities to make sure that the vulnerable is mainstream is integrated into the development agenda.

M: Ok. Please what about your role, you personally, what is your role as director?

R: Well, I have officers under me that report to me. Then I also report to the DCE through the coordinating director. I make the input into the composite budget of the district assembly and like I said, I take critical decision on the vulnerable and anything on government policies concerning gender, women, children and then those who are not privileged in the community of society we support them. Then we also do adult education.

M: Please what about in relation to children and.... what role does your department play?

R: I think I just mentioned that anything concerning children we are responsible. For example, abuse like child marriage, element, child neglect and the rest, we are responsible if there is any case. Even if the child is in conflict with the law we have to go to the court and defend the child. So that the child is not mixe3d with adults in the cells or prison. So anything concerning children, even if you send them to the cell or prison, he will be referred to our office.

M: Alright, please can you take about some of the services you provide to children, care givers and children?

R: We provide many services. If a father is not responsible, and he is brought, you know a mother too can be irresponsible but you know but mostly it is the men that are brought to our office so we make sure that we make them responsible.

M: How do you do that?

R: For example, normally, it happens when there is divorce or separation, then the woman is supposed to meet the man, so when that is not done, they come to our office and we are able to calculate based on the man's income, the portion that he is supposed to give to the woman every month and we are even to compel them to do that.

M: What about counselling for dropout students? Do you offer services like that?

R: You know we do guidance and we do counselling and when I say we do guidance and we do counselling normally guidance is done in a group or a focus group discussion or mass interaction. Like you have a bigger crowd, so you tell them generally the dangers that a person or a group of people can face or the dangers that they are exposed to and how they can face it. But when you

come to counselling that one is one on one, that one you cannot do group counselling. Even though maybe there is a term you can use but when it is group, it is guidance but when it is one on one that one it is counselling. So that one it means the person has a peculiar problem and you have to deal with that one specifically and that one there must be some confidentiality... so...

M: So can we go on?

R: Yes.

M: What about rehabilitation of abused children?

R: We do it but we dont have a rehabilitation center.

M: In the district?

R: No.

M: Ok.

R: What we do is that we have child protection committees in some communities and then when a child is abused, we have care givers in the communities that the child is supposed to be taken from the situation that the child finds himself or herself to the care giver. Normally the care giver is somebody who is trusted or who has a track record of good behaviour and respect in the community. So the child is taken to the care giver temporarily before the case is reported to us and then we go into the case. The reason is that we are not physically present in all the communities and some of these issues can happen at midnight, weekends, so that should be the firts point of call before we come inside.

M: Ok. What about counselling for violent parents?

R: Yeah, we do.

M: How do you do that?

R: When we hear the issue or the issue is reported to us. We invite them through a letter. When the person comes, we schedule a date with the person. When we finish the first session we schedule another date for the person, until such a time that you see that the person is getting ok, then we can stop.

M: So do you sometimes prosecute offending parents?

R: We do. But that is the last resort. Normally when they come, unless it is a criminal case, we want to resolve it. For example, if it is a paternity case, the man is trying not to accept the pregnancy that one we try as much as possible to let him accept it and then we end it there. But

if he is not trying to accept it then it means you have to take the person through all the processes. And you are likely to end up even doing a DNA to confirm. But if the person accepts, as much as possible we want to make sure that the person accepts and then you will just end it there and then see how best the woman can cope with life if she delivers, is she going back to school, what work is she doing and so on.

M: You do all that?

R: Yes.

M: What about stigmatization of children with disabilities?

R: That one we do general education. Mass education in the community.

M: Alright, so based on your experience working in this community, can you tell me about your observation on how issues concerning adults and child are resolved, or parent's adults or child-child relationship in this community?

R: Like what kind of relationship? I dont understand.

M: What I mean is that you have been in this community for some time. So how have you observed the relationship between children and adults?

R: Well it is... if you make a generalisation, some parents gave a good relationship with their children. But majority will like to take decision for their children. So normally children are not involved in decision making but we also have few cases where children are involved in decision making.

M: Can you give an example?

R: An example is if you want to buy a Christmas or Salah dress for your child, some parents will ask the child, what do you want for Christmas? What kind of dress or what shoe do you want for Christmas, but some will just buy for them.

M: So do you think the relationship is cordial or hostile?

R: That is why I said we have cases where the relationship is cordial. Then we also have some cases that are not cordial. But we have more cases that are not cordial that ones that are cordial.

M: Now what interventions do you have in streamlining or aligning parents/ guardian relationships? Especially those that is inconsistent with child welfare environments.

R: We have a district child protection committee that is responsible for child welfare. When there are issues of child abuse, they sit on those cases. Then when you go down to our communities,

we also have community child protection committees. In some of the communities, we are still expanding. The community child protection committees are also to respond to abuse cases that involves children at the community level. So that before they are reported to us. So like I said, because we are not present in all the communities, we try to put up those things so that the people can immediately report those cases to them before they come to us.

M: Ok. Wat about interventions for parents guardian child relationships?

R: I think it is the same thing because of you talk of parents or guardian child relationship, a guardian may be a person who has not biologically given birth to a child but taken custody of the child so, once these systems are there, they take care of all kinds of issues.

M: The second one is talking or parents or guardian who have children with disabilities.

R: Yeah, when there are issues we intervene, when there are no issues we do general sensitization.

M: Alright, how do you access the type of coordination that exist amongst child and adolescent related institutions?

R: Well from where I sit there is a cordial relationship, there is no problem at all. However there maybe information gab. Access to information, some people are not aware of certain things and some people see it and think it is some big place. But from where I sit, our doors are open and when they come we tell them but when we get any opportunity to meet them we tell them that our doors are open so if you have any issues you can come.

M: So like the department of social welfare, ministry of gender and social protection, DOVSU, is there information sharing between you? Are there times you come together, to talk about...

R: Mostly unless an NGO brings us together. Apart from that, there are instances where DOVSU comes, we dont have DOVSU in the district in the first place but there is DOVSU in the region and sometimes they bring their activities, like sensitization and other things to the district and when they are coming that one they pass through us. Most of those activities are supported by NGOs.

M: NGOs like?

R: UNICEF, Plan Ghana...

M: So does that mean you have clearly defined roles and tasks?

R: Yes.

M: So the social welfare has its roles and tasks? DOVSU same...

R: Yes.

M: Ministry of gender, same...?

R: Yes.

M: Alright, what about reporting?

R: You know I said earlier that I report to the DCE through the coordinating director.

M: Ok.

R: And copy my regional director.

M: Ok.

R: Or my regional directors.

M: Alright, can you tell me something about the core issues in monitoring or supervising children related organisations in terms of compliance to national or international regulations and rules?

R: What happen is that this time we have many NGOs that are supposed to register with us. But some of them dont register with us.

M: Why?

R: Well, there are various reasons why they dont register. Some are doing illegal things. So they dont want you to know about their operations. Some too I dont know whether they dont know that they are supposed to register with us. Others too think that the procedure they have to pass through is difficult. Because some will come and tell you they want to register, when you just give them a list of items they have to bring, and then they will tell you it is difficult. Sometimes I ask, what is difficult in all these things because the things are not... I dont see any difficulty in all the things that we ask them to bring.

M: Ok.

R: So there are various reasons why we dont want them to register. If you want I will just tell you want they need to bring for us to register them. You just need a certificate of incorporation and commencement of business, just a photocopy. You know when you are operating as an NGO you are supposed to register with the registered general so they will give you a certificate of incorporation and commencement of business. So you just give us three copies of that, photo copies, then you give us your constitution, I dont see these things to be difficultly then you give us a brochure of your organisation, and what we expect to see in the profile or brochure is your

vision, mission, objectives, goals, and program areas, board of directors, staff and any other useful information. Then when you give that to us, we will conduct a social investigation about your NGO and give you a report. Then we will also give you a form to fill, that one we call it profile form, then that is all, you just write an application, then address it to me or the director, then your application should have the logo of your NGO. Like your letter head, attached to these things.

M: So are these some of the issues that makes it difficult for you to monitor or supervise?

R: Where I am dragging to is that because some of them are not registered with us and most of them are also doing projects in relation to that, definitely we will not be able to monitor them.

M: Any other thing you want to talk about?

R: Yeah... and you know some of them too, they dont want us to collaborate because they dont want us to see inside.

M: What do you mean by the dont want you to see inside?

R: Like some of the things they are doing they dont want to share with you. So ....

M: Why do you think they dont want to share?

R: So like I Said some of them have dubious things they do, so they dont want to open up to us.

M: So, in this community, what makes children unsafe?

R: Well I am dealing with the district.

M: So in the district what makes children unsafe?

R: In some cases, elopement. Then we also have few cases of female genital mutilation. Then the last one is child marriage or early marriage.

M: Ok. So how do you handle these things?

R: As for child marriage and female genital mutilation and development, as for elopement, when we get you we stop it and we rescue the child. Then we do sensitization from time to time.

M: Can you please tell me the rate of the child marriage, the elopement a the female genital mutilations?

R: The female genital mutilations are about 3%, child marriage is around 25%, and elopement is also around 2%.

M: Now apart from these issues, can you also talk about your perspectives on rehabilitation services for children in this district?

R: When you say perspective, what exactly do you mean?

M: Do you have rehabilitation services for children in the district?

R: We have rehabilitation services, but we dont have a rehabilitation center.

M: In the district, but do you offer those services?

R: Yes.

M: How do you do that?

R: You know when we have pregnancy issues, and what we do is that the girl, we interact and council the girl to know whether she will like to go back to school or she will like to learn a trade. I even have a picture where a case we reported to us and then the girl said she doesn't want to go back to school.

M: Ok, did you ask why?

R: Definitely we investigated it and we realised that even if we send her back to school, it will not yield any results, because what we even did was that we assign her to the guidance and counselling coordinator of the GES. Because she was in school, so we bought this sewing machine and the accessories. This is the assembly woman. So we handed it over to the assembly woman to be handed over to the girl to go and learn and I think this is rehabilitation.

M: Ok.

R: So if she wanted to go back to school, we would have allowed her. The father wanted the money we took from the man. But we said no, we will not give him the money.

M: So you took money from the man who got her pregnant?

R: Yes, to buy these things.

M: What about access to therapist?

R: You know we have different types of therapy, so I dont know. Mostly, if you need any therapy, you have to go to the hospital. For example if it is stress, or depression, we cannot give any.... well we will work on it but sometimes the health people have some drugs that they will give, apart from the counselling that they will give. And then like I said we have natural therapies and

then these scientific or maybe machine or drugs therapy, I dont know the term to use. But the therapy we give is counselling.

M: Do you have a care home in the district?

R: No.

M: Ok, what about probation services?

R: Yeah, we render probation services, even though we dont have a court, they are now building a court for us.

M: So how do you render those services?

R: When there is a case in Wa and it involves our district, we come to Wa to render the probation services.

M: Ok, let me ask this before we go on. Do children with disabilities feel safe in this community?

R: From where I sit, I dont see what is making them unsafe.

M: Ok, so they feel safe?

R: Yes.

M: Alright, what is your view on formal or informal child protection services available in this district?

R: It is like sometimes we are repeating the questions?

M: Is that how you feel?

R: Because as for the formal child protection, you know I told you we have a district child protection committee, I mean that is the formal ones. The office of the social welfare and community development is there. Apart from that we have a child protection committee which we are part of. Because we selected people from traditional rulers. We selected somebody from GES, health, to form that child protection, when you go down, we also have community child protection teams. Then the informal ones, the family system is there, the extended family system is there so that is why I am saying maybe you are repeating.....

M: I am sorry if you feel that way. What type of cases would you use the formal systems and which one will you use the informal systems? Can you give us an example of a case where you use the formal system to solve and a case where you use the informal system?

R: All the cases you can use the formal system and you can use the informal system depending on the gravity of the case. For example if a husband have a problem. Sometimes the issues they raise are minor issues but it is husband and wife issue so you can refer them to go back to their home and resolve it/. But some of them too, they need some technical advice, I dont know if I have made myself clear, there was an issue that was brought to my office, and the things the woman was raising, I dont see them to be any serious issues. They are just some petty problems. The husband's father is doing that, is doing that... he goes to drink and comes to make noise, you see those things you have to handle them in the house.

M: So that is informal?

R: That is informal. But there are also some issues...

M: Like? Can you give me an example?

R: Like there was an issue like the man is not responsible and he works in a different place, so it is like he is running away from the woman and because he works somewhere he has got advantage. So when he goes there even weekends, we don't come back. So such a case, you will need to know what a woman is doing that is sacking him from the house and then you will also need t investigate whether there is something there that is also keeping him from going home. The local people may not be able to do that kind of critical investigation of the case so that you talk to the woman and talk to the man.

M: Alright, thank you very much sir, we are done with the interview is there any other thing you want to add?

R: No.

M: Alright, thank you very much.

M: And what else?

R: Do you want to get everything?

M: Yes, tell me everything.

R: Okay. I also develop and implement technical activities such as EPI

M: Please what is EPI?

R: Expanded Program on Immunization.

M: Okay

R: I have about eight technical works. Do you want me to mention all?

M: Just a few, maybe one or two

R: Okay. The disease control unit is one of them. The public health unit, the health

Information unit. We have the mental health unit also in the district. So I coordinate the activities of all the technical units.

M: Okay sire, so having mentioned your roles as the district health director, can you please tell me your department's co-role in terms of childhood developments?

R: For the departments in terms of early childhood development, I would not go into all the eight departments that I spoke about; maybe just one or two

M: Those in charge of early childhood…

R: Yeah. The first one is the CHIPS unit. Currently we are even into ECD for all the 30 CHIPS zones we have in the unit and we started this as a pilot in 2017; at the near end of 2017

M: Please can I ask what exactly you do?

R: For the ECD activities, we were actually taken through the activities by the BIBLE. Do you know the BIBLE?

M: No please.

R: It's an international organisation. So they took us to Accra for the maiden meeting and followed us to upper west for the technical training. We have to involve all the 30 CHIP zones that we have in the district and we have to also train volunteers. Actually the project is riding on the back of mother to mother support group that we established in the district. So the BIBLE is not giving Ghana Health Service direct funding to roll out their activities; so as to when we are having meetings with the mother to mother group, we implement the activities of early childhood development. So we have been taken through 15 steps. I would not be able to mention all those 15 steps

M: You can mention a few maybe one or two

R: Okay. So we have some steps that we take the mothers through to be able to tackle their children, show love to them and let them feel good around them. One of them is teaching the mothers how to make toys by themselves for their children. They can use clays, plastic, paper or any available they have on the grounds. They can use these to play with the child to display the love that should be between a mother and the child to their children. Early childhood development has to do with a window from 0-3yrs. So within this window, you're supposed to show these children some love. If this period elapses without that love, the child may grow in a different arena that you and I expect the child to be in. I always use myself as an example. When I was growing, my parents do not know the essence of early childhood development. My parents didn't show me that love I wanted between the ages of 0-3yrs; just because they didn't know, if they had known, they would have taken me through it. Those days if you hear the sound of your father's motor bike when you are outside, it was just always a scary moment. You have to rush and go back home. If your father enters the house and mentions your name and you are not present, he's going to mend out some punishments to you. Our mothers too at the time, do not have the time. My6 mother was always doing her business and forgetting to show that needed love for the child. But now that I have gotten some basic training on ECD, I think it is so paramount that, every parent, mother and father should show that love to their children. It has an effect on the minds of the children

M: Okay. Sire, in your presentation, you mentioned that you teach the mothers to mould clay items, toys etc., why do you do that?

R: If you use those clay materials to play with the child, it increases the bonding between you and the child. You see, you cannot just play with the child without showing any item. I was told, not from ECD though, that if the child begins to see beautiful things, it enhances the child's cognitive development. So let's say you show a child a red or yellow flower in the morning and in the afternoon, you show that child a different coloured flower, while you keep consistent in this act, you are trying to enhance the child's cognitive development. It is on that score that we decided to teach the mothers how to mould with clay to play with their children

M: Okay. Can you also talk about the relationship between your department and ECD?

R: We are into partnership; they don't give us direct funding but they support us to build our capacity. They also do come around anytime we having some ECD sessions with the mothers. They come to observe and they'll reveal new things to staff who are on the grounds. There was a time, one of the attendants to the BIBLE came around just to have a first-hand information on ECD acti8vitries in the district. So they offer us technical support, capacity building and for the finances, they only give us sitting allowances and feed us during our training sessions. Remember I said the activity is running on the back of mother to mother support groups.

M: Okay. So can you tell me about some of the cultures that operate in facilities in this district about child welfare?

R: What do you mean, if you say a facility?

M: Health centres or Hospitals in the district

R: I was thinking you were referring to cultures in the community but as a health facility, what cultures do you think we'll have if not trying to render quality services to our people?

M: What I was trying to say is that do you have special routines, traditions and activities that are peculiar to your district that you have initiated that other districts do not practice? You could have special initiatives as a district that you do, and those things are peculiar to only Wa west district.

R: So as a facility, our innovations…

M: Yes as a district; They could be things that you do surrounding child welfare issues. It could be routines that you've instituted that the health centres or the CHIP compounds

are practicing and those issues are surrounding child welfare and these things are peculiar to your district. It could be that you have instated those things as the director, for this district

R: I cannot say much in this regard. Other districts are equally doing same as we are doing. Like the mother to mother support groups that we have formed. Every CHIP group is supposed to form a number of mother to mother support groups which we use to spread health messages; anytime they hold their meetings, a health worker would go and speak to them on family planning, child welfare clinics, malaria prevention or any health topic. I know other districts are equally doing these. We are actually the one of the districts among three districts selected to pilot this ECD project. So while we were having the sessions with the mothers, other districts were not doing it but either early this year or late last year, the other eight districts have been brought on board. We were able to implement it so well that the BIBLE saw the need to scale it up to cover the other eight districts. So ECD activities are all over the region.

Also, Plan Ghana is also supporting us to do Community Child Protection Committee Mapping. Those committees have existed before but they got dormant because there was no funding and follow ups on them. But Plan Ghana supported us with some funding through social welfare so we went out and did some mapping on the existence of those Community Child protection Committees. We are done with it, and we may be following up and monitor the activities of this committee on child protection.

The other routine could also be the welfare clinics that we hold. The Target child for this one would be from 0-5yrs. Every single health facility is supposed to have this Child welfare clinic either outreach basis or static. Outreach implies that they'll leave their community to go and do it somewhere else whereas Static implies that they'll stay in their facility while providing the services. We basically provide services on immunisation and growth promotion; we monitor and promote their growth. We cannot communicate with a child under five years so we need their mothers. So the mothers come with their children before we render our services to them. Before the session, they give health talks; it could be on any health topic before they do weighing. And if the result from the weighing isn't good, they have educated or counsel the mother either on nutrition or something else. When giving an immunization, you'd have to tell the mother what vaccine you're giving to the child and its relevance. On malaria prevention, because the child is vulnerable to the parasite, you have to educate the mother on how to protect the child from mosquito bites. These are some of the things we do.

M: Why do you use mother to mother support groups to talk to the mothers on the ECD activities?

R: They do visit us but these groups are already existing groups in the community. Instated of organising a separate meeting to invite them, we only integrate our activity into their already organised ones. So immediately they are done with their activities for the day, the health worker steps in and educate them on a health topic. It is easier to meet them at the time their meeting because it saves time and energy.

M: What are some pf the major health problems of children from 0-19yrs in this district?

R: Malnutrition; from 0-5yrs, most of these children are faced with malnutrition especially underweight. Let me term it as severely acute malnutrition. This district is noted for high prevalence of malnutrition cases. So we have a lot of those cases and you can see I have posted on the board. Fortunately for us, we are running a program to take care of the malnutrition children. We call it Community based management of Acute malnutrition (CMAM). So when we identify them in the community, we admit them and put them on some food supplements and monitor them for a number of months until they are rehabilitated, then we'll take them off our register. That's why we say they are community based. Those that are so bad, we admit them at the hospitals and that person would become an In-patient and the others that are managed at the sub districts or community or health centre levels, are out-patients because they come and go to their homes.

The other thing I would like to speak about is teenage pregnancy. We have recorded quite a number of pregnancies in the district. If I give you two or three-year graph, you'll see that it has been on the ascendency. I would not be so specific with the figures. There were 391 cases recorded for 2012 which reduced slightly to 376 and since 2013 it has been steadily increasing to 537 in 2017. The 2018 figure has slightly reduced to 528. These occurred between 10yrs -19yrs; the early and late teens. In 2017, I realise the number of early teens thus 10-14yrs, were as many as 6 in them and that's worrisome! I think it is peculiar to the district and I realise that it has to do with cultural issues. I don't know if your questionings would permit me to speak of cultural issues

M: Oh yes, I was going to ask about what in your view accounts for these results, so you can go on

R: Okay, I would say cultural issues. Personally, I saw a pregnant child that was 17yrs in the health facility. Her mother in-law has brought her to the facility to do Anti-Natal Care (ANC) and that's one in a thousand because they shy away from this place. So her mother-in-law brought her thus the child's husband's mother brought her son's wife. As we were interrogating her, she was so proud of the lady as her son's wife even though the girl was only 17yrs of age; she hasn't seen anything wrong with it! The girl happened to be a school girl and has to become a school dropout because of the pregnancy.

Mostly funerals are attended usually at night. These kids also attend these funerals to mourn; I have not conducted any survey to ascertain the fact that the adults are responsible for the pregnancy of these children but, what I have seen and heard suggests that some of the adults take advantage of these little children on the funeral grounds and make them pregnant. So we had instances where adults were responsible for the pregnancies of these children; they grab them at funerals, lure them with money and sleep with them.

M: So you are trying to say that allowing the children to go to the funerals at night is also a cause of teenage pregnancy?

R: Yes, it's a contributing factor. If they were not there, where would the men find them?

M: Sire please, what about the malnutrition, what do you think are the factors that cause them in this district?

R: I failed to tell you something; the district about 2 or 3yrs ago was rated the poorest in the country. We don't have so much to eat or live on. The poverty prevalence rate was as high as 29%. The malnutrition rate was high as well. All these was recorded in the report; if you want the report, I can log online and get it for you. So we are poor, we don't have so much to feed ourselves and families. We are not engaged in activities that would bring us food as well. Wa west district is so vast; the land is available but we are not into farming. If every home could farm well, there could be food for every home but majority of them do not farm. You know we have barely 3 months' rainfall per annum. So the little proceeds they get from their farms, they exhaust before the next farming season. So we have to be dependent on the cheapest food from other sides which is Kokonte. Those who have dried their vegetables, live on it during these periods! So it is poverty that is the main cause. I wouldn't say it is ignorance because if I say it is ignorance, what it means is that we have food available and we don't know how to use it. So I wouldn't talk about that but poverty as the cause instead.

But of course, if you still want me to talk about ignorance as a factor, I would talk about it.

M: If you think it's a factor, you can talk about it.

R: Yes. When you're still breastfeeding a child, the nutritionist would tell you that you have to let the child suck both breasts. Even with that, you've to ensure that you empty one breast for the child before the other, you don't have to be giving one breast to the child to suck for few seconds and change it to the second one for the child. If you do that, you're only feeding the child with water! The nutritious part of the breast milk is stored beneath

the nipple; so what exactly or closest to the nipple is just water, the nutritious part is beneath that layer. So you need to bring that water before the nutrients flows; God in his majesty, made it so, so that, you can take in some water before the main food comes in. So if you don't empty the breast instead, you keep changing it for the child, the child would not grow well.

M: The nutritional value is lost!

R: Yes, that could be one of the factors but I don't want to go there!

And again too, you can have the food available but you don't know their mix so you can just be giving anything to your child and that can also affect the child's nutrition. But that's why we are there as health workers; we do talk to the mothers as to how to care for their children, what food to give to the child. Especially during our raining seasons where we have our vegetables, we make the mother feed the child on that.

M: Sire, can you also talk about forms of initiatives and programme the district has for addressing growth and development related problems of children?

R: Addressing growth and Development related problems?

M: Like the malnourished issues, Disability issues, if you have overweight; do you have initiatives or programmes in place to address these issues in this district?

R: For the malnutrition issue, I think I mentioned it earlier where I spoke about CMAM programme. UNICEF was supporting us with some food supplement called Plumpy'nut but for a very long time now, UNICEF has not provided us.

M: Why?

R: I don't know!

M: Have you found out?

R: Now we don't have plumpy'nut anywhere here in the upper west region but we cannot just allow the project to die off so we still identify these children using the MOAC tip. Then if we don't have the plumpy'nut, we still counsel the mothers as to how to use locally available food

M: Like?

R: Those mothers who can afford the egg or milk, can buy it and start giving them to their children.

M: **Aside that, what else can they use?**

R: We have the Bambara beans, they can boil it very well and very soft. Add some oil and give to the child. They can use groundnuts as well. You know that is what is used in making plumpy'nut. Plumpy'nuts are made of groundnut paste. So plumpy'nuts have not been in the system for some time now.

M: **And you said you don't know why?**

R: I don't know why they have not given us!

M: **And you haven't also taken the pain to find out why it is not being provided?**

R: Oh no! We also get the supply form the regional store, so, if you tell the regional nutrition officer and he says he's not having, what do you do? But we don't leave them, it is a health disorder so we cannot just leave them like that. So once we identify you that you are severely malnourished, we have to register you on the program and we do the best we can do to you. But the plumpy'nut was really helpful! Within some few weeks, the child would be fully rehabilitated; that's why I showed you the before and after pictures of some of the cases. But for a long time now, we don't have them in the system

We still have another project we call the Infant and Young Child Feeding (IYCF). It is also a programme that we run with the Child welfare members. So we have a register that we register these children in and we provide counselling to the mothers as to how to take very good care of their children to make them grow well. So I think this is a preventive and not a curative method/ approach. Because, we provide counselling to the mothers alright, and that's okay but, we talk to the mother to take very good care of the child so that they'll not encounter various problems.

M: **What about issues of children that are overweight, underweight or disabled children, do you have any initiative to cater for them?**

R: No, we don't have any special initiative for those children.

M: **Okay.**

R: If we notice any kid with an issue of overweight, we have to refer him/ her to another place. But, rarely would you see such cases in this locality. I told you that we are poor so rarely would you see such children. For the disabled children; the children with weak limbs, blind or deaf, we usually refer them to the bigger hospitals. Those with Cleft cases too, we do refer them. Sometimes, they organise surgery operations in Tamale. So when

we hear about them, we tell such mothers to take their children there but we don't have any special programme to take care of them

M: **What about home visitations, do you sometimes visit parents at home to talk to them or monitor growth of children?**

R: Perfectly well; we do that every day! We have standards to meet. So in a CHIPS compound, every worker in that particular zone, is required to do three (3) visits within a week. And with each visit, they're supposed to visit 8 homes. So we have 24 visits to make with a week, we have 96 visits to make within a month and we have thousand one hundred and over to do in a year. We have a well-designed home visiting register in which we take the details of the people that you'll meet, what education you're giving to them, what were some of the issues you identified within that house hold and how you solved them. Every health CHIPS compound has their register and with a port on it to the region. We do that a lot/

M: **What about seminars for parents?**

R: We don't hold seminars but we hold durbars. In a community, in a health centre, I cannot say I am holding a seminar! If you say, community durbars, fine!

M: **Okay, so you do hold community durbars?**

R: Yes, so we hold the durbars to talk to them on health issues. We have various forms of durbars. We can have sensitization durbars; we can hold a durbar in the community to sensitize them about some health issue. We can have a fee-back durbars; we can a hold a durbar to just give a feed-back on our activities or some particular health situation within the community to them. We can hold a durbar to have a plan for health issues within the community. If you can see, we have CHAB thus Community Health Action Plan. We can do it together with a session of the community, then, we can now have a bigger durbar and inform them about what is happening in terms of health within their community. We can hold durbars to launch an activity; if we are having a particular problem or a condition that is a problem to us. To be specific, Lymphatic filariasis; in simple terms, Elephantiasis thus those with the bigger legs. We do record a number of such cases within the district. So from time to time, we hold durbars to launch such activities if we are to do any mass drug administration. We call people together, educate them on the disease and launch it and start the activity. We hold durbars to educate people on Tuberculosis and HIV cases as well. We always hold community durbars for a reason!

M: Okay. Sire please, are there any other interventions that you do apart from the ones you mentioned earlier?

R: With regards to the child?

M: Yes

R: We also do radio programs but it is not solely to the child. We believe that if the issue is regarding the child and you tackle the parent, you are equally tackling that particular issue affecting the child. Of course, the child is innocent and helpless, it is the parents who are his/her caretakers. So, we have to tackle them. So on our radio programme, we educate the masses on some health issues. We have a full plan on that, so one is one child welfare clinics where we sensitize, inform people on the importance of child welfare clinic. The second one is family planning activities. We try to tell our mothers or the women, the importance of planning their homes. Then malaria prevention which are common.

Apart from the radio program, we have one group that we deem very important and we call it father to father support group. You remember I said we have mother to mother support group.

M: Yes

R: So we deliberated on this and did a lot of stomping said that, why not form father to father support group? Because, the health of the child is not only dependent on the mother but the father as well. We also noticed that the fathers are a bit reluctant when it comes to the health of the child. Indeed, they're not supportive. We are trying to lure them in, sensitize them and make them understand that, these and that are necessary of the child, so we try to bring them on board. We started it with only family planning. The man is the head of the family so he decides whatever goes on in the home. So if a man discovers that the woman has gone out to take a family planning device without his knowledge, he wouldn't take it lightly with her. So we decided to form this father to father group, to educate them on family planning.

M: Okay!

R: Those groups would be the ambassadors of the activities to their friends. If your colleague who used not to support that idea has come to tell you about the goodies of it, that would help. Later, we decided to add other health issues and not only on family planning. Currently we have formed about 28 father to father groups in the district. We are into some casual friendship with University of Massachusetts, USA, so they do come here every year. We started in 2017. So one of them actually came and supported us to form

about 12 father to father support groups last year increasing the number to the 23 that I mentioned. So that resistance would no longer be there as to whether a wife can go for a device for family planning and it would or not become an issue. We are trying break that bond so that all of them can be together in understanding about these things.

One other activity that I have to mention is KSS

M: Please what is KSS?

R: Knowledge Sharing Sessions

M: Okay.

R: We have been doing this for a while now; for about more than 2yrs now and it has been accelerated by an NGO in Tamale called Savannah Signatures. They provide us with the bresource3s to hold such services. It’s a session that we hold with community members to discuss health issues. We do go with a video and after they’ve finished watching the video, we’ll pick a mic and ask questions and interact with them. We can also go and tell a story tailored towards health and questions are asked afterwards. The mothers are the ones who turn up well; some do come to share their experiences with other mothers.

M: How has the feedback been since you started the KSS?

R: It has been helpful; it is yielding results! We get good attendance during our child welfare clinics. We talk on health seeking behaviours. For instance, when you are sick, where should you visit first? The health facility, herbalist, traditionalist, friend or chemical shop? You have to visit the health facility first. Our target is always one opd per capita. Last year we got 0.93 which is closer to one. This per capita means that in a year, from January to December, the number of people we have living in the district must visit the facility once thus individually. So the computation is that you divide the number of people who visit your facility by the number of people in the community and if it’s approaching one, it means the patronisation is high but if you get 0.5, it implies that half of your population has visited your facility and that’s not good enough.

M: Why is it not good enough?

R: You expect everyone to come not necessarily that you should be sick before you come. We have also started something that we call wellness clinic; not a clinic that only takes care of the sick. If you are well, you can just walk to us, check your BP, temperature, weight, height, we’ll calculate your BMI for you and we would counsel you and that’s a visit to our facility.

M: **Can you tell me about the major funding situations related to elements of child and adolescent health and development interventions in the district?**

R: Talking about funding, you have reminded me of one key activity of ours that I didn't make mention of earlier and that's organising trainings for adolescent sexual reproductive health. When we noticed that the teenage pregnancy was on the ascendency, we organised training session for the adolescent on sexual reproductive health for about 9 days in three badges. So that's one thing that we also do.

M: **You did that for nine days and after that?**

R: After that, we would follow up on them and see. We called them from selected community and schools. We started with the schools. Plan Ghana supported us for that activity. We used existing school clubs; and we were more interested in the females

M: **Why?**

R: Because a man would not get pregnant and drop out of school, it is usually the ladies that are the victims. So they're our targeted people. So we selected 10people from 10 communities which summed up to 100 people. So the next activity is to follow up on these clubs to see if they've also formed clubs to start teaching their colleagues what they've also learned from us. I think that also helped. You'll marvel if you were there to see their response to the questions asked. So it's going to make an impact when they go out to share to their friends and families.

To our main funding, it is always difficult when it comes to money in Ghana. Plan Ghana is one who gives us funding

M: **I mean major funding**

R: For?

M: **Elements of child and adolescent health and development interventions in the district**

R: Oh I can start with the government. The government is supporting to give us finding and logistics for immunization service. Global fund and UNICEF also supports. GAICA is another source; they even build facilities for us; they build CHIPS compounds for us. Currently we are into a project called Life cause approach, it is GAICA who are seriously doing it and it's another phase of their project. So they do capacity building. It's not easy to train a Community Health Officer (CHO). We identify people we want to be trained as CHOs and they'll train them for free for us. DFID also support for maternal and child health activities. The district assembly is also one that supports with logistics and fuel for

external activities. Under minor sources of funding, Savannah signatures also provide small amounts of money, BIBLE is one on the ECD. UNFPA is also one but they have not given us any funding since 2018 but they used to support us on family planning activities and pregnancy classes. Of course we determine the priority areas and they'll give us funding for that but since 2018, we've not received funding from them. IPAS also used to support us on family planning. They give us planning to organise durbars in the community and we crown it with a float on the principal streets of the community. But since 2017 till now, we have not receive any funding from them

M: **Which of these, would you consider as major sources of funding?**

R: For those who have folded up, where would we categorize them?

M: **Let's work with the ones you're currently working with**

R: The government is a major Supporter. UNICEF and GAICA. Savanna signatures and Plan Ghana could be minor supporters.

M: **Do these funding sometimes come with conditions?**

R: Of course! There's no funding that comes without a condition.

M: **Can you share some of them?**

R: If you are getting money from the government to roll out an activity, it comes with a spread sheet. The spread sheet would tell you how the money should be spent so we are guided by that particular spread sheet in the execution of our activity. Those coming from NGOs, they would also come with Terms of Reference (TOR). In this, they'll indicate the total sum of the money they're giving to you and how they'll give it to you; the percentage they'll give you for the start and end of the programme. It will tell you what to use the first percentage for and when you are to retire, you have to go by that till you retire. For instance, if you are to use GH¢ 100.00 for stationary, you cannot use more than that unless you have a strong reason to convince them on the need to use more than that amount on stationary. So they come with conditions. For the others, for instance, if IPAS gives you money for a community durbar and do float at the end, they must see that in your retirement and your report. No donor would give you money without asking for a report on it. You must provide both technical and financial reports. The financial report is the retirement/liquidation and the technical report is the activities you have carried out.

M: **What about areas of investments?**

R: I am not clear on that

M: Do you have areas of investments as a district?

R: As a district, no but what I can say is that we render service to the public under National health insurance. So any child will reimburse us later. So I can say that one is our internally generated fund (IGF). When we attend to a sick person, the person is supposed to pay us but not directly; through the National Health Insurance company card thus a valid ID card. So we generate some money at the end of the month and National health insurance would reimburse us. So when they reimburse us, we sometimes go into it to finance minor project, or top up some projects. When we are going to some NGOs, they'll ask us what we can also provide. So with the IGF, we can say that we would that we can do this or that in support of the programme as well. But we don't have the mandate as a public institution to go and invest, get some proceeds to carry out an activity; we cannot do that at all!

M: So what about Sustainability?

R: In terms of sustainability, some NGOs would have a plan as to how they'll pull out and the activity would still be on going. So if you are an NGO or a donor supporting health with funding to carry out an activity, such plans are always there so that when they fold out, the programme can still go on. Remember I said that the ECD that we are doing is riding on the back of mother to mother support groups. So if the BIBLE is not around to help support us in the execution of the project, the mother to mother groups and health workers are still available. Now they've build our capacity with the needed tools to be able to carry out the activity. The 15 steps as I mentioned are still there, the counselling cards are still there. People's capacity has been built so they know what and what to say to the mothers. So the BIBLE is gone but the activities are still there. For instance, GAICA is training CHOs. They are building the capacity of CHOs and the district to be able to monitor the activities of the various CHIPS compounds. So that is sustainability, when GAICA is not there, the activity would still go on. Plan Ghana's support for the adolescent sexual reproductive health. We are using existing clubs to run this, so if plan Ghana isn't here to support us, the schools and the clubs are there! So those are the plans to ensure that there's sustainability of projects when the donor isn't around to support the project.

M: Oh okay. What about how your outfit record information on special children?

R: We have as a public institution or Ghana health service; we have a common platform called District Health Information Management System (DHIMS). That is the system that keeps all the data that we capture from the field; if you go out to roll a CWC session, the number of children that you met, those who have receive a particular intervention, what service you have rendered are captured in that system. We have reporting formats. So

we go out to the field with a register and summarise all that are in the register into a monthly reporting format and report to the district. We have officers who would enter these figures into the DHIMS. So these includes the information or data about special children as well

M: **Okay, but do you have a designated department for Special children?**

R: We have department that handle for instance the teenage pregnancy issue that I talked about is handled by Reproductive and child health unit. Diseases control unit also go into child welfare clinics which involves children. Nutrition unit also takes care of the severely acute malnourished children

M: **I was referring to children with disabilities. Do you have a special department that handles solely issues concerning such children?**

R: No. So when we get such cases, we would refer. When we get child with Ear, Nose and throat defects, we don't have a specialist here so we have to refer him/her to Wa where they have ENT specialists.

M: **So any special cases needs are referred because you don't have the specialists here?**

R: Yes, we don't have the specialists; remember this is a rural district.

M: **What about some of the interventions you provide for parents who have children with special needs?**

R: Apart from the malnutrition patients, we don't give any other thing.

M: **But do you do screening to find out cases like that?**

R: Yes

M: **How often?**

R: Monthly

M: **Once a month?**

R: Yes, or let's say quarterly; it's not so regular. We have an eye unit. They go out from time to time to the schools to screen people with eye conditions. We also have the dental unit. They also go out from school to school to look at the oral hygiene of children We have the public health unit at the hospital and various health centres. They go to the community to screen people on various public health conditions. We organise integrated key search and we form groups to go out from one community to the other. We have 226

communities so we cannot visit all the communities at a time. So we dwell much on the hotspots. And the hotspot is where we have ever recorded a particular health condition. so we take the groups to visit such communities to search for such cases.

M: So when you screen and get them, what do you do?

R: We can refer them to the facilities for treatment and those that are beyond us, we refer to the region for treatment.

M: Alright. Having spoken about what you do as a district regarding health issues and interventions, can you tell me about the capacity of health personnel in this district and how efficient they are to deliver child health services?

R: My staff have the capacity to do that. I don't have one particular kind of staffs. We have different kinds of staffs who render clinical services, public health services among others. Each them have some basic training from school. If I say basic training, I am referring to their profession. If you come as community health nurse, you have basic training in community health nursing. If you come as enrolled nurse, you have basic training in enrolled nursing. If you come as Physician Assistant, you have basic training in Physician Assistantship. So in all those professional areas, they have some basic training in them; they have been given the capacity to handle professional issues in regards to their fields. We still call for refresher training for those who have been on the field for a long time to update their knowledge. We also have something that we call the CPD. In this CPD, staff need a point to be able to renew their pin and before you renew it, you have to go for the continuous professional development trainings to be able to get some required number of points to be able to renew your pin.

M: How often is the renewal done?

R: Every year! Health is so dynamic; it keeps on changing. So if you are having your previous knowledge from school and you're still holding on to it, you may become archaic. So you need to be abreast with current issues. So they have the capacity to handle children within their scope of work.

M: What about number?

R: The district is so vast. Like I said earlier, we have 226 communities and 40 health facilities within the district. To me, I think that it is still not enough. We need to increase access. We have up to 2030 to attain universal health coverage. Our director general is so emphatic that he wants to attain universal health coverage by 2020, next year! The vehicle would be the CHIPS concept so I as a director thinks that we need to increase

access by bringing in more health facilities to be able to reach out to many. So if we are increasing access, we need to increase the numbers of the staff as well. Currently, we have about 306 staff, we have just been given 69 staffs so we have about 375 staffs on our pay roll; they are those we are using for the 40 health facilities.

M: And they are not enough?

R: Of course not!

M: So what number do you think…

R: Before I can answer this one I have to do a lot of human resource assessments to identify gaps before I can but I cannot just give you a figure. It's technical. So if I am thinking of increasing access thus building more health facilities, I should be thinking of increasing the numbers as well else, if you build the health facilities, you'd use the same number of staffs to run them and you may not be making the map!

M: So it means because of the numbers you are not able to cover all the geographic locations? Are you able to cover all the areas?

R: Yes! We still cover all the 226 communities. But we have been using strategies; organising outreach activities. So if all the outreach points are turned into health facilities, there would not be the need for outreaches. But we still cover every corner of the district through outreaches.

M: Okay. What about how you identify personnel who have capacity deficiencies in delivering child welfare services?

R: Oh it's very simple! It is through effective monitoring and supervision.; paying a supportive visit to a facility and watch them render the service to the people. The protocol is there, the SOPs are there; so if t person isn't doing it to standard, you'll know that the person is deficient or not up to task. And, using the technical people to do monitoring and supportive visit on their technical areas. For instance, you picked a disease control officer to support a health facility organise a child welfare clinic. He knows how BCG vaccines should be given, he knows hoe Penta vaccines should be given, he knows how any of the antigens should be given so if it's not done well, he will know. Maybe, he would not prompt you there and then but he would raise it up at our staff meeting that our staffs need training on such areas so that we would refresh them on it. Sometimes, he may be doing it out of ignorance so once you spot it, we take the necessary actions. And again too, in the area of nutrition, when we are trying to identify persons with acute malnutrition using the MOAC team, if the person isn't using the MOAC team very well,

we will identify it, we are technical persons and for this you can correct right on the spot. We call on the job training or Onsight coaching. When we receive new anthropometrical equipment, if we think they cannot use them well, we bring in the equipment unit people to come and take them through how to use such equipment.

M: Okay, Thank you. So I'll like to know that when you identify people like that, do you have interventions for them?

R: Yes. The intervention is calling them together to give them refresher trainings

M: Is that the only intervention you give?

R: Sometimes, we can attach them to people who have much knowledge on the job so that they can mentor them

M: In what way do you think the health directorate can support parents in care and nurturing of children?

R: I would say holding regular community meetings with the care takers. Intensify our home visits to be able to take them through health issues. We can also do radio programmes to be able to reach out to the masses on the health issues that confront the district. The ECD programme, using the mother to mother support group is also another thing that we can do to communicate to them on how to care for their children. That's all I can remember

M: Sire, please is there anything you'd want to add that we have not discussed?

R: I would say that I am a technical person and I believe in technical things. So I believe in what you are doing; it's so scientific. Conducting a research to be able to identify issues and come out with interventions to solve that particular issue. It is very keen. So I know that what you're doing would benefit us as providers. Hopefully when you are done, a report would be disseminated and we would get a hold of it; though not directly to me, it would be to the government and it would be published and everyone would get to know about it. So that is the way to go. But I would want to say that if that is done, we should not end it there. We should come out with the interventions to help solve the issue that are on the ground. And for me, I believe in organisational development. UNICEF or our development partners should begin to look at how to develop organisations. I believe in it because, it allows the staff to bring out their best. The leaders are so strategic in getting the best from their staff. Staff are motivated to bring out their best. Teams are formed to bring out their best. When we were in primary school, they used to the us that divided we fall, united we stand! So when we are together, we have some cohesion and we can do a lot but if you're an individual, you may not be able to do much. So if development

partners can begin looking on how to develop on these things, it would be really helpful. We would really be getting the best from the staffs; we would be building teams. And it doesn't matter whether the person has been trained on his professional area or not, but he needs that knowledge of organisational development. So our partners should begin looking at that. I believe in it! If you know what to do, before I come to the office, you've already done it and you know that I would like it. So it should not be a system where you'll be sitting down and waiting for directives before you act; it's like you're working for that person. It should not be like that. develop the system in such a way that everybody knows how to go about things just for the attainment of that common goal that you're all looking at. So development partners should look into the area of cognitive development

M: Okay. Thank you very much.

R: Okay

District Name	

M: Helloo good morning or is it good afternoon? Good afternoon, good afternoon.

R: Good afternoon.

R: You welcome.

M: We are about starting our discussion. Please an you describe the general work of your institution?

R: Thank you, the work of my I sous the…1. To monitor and supervise at the KG level, kindergarten. That is from 0-8. Then 1 to class 3 and also to give reports to my director at the end of every term.

M: OK.

R: And also to report issues, my observations, challenges in school to my director.

M: So that is the general task.

R: Yes.

M: OK, so what is your role?

R: My role is to visit the schools, sit in the classrooms, see to what they teach and also advise teachers on how to take…handle the kindergarten pupils and how to I mean, go about their…the use of TLM to help the children. Now the KG level, you are not suppose to…

M: Madam please can you speak a bit louder?

R: OK.

M: Because of the recorder.

R: The KG level you know, at the age, they need some special care…

M: Care.

R: And the…our system we don't have teachers that ha e gone for that training. So it is my duty to organized In Service fir the teachers on how to handle the KG pupils.

M: OK, thank you, please in relation to children and adolescent, what role does your department play?

R: Children and adolescent?

M: Children and adolescent, yes.

R: As I earlier mentioned, my this thing is from 0-8.

M: OK.

R: I work with them.

M: So for the adolescent too you don't do any direct job on them.

R: Yes.

M: OK, so please tell us about some of the services you provide to parents, care givers and children.

R: With parents I think, if they will be some kind of education, to educate them to understand how they going to handle the, their kids. Some parents are there, I mean like in our our this one...how do you call it? Like our homes some children they have problems on the part of their learning and the we don't understand. May be somebody, you know we have somebody who is a fast learners.

M: Yea.

R: Others are very slow and in our local when we see such a thing oh this child is not intelligent.

M: This child is not intelligent.

R: This child is stupid, cannot do anything, you are in KG and this is in KG 2 and that person will be able… I mean that child is not able to I mean let's say, say numerals and the how do you call it? Alphabets and a a child in that same class cannot do that and some too can identify the colors and other things but some cannot do.

M: Cannot do.

R: And when it comes that way they say this child is not intelligent and it is not so. They don't have any child who is…

M: Not intelligent.

R: No, we don't have that.

M: So what training do you give to the parents in regards to these issues?

R: Since I reported as a coordinator, early child coordinator I haven't had any…

M: You haven't had any.

R: May be God willing next academic year. We had a workshop on such issues.

M: OK.

R: And the parents are many, at certain times we have to invite them to our classrooms to also teach them. At music and dance.

M: Teach the parents?

R: Teach the children.

M: You invite some the parents to the classroom?

Y: Yea.

M: OK.

R: That's now they are now bringing.

M: Oohkay.

R: So we will organize workshop next academic year.

M: OK, so what are so e of the workshop you want to organize for the parents? Can you mention of the topics that you will discussing?

R: Hmm, topics like, just I mentioned, you know some parents they are good at this music something and the dance.

M: Cultural dance.

R: Yea, we invite them to come to the classroom then teach the children.

M: OK, do you also have…

R: You know at that age, there are, I mean they want activities.

M: Yea.

R: Hmm, they learn through activities.

M: Do you also have programs to syntheses and parents how to handle violent children, like children, the violent ones.

R: At…

M: Do you have…

R: So far there is nothing like that here.

M: OK, based on your experience working in this community, please tell us about your observations on how parents and child… on how parents to adult- child relationship is in this community.

R: This your question.

M:But you understand the question?

R: How they relate with their children?

M: Yes, parent child relationship in this community.

R: This your question, Kwabri East?

M: Yes, Kwabri East, specifically Mampongteng. Mampongteng is the community we are talking about.

R: I'm not from Mampongteng. I don't leave here.

M: You don't stay in the community at all?

R: No.

M: So you've not heard anything as to how the parents treat their kids.

R: No.

M: OK, so we can…

R: Some parents.

M: Some parents.

O: Some parents not all.

R: With that children because of some some a how do you call it? A peer group influence.

M: Yea.

R: Like, some children are there they will not go to school, they are in town doing things that are eerrmm, like a a taking drugs and then a how do you call it? Drugs and a… Some parents have complex with their children who have join their peer groups who are drug addict.

M: OK.

R: And a a let me use this word, excuse me some prostitution.

M: Ok

R: You know our girls in the the jhs at their level they need one or two things. I mean to keep themselves neat.

M: Yea.

R: The parents are not able to provide, they go in for boyfriends that can help them. For example some are there, the parents could not even provide them with pads.

M: Yea.

R: When it happens that way, the girl has to go to the boyfriend for pad to keep herself clean.

M: And these things…

R: And the boys too you know these days they say “as for me I’m a guy. They say it when they take that thing, weed, the weed.

M: Yea.

R: The cocaine, tramo see they behave in a way and you know at that age, you know they they admire some of these things. And then you see that they are doing that in groups they also become addicted to those things or addicted to…through that they have complex with the parents at home.

M: OK.

R: And even in our schools, in our schools, we have some challenges, you know when they take in those things, they look at the teacher to be like a colleague or their…

M: Small boy.

R: Yes.

M: OK, thank you madam.

R: You welcome.

M: Our next question is what intervention do you have in streamlining or aligning parents to guardian child relationships that are inconsistent with child welfare environment?

R: Almost I reported, the the Girl child coordinator, there was a case that was reported to her and she made a follow up.

M: Ok

R: To the the the home to meet the parents, that was about teenage pregnancy. They gave them, they counsel them. When it happened that way, when they are pregnant or that kind of this thing, they don’t go back to school. So the coordinator have to go and then counsel them to continue with their school till..

M: Do you work hand in hand with the Girl child coordinator?

R: Yea.

M: OK.

R: We even attended a workshop where…that was last March, concerning girl child.

M: So on the same question, what intervention do you have in, do you have in streamlining or aligning parents guardian child relationship? That's parent who have children with disability that has inconsistently child welfare environment just like the one you answered but this one those with disabilities.

R: On the part of the disability they refer them to the par apathetic officer. They handle such cases.

M: In the first place, what are some of the bad practices that are done against children with disabilities?

R: It's stigma.

M: In the community, stigmatization, OK.

R: And the when we are building our school too what do we call it, we don't build access, like they make big big steps where they cannot access into the classrooms. And they don't also have the blind, how so they call it? The walking stick…

M: Yea, the white stick.

R: And the cripple, they use…

M: Either the wheel chair…

R: Wheel chair.

M: Or some walking crutches

R: Crutches, they don't have such to help them and move.

M: So what is your office doing about it?

R: Hmm, as for the office, our office they are a lot if challenges.

M: OK.

R: A lot of challenges, so they refer to the district assembly to to help.

M Please how do you access the type of coordination that exist among child and adolescent related institutions? Like the department of social welfare, ministry of gender and social

welfare protection, division of family health, the others, the Dodsu. What is the coordination that exist? Is there anything like that?

R: Yea.

M: Or they work in separate ways?

R: Hmm, when was that, by then I was still a, I was in the classroom. And there was an issue, accident that occurred at Ahwia, a student girl that was selling hmm this, poor water and a car knocked her. Oh, the whole leg was condemned and the then early girl child coordinator took the matter up and the case was referred to the social welfare. And then they took the matter up, they took care of everything and it looks as if they gave him the the leg the leg was amputated then they fixed one for him.

M: OK.

R: The woman did very well, they even donated I have forgotten the amount. It looks like we are working hand in with…

M: So do you share information? Do you share information? These departments that I have mentioned.

R: Yes.

M: You share information.

R: Yes.

M: Do you normally meet to discuss issues?

R: Since I…work here.

M: You have not

R: Yes, I came on January.

M: OK.

R: Thank you, the question again.

M: The, I said how do you access the type of coordination that exist among child and adolescent related institutions. And you started by saying there was an issue with a girl who was knocked down by car.

R: Yea, not that I earlier mentioned that…

M: I was asking that you have been meeting…

R: Not, since I reported, there has been nothing like that.

M: There has been nothing like that.

R: But a a as a teacher, I used to hear and the girl child coordinator we were friends.

M: OK.

R: We were friends, I used get information from her. So now, she also when there is such, she reports to the social welfare then they took the matter up.

M: OK, madam please could you tell me something about the core issues in monitoring provicing children organization in terms of compliance to national or international regulations and rules.

R: International…

M: Issues preventing preventing you from perusing or ensuring that compliance is adhere to, that's what I'm talking about. Or I should read the question again? Could you please tell me something about the core issues on monitoring and supervising children on related Organizations in terms if compliance to national or international regulations and rules.

R: Hmm, issues like, you know when you are to go out for supervision you need some funds to do that and there are some places we wish to go but we don't have the means. We don't have the means to go there it's difficult.

M: I can hear you.

R: Monitoring and supervision you need a a means of transport or you need funds to take trotro or taxi to the places that you are to do your monitoring. And the this thing, there is nothing like that, we don't gave so it makes the work difficult even though we are always willing to do but we don't have the means.

M: So apart from the…

R: So there are certain places we cannot go.

M: Why?

R: We don't have the means.

M: The place is not accessible.

R: It's not easy and the and the you have to attach, you yourself what you will even use to do one or two things. We sacrifice and you see, the day that you don't gave money you won't go and then you have to do it on regular basis. No help from anybody it makes it, it makes the work difficult.

M: Difficult, please can you describe what makes children unsafe in this community.

R Children unsafe, hmm, they have financial problems in this community. If you look a long the road, there are these children selling pure water, child labor. Some carrying heavy loads on the pan at their age it's a problem. And at times you go to the school, what they will even eat, no uniform, no sandals and the parents cannot provide, some of them cannot provide. It makes…they don't have exercise books, pens, pencil…

M: To write in.

R: To write in, the parents you tell them, the teachers will try their best but…inviting them to come there and explain things to them. When I go I will do it, I will give money to the child…

M: How about social issues that are happening in this community that are not safe for children?

R: Hmm, the question again.

M: Some of the things that make children unsafe in this community.

R: Some of the things are, hmm, drug addicts and then some of the parents too are drunkards and they send the children to buy for them. When you send them, go and buy tot for me then the child will go and buy it and the day the child will say oh let me try and see and by doing so the child too become addicted to… And it will come to a state the child cannot do without taking the alcohol or taking the drug.

M: Any other issue?

R : And a lot of a lot of a a how do you call it? This game something, the gambling.

M: Hmm.

R: They have to pay in money before they do it and they don't get the money, they have to do what? To steal and you go round the community you will see those a I don't know the name of that teem. Some kind of game they put in money to also get something out of it. I don't know the name of that thing and that thing is also not…

M: Doesn't help, any other issue?

R: Teenage pregnancy too in the community for some of the girls, the parents cannot provide them with… so they have to also go out to look for boys to boyfriends to have affair with them before they can provide their…and if they are doing so and provide take any preventive measures at the end they will become pregnant and it will become a problem for the family and the community. Just I said earlier on, even petite petite things like pad and panties, parents cannot provide and they look as if it's a challenge. This my friend the the daughter she is doing this for the mother and you you are here you cannot do anything. But the girl is not working, she u s still schooling, how can she get that money or how is she going to get that money to I mean… Maybe the mother wants a funeral cloth and cannot get and the friend's daughter has been able to go out take a boyfriend somewhere get the money to do that for the money. So that girl too will be compelled or fir e to go out to also have a boyfriend to get something to help herself and then the mother and the siblings.

M: Hmm, OK, thank you madam. Please do children of disability feel safe in this community?

R: Aah, they are not all that safe.

M: Please why do you say so?

R: Some are locked up in their rooms because parents feel that the…oh, if my child have disability then take him or her out, they will say aah this person's child has given birth to such a thing. They don't even I mean, I mean take them as human beings.

M: So they are locked up in rooms.

R: Yes, some of them, they cannot come out. The mother feel that she will be stigmatized because of that she locks her or him in the room go out and do whatever. And the poor boy or girl will he there suffering so there are a lot of challenges.

M: Hmm, you have mentioned only one.

R: Their school, this time we have inclusive education.

M: Yea.

R: They don't it.

M: Is it the parents who don't do it or the school who don't accept them?

R: No, is the parents.

M: The parents don't take them…

R: To school and some if them can perform very well. Because they are locked up in their rooms, how are they going to learn? They cannot get access to education.

M: Thank you.

R: Welcome.

M: Please can you tell me your perspectives on rehabilitation services for children in this district. If they are any rehabilitation centers.

R: I learnt there is one at a a Djamase.

M: Djamase close to this place?

R: Yea, I have never been there, but I have never been there.

M: So what can you say about rehabilitation services?

R: It's helpful, I wish a a your your office will help us. You know it makes the disabled, some of them independent. It helps them to do a lot of this hand work and when they finish, they can also do something to help themselves and their families. If the government and the NGOs could help.

M: Is that all you can say about rehabilitation centers? Do they have care homes where children who are malnourished and stuff like that go to spend some time for special needs. You said there is one at....

R: Djamase, yea, as I said, I have never been there.

M: OK.

R: I heard that there is something like that over there.

M: Please what is your view on formal and informal child protection services available in this community?

R: Formal and informal…

M: And informal child protection services available in this community? So let's talk about the formal ones first.

R: Like a…

M: So Let's talk about the formal protection services available first.

R: OK, the formal services we have social welfare, we have Dorsu, then we have a a education service, we have the early childhood coordinator, the girl child coordinator and then we have the guardian counsel and then a, how do you call it, par apathetic officer in charge so when there are issues concerning disabled disabled children, they refer…

M: This one is not only disabled, all children.

R: All children?

M: Yes.

R: OK, they refer to any of these offices.

M: If there is an issue within a child and the parent or a child and an adult.

R: A child and an adult.

M: Even a child to child issues.

R: The child reports to the teacher if there is an issue.

M: Yea.

R: They refer to the officer and the schedule officer. The schedule officer too will a see the director and then they refer the case either to…if the case is concerning social welfare they refer to social welfare….

M: OK and then what about the informal ones?

R: The informal ones, we have the assemblymen and women and then the unit committee members. Then we have the chiefs too, they are in charge when cases of that happens, they refer to the chief and the other people.

M: OK, please what type of case or cases would you use formal systems to resolve or solve?

R: For instance of if a child goes to school and there is a problem, like the child is wounded or the child is beaten by the teacher. If the teacher beats the child and the child is wounded, the case is referred to the circuit supervisor. Then let's take it that if the child is a kg child, then the circuit supervisor will also refer the case to the early child coordinator then the coordinator will go to the….made a complain or report to the director. Then the director will sit with them and see with the other officers and see how they are going to handle it. So the…

M: How about the informal structure? What issues will you use informal structure to resolve?

R: Informal structure, when case like that happens, we go to the assemblyman or the assemblywoman.

M: What kind of case will you report to…is the…the question is in two ways. Cases that you will report to the formal structures and the ones that you will report to informal structures.

R: The informal structure like, if if there is a child if he attends school may be at a point a she becomes pregnant, you know when a child becomes pregnant she feels shy to go to school. Or the parents may even oh, you are pregnant so don't go, don't go to school. Such cases, we repot to the a a assemblyman or woman then they sit down with the parents and advice that when you are pregnant, it doesn't mean you shouldn't go to school on or stop schooling. You can go to school until you give birth and after birth you can if there is somebody, your mother or your sister that can take care of the child then you continue with the school.

M: Please can you tell…

R: And the the those who are smoking, the weed smoking. Those kind of things we report them to the assemblyman, the unit committee and the chiefs to solve that problem.

M: OK, please can you tell me about the case you used the informal system to resolve since you resumed office.

R: There is no case like that since I resumed.

M: There is no case like that.

R: Since I resumed, only in January, thus year 2019 so thus is my fifth month in office and I haven't have any case like that.

M: Hmm, how about the formal structures? We also want to know more about anther case that you, your office adapted the informal scheme to resolve. That one too you gave not had any?

R: Like parents reporting or?

M: Issues that you your office has used the formal, either the formal or informal child protection services to resolve it.

R: I haven't done anything like that yet.

M: OK, do you have anything to add to it?

R: Hmm, what I have to add is a a, your thus thing is a nice program. We pray and hope that it will continue. God will give you the the strength, the financial support so that you can help our little ones.

M: OK.

R: Some of them actually, they are having problems. We hope that your office will help this community to solve such problems.

M: OK, thank you very much madam. I really appreciate your time and your patience to answer these questions, thank you.

R: You are welcome.

M: This is the end interview, the time is 12: 56, thank you.

M:

R: Good afternoon.

M: Like I said earlier, we will be audio recording this interview for the purposes of transcribing and also they can listen to the audio and then make informed decision on

M: **Please in terms of Early Childhood Development, that is ECD, what are your department core roles when it comes to early childhood development?**

R: They say mens sana in corpore sano meaning a healthy mind can be found in in a healthy body. And mental health and the physical and psychological development of the child is very important to us. For us, against infectious diseases, we have the immunizations so for about thirteen immunizable vaccines that we carry into the field. Also their mental health as far as the children are concerned, we have now opened mental health service. Eventually we look at the psychological development of the child right from the beginning to adolescence. So it is hopeful that we can capture totally both physical and mental health of the child. There are so many branches at the child welfare province. We weigh them, look at their nutritional status and record it to see their progress. Whenever there is an event like a child's mental, psychological or physical growth it is not within the norm, we are able to detect the anomaly, that's very important and quickly bring in the specialist and other stakeholders so that we can correct it. Is it malnutrition, mental health, injury or whatever? We are able to see it so that we can do the corrective action so that the child's physical, psychological and mental can grow normal.

M: **Thank you very much. Please what is the relationship between your department and Early Childhood Development?**

R: The Early Childhood Development agencies at the district level, what I will say is that, the Social Welfare Division of the assembly handles the psycho-social and developmental problems because parents have to care for the children. Meaning there are so many marriages, traditional weddings, there are a lot of single mothers who are

caring for children at this age. It's either they are separated from the men, there have been a divorce or a teenager who has just had a child but has no father and is looking for daily remittances to this child and it's not coming. For that the Social Welfare, have their record for such problems and it's quite significant so how does this affect the child's upcoming, that's the mental psycho-social aspect. But when we are talking about is this child going to cough, susceptible to pneumonia, virus, that is, a virus that gives diarrhea or diphtheria or immunizable diseases that is the responsibility of the director of health services so that the vaccine are procured, housed and well-seasoned at the correct temperature. The donors give so at the temperature that should be given from overseas, down to Accra where it is also transported to various regional capitals, to the district; it must be kept potent and viable for use within a certain period of time and it is the responsibility of Ghana Health Service right from the Director General to the Regional Director General to the Director of Health Service to make sure the vaccine are potent and can do the work. Then comes to giving it and that is where we also train the community health nurses to also know how to administer the vaccines at the correct time so that it becomes effective, and also, how to maintain the cold chain system at the districts and sub-districts. When I'm talking about cold chain system, it is a system of keeping the vaccine in a refrigerator and arranged in a certain manner so that the vaccine does not lose its potency. Also, we have what we call the vaccine via monitor which is also able to detect that the vaccine that you have kept in the refrigerator is losing its potency therefore you either change it or use another type so we have capacity, skills and skilled personnel who maintain this cold chain and I am supposed to supervise so that they maintain this cold chain right from the district to the sub-districts till the day they administer the vaccines that is why you look at my map, there is what we call the coverage. These children, there are age groups from 0 to 11 months, from 11months to 59 months, that is before they get their fifth birthday. Every child in Ghana is supposed to be protected against these diseases and it is my responsibility to vaccinate them. After I have vaccinated them, before they get the immunization. There is a difference between the vaccination and immunization. And to get immunized, and also to get head immunity, if you are supposed to immunize hundred and you immunize only thirty you have nothing because when the disease strikes, it will affect them but if you are able to immunize eighty five to ninety percent of the children then you have got what we call the head immunity and you have had their health protected. Now if you protect their health by immunization, they should also feed and our nutritional officers also check their weights, their nourishment and their biceps. They use what we call the upper biceps circumference to check whether the child is growing in height and weight. These health and weight are compared in algorisms. These are curves which are able to detect that the child is growing and developing well. Now if they fall below the

algorisms then we are able to detect. So you see that it's a whole complex of systems and capacities that are able to monitor the normal growth and development of the child and with these measures in place, we are able to achieve proof and development of the child.

M: **Thank you Sir, we will still continue. Can you briefly describe to us the cultures that operate in facilities in this district about child welfare? Are there any special routines, traditions or activities that are peculiar to your district?**

R: Well, we are scientific people. There are norms and procedures that guide us. The culture of the people or a group of people is the way they behave in a routine manner and if it becomes consistent. If it's one or two things it becomes a habit but if it is a systemic routine thing that they do year after year and it has been documented, then it becomes a culture of the way they do the things. The Ghana Health Service has these performance norms. It's not like a cultural group drumming and dancing but it's a performance norm which the CWC as we are saying, they meet the mothers, talk to them. We have the clinical sections in the facilities, that is a norm of performance but they also visit their houses and they talk to them one on one. For children between zero and five years, we have them but between five and ten, ten and nineteen, you see that there is a deep. We don't have a well-structured system which catches adolescence and I wish that when they pick it, PPAG (Plant Parenthood Association of Ghana) was able to initiate the ten to nineteen section in their offices. I don't know how many still exists in Ghana. So you see that we do well between zeros to…zero to one is the best; that is zero to eleven months. We see the bone between the child, mother and health worker very strong, then after eleven months, it weakens to twenty three months. Between them, the woman thinks they have finished the eleven months and therefore they are free, that is when the children get malnourished because weaning the child and expecting to get pregnant one year, two years, after two years, twenty four months she will get pregnant again. Apart from what they have learnt in school, what exist between the mother, child and the health worker that enables the mother to come again? We have this culture of greeting people in Ghana, first greeting. If you go to Europe people don't greet, you come; talk and you see me next time. No, the Ghanaian culture is that people welcome people and make them comfortable in a comfortable setting and even if it's a tent a bench. The women are well dressed. On Saturday this man sung a song "she did not go for weighing, she gave birth and because she did not get a cloth to wear she did not go for weighing". It's was a very strong song. It a culture in which men gives their wives money to buy cloths and dress well and the child too to go to the child welfare clinic. And If this thing doesn't happen, there is a bond breakage between the mother and the husband then also between the mother and the health worker. How will we

correct this culture of compulsorily you have to give her a new cloth before she comes to the child weighing. What we advise the health workers is that, they should welcome them no matter how someone dress or inadequately someone dress but the mothers too have realized this. They would at least make an attempt to dress them and the children well before coming. We give them shelter, if you go to the back of my office; we have collaborated with an NGO in England to build a child welfare department. So comfort, the culture of making mother and child comfortable at the welfare. Three, the culture of not wasting too much time of immunizing the children and the culture of not making the environment dirty, unattractive so that when the child goes, the mother goes and they won't come back again. So what do we tell them to tell mothers so that it will reassure them that what they are taking home (the immunization) for their children are for their health? So we invite the culture of those who have succeeded, those who have already gone through the immunization and have had very healthy children to come around and show. Number two, the culture of mother-men pair, that is the woman-husband pair; the husband is accompanying the mother or the woman to the clinic and also sharing with him some of the issues that are concerning the child as the health worker is narrating it to the mother. That is also a special culture we have here. What can I tell you again? The whole atmosphere is telling them the importance of the jab that they are taking. I was in Navrongo in the year 1997, 2000 to study the meningitis outbreak, a lot of people died. And that time the then president, Jerry John Rawlings had to go to the north to jab, vaccinate or take the injection himself to demonstrate to them that the vaccine is good. They are refusing the vaccine. What will demonstrate that the vaccine that you taking is good. We have to get somebody who has succeeded in going through it all and also exclusive breast feeding. The content of the breast milk, that's the policy of exclusive breast feeding from zero to two years. Sorry the first six months and then supplementary feeding from six months and above, that's the importance of this. To the child, the content of the milk is adequate provided you give the child enough milk and you don't give water because there is a lot if water. That's what we immediately keep saying the child gets water from the breastmilk already. Don't give water and others things not proven to be non-infectious material to a child whose immune system is not too strong. What you have has already been sterilize in the milk, don't give it to men it's not theirs at that time. Give it to the child and let the child take it alone so that the culture of exclusive breast feeding, the culture of completing your immunization, the culture of bringing the child after a year. Let the baby come after a year. Do not stop, that's natural policy and the culture. After they have completed the immunization, let them continue for at least two years and that is where you will see anemia, a child that has not been fed well and the blood has gone. We also have the culture of ITN, Treated Insecticides Net to prevent these children

against malaria. Do you know that the insecticides have been imbedded with some insecticides so that when the mosquito lands on it,. It's a culture we are developing, the white man in collaboration with Ghana heath service that it is helpful for you and the mother care so that at the welfare clinic, ITN and also good nutrition, the nutritional options that are available for oil rice and they child becomes kwashiorkor so we have inculcated these behaviors and tendencies so that the mothers will pick. But the problem is what percentage of the mothers do this, that is the great challenge because is it the talking and the home visiting that will bring home or is it the pocket of the family that is bringing malnutrition after two years or is it the culture of fufu and soup and no more vegetables, no mangos, lettuce, cabbage, fish? Ok oil rice and then they put animal skin also called in our local language " welle " on it and you see the child going to school two to five years you look at her and then kalypo, you see oil rice and you see animal skin also called in our local language " welle " on it and it becomes a routine. How can the health worker develop the culture of inculcating this into the crèche, kindergarten so that the mother shifts from this bad form of nutrition to a healthy nutrition that is an indicator for the development of the child? So we have a dip there and then when they are able to make it to ten years, then we have the dip of adolescents which will come later.

M: Thank you very much. Please what can you say about the major health problems of children, age zero to nineteen years in this district? What factors in your view account for these conditions?

R: It's a very good question; we didn't talk much about zero to nineteen. Well from zero right from birth and the anomalies. Normally one important anomaly that we get is the birth palsy and mental retardation. It is very difficult to understand how some of the children get this mental retardation at birth. Is it through the way they were delivered, maybe the head was stuck and it pressed between the bones of the mother and compress the brain that one factor. Another factor is that, they take herbal medications; I'm talking about zero to one year. They take concussions to ease birth, birth easing concussions, at thirty six weeks it is okay. They use enema. This enema in the vagina to ease the birth, unfortunately, these herbs and concussions have effect on the child's brain. Sometimes it does not manifest anything right from the beginning; it's when the child goes to school and cannot learn and also iodine supplementation. They are supposed to exclusively take iodated salt, the mothers.

M: During pregnancy?

R: Yes during pregnancy, anytime. Not even during pregnancy, every day. The salt of the nation must be iodated. Why, because iodine is very basic in the development of the

child especially the brain, predevelopment. That is what we call cretinism. Very low levels of iodine have made the child's brain mentally retarded. So how do we inculcate this in the child welfare clinic? So that is the zero to one year and from two to five and okay if there is a congenital anomaly in birth like the whole in heart or anything that will make life not viable. So we have what we call Early Perinatal Death or IUDs (Intra Uterine Deaths), still deaths, perinatal deaths, late deaths and then death after two years. Now the perinatal deaths, most of the children can last for seven days. The reason been that, maybe there is a congenital disease that made him survive. There is a serious heart disease or a chromosomal anomaly, because in the nucleus of the cell, we have what we call chromosomes that make you what you are, make you different from me. These anomalies when they are severe in the children, they don't survive. There are serious ones, if I mention them; we have what we call trizomal 21 and so on. These are amazing things to you but some of them survive while others don't. The things that do that are either chromosomal or congenital anomalies and they survive that. Then we come to the infections of early childhood care. When we come to the infections like pneumonia that we don't keep the child well or doesn't have the nutritional, the vitamins, iodine, calcium and not growing well. These are anomalies due to lack of supplemental micronutrients and vitamins that the child lacks. If the child survives this against the immunization, then comes the infections. We have bacterial and viral. If you get measles, in those days when we were in Komfo Anokye, they were picking the child like chicken. Everyday about five, six ten of the children will die, in the eight's and early ninety's but thanks to the immune vaccines that was introduced to the first six vaccines, the child survived. And now due to the measles supplemental immunizations, the children have survived. No child has died of measles in the past five to ten years or so. So you see what the effect of what we are doing to the children and their survival and what iodine alone and vitamin A can bring about their healthy mind. So we do the iodine supplementation in the markets, we encourage, we do the survey in the market to see how many of the salt that the woman are selling contains iodine and it's always a challenge. How should we be able to inculcate this simple idea to the African man? If you go to Europe and America it's a routine. You are leaving my child without iodine? Are you sure of what you are saying? Where they know that iodine is not in the soil or in the salt endemically they make supplementation but here in Ghana, even if you prepare the iodine and send it to the market they won't buy it. They will go and buy the crystal one, there is nothing in it. So the mental health, look at how people are behaving, Shatta Wale and…. but with mental health, intelligent children. How many of our children, they are good academically but some have challenges and there is a variation. How can we make it uniform so that every child is taking iodine and is mentally developing well? So that is it. From ten onwards then comes the psychological problems

because if the child is a girl, she going to have what we call telack, the nipple is going to grow, what we call puberack, they will grow pubic hair, what we call menacky, she tends to have her first menstruation and psychologically it is a total psychological arena era than when before she menstruated or for the boy the first time he saw semen coming from the penis, And will say mama what is this? And some will not tell the mother so how many adolescence corners do we have? Can you tell the first time you had sex with a woman, then you are still a young boy. Am I lying? did you tell your mother ?, you never tell her and if the girl got pregnant. So how many adolescence corners do we have? That will address the mental, psychological and physical development of the child. The physical development is weights and heights and also the pubic hair and the artillery. We have what we call adrenal hyperplasia, it's a big word. Adrenal hyperplasia in which there are hormones. Your hormones especially your sex hormones become beautiful. The girl grows breast, the child becomes tall, lanky, beautiful and handsome. The girl also becomes beautiful but they have a psychological of violence, aggression, demanding, introduce the self into sex, learning about their sexuality, of thinking about their future development. Are they going to be like you, as you are good sitting down or end up in the streets? All these are running into the adolescent child's mind and can he/she grow normally with all these thought on her mind. They spend quite a lot of time out of your reach, in the secondary schools and that is where they learn a lot of things. When I went to secondary school that is where I learnt the words masturbation and lesbianism. They were all there at that time, that the Roman father will tell you that we don't want to see you masturbating or if you wet your bed inform your colleagues, they will beat you up even or don't wet your bed. We were in the boys' school so what were we doing all those days with ourselves before we got into universities, finished and before we were able to marry. Now before you hear they have their girlfriends and they have everything. We have what we also call acceleration, note the word. Acceleration means that in the past, these hormones of development were lower. Now these hormones develop early and the children get to know themselves early that they are sexually active and they become more aggressive earlier. Nobody can tell whether it is culture or development that has brought this acceleration but the children know things and know themselves better than us. So the adolescent corners are supposed to handle all these things. One, sexually transmitted disease ok, that's the physical aspect of it. You go and have unprotected sex with somebody, are you going to ban your children from going out? No, but are you going to also suppress them when you see that they are having sex? No, it's to teach them the right way of engaging the opposite sex and also carrying along condoms when they feel the edge and cannot resist it. In our time, you dare not, so why were we hiding our sperms and emotions?

M: **Good question.**

R: Only God knows. Why were we hiding our emotions? And I graduated and even went to Eastern Europe and spent eight years while hiding my emotions. So most of the time, the children learn it from themselves and now that there are videos and other things. Where were the videos at that time? People learn it then from peers who told them about, you know, how the female sexual organ is and how you can enjoy going to a woman. Those who did it got women pregnant. But now the psychology of the adolescent now is totally different. The physical strength is ok because they have a lot of food around, they eat but the psychology. The mental and psychological strength of today's child, African child is the biggest problem and that is where mental health is also been developed and inculcated into the service so that, it will address the needs of adolescence. But the problem is that how many adolescent corners do we have? We don't have many. Even if you go to the bigger institutions, they still focus on clinical care. Politicians focus on clinical care. If they will invest money, they will invest money that will treat malaria; they will invest money that will get the medical team to operate but they will not give you money to look at the mental health of the adolescent. And you and the mother and father too, it is your responsibility at home to look at the mental health of the child. Now they say tramadol, marijuana and some countries you know, marijuana has been legalized. They buy it and smoke and they come home with all sort of behavior. Now that there is no money in the system, the child has finished and he/she is unable to further it. The child has Bsc and sitting with you at home, no work, no hope that work will come in the next twenty five years. What do you think will happen to the child, it's a big problem? There is no plan, the whole cycle if you look at this man, Kyiriabosom who was telling us about the industries that existed in the late sixties by Kwame Nkrumah and those people and no politician has attended to them and there is no work for anybody apart from teaching and maybe we the doctors and nurses, what work again exist for the adolescent? And what they are asking the government is that they have paid so much fees to finish nursing and the girl is sitting at home. How will you psychologically tune this girl to understand you at home? Now to feed you if are not careful she will bring pregnancy home so you realize that from ten to nineteen, we have a problem in Ghana. Seriously!

M: Thank you doctor, we will still continue. Please, can you tell us about any form of initiatives and programs the district has for addressing growth and development related problems of children? Example, malnourish, disable, over weight.

R: I will talk a little about obesity. We knew in Africa that its malnutrition that is worrying us but now obesity and its related diseases like early childhood diabetes is becoming a problem. Early onset is the type 1 which you need insulin, late onset is the type 2 which you don't need insulin but the children put on a lot of fat. They children over eat and it's

also hormonal, its pituitary and what we call lepton adipose lepton and how good fat can be accumulated and bad fat can be shed. The balance between eating and exercise to make a healthy mind and body is missing. As soon as the live, even in the secondary schools, they do a little. Some of the schools do well by getting them to do exercises nut when they come home, they don't continue. So obesity, body mass index, the range; above twenty nine (29) I think is a problem. So we weigh them, the school health is supposed to cater for the weight of the children in school and they are supposed to refer to us people whose body mass indexes has exceeded the norm but they don't report. But obvious ones like clear obesity, now they refer them to Okomfo Anokye. What I'm telling you is that it's hormonal, super adrenal, pituitary and then the adipose tissue. These are complex concepts but I'm giving you a generalization of it. It's either in the brain or the pituitary, super adrenal or the adipose tissue. These combine in several ways, some are genetic, acquired by over eating because the food is there so you have to distinguish this overweight issues. In the schools, the JHS to our level, we go and do the weighing and we inform the teachers to alert us if the child is becoming overweight in the school then we refer to the specialist for them to attend to. When they live school that is where the problem is because between ten (10) and nineteen (19), there is an algorism of your weight and height and if we don't achieve this, or the BMI has over short, then you inform us to send you to a specialist. This is also the time for depression. You remember about a year ago within three weeks, about 10 adolescents committed suicide. Did you hear it?

M: Yes I heard it.

R: What did the government do?

M: We didn't hear anything.

R: Now it's quiet. This aggression and suicidal tendencies is coming from what we call adolescent depression. When we enumerate all these, what is going to be my future? Do my parents care for me? Apart from feeding me, what is the potential? Or all of a sudden you lose your job or your income has depressed and you are supposed to care for this child to maybe university level. You have been able to just get him/her to Senior Secondary School (SSS). The child is sitting at home, what do you think this child would do to you? So the suicide, aggression and children attacking their parents and thinking that they have misled or disappointed them in life just like you were able to reach your doctor and now it's my turn and I do not have that opportunity. The only thing is to set psychological institution. Our mental health is just behind, they are not well dealt with, there are mental nurses who have gone to training, some are now training them for the two or three years training. Initially, they were just two weeks, three weeks and they

will come and say they are mental health nurses. You can't be a mental health nurse with three weeks. The nurses are now properly trained, the doctors have also taken interest, and there is a mental health corner at the ward so that if somebody goes out the way but that is not only the mental, the psychological too. The person is healthy and doesn't have all this mental problem but always the stressor is coming from their peers. Rich people's children who have succeeded and you don't have, what are you going to do? And it is not a society that has the welfare system that voluntarily gets children to enter schools or get jobs for children.it is recently that NABCO is coming in for these graduates. In our days, we had vacation employments, when you finish, the long vacation; June or July to September. Two or three months the department will just come and pick you and introduce you to simple things in the office, the factory. Before September, at lease you have some job, you have some pocket money and they have pushed all these things to the background. In a society where you complete school and there is no work for you to do, where are these adolescents going now? So you see that the initiatives by the government is the NABCO, do I even think that we receive them here but when they come with the mental problems, we direct them to those who are healthy but are under stressor we get them to psychologist and other people, the church, now the churches are doing well. Are you around here or you are from where?

M: Yes, I'm in Kumasi.

R: Do you know Rev. Obofour?

M: Yes

R: Although they are collecting a lot of money, which is worrisome but they are doing a lot of great work. They are reducing the number of people who will otherwise have committed suicide. I'm telling you, because that is why I'm a director here. I will not go and destroy it but we go and talk to them not to go to the excess of collecting so much money from people because they already don't have but giving them hope. I don't know what is going on in the mosque but in the churches, some of these charismatic churches, they are doing a great job and that is why Ghana is almost always calm despite these… compare to other countries that surround us, you will think that this our destitute situation a lot of explosions is going to happen but there is a limit to everything. For now, the churches have done a lot of good job.

M: Ok Sir, what is the district also doing? What initiative does the district have for the disabled ?

R: What we do is that, we engage the churches.

M: …when it comes to the disables.

R: The disabled, we liaise with the social welfare. I had one child who had bell's palsy, we liaise them with the social welfare. Last year for instance they bought them fridges and other things worth one billion. They distributed them here, so we are part of the committee that screens people to see that actually they are legible to benefit. If they are sick like polio, that one there is little we can do about it than to get them employable skills. If they are very young, we treat and refer to social welfare so our work at the district for disabled is to liaise with the Assembly. Our work when they report at the facilities, we liaise with the Assembly so that if there is an employment opportunity they…. But there is a whole huge of people who are healthy and yet they don't have work. So there must be a balance between the disabled and abled people in terms of… I want to see a day in which adolescent children that have no work to do are also assembled and given fridges. You get what I mean? [Inaudible 00:46:49] there are a lot of children adolescent who are also roaming about in the streets and they may need just GHC100.00, GHC200.00 or even a fridge to start working and they don't have and I think our job is to help screen so that they will get these…

M: Please Sir; does the district have some initiatives like maybe home visitation of parents, any other intervention or seminars?

R: Yes, we do workshops on HIV. Workshop, the best dangerous thing that can ever happen to you is HIV/AIDS so this is the trying time. We go home, so when we go on a home visit we tell the mother times are difficult but it is worse we leave this child to an STI back home or leads her to prostitution and bring you back HIV/AIDS rather try and find some employable thing from the selling or something to occupy them. So the home visit is one aspect then we also teach them on the STDs. We visit the hospitals and distribute condoms and other packages so that even if the child is stubborn and reaches a situation where they want to engage in this practices, he/she can still have the opportunity of picking a condom to protect. Also Family Planning (FP), we also teach family planning. Do you know that Ghana is still lacking behind in introducing FP to second cycle schools? Before we understand this, we frown it, the perception is that we are teaching the children bad thing too early. That's the perception; even if you go to high quarters I'm sure this is what they will be talking. But the children already know this. They know emergency contraceptives; now they know cytotec, they know them so what are you talking about. We wait till they get the pregnancy, after the abortion then they try to introduced them to FP's. No and the FP is given to the mothers. Family planning is given to adult mothers. FP and contraceptives are not for adults but they go and get it. Where are we? Tell me. I'm going over it; we don't consciously teach FP and

contraceptives to SHS, it's a taboo. We went to catholic school to preach contraceptives; they drove us away but how many catholic children are getting pregnant? So its hypocrisy, we have to find a way to get this adolescent help. It's a big question but at the time it will reach if we had done this for this adolescent, just letting them know the good and the bad, we would have prevented a lot of pregnancies and maternal deaths. Some of them are coming from septic abortion so our initiative is restricted to adult mothers. Number two, adolescent help we are still talking about HIV and STDS without focusing on what the children can do to prevent these STD's because we don't preach adolescent contraception. Seriously, and where are this adolescent farm, they are mostly in second cycle schools or if they drop out of school then maybe learning trade but as of now I tell you that it is mostly their own initiatives and what they hear from their peers that they have done it and has been successful and they also go ahead. It is never a conscious effort by us the health workers who will come. Have you seen someone wearing school attire come to you doctor that sir, it's time for you to give me my contraception, it does not exist. And yet we say we are fighting STDS, HIV. In Europe, it is normal for you to visit an adolescent parent as a second cycle student to learn and to pick contraception because you are matured and have the emotions but wants to prevent yourself. We are still far away from it that is what I want to tell you is that until we consciously, not forcing contraception to children but open the avenue where people can learn contraception to prevent themselves well.

M: Thank you Sir, please can you tell me about the funding situation related to the element of child and adolescent health and development interventions in the district?

R: Yea we have funding from GARVY for the vaccines. For adolescent health, at the community level we have what LCHMC prime, MCHMP, Maternal, Nutritional, Neonatal, Child Health Funds and It comes periodically that we give to the community level; to community health nurses so that they can use the money to go to the homes direct. The money doesn't stay here, the money dump on us, huge sums of money but nothing stays in the district, it goes direct, over us through a directorate. The purpose, go to the home, use some as chop money, we use some as transport, go to the house and ask how many pregnant mothers are there, how many children zero to eleven are there, how many adolescents are there, listen to what they tell you about their health, don't go and force anything on them but listen to them about their health problems and even their perception about health, their perception about malaria. So when he is getting it he goes to sit in the sun or if you are pregnant and your feet are swollen, you are going to have a sign. We have what we call pre-eclampsia, the pregnant woman will start Fitting, and they don't know that when you swell like that you will faint; they tell you the child will be a son, so some of these things, go and explain it to them for the

adolescent. I have been once like you; one day you may meet somebody. One day if you meet them, make sure that day you carry a protection and for me these are the type's protection. It will save you from serious diseases like gonorrhea, HIV, syphilis and be able to send this message. There is one thing telling and another sending a message and third thing, the message going into the memory of the person and the person keeping it. And at the moment that he/she needs it, she will recollect and pick what was said to her. Now HIV cases are picking up again in Kumasi because we have relaxed. It's like a wasp and rotten meat, a blue bottle and rotten meat, so you need constant engagements. With all the millions of cedis that came to Ghana on HIV/AIDS, what do we have on HIV/AIDS except the headquarters, the huge building at the headquarters? I was expecting that at the district level, there will be structures, adolescent corners everywhere, there is music, there is Shatta Wale, but after they have listen and danced then you introduce the education. Only a few will because they shy from our directorates, the facilities so they meet they people only at the peer level. How are we going to overcome this barrier? So we need to do a lot of work in this direction. The initiatives are there as I have told you. The MCHMP funds, we also get global fund for malaria, tuberculosis and HIV but it is not as good as the MCHMP that is currently coming for. I have told you about GARVY, MCHMP, I have told you about global fund.

M: Thank you. Please can you tell me, how does your outfit record information on special children?

R: That's a big question. Now let us define special children. Who are special children? In my understanding, mentally retarded are one, disabled children and any child that have become destitute or ill or not progressing in health as required. There are special schools in Accra and elsewhere. There are special schools for monguts, trisomia and all those ones that are serious. Then we have also these NGO who are operating the homes. When I was in Obuasi, there was an Australian woman who was operating such poor children not necessarily retarded. She will pick poor children as well, feed, clothe and school them, they need special schooling, special teachers. For instance, you don't go and ask a monguts as in other schools so they need special teachers that here in Kwabri, we don't have one special school.

M: Ok thank you. Please describe for me some of the interventions you provide for parents who have children with special needs?

R: Children with special needs, who are children with special needs? There are a lot of them, we have what we call, is it irritable children? People who cannot pick the lessons, I don't know, there is a special name for them. They are unable to pick their lessons well and slow learners, are they special people?

M: Even some tantrums.

R: Yeah, tantrums and truants, there are so many types, maligning. He comes to the school and then leaves. Then also children on tramadol, glue, these are all special people but how will you identify such a child in school; Irritable children, tantrums, truants, maligning, on tramadol and on glue and even marijuana. First identification and alcohol syndrome children; First you have to identify but in Ghana you cannot. Take the ordinary JHS teacher, will he able to see an alcohol syndrome child? The best one you see is children who are chronically truants and tantrums but are the able to see glue and….

M: It will be very difficult.

R: Yeah, very difficult. Tramadol is coming in recently and alcohol syndrome, we take it as normal, "child of a drunkard" but there are symptoms and signs, even the facial appearance, I have one doctor who was drinking akpeteshi constantly. One colleague doctor constantly gets drunk in the house and he has children, so you could see that they have alcohol syndrome, how will you be able to help such a person? First of all what are their needs, is it vitamin, mineral, mental deficiency? Is it correctable or uncorrectable? Can an ordinary physician manage it or you need the services of a psychologist and a mental health worker that is where we are. The link between us and the education service on this special teaching is missing. So they pass them all to failures and success, they are writing the BECE but I tell you some are even pregnant because there are destitute homes. Not that the parents don't have but there is so much conflict in the house that the children are polarized and there is always fight in the house and the children have stayed with this and are going to write examination, what do you think will come out of it? So it is time to introduce Psychologist and mental health workers to JHS and SHS. As of now, we are relaxing and expecting that the patient will be sick, they will carry him, "he is mad oo" that is what we know about mental health and whore besides you, you go see somebody, I think I'm getting crazy. Will you go?

M: No.

R: Until you get the craziness. But in Europe and elsewhere, I think your behavior is changing so maybe see a psychologist or a mental health worker to help you, it normal. It's normal that even at the family level, they have a psychologist and a mental health officer who will be visiting them regularly. Someone saw a doctor Akwasi Adjei said almost half of Ghanaians are mentally ill. Have you heard that he said that?

M: Yes I heard.

R: He said that some time ago, last year. Almost half of Ghanaians have a kind of serious mental problem. Who is going to care for this? Or almost half of Ghanaians have social needs for something and it's not only adults but children. So after the mental health bill had been passed, I was expecting that after its promulgation, they will discern and train people at various levels but they have limited it to the hospital. Is there a mental health bed at the hospital? Is that how mental health is been treated? No, and to lift us from this barrier of craziness that if you mentally not in order, it's not anything unusual. Look at the amount of stress that comes to workers, no money and others. It is not a shame to say that you are mentally over stressed and you need help, as soon as you mention it, the people tag you that you are sick and unless we over go this barrier and look at this social needs especially mental and psychological health needs of the adolescent, we will end up in the sort of people we produce. Maybe it's a mineral, iodine, it's a, b or c. you will do the lab test, maybe it's chromosomal. You do a lot of lab tests routine before you enter secondary school in Europe, a lot of tests that you will go through to see if you are mentally sound before you enter the school, nobody will allow you there.

M: So in a nut shell you mean because you don't have any facility here?

R: We have the facility but have been related to receiving patients, that level. But to visit home, to visit the family, family doctors are now emerging to visit the home and see what the mental status of…

M: So that is the problem?

R: That is the problem. The go there to see your hypertension, diabetes but they don't enter there to see whether are you mentally sound this morning, no they don't. Or is the family mentally sound, to make it normal for them to also respond to you that, yesterday I had a quarrel, the children we had this and that can you help us, it does not exist. And if somebody makes that attempt to, the tags, stigma. Even your specialist who will see to you will start spreading it to others, "not knowing this guy that is walking…." This gossip nature of our culture is making us late. In Europe and America it does not exist like that although there are a lot of mentally related crimes so you weigh the balance. Because there is a lot of exposure to these drugs there, the level of mental related crimes is also very high but we are coming and this is the time that if we organize ourselves well, these special needs would have been well catered. Once the mental bill has been passed, these special needs will also come in and later we are sending vaccines to homes, we want to care for pregnant; we will send people to homes to look at the mental and psychological needs of the people at the home level as routine, that one is missing.

M: **We will continue. Describe for me some of the interventions you provide for parents who have children with special needs. Whether maybe you screen…**

R: Yeah, the screening, we screen sickle cell. The special needs, sickle cell is among, do you know that? I failed to mention sickle cell. It's a disease that needs special attention the blood cell is not circular; it's just like a cell, it goes to jam the peripheral and causes a lot of problem. So sickle cell, Okomfo Anokye does this screening and the hospitals have been encouraged to do the screening at day zero now it's there. We were reporting the yearly number of children, who have been screened, neonates who have been screened, so that is one. For other specialties, we don't.

M: **Okay**

R: Where do we screen? We screen people for deformities like, birth defects and what do we call it, the legs are turned upside down, there is a name for it I have forgotten the special name for it. And then holosis valgums, genu valgums, genu excavatum, carina excavatum, VSD, that is vulvulaseptal defect. We screen also for mongoose, no they detect it, we screen for cleft palate, so these are people who needs special attention. Do you know cleft palate?

M: **Yes.**

R: We screen for cleft palate, mongoose, sickle cell but some of the fine things like tenasyndone, it is an anomaly and we call it chromosome 35 S.O. A mongoose is trisomial 21 that is instead of S.S at chromosome 21, it becomes SSS and this tenasyndone I'm talking of, we call 45 S.O in which you don't get the 46 chromosomes, one is missing and they have a special feature, they come with a wordneck. Apart from everything, their stature and other anomalies, they have this wordneck something so detectable early but our people do not see it till they are grown and whatever but what can you do to an S.O. In African very little, what can you do to a mongoose but we have to create these special schools. In Accra there is one, I don't know here in Kumasi there is nobody. Have you seen one?

M: **No.**

R: No, they tell you even the healthy ones we have problems. If they have the culture of sending you early to a river to finish you up. The very bad ones, they don't even let them live. Do you know that?

M: **No I don't.**

R: Yeah you don’t know, if they give birth to you and your deformity is so bad, they won’t bring you home, they go and leave you somewhere. You will be called “child of water” But do you know Stephen Ox?

M: Yes.

R: Stephen Ox, who is one of the brilliant physicists in England? He developed this anomaly, he developed what we call, I have forgotten. He developed something that made him stiff and it was progressive but the brain was there. So who knows that if the president becomes a cripple we will be safe?

M: Nobody knows.

R: If we get a cripple president we will better than Akuffo Addo or Rawlings, so the Roosevelt was lame. Do you Roosevelt?

M: Yes.

R: He was lame so those people who have special needs, it’s time we pay attention, maybe one day. They are very meticulous. I saw one cripple with the two feet, his farm even an abled person cannot do it. He had cocoa, cassava and a lot. A disabled had been able to do this and how can’t a full blooded person can’t do it. So the schools must bring up but it is very difficult to run to bring them up because of their special needs. The bus you have to create a special gate, the stair case, the hospital you have to create something special for them.

M: User friendly.

R: Yes, user friendly hospital. I’m waiting for the first disabled hospital in Ghana in which the stairs, carriages and everything are specially for disabled people and let’s see what will come out of it, whether we won’t get a president from there.

M: What can you say about the capacity of health personnel in the district to deliver efficient child health services.

R: I am sure of our section; those that we have trained to deliver the seed of these field are well trained and that is how come you haven’t heard polio for some time now. You haven’t heard a lot of cripple people begging in the streets since 1989 that we introduced the first vaccine. Now we are about eleven or something that means that the thing is working if we focus on it, it works. So what should I say?

M: But they are talking about number, skill set and geographic coverage.

R: The number of skills, the community health nurses, we have about forty (40). Their skills, they need it to immunize, they cover the whole of Kwabre East, a population of one thirty two thousand (132,000) and there are four (4) sub-municipals, thirty five (35) communities and thirty one (31) cheek zones, the number of accurate points is sixty one (61). We have sixteen (16) facilities: government is six (6), CHAG one (1), private nine (9), so we cover all this with the number of nurses who go and do… so they have the capacity to do the work and that is our strength. We were the first in Kwabre in 2018. The whole Ashanti we were first in performance, we were able to send services to the in highest and the best performance. If its immunization, we able to do to cover all those eligible children. So this is the award that gave us for 2018 and we are hoping 2019 we can do the same.

M: Congratulations. How do you identify personnel capacity deficiency in delivering child welfare services? What interventions do you have for personnel who are lacking in capacity?

R: For instance, we do workshops and when we do them, we have pretest and posttest. In the pretest we are able to detect those who have forgotten and during the workshops, we correct it so we conduct what we call in-service training program. It is a stratified or a well-structured program for all categories of staff. We don't go and pin point something that you don't know but we are able to detect by using this strategy. When we call them to a workshop, we do a pretest. We also go on the job training, field work. As they are doing the work, we supervise them and in the supervision we are able to detect those who are veering off and all. For instance, at what angle should you send a pin, we have angles, the needles for TB vaccine, this angle for diphtheria, go this way or give it here, don't give it there. Even at the nationals, people have problems so on the field. So one is workshops, one on the field and the third is examinations. Examinations come from time to time and we get the results and we also able to correct and retrain for the underperformance for the cold chain. We have cold chain monitors for the vaccines and every day during the week you should be able to mark which vaccine and the temperatures. So when we have a problem or we hear that something has gone wrong, we tell you that if something goes wrong, report to us. If you don't report the vaccines will spoil so they report. Through this reports that we receive we are able to tell the mistake that they have made and in a stream way, the monthly report that they submit to us, we go through and are able to detect people who are falling short of their responsibilities.

M: Ok the last question. In what ways do you think the health directorate can support parenting in care and nurturing of children?

R: This is a big question. Maybe it is a faculty that is coming. Parental care through the Ghana health service because as of now, the directorate only reaches the parent through the child, through services of the child. So how can we now encourage parents of their child? Through the child welfares, the home visits, adolescent corners, trough parents coming to weighing. These are some of the ways that we can encourage parents but we have never sat, calling parents on a workshop. It has never flown from the national to the region to us. Maybe we should start it, that all a parents of children under five years, come for a workshop on parenting from zero to five. How do you sit a child who is crawling, we have our traditions, you fall and rise.

M: Like when they are crawling.

R: When they want to step, what do we do? They hold you, then after they leave you expecting that you do what?

M: You walk.

R: And God know the number of falls from the bed. I once came here when we were doing the immunization; the child was a sickle; SS and then they immunize her when we were doing this measles immunization and she fell seriously sick and had to admit and treat. The mother wanted to blame us for the immunization, that the immunization is what has made her child sick, it was the first day exercise. They brought the child to me as the director, when there is anomaly we call it AEFI (After Effect Following Immunization). These are people who after you have, the first day or a few minutes after you have immunize, up to twenty eight (28) days, anything that happen to the child report to us. So they brought this child the first day, as soon as the child was immunized he brought in five (5) hours, doctor, this child is dying so I had to step in and go treat the child. After the child had become well, the mother wanted to put the blame on us, then we saw a scare on the child's head, what about this one? It was there and then they said, the child fell from the bed. The child was SS, we should have also taken precautions, now you adding problems so through the screening they are able to bring…. I have told you about sickle cell, early screening, they are able to get the SS one, that's the worst of all but AS you can run around with it. We do what we call Electrophoreses. When they see that you are sickling positive, immediately Okomfo Anokye they would do the Electrophoresis for you or anywhere they will do for you whether you are AS or SS and then we start giving you treatments. So some of these early screening exercise interventions, abled children who have otherwise died early to survive especially sickle cell and now the department of Endocrinology is also bringing another thing, early diabetes. Screening for early diabetes in children, they have them now. They will treat them for malaria; every small thing, they check for malaria, they will have to go and

check the blood sugar and if you detect that its diabetes or the sugar is high, it is likely to be childhood diabetes. This is something we don't know. A lot of children have died because doctors are misdiagnosing ealy childhood diabetes, so these are some of the things, interventions that we can do to children to detect forms.

M: **Thank you very much doctor. I'm most grateful for your time.**

R: Go and edit it, we've talked a lot.

M: **I just speaking to the District Health Director, time now is twelve minutes past three. Thank you very much, this is Solomon Anapey.**

M: **good morning sir. Thank you very much for your attention.**

R: welcome

M: **ok, please in relation children and adolescent, what role does your department play?**

R: those are the vulnerable in the society, children, adolescent, women, the aged, they are all vulnerable and as such we take care of their concerns. We address issues relating to children, adolescent, when they come to our domain as police officers. Their welfare is our concern. Generally, yes, I think their welfare is paramount to us.

M: **ok, can you also tell us some of the services you provide to parents caregivers and children?**

R: in fact when they come to us for any reported case, we do our best, if has to do with going to apprehend or invite the parents - normally they come with maintenance issues - they come with maybe the children having been defiled, the female gender they face

this challenge of defilement, rape and if it has to do with a male or boy, they too sometimes they are sexually abused. So when such issues are reported to us we take steps to actually find out the offender. We arrest, we question, if evidence is sufficiently enough we send it to court for the law to take its course; but if it has to do with a maintenance issue, we invite the man why he should he should not be responsible because when you give birth to a child, it is the responsibility of you the father or the man to bring up the child, his or her upkeep is your responsibility. So if you are failing in such a duty, we find out. And then again if we need to process you for court or we refer you to this social welfare people to come in and maybe peg maintenance fee for you too...which is mandatory. Every month come and pay...so we offer all such services. There are some of the parents even when they bring those children to us who have been defiled, we issues medical forms to them, they will tell you they don't even have money to take the child to hospital. We do volunteer, I use my own personal money to take the child to hospital for the doctor to observe, examine and treat come back with endorsement paper for...in fact we do a lot.

M: this same question. Is there any situation where sometimes you have to counsel parents or caregivers or children to return to school, maybe also issues with stigmatization, children with disability?

R: yes! We do a lot. Normally when they go through this trauma they find it very difficult to go back to join their peer groups because they might be teased and all that so we counsel them, we have to actually console them, assure them that they should back, there wouldn't be anything of that nature and then we take pains to even go to the head of the school, we see the head teacher in charge and we have that rapport with the person that, he should ensure that when the person comes back the peer group don't jeer at him or her, for that matter we go, we take that step.

M: and what about children with disability too?

R: oh yeah! They too, but for them I don't think they face that...they don't face the problem of being teased because of their disability; but if they are also abused in one way or the other, they come to us, we also counsel them. Of course they all have their institutions where this handiworks, we go to the school to see the head and also entrust the person with the care of the head of the institution, and they fit back into society.

M: based on your experience working in this community, please tell us about your observations and how parents, adult child relationship in this community is?

R: yes, I have been working here for the past 7months; I came here somewhere last October. I was posted form the division and since I came and assumed charge of

DOVVSU, I realized that cases of defilement, rape are very prominent here. You see, there are a lot of developing sites here where settlements are not all that complete, so children face problem of being defiled, women rape and all that; and all due to the fact that the youth, the boys in general around here too some of them have taken to these drugs tramol and the weed and all that so you will see them hiding in the bushes and these developing sites, at the ghettos, having taken that drug and getting booze or intoxicated, their only expectation is to exhibit their sexual drive on these vulnerable children. So the slightest sight on any of such children around the developing sites, they will lure you into the bush or in an uncompleted building and have a field day. It is very rampant here and because of that, as the officer in charge, I don't compromise with such cases when they come to me. The moment they come I force my investigators to expedite action, get all the medical evidence that we want in the case, the material evidence that we need to and within a space of 24 to 48 hours we take the case to court and all of them are currently in remand now and...

M: ok, thank you very much. Sir, what interventions do you have in streamlining or aligning parents/ guardian child relationship that are inconsistent with child welfare environment?

R: oh, we do a lot of education. My staff, we use to go around schools, sometimes we organize workshops, and even go to churches when the need arises, we are invited, we go and give lecture and impress upon parents as to how to handle their children so that they don't fall prey to these misfits along the developing sites for them to capitalize on them and...We educate them a lot to be actually provident. They should do well to provide for these children adequately so that the girl child will not be lured by somebody giving him or her, a token, "come for GHS5" and then he takes advantage. In fact we do a lot of sensitization with the parents in that capacity.

M: sir, what intervention do you have gain in streamlining or aligning parents/guardian child relationship for parent/guardians who have children with disability that are inconsistent with child welfare environment?

R: those with disabilities, you know we have this institutions, vocational schools around who actually take care of these disabled children. So we always recommend that such children are enrolled in such schools where they will learn vocations or trades. With that when they finish they can also establish themselves, these shoe making, kente weaving and all. They are all trades around for people with disabilities. So there again we educate children or the parents of such children to take advantage of such vocational skills or schools around and then let the children benefit from such packages.

M: **thank you sir. How do you assess the type of coordination that exists among child and adolescent related institutions, example, social welfare, ministry of gender and social protection, the division of family health of Ghana health service, DOVVSU, ministry of education of Ghana education service?**

R: oh! As far as I know or to the best of my knowledge, all the institutions you mentioned here are up to the task, because the welfare of the child is paramount to everybody, for that matter all these institutions. So when issues regarding children come to our knowledge, or come before us, we treat such cases with priority and preference. I think I cannot mention any institution that drags misfits regarding children's issues. I think we are all up to the task.

M: **could you tell me something about the core issues in monitoring or supervising children related organizations in terms of compliance to national or international regulations and rules?**

R: that question I cannot...

M: **let's continue. Okay, please describe what makes children unsafe in this community, in both economic, social and...**

R: yes, yes, yes! Like I was saying, a lot of parents are not caring. They are not providing the children the basic necessaries that they require. A lot of fathers go about marrying two, three, four women; they give birth anywhere anyhow unattended to, not considering the economic situation on hand. You will be surprised to hear some of the parents, the fathers are not working, they are not in any employable job but yet they take delight in dating two, three, four women and give birth there and then. When the children come up they don't show any attention, they don't provide any service to the children. They leave the children unattended to, to their fates to do what they like, so in fact socially, economically it is a problem in this community. The children are there who don't have fathers - the father exists alright, not that they are dead but they don't care about the children.

M: **sir, do children with disability feel safe in this community?**

R: ok, in my community Kwabre here, I can say they are safe because we have not received reports of them being attacked or brutalized by...no, they are safe.

M: **Sir, kindly, tell me about your perspective on rehabilitation services for children in this district.**

R: well, when you talk of rehabilitating, I don't know.

M: **maybe information services, care homes, access to therapists and you know...**

R: ok, I think that has to do with the disabilities or those with disabilities. Like I said there are institutions around who take care or train those with disabilities. Children in general, I think with all these institutions that you mentioned, a lot is being done to safeguard their interest and well protected to the extent that they don't face threat from anywhere because as soon as we receive reports involving children, we take necessary steps that will bring us the needed results, like if we have to prosecute offenders, we do that with dispatch; those we have to refer to social welfare we do so and they cooperate a lot with us and we work hand in hand.

M: **we have just about some few questions more. Sir, what is your view on formal or informal child protection services available in this community?**

R: hmm, for me, I will say they are doing their best, but yet still they need to be well resourced. They should be more resourced than they are at present, because social welfare for instance, when you send case there, they face a problem of transportation and all that so sometimes it is we the police who have to see to the transport of the officer, the child involved, here and there. So it all bothers on the economic situation of the country. So I will plead that these institutions should be well resourced so that when issues come up no officer will be dragging feet by refusing to do one thing or the other, blaming it on financial, sometimes they even have to come to court to testify, they will say they don't have transport to do that. So I will say they should be well resourced.

M: **ok, sirs, what type of cases would you use the formal system and what are those that `you will use the informal system?**

R: ok, by formal you can explain.

M: **maybe by formal they are trying to talk about the court and then the informal maybe trying to settle it at home.**

R: yeah, with the rape, defilement and indecent assault, all those ones I will prefer they go the formal. When it comes to no maintenance, I think these ones are negotiable.

M: **ok, so when you talk about non maintenance, maybe a father or mother refusing their roles or responsibilities.**

R: yeah! Or sometimes the woman may expect him to remit her GHS300 a month but you are giving her GHS150 which she feels it is not enough. That one too, the man will come and explain that the job he is doing or he is unemployed. So for the non-maintenance issues, I think that one, they can be resolved by ADR, alternative dispute resolutions,

that one at a round table, you can sit with them hear the views of both parents and maybe advise, "oh, ok if you cannot pay the GHS300 that the woman is demanding try and give her GHS250 at the end of the month". I think we have been doing those ones and it works.

M: ok. We would also want to know more about another case that your outfit adopted the informal scheme to resolve. What were the outcomes?

R: oh yes! Like I was saying, the non-maintenance, when it comes up like that I try actually to see the position of the man at present as compared to how it was previously. If it has to do with losing a job, maybe previously he was in good employment; he was giving you the woman so much. If the woman can confirm or testify that, "previously he was giving me this amount but now this", then that one it beholds on me to also talk to the woman, "oh, considering the man's position at the moment, you too do something to augment his efforts. If he is giving you GHS300 previously and now he says he cannot afford the GHS300, take GHS200 or GHS250 but you too add water somewhere to come and supplement..." and most of such issues work when we meet like that, when we meet viz a viz, one on one.

M: so sir can you really recall any o such situation that you last dealt with and what the outcome was?

R: ok, that one then I should have invited the woman who brought you, because the dockets are...me, when they come to me like that, after writing and meeting with them they go back, but the records they have it. I don't have any name or docket here for you.

M: oh no! It is not about name but maybe the last issue that you sat on and what the outcome was.

R: I think about less than 4weeks ago we had an issue like that, where the boy I think he is a driver, he had an issue with the woman and they were actually living together but when...he is a trotro driver, when the vehicle was taken over from him, he could not actually pay the house keeping money he was giving the woman, I think he mentioned that he use to give the woman GHS200 every...but when he lost the job, he cannot actually make it so now he gives her GHS100 a month and the woman said it is woefully inadequate and it came to me and I said "ok, the woman, you can also assist him", even the woman because of the inability of the man to provide for them in the house, the woman got offended, packed her things and is living with her parents while the boy lives in the family home. So the woman expects that at the end of every month the man should send the GHS200 and the man said, no because he has lost the job as a result of which even you left with my child to your mother's place, I cannot pay the GHS200 this

time so please let me give you GHS100 that I sue to give. The boy says sometimes he gets spare driving, then I said he should try as much as possible to make it GHS150 for the woman to also find the supplement somewhere and true too about three to four weeks now, I think they all agreed, the man said he will make it the GHS150 suggested and the woman also accepted that if she gets the GHS150 at the end of every month she will take it and also supplement it. I think the boy is about 3years old, he has started nursery school. So we have a lot of such issues.

M: thank you very much sir. I really appreciate the effort.

R: you are welcome. I am also happy.

M: thank you sir.

M: Can you please describe to us the general work of your institution.

R: For social welfare economic development, we've three main broad programs that is, Community care, Child right promotion, Justice Administration. Under each program, we have various activities we that we undertake. When you take child’s right protection for instance, it entails registration of day care centers and then inspection. Then under it too, we also undertake inspection of residential home for children, what we normally

call children's home or orphanage homes. We also register cases in relation to child maintenance, welfare and other cases that relate to the general wellbeing of children. We also conduct public education and sensitization on the rights of the child in the communities within the municipality. Community care; it entails registration of non - governmental organizations and community based organization and as well as monitoring those organizations and also look at how NGOs can come together to assist communities to undertake development programs. Then we register persons with disabilities and link them to resources within the community such as registration of indigenes among them with the NHIS; and we also link them to the district assembly Common fund. We register them to access the fund. Then we also undertake monitoring to see whether those funds they have accessed they actually put them to better use. Then Justice Administration is concerned with children in conflict with the law and then when we go to the court room, we write social enquiry reports to the court and then we make our recommendations. Social enquiry report for couples that want access or custody; and then generally, I also sit as a panel member for the family tribunal and the Juvenal courts.

M: So tell us your role as a social welfare/ community development officer.

R: On the community development issues too, we also do organize empowerment programs for women who are not employed or those who are not in the formal sector and want to get something doing and we assist communities to carry out their self-help programs within their own communities.

M: In relation to children to children and adolescent, what role does your department plays?

R: Relation to children adolescents, last year we had an assistant from UNICEF and then we are embarking on child protection in our municipality, for now we have fifteen selected communities that we are engaged with. We have registered and trained community child protection committees within the various communities we working with. Then when we go there we normally use the kids that were provided by UNICEF to do our facilitation. We also organize symposiums for parents and adolescent. Normally we us the schools, before we started we had to write a letter to GES for permission to enter their schools. Anytime we go there they already know as. We normally meet the girls and talk to them especially about teenage pregnancy, drug abuse, and the rest.

M: Please tell us about some of the services you provide to parents, care givers and children in the communities.

R: Services we provide, for the parents, it's normally with non - maintenance of children. Usually, we have clients coming to our office to complain that their husbands or their ex-husbands have not been taking care of their responsibility as fathers. What we do is to issue an invitation letter and invite the two parties and then we sit and deliberate on the issues and then make appropriate recommendations as to the care of the children. And then when we are done with the recommendation we monitor to make sure that the parents are actually complying with the recommendation. Where they have failed to comply with the recommendation, we refer the case to the family tribunal and it will be addressed. And then for the caregivers, when we register parents with disabled children, we assist them, in terms of helping them to gain admission to the various special schools. we write letters and then we ask for them to be admitted, and when necessary, whenever we receive the common fund for PWDC, those parents with special children are given a priority, we give them assistance to be able to continue education of their children. Whenever we organize meetings, we send the information to those parents who are interested in getting something do to be part of those trainings, normally, we do it with (BAC) Business Advising Center. So they will come with all their support, we don't have the fund but rather the expertise in mobilizing the people and if possible start the business on their own.

M: You mentioned that, you give assistance to caregivers and children with disabilities, what type of assistance do you give?

R: Financial support, were the children cannot enroll and to start business so that he or she can support the child were the child is capable of going back to school or starting an apprenticeship.

M: Please tell us your experience working in the community as a social welfare.

R: What I can say is that, parents aren't doing bad but in some societies you still have some challenges, there's a saying that, “A child is to heard but not seen" sometimes children have problems, they then find it difficult in telling their parents but rather with their peers. It will interest you to know when some of these adolescent boys and girls come to our department, the things they can tell us but don't feel comfortable telling their parents and also through our conversation between parents and children. Let me give you an instance; we are monitoring the kids at Juvenal sector any time his sent he tries to find his way out back to the community and then when the child is back the parents will habour the child , they refuse to infirm the department the has come back. If the child goes to commit an offence the parents will be like this boy is giving us trouble. When we were interviewing the child, he told us that somebody in the community gave him an odd ring that whenever he is going to steal or rob he should put on the ring that

he will never be caught, this child has been wearing the ring but the parents hasn't bothered to ask their child where the ring is from. When the provision officer questioned he told us that it was a mallam who gave the ring to him......that whenever they go out to rob he will go and pay a percentage of the money to the mallam. So the kind of relationship between parents and children, others are cool but some isn't. So we suggest that parents should encourage good communication skills with their wards.

M: So what intervention do you have in parent child relationship that are inconsistent with child welfare.

R: For now at the municipal level, we have formed child protection committee in communities where the child is at. Where we don't have, we liaise with the family members. sometimes, we do sensitization programs like; going to churches, schools, women groups, and etc....through that, we're able to communicate social challenges as to what they are facing and we try to get their views.

M: Can you please relate this question with children with disabilities?

R: The Assembly made it a policy that anytime we receive the district assembly common fund, 10% of it should be allocated to the children in special schools and also this we are not in schools but hospital who need medical assistance, 10% should also be giving to them to support their medical bills and the rest is shared among the other groups.

M: So how do you accept the coordination on child and adolescent institutions?

R: At our level here, for instance, DOVVSU; they have been of great help to our department. Anytime time we call on them, they are ready to assist us. Last year we had one coordination program with them, and this year's coordination program we shared ideas and also try to look at each other’s short comings and see where we can help. The court has also helped the judiciary with child maintenance cases. When we refer it to the court, they just look at the recommendations and makes sure the person complies with it. As for social welfare, we give recommendations and the courts give orders and you know when the court gives order you need to go by it because if you fail to do so or flaunt it there are sanctions attached to it. The coordination is there, CHRAJ, they also have activities with them. Sometimes when cases come to our offices and see that the case will best be solved by CHRAJ or DOVVSU, we just need to refer those cases.

M: Apart from the referral, do you normally meet as a group?

R: As I earlier said, we have not had series of meetings, but last year we had one and other time "KMA" and called all the child related institutions to (RCC) and gave us a day's training. We were having an NGO working on child welfare; they usually have funds but our level here, we looking for funds to organize that training or coordinate meetings.

M: Could you please tell us something about the core issue on monitoring children in terms of compliance to national and international rules and regulations.

R: I'll start with social welfare, though we have all acts and laws governing an institution. Sometimes you have a limit where your power can reach. When one handles cases and you think as for this case you need to refer it, you don't need to keep it. With compliance, there are a lot of them but going forward to carry it out their implications looking at early child care, you go to a day care center and see the facilities are not appropriate in good conditions. And you will want to close it.......you as an individual cannot close down a day Care center because there are other institutions you need to consult. Before you will be able to close down a day care center, you need an order from the court.

M: What do you think are the courses?

R: Because we hardly interact with the institutions and there's also inadequate communication and coordination among this institutions which they are unable to carry out mandate.

M: please describe what makes children unsafe in this community.

R: Yes! For children, we have a lot of irresponsible parental behavior in the community which makes children unsafe. We have a child about 12 years to 13 years on the street at night selling, which they are exposed to a lot of dangers and also thus, children who have migrated from the northern part to stay here as migrant workers go into the bushes to and get paid in other to be able to fend for themselves. Most girls lack sexual education which makes them drop out of schools due to then not knowing the implications which affects them. Issues with child labour, where children are left to fend for themselves.

M: Do children with disabilities get unsafe in this community?

R: I can say yes to some extent, for our municipality, we have vibrant association for persons with disabilities and had several engagements with our parents. It's only few instances people do report that they have seen this (PWD) unsafe.

M: Tell me about your perspective for children in this district.

R: We don't have facilities except the community, normally bring that to them. What we do is to write referral letter where they have access to all these services. We don't have rehabilitation facilities for children.

M: What's your view on formal and informal child protection services in this community?

R: The formal protection, we have DOVVSU, CHRAJ, police, courts, etc.....all this services are there for the children to assess, but most of them don't even know this institutions so we have to get closer to them and for them to assess the services. Informal; churches, communities, some of them also refer cases that are beyond them to us and we also try to handle it when it’s beyond our standard, we also refer it up. Some of them have been trained to the extent, if when a child is in need of care; they know where to take the child to.

M: What type of cases do you use the formal and the informal system?

R: Such cases like rape, defilement, those cases shouldn't be handled as informal because they are laws and when a child is defiled, and thinks you can sin in your community and negotiated, and the cases will end which I don't think is the best. R: Cases which affects the emotions of children should be handled formally like; rape and defilement. For the informal, when someone beats up your child small, then you take it to the social welfare. You can handle it informally if it's not severe one. But where the right of the child is abused, you can use both the formal and the informal method of handling it.

M: Please tell us about cases that you used the formal system to resolve.

R: Okay! I'll talk about the case of child abuse assault of a 9 years old child who was a girl. She was brought from Mali to stay with a family member, that's, paternal grandfather. I was in the office one day and a call came from the FM station that a Child's right is being abused and the child has been locked in the room shouting for help. So the neighbors had to run to the scene, when they got there, the room was locked whiles the child was inside. This happened in an Islamic community. Together I, the police, and the media and the person who reported the cases quickly got to the scene and broke into the room. There they found the little girl with sores all over her body. We questioned her and she was like she was accused of stealing GHS60 which she knew nothing of. So her grandfather of whom she was staying with placed an iron into fire and used it on the girl.....So the court asked that her grandfather should be brought and the man was sentenced to 8months in jail. The courts stated that this man is off age that's; 65years and instead of him behaving like "God" by taking care of the child he is rather abusing

their rights. The courts then wrote a letter to the Mali embassy for the biological parents to come but the report

given was that, the biological father of the girl is mentally ill so the mother came and the court handed over their daughter to them and also the social welfare gave them the necessary documentation to sign and the girl was handed over back.

M: We want to know more about another case of which your outfit adopted more of the informal scheme.

R: Okay! A parent died leaving a four months old baby of which the baby was in the care of the deceased brother's wife that's, sister in-law. The woman died as a result if the treatment giving to her by her husband. The child became seriously ill and malnourished, everything was totally bad. The man then came to our office and said he was to take custody of the four months old baby. We referred the matter to the family heads of both clans to go and try to solve the issue surrounding the death of the woman and come. They came back and said the issue has been addressed. They then agreed that the man's mother should be given custody, who is about 50 years. so when they came they even came with her carrying the baby so we congratulated her agreeing on that decision so the baby is now in the care of the grandmother and we also intend to make follow up to see to it that is faring well.

M: Is there anything up want to add to what you have said so far?

R: Sometimes our challenges, we don't just handle the cases at the offices and leave it there, you need to follow up. If I need a vehicle or assistant of the police, I don't need to pay anything for their services.

M: Thank you very much for your time and conclusion.

M:

M: Can you please describe to us the general work of your institution

R: am here to make the 3 core programs of the department work and make an outcome of it in the district

M: if you say the 3 core program what are they?

R: The three core programs of the department are justice administration, community care, child rights promotion and protection. Under these they are broad areas that i make sure have to touch some specific areas and try to resolve issues around them

M: in relation to children and adolescent what roles does your institution play?

R: the department plays a key role in relation to children, the children's act look forward to the department to make the adolescent and children very comfortable in life so we take the children's amendments act as use it as our key paper or document for the children, so what the department does is to protect the rights and responsibilities of children

M: so when you are saying you use it as your key document to what extent do you use it as in for children protecting their rights

R: use it to what extend? Can you clarify it

M: as in using the children's act as a document

R: as pertaining to any conflict or in contact with the law that is our key documents so without it you would not know your right from your left what is says about the law pertaining to the punishment for an offender so that is what we use to speak on behalf of children to intervene on their behalf

M: can you please tell me about some of your services apart from child rights and protection

R: we do community Care, it involves every individual in this municipality, we also handle cases from the family settings, family welfare cases, we also try to arbitrate issues concerning family people who are in need of help and how to go about their daily activities responsibilities. People are becoming irresponsible so we make sure people come to their toes come back to their responsibilities

M: do you also offer violence protection against children services

R: yes we do child protection services that is why that we do teach or sensitize the youth or children on their rights and responsibilities so that is they are going wayward or going into conflict of the law so that social worker have to come in, the agency they have to come to and the channel they need to take that's what we do for violence against children

M: what about the rehabilitation of abused children?

R: hmmmm

M: madam why hmmm

R: you know there are a lot of things that goes into rehabilitating a person especially an abused person. When we find a client in this situation what we do or we will do

M: we want what you do

R: what we do is to make the client comfortable

M: what do you mean by making the person comfortable?

R: the person has been abused there are so many state that the person has gone to so you make the person comfortable like am there for you, not that I am a social worker, i can help you, we will help ourselves so from there the counselling begins in that there person comes from that state to another state and through the person's help we are able to come out of that state unless that case does not need a residential type of rehabilitation so can do both home and office counselling but if it need specific hands a specialist to handle the situation so we have to make a referral so imagine such a person who has been abused is the person in that capacity to go for the referral. When it outside my capacity i have to make a referral to the hospital, we know what is happening on the grounds when you go what happens what they seek for they cannot pay so what becomes of it. How can i rehabilitate that person What can i do to help such a person that person needs that help before i can rehabilitate and we have to so that before any medical help . I am not a medical officer when it gets to that stage it turns around

M: if i want to get to your service ends at where since you are not a technical person

R: it wouldn't end but if there is a need for a technical person, that person needs to act before we go to my own ways because a person has been raped what do i have to do i cannot determine if it is a yes or no for the rape i don't know how to determine that I have to do a referral for the person so that yes she has been confirmed that she has

been raped and semen was found in there so if am able to determine all these things then we will be able to go to the courts and seek justice there and we will see that we are all working on the ground and okay. But when we get there and we don't get anybody to do that then what do we do

M: so if you say you don't get anybody to do that are you saying

R: if it needs a specialist attention and we don't have it means if the client needs essential service that we don't have and if we have and they are not willing you can't force anybody to do that

M: i will ask you what you will do later but i want to know what you do about this service the sensitization to parents or caregivers of children with disabilities; it is part of your services

R: yes we go especially when we go to the field and we identify such children who have been kept in the room so we for see stigma issue so we try to talk to their parents and let them to know that they are worth it and so if they open up we make them know there are services that they also benefit from

M: can you tell me some of the services

R: we have social interventions like the LEAP, the PWP common fund if the person is a PWD and you think the person is a burden and we talk to you and you understand we secure through intervention for that person so at least we get you out of the situation that you think this one you have to keep it that way so that the person overcomes that

M: I want to know has the department identify these people

R: yes sometimes they come to us , sometimes there are private complains, sometimes our konkonsa on the field what they see and they approach; if the person is willing and accept us we talk about it

M: why are you saying if they are willing?

R: some of them don't accept the situation they find themselves, it not only with children some are grown ups and you identify that this person has a little this thing out there and its not working well we approach and ask them to come and we ask what can you do for yourself so we can do for them, we apply for the funds for them

M: as a social worker per your experience in this district (komenda, Agona and Abirim)what have you observed about how children and parents relates

R: in KEEA there is this issue I don't know how to tell it if it is parental irresponsibility most parents don't want to take up their responsibility; they don't understand parenting i have brought you to the world so we get cases at the child maintenance unit when we say take care of somebody it entails a lot care, i get 1 gh an di give it to you is is nit okay, no if you are taking care of somebody it entails a lot care, its not only money they don't understand that they don't get it if i have i will give i can't go and steal and they don't make the children comfortable in life

M: with your experience do you know why its happening

R: nobody can tell if its a mind set or moral issue i don't know

M: in relation to Elmina is it the same as you are saying

R: yes even the same in Agona, KEEA in all the zones in all the communities

M: So the apart from parents not understanding their wards what again have you observed with regards to parenting

R: it just their responsibility, they don't want to be responsible they hate being responsible

M: so what interventions do you have to streamline these parent and child and child relationship and child welfare and violence

R: for now the social protection program it is what we are using and every where we are talking about it, we are engaging families

M: we are talking about interventions

R: UNICEF has a social protection program that is on board and that's what we are using. We are hoping parents get the idea of protecting their children and also being responsible. So everywhere we go and every chance we have we educated them on these activities we use our tool box things to teach them things they are supposed to do what they need to do for their children so live will be meaningful to them

M: what about interventions for children with disabilities in relation to parent and child relationship

R: for that aspect the only intervention is the common fund, in this town there some NGOs are also helping, some not basically going for PWDs but when they across person with disabilities and they feel they can help they come together we work

M: has it happen in this district

R: there was one they donated a wheel chair for the person so at least the person is safe from the environment so that we can do another thing for another person

M: will you say that with the common fund are all the disability people on it

R: yes

M: really you can say that

R: yes, every

M: so how is it done, is it annually the benefit or

R: it comes quarterly so as and when it comes we get our 3%. When it comes 3% is deducted into a separate account, there is a guideline on how to use. This time is for education, assisted devices, medical support and income generating activities so the guideline comes with percentages to all these things per application we need to vet each application there is a committee that sit on issues to be decided that this is the amount that came so with income generating activities we should give 10% of this amount, education % and so on then we select decide how many people we should take then we call them for one on one vetting so that whatever we have written on your application you defend it. At first they come for the money and go, the nest time they are here and they don’t make any meaningful use of the money well so when you come and you don’t use it for any income generating activity we will charge you. They can tell us the income generating activity they want to venture into it does not mean the care giver should operate the business but you the one with a disability should operate the business. So you need to defend it like I am a blind person but i want to learn soap making so how can a blind person learn soap making if you are able to defend it then we say okay where do you want to learn it, who is your craft master and what things do you need to learn it and we pay for that

M: so what about children who are not old and their care giver take care of them

R: that is our problem, most of the time the care givers want most of the money to take care of them. The law says that the help should go to that person with disability

M: the person is a child

R: so if you are a child, Most of the time children with disability are rather seeking helps from the hospitals. So what we usually advice for such children they should seek help from the hospital rather than an income generating activity there is no way the department we are going to buy a corn mill machine for the child to operate

M: **so with mostly with parents with children with disability it's the medical help that will do**

R: yes most of the time, some of them are on drugs right from birth that they bring the bills

M: **so from last quarter were they given**

R: yes, they did, they brought the medical bills

M: **okay so how can you assess the coordination that exit among the adolescent related issues example like the institutions with gender, with Ghana health, with ministry of education. What is the coordination like?**

R: with my experience i will say these stakeholders are doing a great work; their coordination with adolescent issues is great; every time they are on their toes. Social welfare together with the ministry of health do their daily at first with dialy now weekly go to the community

M: **why now weekly**

R: they go together with them. With our big tool box when they are doing their grandma's corner they have their corner when they do their adolescent discussion after that we also chip in with their rights and responsibilities and gender is also there to do their safe sex so we always collaborate. With the adolescent at KEEA if they are listening to us and working towards what we are telling them they will have any problem

M: **what about when it come to information sharing with other stakeholders, how is it like**

R: we do share information very well when the ministry of health encounters a problem even before the problem they will call you, when we went out we met this child we need you . We do share information, when gender is going he has go with me because the multi questions they ask she can't answer and so most of the time gender social welfare health they move together

M: **do you have a defined role when you go**

R: yes

M: **so when it comes to data collection, the quality of data collection reporting how is it done among stakeholders**

R: as for data collection i don't know how to answer this, because most of the time we rely on the incomes here going on the field say we are doing data collection we have not

M: with other stakeholders with reporting how is like

R: whatever i need from gender i will ask from gender and they will give it to me before I write mine

M: can you tell me some of the core issues and monitoring and supervisory child related organisations in terms of compliance; national and international regulations

R: what will i say the issues are many?

M: then let's take the national first then we move to the international

R: you want me to say it you already know, here for instance when we go to the field, it always difficult to meet the expectations of our client or target group on time we always come with a huge car with our things so when you ask them to meet you at 2 they come there at 8 or even 6 because they have high expectations from you. When you are engaging at least adolescent there should be a little refreshment that even attracts them, the meeting goes dry like that and the meeting has to be interactive, so when we always ask for is financial assistance, the NGOs are also doing their own, UNICEF is also doing their own, the social protection are also teaching us but what about the rest

M: what about organisations that are child related in KKEA, how do you do monitoring and supervising these organisations to meet national compliance and what are the issues with monitoring

R: now the national has developed some SOPs we have started using it so that what this quarter we used it, but monitoring it that what am saying financial issues, when am going for monitoring its the LEAP motor bikes that is there me i cannot seat on motor bikes. When i came here at first i was using motor bikes and i went for LEAP monitoring my head ached me for a week so i told myself not to seat on the motor bike again and some of the communities are very far so if we have to go 3 times in a quarter i prefer going ones the i call for the vehicle and it sends me there; when you go for monitoring you spend the whole day or two so financially we are handicapped the assembly don't have much vehicles if you want to go and somebody want to use it what do you do. We also rely on them through phone calls

M: the organisations

R: yes, when you need something i have to call them before going and because of the bad network we cannot go. I can't also rely only on phone calls sometimes they also have complains through that we are able to identify what is happening they are going through and we have to go and take the lead

M: so please can you describe what make children unsafe in this district (elimina, agona abirim Or KEEA as a whole)?

R: there are so many unsafe things in this community, i don't know what will make it unsafe if it is a behaviour that they have acquired and they want to leave it. In Elmina per se the area that the overhead is that part is a doom area

M: what do you mean by a doom area

R: there are adolescent or children who are engaging in a lot of social vices like prostitution (why am i forgetting the name of the area) and every social intervention you take there they don't really participate. Now the international aids is helping us to let them know about safe sex, Anyisa that is the name in Elmina you will go and meet 4 people sometimes 6 and imagine you moving there and meeting just 6 people meanwhile there are so many of them moving about. You talk to them they will not mind you they want to know what you brought to them

M: that's the children?

R: yes, she does the job to take her money, so now that you have come empty handed they will not mind you

M: so what account for this problem?

R: It's irresponsibility on the part of the parent, they have just left them on their own , they won't take care of your educational needs, so when they also go to find something for themselves their parents cannot say anything, they will not say anything

M: what about the economic factor here

R: it could be but is that really an issue, if you have the economic means and you don't still protect your child, i will go you know the adolescent stage is very crucial everybody wants freedom and if you don't camp me and i go and i see it s nice and you don't stop me i will go

M: with your years experience in KEEA will you say it might be some social or cultural factors making children unsafe

R : in KEEA its a mixed something there are times some parents will tell you giving birth is a nice thing its expands their family and they boast of their number of children and that makes them happy there is this cultural something that when you are in your teens and you have not yet given birth they call you "saadwi" so when you are talking you are useless. So it is a mixed factors coming in play here. They will say its money if you give them capital for a business they don't know what to do with it some can do something meaningful with it so i don't even know what is wrong with them i don't trust them

M: why are you saying so?

R: They are just too lazy for me. If you are a mother there are so many opportunities you can seize, there so many things other women have done to train their children to become what they are now; people are selling water they will not do it they prefer laying down for sex and they will be given either 5 or 10 cedis as payment

M: so children with disability do they feel safe in these communities

R: i will say yes because we have not experience any abuse against them or there have not been any complains

M: what can you tell me about in your perspective rehabilitation services in this district/municipal or central regions as a whole and Ghana?

R: in all the regions we lack rehabilitation services because my understanding of rehabilitation it means people who need special assistance apart from giving the assistance that will help the person come out of their situation a traumatic situation the person might be in, where is the special assistance I can give where are the centres in KEEA we don't have.

M: here in KEEA you don't have?

R: MAOBAB is doing some sort of rehabilitation for cerebral palsy

M: What is cerebral palsy, are they here

R: yes it is a medical condition and autistic children but it's not enough

M: so who is taking care of these children

R: an institution called Paaldre pui, they admit them and give them a special care, they take care of them

M: what about other children like abused children, what services do you have for them?

R: as am saying if i do my aspect what’s next, if you need special attention there is non in KEEA so where do i make the referral to. In central region Sometime we refer to mephiboseth or secondi that all so if we have

M: so if you have a child like that its only secondi you have to take the child to

R: yes a child in that situation needs a special school its either mephiboseth or secondi

M: so its either abused children, rape cases or these things that you feel you have to take them out for a while, is there any care home or what

R: yes we use the residential homes too, we take them there for some period until we see that the person is safe or has come out of that situation and we continue to see if we can reunite to the same family if there is a nearest family member that will accept

M: with the rehabilitation services do they get asses to therapist

R: no, apart from me the social worker doing my aspect using my counselling skills, if you need a special therapist how do i call some of them even if someone needs a therapist how can i assess them

M: there are non in KEEA

R: Yes, unless I go to UCC and find one psychologist

M: what are you views on formal and informal child protection agencies available in these communities?

R: if you say my view I don’t understand

M: what I mean is that what you have to say when it comes to the formal and the informal child protection agencies available in KEEA

R: Which one if the formal and which one is the informal

M: you tell me which one you classify formal and which one informal

R: my aspect is formal and the other stakeholders own is the informal that what i think

M: so what is your view when it comes informal everything minus you and the stakeholders, that is settling child protection issues for parents and children

R: that’s what i am saying we are trying to make it relevant to our various community members or leadership for our daily sensitization programmes we involve them so there are certain things you need to do so that the children in this community can say that yes

we can confide in this leader when we have this issue some are backing up but there are some communities the child protection channels are working so there is no need to come to social welfare to make a case and write a letter most of the time they try to deliberate on the issues between the children and their parents

M: **let say the case with Elmina, Agona and Abirim is the informal setting working**

R: Elimina,its working to some extent, we have an assembly member who is always on his toes some of the time he joins in and at times he says he want this case settled at home or make sure i do this what we feel is not an abusive case that he can settle we allow him to settle it and he gives us the feedback. So when he accepts it we monitor him

M: **with the ones you allow, what are some of the cases you allow**

R: it basically child maintenance

M: **so which ones will you prefer the formal to handle and which one will you prefer the informal to handle**

R: when we come in like this there are certain clients that are remorseful if they try to understand why i should be there for my child fine but if they don't we still have to come in and use force and that we are not supposed to do but some you are compelled to do it we use the family tribunal system to handle these cases to give a little shake to make them comply. It all depends on the client there are some who does not want to listen to what you are saying what they have planned to do that's what they want to do with such people you will have to allow the system to take its place we cannot allow the community to settle such cases you will use the courts

M: **if I want to understand you all cases has to first has to report to the formal**

R: No, it's not about the case but about the client

M: **not the case but the client?**

R: yes, you get some case from a client and per our talks and deliberations and showed that am sorry am remorseful am not supposed to treat my child that way then we leave it that way. There are times we don't need to take child maintenance fees here they do that at home. We want to make the family system work you can't always take money by bankers We always talk to parents that because they are divorced or are not staying together do not mean they should not talk to each other they should live in harmony. So we see clients that are remorseful we want to do the maintenance fee paying at home

at times the family again sit down and agree on the maintenance fee and mode of collection and both parties agree and it goes well with them

M: you please describe to me a case that you use the formal system that is us you resolved

R: that will not happen because am not supposed to review any case that i handled with anybody except from a highest body

M: not details, not a name just what happened and the out come

R: no, confidentiality, it is a case

M: we want to know a case you adopt the informal scheme to resolve

R: i have already given an example cases that are within like the assembly man case when he comes and they fall within his zone when he come in he is given to settle

M: cases like

R: eeeiii , hahahahahaha all about child maintenance cases

M: child maintenance means

R: the need to give their child upkeep money, pay school fees, buys books.

M: what about teenage mothers not having the fathers to take care of them which system take them on is it the formal or the informal

R: when they come to complain we take it as formal so we give out invitation letter through the various assembly members, unit committee members so that the client won't go if there is an issue with the respondent there can be some violence because of the letter given to him personally so we use the assembly members. So the letter gets to them and someone come that this girl is my niece or this boy is my nephew i want to resolve this issue, i will say no this girl is a teenager or i will say am giving you 2 weeks to resolve this issue and come and give us a feedback. If we feel it's okay we allow them and we accept whatever recommendations they bring but when we feel there is cheating somewhere we can say lets add this or you have left this out lets go back and do it this way. So if the outcome is fine then the social welfare does not have any pending case with that client it has been resolved at the informal system

M: what will you say this is a good outcome?

R: when the respondent has a agreed on all his responsibilities the most basic ones, the we can say it has become a good outcome rather than not doing anything

M: thank you very much we are done, is there anything you are doing with regards to social welfare when it comes to KEEA that you have not mentioned that you are doing

R: Basically you have channelled all your questions to what we do and what we are on the grounds doing is basically basically child protection issues and i cannot recollect anything that you have not asked

M: thank you very much.

**District Name**	
**Community Name**	
**Date of interview**	
**Interviewer name**	
**Transcriber's name**	
**Interview length**	57 minutes, 3 seconds and 1 minute, 11 seconds

M:

M: Thank you very much sir.

R: You are welcome.

M: For the opportunity offered.

R: Hmmm. Ok.

M: Please can you describe to me the general work of your institution?

R:

M: Ok.

R: Erheh. And then what we also have with the, that is juvenile right promotion and the rest. Aha, this so basically these are what we do, but there are activities under what I have mentioned. Because these are goals but we sub activities under the goals I've just mentioned.

M: Can you just may be, each of the goals you tell me one activity?

R: Aha, so for instance if you look at like I said we justice administration, child right promotion and protection and community care.

M: Hmm.

R: So under it we have justice administration, we have what we call social enquiry record report. And 2 we have juvenile family tribunal. And 3 we have probation committee meetings and prison after case services.

M: Ok.

R: And then…

M: You can move on to the next one.

R: The next goal that is child right promotion and protection. Under it we have child survival and development. And then we have children in need of care and protection, yes so when we come to the other one, the community care. Under the activities of the community care, we have the registration of PWDs.

M: What is PWD?

R: It is Persons With Disabilities.

M: Ok.

R: And then we have the hospital welfare. That has to do with; in the hospital setting, we have the office one, the registration office. Where we handle patients like the poor, or maybe we can recommend to the particular authority, that this person having looked at the family background and he is not capable to pay for her drugs and other things and so we will have to write to waive off the bills.

M: Hmmn.

R: Yes. And also we as conduced, those who are treated and abscond from the hospital we retrace up and get them and back to our office.

M: **Ok.**

R: So when we come to running the rehabilitation centres, persons with disabilities we have an institution called the REHAP centre, at Nyoni. So we bring them to attend school and they will learn some trade of some kind are being taught over there so that they will not depend on the street.

M: **Ok.**

R: They will also have something to live on.

M: **Nyoni.**

R: Yes.

M: **Is Nyoni in this Kpandai District?**

R: Nyoni is in Tamale.

M: **Ok.**

R: Tamale.

M: **So if you get someone with disability, you prepare the person and…**

R: And send the person there. Yes to learn the trade of any kind. Then we have the registration of NGOs.

M: **Hmmn.**

R: Aha, yes, Non-Governmental Organisations. And LEAP programme, LEAP programme is also targeted. So these are all the activities.

M: **All the activities under community care.**

R: Yes, under community care.

M: **Ok, thank you very much. My next question is I want to find out your role, your role in this institution.**

R: Erh, my role vis-à-vis what I have mentioned, some of the activities I carried out. And then if you look at the Justice Administration as the first this thing. With children's right and then like family welfare. Aha, for instance, these are some cases, somebody has reported that the husband has neglected them and the feeding and that kind of thing. And so normally we invite the respondent. Aha, then you sit down and put things together. This

is what you have said, let's see, see through and see how you can maintain your children and bring sanity to the house. And also domestic violence, some vice versa, is it here that we don't see women fighting or boxing with their husband. But in the southern sector, it is prevalent. So men will beat their children, eh their wives and sometimes they don't go to the police they bring it here. And through counselling we try to mediate and then bring them together. But once it gets to the police you effect the arrest and that will jeopardize the case.

M: Ok, in relation to children and adolescent what role does your department play?

R: Erh, the role our department play, sometimes we go in to give some talks. You understand that, like schools, what do you call it the mosques, the churches. Aha, to see to maybe issues of you know female like adolescent and that kind of thing. We give talks to them, as to what they are supposed to do. So that, seeing them does not, for instance if you look at female aspect in this setting, they don't even look at them to be. Maybe if you even go to highest school, maybe you may definitely end up in the kitchen. You try to explain to them that yes they also have a role to play in the society. So they should also give them the equal rights.

M: Hmm.

R: Erheh. Yes.

M: Apart from that, what other things do you do in relation to children and adolescents?

R: Yes in relation to children, like for instance this LEAP has come; it means; so LEAP is targeting the poorest of the poor. You see vis-à-vis the children and that goes to help the children. Because we have the social network where school feeding programme comes into play, we have the National Health Insurance, they have been registered by these people of which the children are targeted.

M: Hmmm.

R: Aha, so every 2 months they have a LEAP grant to help themselves. And then, they can go to hospital and they can take care of the children welfare. So these are some of the issues that we do.

M: Ok.

R: Yes.

M: So I want you to tell me about some of the services you provide to parents…

R: Yeah, the services we…

M: Caregivers or children.

R: Ok, Caregivers.

M: So you can take one, let's take parents first.

R: Ok.

M: What are the services you provide for the parents?

R: Services we provide to parents like may be parents will come to report that maybe they have a child and she is going wayward. And so they come to seek our consent, what do we do? Because we need to, so we just advise the person and then bring the person, we will counsel and try to: Because there are able to leave them to us as professionals dealing with children, because they normally beat them to change. But the normal thing is change, you know, you can't beat the child to change. Aha, we need to sit down to dialogue. And so these are some of the services we gave to them and did work.

M: What about caregivers?

R: Yeah, the caregivers too we talk to them as taking care of a child. Even though some of them too it won't be their biological children. Aha, because they take care of their siblings and that kind of thing. Or somebody being hired into the house as a caregiver to take care for may be a working mother. Aha so they should see them as their own biological children and not to maltreat them. Hmmn.

M: Then to children.

R: Children too we said they should honour and respect their parents.

M: Hmmn.

R: Aha, if they honour and respect their parents I think they will go far. Yes.

M: Ok. Do you deal with return-to-school counselling?

R: Yes because some of the, especially if you look at the female aspect some of them under age of 14 to 15 who are being pregnant. And so there are likelihoods that they assume that their education is spoilt forever. So we try to counsel them that may be after the delivery you can still go back to school. So some impact have been made, some of them they've been able to go to school, some of them too they've become child mothers.

M: Ok.

R: Yes.

M: What about rehabilitation of abused children?

R: Yes rehabilitation of abused children, erh sometimes erh we try to relate it to involve, in our setting we involve, when things are not getting well we involve possible the police. And then for instance, as I talk to you there is one child who has been abused by the parents. And as a result she goes out in the night and come back day. And so she has always been beaten. And then I try to meet them and talk to them and also talk the child to tell her parents what she wants. She has been sent to school, but she doesn't go to school, but she sleeps outside sleep and comes. So these are some of the things we talk to them and advise them. Erheh, and use a role model for them to see that I can also be so-so and so.

M: Ok.

R: Yeah.

M: Ok, and counselling of violent parents, Prosecution of offending children and parents.

R: Erheh, counselling of violent parents, it is something that we try to bring them together. Because sometimes, if you want to use the legal framework, you know, they themselves will say no we don't want it. We want it to be limited here. Because sometimes I will use an example, there is one woman here who was assaulted by the husband. He used this animal skin, he used as cane on the back of the woman. And we see the scars, I called the husband, you know the woman even want me to forward the case. So I called the police, but when it was cause for arresting and the man to be put behind bars, the woman said no, no. She doesn't want it to go far. You see. She just wanted to talk to the husband and then things end. So we just, you know some of this case, we need to use the dialogue.

M: Ok.

R: Hmmn.

M: Do you also handle stigmatization issues, sorry do you handle cases that involve stigmatization of children with disabilities?

R: Erh, Here what is happening is that in this setting; when they have a child with the disability they don't come out until then; they don't try to bring the person out, they want to hide the person. Because if you look at the field setting they think is indictment on the family. This family has brought out this person that he doesn't fit to be in the house.

Sometimes they prefer even going to leave the person in the bush by using jujuman to kill. You understand that. So these are some of the issues, so we try to talk to them and use role models who are disables. So now a reform, they are being reformed so they are not even employing them into the social setting in schools where we have these petty, petty teachers teaching them and other things. So they are now getting.

M: Based on your experience, working in this community can you tell me about your observations on how parents' child relationship is?

R: Yeah, here parents child relationship erh….

M: Or parents adult.

R: Ok. Hmmn.

M: Parents or an adult relationship is like, with a child.

R: Here there are, let me use this setting, because here the adults seem to know themselves and leave the children. Because the child can walk in the evening till day break and the biological parents they don't care. You see. But meanwhile their animals they see to drive their animals into the pen, and leaving children. You can even in the night you can come here and take a research; you can see, they can walk in the evening meanwhile, they have homes. They will walk till daybreak; they will not go to sleep.

M: Is it that they don't have a place to sleep?

R: They have a place to sleep. They have a place to sleep but because of the parent-children relationship is not there. They don't see it, once they bring out children, the children should fend for themselves. You see, and that is why some of them who are a bit educated, they normally report their parents here for child neglect, and then we try to mediate and then we get things worked out. That is the situation here.

M: Ok, what interventions do you have in place to streamline parents or guardian-child relationship to be in consonant with the child's welfare?

R: Yeah we try to organise erh. You know these children; they form clubs some of them have sitting places. They normally mount some tables or some chairs and they sit on it; they call themselves what boys, what boys. Aha! You see we all go there to meet them and then to counsel them; there was a day if I organise a meeting and invited them, we just mount the stage and say ok the government a stake to help them provided they want to find a profession for themselves. Aha and some of them said in fact they will want to do that. But they are looking up to we even made a proposal to my regional office to see

whether they can get some source of funding to come and then we can help them and give them some trade that could benefit them. Aha.

M: **Yeah. And what intervention do you have for parents or adults who have children with disability?**

R: Yes, there is what we called the disability fund. It is some 2% or some 3% is being put aside; it is part of the common fund. The district assembly common fund, that is being used for persons with disability who have applied. The issue, the bottom line you apply for the disability fund, to do one A, B, C. You specify what you want to do so the assembly will meet. There is some criteria you meet, then we approve then we grant you. Last year some said they wanted deep freezers; they wanted animals so the community sat and the assembly provided. So TV3 came and even took care of it. So that one is not far, it is there but provided you access. You apply because you are supposed to apply for it.

M: **Ok.**

R: Erheh, so it has been catered for.

M: **How do you assess the coordination between child and adolescent institution, like yours?**

R: Uhmm.

M: **Department of Social, Ministry of Gender and Child Protection…**

R: Hmm.

M: **Division of Child Health Service, Domestic Violence and Victim Support Unit of the Ghana Police Service, the Ministry of Education, etc. How do you work together?**

R: Ok.

M: **All these institutions, how do work together?**

R: These institutions.

M: **How do work together on child and adolescent issues (said together).**

R: Like I said in the hospital, when I mentioned hospital welfare. The station worker at the, we call it the social medical worker at the medical centre what do you call it, work with the medical team. And so while the medical doctor assesses the medical condition, then the station worker also provide the psychological healing to that patient.

M: Hmmn

R: Erheh. Because you can do all sort of giving drugs, giving drugs; the person is not cured. And the social worker will now also use the psychological this thing, to give social therapy to help the person. And so that is the medical setting, the hospital setting. And then if you look at the police. We work with the police while in the paternity case, somebody impregnate the child and when they bring it we look at it, and the respondent is proving arrogant and that kind of thing we involve the police. Because they have the power to arrest, we involve the police to come and take the person and go and put him behind bars. And they see down over there to see if the person is responsible, I will do ABC to take care of the pregnancy. And they come back to this institution, and then we make the person to write memorandum of understanding and write, and then to comply. Aha, that is the action with the police.

M: If I give the opportunity to mark or to grade the coordination between these institutions; what will you say?

R: Oh.

M: How will you assess them?

R: Our coordination, our coordination with them, I will say it is 80%. Because that even encompasses the court. Because where we normally send cases to the court, family welfare, as I was mentioning for them to look at it and then probation. Put things to probation and they will enforce that, that do ABC. Because we don't have the power to enforce that, we work with other networks; we network with other people to enforce that. Like I said the court is also one of the cases.

M: Hmmm.

R: So our coordination with them is quite that.

M: Hmmm.

R: Yes.

M: Ok, so can you describe the frequencies or schedules of meetings you have with such institutions?

R: Yes meetings, what we have like in the LEAP. I was talking about the LEAP; we have the district LEAP Implementation Committee. That is other sectors, like Ghana Health Service, National Health Insurance, GES: all the sectors, all part of it. So we all do meetings and discuss issues of concern and the development of the people here.

M: **Hmm.**

R: So we are meeting. And sometimes they also call us and we meet even here and the regional level. Yesa.

M: **So how will assess the frequency of such meetings?**

R: Erh the frequency, well erh may be like in a quarter. May be we could meet twice. Aha, we could meet twice or once. Aha so that is basically with the frequencies. Hmm.

M: **How do you asses the quality of the data connection oh sorry, collection and then reporting?**

R: Erh, data collection, er on what?

M: **The work you do together…**

R: The work.

M: **And other organisation…**

R: Erh.

M: **Do you collect data?**

R: Yeah. We…

M: **On your mandate.**

R: We.

M: **Do you collect data?**

R: Yes, we. Like disability this thing, we have data on disability, then LEAP we have data on LEAP.

M: **Hmmm.**

R: Erheh, so it is various sectors that have the data.

M: **Erheh, the data, is there a way you utilize data from those other institutions or you formulate information from them?**

R: For instance, when we have assembly meeting

M: **Hmmm.**

R: Or er erh a review of the assembly meeting…

M: Hmmm.

R: So, sectors come with their reports, sometimes is a projection. They will put on a projector, powerpoint and we all see. And then the assembly will coordinate and will put it in a file and then they can also store it. They have a database, so that they can retrieve when somebody comes to fetch for information. Aha. But apart from that each department have their own data.

M: Hmmm.

R: Erheh. We have the data.

M: Ok.

R: Hmmm.

M: Can you please tell me something about your core issues…

R: Hmmm.

M: In your monitoring and supervision of children related organisations.

R: Hmmm.

M: In terms of compliance to the national rules and regulations.

R: Erh in terms of compliance, for instance let's use a case study of like child maintenance.

M: Hmmm.

R: If someone has reported that they are being neglected or the children are being neglected by their father, then we will talk to you, you agree. And then we write the memorandum of understanding for you to sign. And then frankly I will send a remittance of this until you comply, and so that is where we will use the law to make you respond to what you have done. Aha because you see it and then you sign. Aha.

M: Apart from this issue, in doing your monitoring and supervision did you find an issue the so called that is major? That you use the law or you make the people to comply with it as you have just said.

R: For instance, erh we normally called if you look at like social protection that LEAP committees are and the RING are resilient in Northern Ghana. We collect data from as they pay them LEAP what did they use the money for? And then, there like each

beneficiary is supposed to register under health service freely, I think. But the source, anytime they go to the hospital using National Health Insurance, to access medical care, they are paid and they make the patients to pay.

M: Hmmm.

R: Erheh, Even though they have the Insurance Card. They make them to pay.

M: Hmmm.

R: Aha. So that is where that challenge, from the communities, they have been made to pay. We are told that when you have the card you don't pay because the government pay. But anytime they go to the hospital setting, they say they don't have so they have to go to outside and pay. So sometimes these are the issues that come up.

M: Sorry.

R: So we try to meet the network I was talking about. The hospital, the Social Welfare, the hospital setting and say ok, these are the responses we get from the community. And so what do they say to it.

M: Hmmm.

R: So, each sector will be invited to hear the response of the community. And so we invite them and project. And so the community members themselves will be part of the meeting and say yes oh this is what is coming to us. What do you have to say, because that is your sector? And so the sector will respond to the issues.

M: Ok.

R: Yes.

M: Can you please describe to me, what makes children unsafe in this community?

R: Huh, things that make children unsafe here, huh, it is they adopt erh this drug they call, what is being used to, a pain killer…

M: Hmmm.

R: But they abuse it.

M: This drug erh…

R: Tramadol (said together). It's been used as a painkiller but here, they misuse it and they overtake doses and excess. They mix it with other energy drinks, and then when they take it those, they do what is not expected.

M: Hmmm.

R: They go to loot shops; they do what is not just socially accepted.

M: Hmmm.

R: Aha, so that is what is making the people here.

M: Unsafe.

R: Yes unsafe.

M: Apart from this, are there any other things?

R: Apart…

M: Making children unsafe.

R: Hmmm.

M: From 0-19 years.

R: Erh, erh and then they also go through pilfering and that kind of thing. If you sleep in the night they will go and break your kiosks and they still (said together). Aha, you see so sometimes these are what is happening here.

M: Are there issues related to the economy or economic factors that make children unsafe?

R: Here this is a farming district, they all farm as their economic this thing, and so to improve their economy or their livelihood. Aha so if you look at those within, they all farm some of them farm, majority of them farm. But others will not farm, they just want to go and you see. Even if you see a child of 15 will have a motor bike and…

M: Ok.

R: Aha. Some of them will just want to live a luxurious life but not farming-Going to pick people's things.

M: Hmmm.

R: Aha.

M: Are there norms or cultural practices that make the child unsafe?

R: If you look at the female setting, here if you look at the female setting we have what we call, especially in the Komkomba tradition; what we call the barter trade where we practice. So a child may be about 15 in the primary school and then they will bring the child out and exchange for marriage. You understand that, that is sometimes make the child unsafe because the child may want to pursue academics and you take and exchange for marriage. Sometimes they take the child to Togo and that kind of thing. So I have been meeting with the assembly to meet and tell them this is not to practise. So I'm sure now the ladder is declining, because the message has gone down, it is declining.

M: Hmmm.

R: Aside from that, this is where they are unsafe. Hmmm.

M: Ok.

R: This side.

M: Thank you. Any other?

R: Hmmm.

M: Apart from that are there any other cultural practices like in the south puberty rites…

R: Here they don't have it, is just the exchange for marriage.

M: Exchange for marriage, hmmm.

R: Erheh, Exchange or child mothers or early marriages.

M: Ok.

R: Aha, Early marriages.

M: Hmmm.

R: Hmmm.

M: And how does that affect them, the child and makes the child unsafe?

R: Yes, you see at the age of 16 or 15 they, let me say the child did not develop and adult mind. And with that if she is married she will be having and adult thinking meanwhile she is not an adult.

M: **Hmmm.**

R: It means you make the child to think so much and will have an adverse effect on the person.

M: **Hmmm.**

R: Aha. That she will be thinking, as an adult like 40 or 60 will be sleeping with 16 years old girl.

M: **Hmmm.**

R: And you know this thing, definitely it is not done.

M: **Hmmm.**

R: Aha, you see. It will have a bad effect on the child.

M: **Ok. Do children with disability feel unsafe in this community?**

R: Like I was saying in my earliest submission, I mentioned that.

M: **Some.**

R: Yes some of them leave them in the bush, erhe they will go and call a specialist. And we say erhe go in the bush. Literally means, he will go and kill the person.

M: **Hmmm.**

R: Because that family setting will not want to have a disability. Because if they have a disabled, there is a saying, there is a curse in that family. That is what they explain here.

M: **Hmmm.**

R: Aha, so that makes them astray.

M: **Ok. But those who are fortunate and they don't go to leave them in the bush and they grow in the society.**

R: Yes they grow in the society.

M: **Are they stigmatized, are they safe, apart from the instance that you have mentioned that they…**

R: Yeah.

M: Some at infancy they throw them away, they kill them.

R: Hmmm.

M: Those who survive…

R: Hmmm.

M: Is there a way that; are they unsafe in any way?

R: Erh I think the stigmatization of the person with disability is there.

M: Hmmm.

R: Yes, because some of them they just look at you and say; you a disabled. You see.

M: Hmmm.

R: So if they say, you a disabled; is not by your likeness you came in that form.

M: Like they look down on you.

R: Aha, they look down on them. Aha, you see they look down on them. So these are some of the issues.

M: Hmmm. Ok.

R: Erheh.

M: Tell me about…Erheh tell me about some of the reasons why the disable or those with disability are or they feel unsafe?

R: Hmmm.

M: In this community.

R: Erh, they feel unsafe because their voices have been heard.

M: Hmmm.

R: Even though they have a group, some of them formed a group, Association of Persons with Disability.

M: Hmmm.

R: Aha. Some of them their voices are not been heard. Even if you look at the social setting, they who are supposed to aspire to higher levels, how many of them are employed? If

you look at the statistics, the regional and the national, how many of them are employed? So certainly if you look at somebody will tell you before travellers come to Ghana, they spoil before they come here.

M: Aah, the Chamber pot.

R: Yes, yes, the Chamber pot. Aah (all laughing). So you see the issue. So the international level, that is why there is this formal gender minister, this Djaba, yes…

M: Otiko Djaba.

R: Yes. Djaba.

M: Currently.

R: Yes. Erheh. The foundation…

M: The formal one was Nana Oye Lithur.

R: Aha, any of any of, Otiko Djaba is not there again.

M: Aah!!

R: She is just resigned.

M: Aah! Ok.

R: And somebody took over.

M: Ok.

R: So we are now currently on disability with the forms and national; they try to make their voices heard.

M: Ok.

R: Since, we now champion the disability course. Aha so now they will make view of it.

M: Hmmm. Tell me your perspective about rehabilitation services for children in this district.

R: Yeah person with disability…

M: Or region or country.

R: Erh erh Like I said for, if you take the national level per se. Person with disability even those who have gone to university, in the social setting the employment the figures are nothing to write home about. If you come to the regional level, how many of them is; even if you look at the social structure, the infrastructure, you see storey buildings how many of them. Because you can just see the indication here…

M: Hmmm.

R: They have put up the structure which is a storey; it means disables are being discriminated against- Because he or she cannot access the director, because the director is sitting at the top.

M: Hmmm.

R: So you can look at this is the national perspective; they have been discriminated upon. What about the regionals; so it is now that persons with disabilities, they have now articulated their voice, for government to see that now any social infrastructure that they put up. They should make provision for disability fending. So that when they come they can also access to see the big man.

M: Hmmm.

R: Aha.

M: Ok.

R: So now new structures that are springing up, disability, they are being disability friendly. So they can go with…

M: The perspective I was talking about is the rehabilitation services for children.

R: For children, Persons with disability.

M: No; for children in general…

R: In general.

M: Or, broadening?

R: Oh ok.

M: In this district…

R: Hmmm.

M: this region…

R: Hmmm.

M: and the country.

R: Hmmm, ok.

M: From your opinion do you think there is rehabilitation for these children, 0-19 years in our nation?

R: Erh…

M: Right from the district level.

R: Level, erh.

M: You can talk about the district level first.

R: Hmmm.

M: And when we finish, you talk about the region and then the nation.

R: Hmmm. Ok.

M: Also.

R: Yes like I was saying, here. Like first submission I was saying that the children are left here. They are supposed to be catered for by their parents. Or make sure that they protect and promote their living. But it looks as if it's vice versa; it is rather the goats, the animals…

M: They remember.

R: Yes, and leave their children to fend for themselves.

M: So the institutions that are here…

R: Hmmm.

M: Do they have a rehabilitation service?

R: Hmmm. Yeah.

M: For these children?

R: Erh.

M: **In this district.**

R: In this district, we don't have but what we do is that, some of them who have the consent, may be they neglect their parents, their children. And some of them find their way here. Some of the time we use the media, the fm.

M: **Hmmm.**

R: So we normally send this message to, so who have that will come and we will meet.

M: **I will mention some of them, probation services…**

R: Hmmm.

M: **Care homes.**

R: Yes, care homes.

M: **Access to therapists?**

R: Hmmm.

M: **Do the children in district have these available?**

R: Erh, for access to therapist here, they normally go to the regional level if they need fare, to the hospital setting…

M: **Hmmm, to Tamale.**

R: To Tamale, where they access the therapist there. But the other one you mentioned of…

M: **The care home (phone rings).**

R: Yeah the care home, here we don't have the care home; we have early childhood development centres.

M: **Hmmm.**

R: Aha we don't have the children's home.

M: **Hmmm.**

R: Erheh, where these people are. For Kpandai we don't have it.

M: **So the early childhood development centres…**

R: Hmmm.

M: **What is it that they do for the children?**

R: Erh, it is in the form of nursery, they give tuition, teaching services…

M: **Aah nursery and kindergarten.**

R: Kindergarten.

M: **Crèche.**

R: Yes.

M: **Ok.**

R: It is these people.

M: **Aha, ok.**

R: They give it to them.

M: **And you don't have the addition services?**

R: No. no.

M: **Ok, we have 2 more to go.**

R: Ok.

M: **In your view…**

R: Yes.

M: **Kindly tell me your view on formal and informal child protection services available in this community.**

R: Child protection services like the formal (phone rings), we have..

M: **Can we take a break?**

R: Hmmm.

M: **Before the break, let me take the question again. What is your view on informal and formal child protection services?**

R: Hmmm.

M: **Available in this community.**

R: Ok, this district the social welfare services is there for child protection and that kind of thing. In the community level we have formed some committees like the message ballot committee.

M: Hmmm.

R: Yeah, to promote child development this thing welfare over there.

M: Hmmm.

R: And so this is what if they have some minor cases on the child we handle. But major we refer we normally refer to this place.

M: Hmmm.

R: Yes.

M: Ok and these you mentioned that they help in child related issues.

R: Yes.

M: Do they also tackle disputes between children adults and their parents?

R: Yes like I said.

M: The informal…the community

R: Yes the informal, we are training them, that is at the community level.

M: Ah ok.

R: Yes. We train domestic violence, so the communities they handle these small cases. But when they are the major if we see that they are major, when they are going off track then maybe they refer it to us. But at the community level we are doing well.

M: Ok.

M: So why did you create that at the community level?

R: Because if you look at sometimes the distances are far, our district is a wide district.

M: Hmmm.

R: Aha, so we put these institutions to be there, to handle it. Because we cannot even hear getting access to phone, erh…

M: Internet service.

R: Yes, the connectivity sometimes is not there. But at their level there, they can sit down and do and possibly come and tell us that this is what they have done. I normally call them for a meeting, a review. So like 8 districts or each community will tell us what they have done so far. These are the activities they have done, and there are challenges and their success.

M: Are the community members aware of this?

R: Yes. They are aware (phone rings). They are aware, because normally. We go out there; there is a regional hospital and police out there. Come here, we will form a committee and then we move there; both the religious, the Christian and the Islamic. And we make them pick their people who will be in charge of (laughing). So…

M: Ok, who will be in charge of those issues.

R: Yes.

M: What type of cases will you use formal system to solve? Can you please tell me the case; you will use the formal system to solve? And what are your reasons?

R: The formal case I will use to solve if a case is referred here. Because here we are with the legal, like the police, so when we are not able to but majority are being solved here, but when we are not able to. When we look at the arrogance displayed, for instance when we looked at is it the complainant or the respondent? He is proving arrogant and trying to bring some physical this thing then we try to involve the police. We just call the police to handcuff the person and go.

M: Hmmm. Ok, the time you use the formal is when the person is becoming uncontrollable.

R: Yes, Very good.

M: Then you bring in the police.

R: Yes.

M: And when do you use the informal?

R: The informal is like I was mentioning the community level…

M: Hmmm.

R: Where they handle and report to us.

M: **And so what was the outcome of the formal system that you have so far used to resolve cases?**

R: Hmmm.

M: **What was the outcome? May be you can just cite one, take one as an example and tell me the outcome of it.**

R: In here, I was the domestic violence…

M: **Huh.**

R: So there was an instance, a husband assaulted the wife. And the lady came and report. And we invited the person. Unfortunately the person is even a teacher, he came and sat down. And we said this is what, there are laws that binds, may be if you assault this thing; that is a criminal. You know we have to take you through so these are. So from henceforth you make sure you treat the woman, because the woman has some scars and that thing. So finally we went, because I have to tell the police; she may have been forced to sign and maybe the husband may have been arrested. So we have to use dialogue and then bring them together. And as we speak now they are now happy staying together.

M: **My final question; we would also want to know more about another case.**

R: Hmmm.

M: **That your outfit adopted the informal way to solve it.**

R: Hmmm.

M: **And what the outcome was?**

R: Yes the informal.

M: **Yes.**

R: So the informal I was telling you about the community…

M: **The community…**

R: The community this thing

M: **The committees that you have…**

R: Aah, the committee.

M: **Yes.**

R: Yes there is a community called Balai.

M: **Hmmm.**

R: A man accused the husband of flirt, of being flirting outside and then the…

M: **A man accused the wife…**

R: The wife.

M: **Ok. Flirting…**

R: Of going outside the matrimonial or the marital home, and there was a case. And even accuse one boy to be the reason behind it. And then the lady called me, and that was the issues. Is within her level, a woman called Stella so we use the expertise, we taught her to see how she can resolve the issue. So she went in and then she was able to calm down and talk to them. And then when the regional doctor came for our review meeting and the woman was there to speak out this issue and said this was what the case she handled. And I think that national, we celebrate is it the this DOVSU; they are came to Kpandai here and because of the issues, we celebrated here.

M: **Ok.**

R: Yes.

M: **These are all the questions I have for you.**

R: Ok.

M: **But before end our discussion, may be you have something that you thought you would have asked.**

R: Hmmm.

M: **From beginning to end, you didn't ask, it didn't come up.**

R: Hmmm.

M: **And you feel it is important in relation to the topic we are discussing, parenting the children, if there is something that you have not spoken about.**

R: Yeah.

M: **And you want us to know.**

R: You see when we are talking of institutional welfare; here we have cases some of them are very violent.

M: **Hmmm.**

R: Because in trying to bring families together, the husband may try to be very violent. And we would wish that if the government will include police attached to the social welfare offices. Erheh.

M: **That is DOVSU.**

R: Yes, yes. Attached to officers, so that these violence cases will stop, because in a case one of them wanted to assault one of my boys here and then we had to call in the police to come. Aha. You see so these are some of the issues (laughing).

M: **If you don't any other thing.**

R: Erh; but here some of the people come here poorer and poor and at the end of the day they realise that this is social welfare, and they coming for money to buy food for their children. They are single mothers; they are widows and that kind of thing. And our setting here there is no fund meant for that.

M: **Hmmm.**

R: Erheh, so I think er, if we could have, UNICEF can make a package for us that we can access and we can take to support these people, I think it will go a long way to help.

M: **Ok.**

R: Er

M: **Thank you very much.**

R: Thank you very much.

M: **For your time. The discussion has ended.**

R: Hmmm.

M: **And the time is 10:34.**

R: That is ok.

M: **It was fruitful.**

R: Yeah.

M: **Thank you.**

M: **In addition to…**

R: Yes.

M: **The discussion that I had with the rep for social welfare, actually he is the in charge, the boss…**

R: Yes.

M: **The boss for social welfare Kpandai.**

R: Hmmm. Yes there is case from Wiah, a married couple and then he has two children with his wife. And then the husband accused her of having extra marriage relations and one guy is being identified from Ketejele; who's been sleeping with the wife. And when the guy made an arrest and the boy replied we had to call the chief from Ketejele you know to come and apologise to the husband and say No that she doesn't know he is still married to the man.

M: **Hmmm.**

R: Meanwhile those people they have been there for almost 2 years now. So the guy, the husband confiscated the boys motor bike and then we had to come in and finally the matter brought to police. So the police said this is a civil issue. They should bring it to the social welfare. So that is why they are here for us to solve the matter.

M: **Hmmm, ah those I came to meet.**

R: Yes those; you came to meet.

M: **Ok.**

R: So that is the issue we are…

M: **Hmmm.**

R: we are treating this morning.

M: **(Laughing). Thank you.**

R: You are welcome.

M: **Ok, bye.**

R: Erh.

TRANSCRIPT OF NTN_NT_TO_DSW_090519

F: Please describe for us the general work of your institution?

R: The general work of our institution is to empower the vulnerable, the excluded, the disadvantaged in society by improving their living standards. And we also have community engagements in a form of sensitization, and all these are geared towards improving their living standards

F: What is your role in this institution? You said you are the director, what is role?

R: My role actually is to actually coordinate and supervise field officers and also to see to the day to day administration of the department. I supervise, I monitor, I coordinate the activities of field officers in the district and also report to the coordinating director and copied to my regional directorate.

F: When you say you report, what kind of report do you give?

R: We do quarterly reporting but we also have activity reporting for the district. Every quarter, it is mandatory for us to report whatever activities that has taken place in the district during the quarter. Sometimes too, when we have work, like we have an activity, you will finish activity and still write an activity report

F: In relation to children and adolescents, what do your department do?

R: Yeah, with children, our department is deeply involved in promoting their rights and also promoting their welfare in terms of education, in terms of health and in terms of their abuses. We ensure that children abuse and violations are not encouraged. We sensitize parents on the need to protect children's rights, the need to guide them and we do a lot things and we call it child protection. We do child protection, we do protection, prevention and promotion of their rights

F: Besides the sensitization that you do in relation to children, what else do you do?

R: Yeah. In terms of the children we also engage some of the parents to know practical engagements. You know sometimes we have something we call child protection tool manual. So we sometimes let them see practically, how they can help bring them up as meaningful citizens. Yeah.

F: Tell us about some of the services you provide to parents, but before that, I asked about what you do, the role that your department play with regards to children, how about adolescents?

R: Yes

F: Is it the same, or?

R: Yeah, it's the same, is almost the same. But sometimes our emphasis on children is more intensified, even though we also engage adolscents, because we believe that they are in their useful age. They do a lot of things out of ignorance, so we try to engage them. We try to let them know that the wold is not like the way they are looking at it. The world is not the way they are looking at it

F: Ok, please hold on, so what do you do with the adolescent, is it the same sensitization?

R: Yeah, we do a lot of sensitization.

F: How do you do the sensitization?

R: Yeah, the sensitization is in a form of education. We go to the communities, talk to them, we organise them in a meeting, it could be community durbar, it could in a form of a community durbar. It could also just be in form of focus group discussion, and then we meet them, we try to sit with them, listen to them and tell them what they should do or what they should know and what they should not do.

F: How often do you do that in a year?

R: Sometimes UNICEF support us, so it depends. We also have government when we have our funds, they will also something similar. But sometimes, UNICEF support us, we call it child protection program. They support us to do the sensitization or the community engagement

F: Please tell us some of the services that you provide to parents, caregivers and children. What are some of the things you do, the services you provide to parents, caregivers and children?

R: Yeah, like we link them to opportunities. But when I say linking them to opportunities, what it means is that …

F: You mean the parents or the children?

R: The parents, for instance, like government policies that are geared towards improving the vulnerable. We link them to these opportunities and we sometimes let them know about these programs and how helpful those programs can be. In the communities, they are not privy to some information, so we let them to know. For instance, there is a program, and if you are this or that, you can benefit from it. For instance, when you are LEAP beneficiary, you are entitled to national health insurance free registration and renewal. You don't need to pay anything, so we encourage them to take that advantage.

F: So bring them closer to these opportunities?

R: Yes

F: What else do you do?

R: We also help in terms of project management. When I say project management, project can be anything, in terms of infrastructure, it could also be like self-help project, like coming out to have KVIP or a community latrine, we help them to construct it and how to manage it

F: How do you help them to construct it?

R: We work in close partnership with Environmental Health Unit. When they see that a community or a household need a latrine for instance, then we involve environmental health unit and they come with their expertise.

F: I will be mentioning something to see whether it is part of the work that you do. I know you have already mentioned child protection services as part of the things that you do, how do you that when it comes to child protection, what kind of services do you provide? What do you do?

R: It's the method that ….

F: What things do you do to promote child protection?

R: I was saying community engagement, like we will engage them one to one. We also can have durbar and we will talk of the effects of child abuses, the effects of teenage pregnancy, the effects of things that children are not supposed to be engaged in, so we let them see the reality. So we have some methods that we sometimes use. We have some tools that we sometime use and these could be flat cards. Sometimes we also do balloon games and lots of tools that we do

F: That you do at these durbars?

R: Yes, during the durbars or during the talking, and sometimes we even work in partnership with Centre for National Culture. They can come in a form of drama, they will have a drama and the drama will aimed at conveying the message of going to 'kayaaye'. At the end, they themselves will tell whether it is good or not. We try to make it practical.

F: Do you do return to school counselling, is it part of what you do?

R: Yes, like those who have been unfortunate to be pregnant and they have dropped out of school. We also encourage them to go back to school. They should return to school. It is part of our sensitization program

F: How do you identify these children?

R: We work. We have volunteers or what we call community contact persons. Sometimes we link with the community contact persons and they will identify those specific groups for us.

F: How about violent prevention, is it part of the work you do?

R: Yes, it is part of our work. Because like I said, child protection that I earlier on mentioned, we do the effects of child abuses, violations. Child trafficking, we do a lot of talks on that

F: Ok, rehabilitation of abused children, is it part of the things that you do as a department? Is it part the services that you provide to children and parents?

R: Yeah, is part, but you know the districts do not have rehabilitation centres. But we sometimes do referral, if we see, we can refer them to the regional level and they can take charge of that.

F: Do you do counselling of violent parents

R: Yes we do

F: May I know how you do that?

R: Yeah, we do it in a form of sensitization, in a form of talks. We can invite the parent, opinion leaders, or together with the community elders to talk to the person concerned. Because if we think that, we are going to talk to the person concerned, we may have problems with him or her, so we try to involve opinion leaders in such cases. Sometimes, we even involve chiefs.

F: Chiefs within the communities?

R: Yes, those who will talk or those who will speak and the person will listen. Especially when it comes to violence, when we look at violence against children, sometimes our communities still think that, it is their children or that is their child and they can do anything at all. Sometimes we say no, although you brought this person to the world, you do not own the person's life…..eheee

F: How about prosecution of offending children and parents?

R: Yeah, it is part because we work with the police but sometimes we believe in dialogue. We believe in talking to the parents because the prosecutions are not appropriate

F: Why do you say that?

R: The reason is when you at northern culture, it doesn't help, the prosecution doesn't help. Ahaa. The prosecution doesn't help northern culture. Well, you can prosecute the person, but the question is after the prosecution, can you take responsibility of the child. So when you come, you talk to the person and let the person understand how he or she should have gone about it. For instance, we had a case at a community under Nyankpala called kukuo Naayili.

There was a case of a father forcing his daughter to get married and the girl was even under age because she was below 18 years. So we had to come in by talking to the father and even the father did not want to listen, so we involved the chief and the opinion leaders and he understood. Now the girl is back to school. And so sometimes, assuming we had made an arrest, the issue is that would he be prepared to do what you are thinking. It is a gradual process. You know northern culture, sending someone to police station or reporting someone to a chief is not all encouraged. When you do that there are a lot of questions.

F: Is it a cultural thing?

R: Yes, it is a cultural thing.

F: The last one, how about stigmatization of children with special needs?

R: Yeah, for that one, we were even having a program called inclusive education, so …

F: A project by your department?

R: By UNICEF. It was sponsored by UNICEF together with GES. We were part. As for the sensitization of the stigma on disability, in our district I don't think we have much to do because we let them know they are human beings like us. It does not mean because they are having a form of disability, they cannot do what we can do. And you see a lot sensitization have gone on as far as disability is concerned, so there is no stigma as far as our district is concerned.

F: Ok, you don't find that here?

R: We don't find here

F: Based on your experience in working in the Tolon district, can you tell us your observations on child relationships? How do parents relate with their children and how do children relate with their parents?

R: Yeah

F: What have you observed?

R: It is improving. When I say it is improving, some communities, especially where child protection programs are existing, they are realising that there is the need for them to give attention to children, to let children to also have a voice. But not withstanding, some communities still have a few challenges. But that does not mean that there is no cordial relationship between children and their parents. They relate well, they listen to them. But some communities too, we still face a lot challenges with regards to

F: Are children able to say what they want to say?

R: Yes, some communities, they are able to say.

F: Ok, what interventions do you have in streamlining the relationship that exist between children and parents, which are inconsistent with the environment? What have you done?

R: You know as a government institution, if we do not have support, it is always a challenge. But we are also doing our best, the little support that we are getting from donors like UNICEF and also sometimes we get a little of quarterly impress. And when we get, we use it to do purely sensitization. We believe that sometimes, it is ignorance. That is what we believe. If somebody knows what is good, because we always let them know that the children are the future leaders, so proper attention should be given to them. But we believe that the system that we work is just based on talking to them, sensitizing them, educating them on the need to give children the needed attention. But we don't have like, say programs, funding programs, besides the child protection program that UNICEF is supporting. And we don't have our own funds.

F: So besides UNICEF supporting, there is no other funds coming elsewhere, including government? Is there any other finds from somewhere?

R: Government help us in the form of quarterly impress. It is not even every quarter, sometimes it comes, other times, it doesn't come.

F: The next question is to know the interventions that you have in streamlining or aligning parent or guardians – child relationships for guardians that have children with special needs, that are inconsistent with the child welfare environment? Do you have something that you do in that regard?

R: Like having methods or systems in place …

F: Yes, for children of parents with disabilities. You know there are some environments that are not consistent for child with a disability to be in, do you have some programs that are geared towards that?

R: Specifically, like I said, is education. But in Tolon district specifically, where I am coming from, I don't find issues like that. Children with disabilities are accepted, especially for the past three years up to now, children with disabilities are accepted. You see like I said earlier, it is just sometimes out of ignorance, but when they have the information that a child that has a disability does not mean that it is a curse. So sometimes when you let them know, well, it may be happening somewhere but as far as Tolon or communities in Tolon are concerned, it does not happen.

F: Are these children accepted?

R: Yeah, they are well accepted

F: Are they encouraged? Are they put through schools? Are they also put through some form of training?

R: Yes, like you said, we, sometimes when you see a child with disability, it depends

F: Can we use the word special needs, so that our discussion is not limited

R: Its true. You see when you have children with special needs, it depends on the kind of need or the kind of special need that the child has, for instance if a child is blind, and they come to us, what we do is work with them to teaching hospital and from there, if the child is recommended to go to school for blind at Wa, we facilitate the process

F: When you say you facilitate the process, what do you mean?

R: Facilitating the process means, we have a fund we call the disability fund. And this fund is used to support the child – admission, prospectus and we even pay some money into the school accounts, so that when the child gets to the school, he can feed on. For instance, this particular month, we are sponsoring four children of those nature. The district assembly through the disability fund facilitate children with special needs, especially needs that cannot be handled at the district level

F: What type of needs?

R: Like I mentioned earlier, children that are declared or proven by a medical doctor to be blind will be facilitated by us to the child financially. And the fund will be used by the needed prospectus, and for the upkeep of the child to school. And we also let the parents to know that, once he is having that special need, does not mean that he cannot go to school. We make sure that we let their parents to know. I have a lot examples on my phone, after we are done with questions, I will them to you

F: I remember the last time I called you and you said you had sent a child to the hospital, is that one of the examples?

R: Excellent. That is one of the examples.

F: How do assess the type of coordination that exist among child and adolescent related institutions. You are an institution, what other institutions do you work with? What coordination happens among these institutions?

R: In my district, we have a very cordial relationship. We meet with NCCE, we coordinate with CHRAG, we also coordinate with GES, we coordinate with health, we coordinate with NGOs like Basic Needs, DOVSU and then Right To Play and organisations that are into child protection. We have a very cordial relation

F: How about Ministry of Gender and Social Protection

R: Yes, it is also part because that is where department of social welfare fall under.

F: So how is information shared among all these institutions?

R: Yes, we share information by workshops, where am just coming from. I am coming from a NORSAC inception workshop. And the workshop is just purely on social protection, purely on LEAP beneficiaries, how they can support LEAP beneficiaries to be financially independent, how they can graduate those that potentially productive to graduate. To graduate means to exit them and make financially independent. So this is a coordination because if we were not having a proper coordination, I wouldn't have been invited. I even went there with my officer who is the LEAP Focal Officer, he is in charge purely on LEAP

F: So our discussion here is centred on children and adolescents

R: Yes

F: So when it comes to information sharing, besides the sharing information during workshops, what else do you

R: Sometimes they bring questions like you are asking me. They come to ask basic questions like what are the issues regarding this and that. So it si a form of sharing information. And maybe say, if we know this organization is working into this, and we have a particular issue, that relates to that organisation, we can go directly to them

F: So do you also for instance you need this information, you can go to say DOVSU to collect that, is it part of what you do?

R: Yeah, it is part. But it depends on the issue. If the issue is has not been brought before you, you cannot do anything. If there is an issue that has been brought before you, we go to get information

F: Do you meet?

R: Yeah, we meet, especially when this a workshop. Because sometimes workshops regarding child protection or social protection, all these organisations you have mentioned, we come together. So there, it is an opportunity for us to share.

F: Besides the workshops, do you have some other scheduled meetings that you have? For instance, ones that you would have health or GES or DOVSU or the police, do you have some scheduled meetings?

R: Yeah, we are supposed to have scheduled meetings because there is a committee known as District Social Protection Committee. And this committee brings on board some of the key stakeholders for discussion?

F: How often do they meet?

R: We are supposed to meet once in a quarter, but sometimes due to lack of funds, we are unable to meet. But when there is a pending issue, we meet. There is also a committee at the district assembly level called Social Services Sub-Committee. All these things are discussed

F: Does it involve children and adolescents?

R: Yes, everything is inclusive. It involves children and adolescents. And it also involves other social issues

F: How about data collection and reporting on issues of children and adolescents?

R: For data collection, for the department, …

F: Do you work together as departments on that?

R: Yeah, we do, especially, because of lack of funds we centre on these day care centres.

F: In the schools?

R: In the schools, it is the place where we have children and we go to have a data on them. Even though it is not part of our duty to collect data on children and adolescents, that does not mean that when we have funds, we cannot do. We can also do

F: These departments and organisations that we have just mentioned, does every one of them have clear rules when it comes to children and adolescents, right?

R: Yes

F: Do you find a distinction in the way you work?

R: They have specific rules, but especially with the children. For the adolescents, it is just recently that an NGO called NORSAC is introducing a project targeting adolescents. And also it this year that UNICEF is also helping us, supporting us

F: When you say helping us, you mean social welfare or some other department?

R: Yeah, supporting social welfare for community development. To actually look at the adolescent. That was the workshop or the meeting that we had last week and you were looking for me. It was one full week workshop or meeting

F: That was during the holidays

R: During the holidays, yes. And also we are yet to actually go into the adolescents, but previously we had been concentrating on the child protection

F: I asked earlier about the data collection and reporting and you said you normally go to the day care centre to collect data on children, how would you describe the quality of the data you take there?

R: The data we take there is because, for instance, in Tolon district we have about 15 day care centres. So the data we take there, I will say the quality is not bad. But data on children and adolescents, we do not have for now, becauses resources to collect this data has not been provided to go and take this data. Because if you ask me how many children we have in Tolon, I won't be able to tell you.

F: When you say resources have not been provided, what resources are you talking about?

R: Resources in terms of funds, in terms of motor bikes, or not necessarily motor bikes but a means to get to the communities, any kind of means to get to the community to take the data

F: Do you take data, for instance from the other departments? Let's say, you go to GES or GHS?

R: Yeah, we do and they also do. For instance if they need data and they think we would have it, they would come. If we also need data and we think GES will have it, we would also go

F: Please tell us some of the core issues in monitoring and supervising for child related organisations, when it comes to monitoring, does it conform to national or international regulations and rules?

R: Well, I can't say much because I don't know much about the international regulations. But I believe that what we are doing is in line with our country's rules and regulations, because we don't just pomp in trying to help children. Before we go, we will have to observe the community level entry process by meeting the chief, meeting the opinion leaders, meeting the assembly persons, so we make sure that we follow the right process before we get the audience, before we get our target group, before we get the children. So I wouldn't say we don't follow the right procedure we just don't walk to the community and start talking to children or we start talking to parents. Before we even go, we have to and see the chief, by

letting him to know we are coming on so so and so date and we are coming to engage and this is the purpose. And he will give us the permission. Sometimes, it is even the assembly person who will lead us there. So once we involve these key stakeholders in the community, we think we are not against any rules and regulations. But it could happen that international standard, even though I do not have much information, it may require that you have a paper before you do certain things. For that one we don't do it.

F: But, how about within your department, the monitoring and supervision that you do, is it in compliance with national regulations?

R: Yes, it is

F: How do you do it?

R: In terms of the child protection, right? You see, before we go out, we have an itinerary and we have our activity, and we also have maybe questions for what we intend to do, and we do it in line with national standards. We just don't do out of just doing. And then monitoring, we go to see after sensitizing or talking to children or parents or stakeholders. We want to see the steps they are taking, are they improving from where they were to where they are. We try to monitor. For instance, like I said earlier, if parents were encouraging children to go to 'kayaaye' or moving children out of school, and we go to sensitize them on that. In the next month or two, we will go there to try to see whether some have been sent to the 'kayaye' or not. If we see that even one or two has not been encouraged to go, then we will know that our sensitization is making an impact or they are taking the education we give to them

F: Can you describe to us what makes children unsafe in your district?

R: The use of mobile phone is one.

F: What makes it unsafe for use by children?

R: You see, one, it promotes a lot of teenage pregnancy because once children sometimes use phone, if their peers want them, they do not come into their homes for the parents to see. They just use the phone, and phones that are smart, there are a lot things that they can see and would want to practise. Especially with regards to the adolescent, because they are so curious at that age. They want to see whether what they have seen in the phone and want to practise. Apart from that, you see, like I said, especially in the night, especially where in a community that have what we call 'ghetos', it is also another spot that makes children unsafe, and even adolescents.

F: How does the 'ghetos' make them unsafe?

R: You know the 'ghetos', they do a lot of things there

F: So the 'gheto' here means a sitting place that these children sit

R: Yes, especially the adolescents

F: Ok

R: When they sit there, especially in the night, they do not discuss things that will help them, they rather discuss things that will not help them.

F: What are some of the things that they are likely to be discussing?

R: For instance, issues of 'sakawa', they discuss it there

F: Ok

R: Issues like friendling one another is also a possibility

F: Between boys and girls or of the same sex?

R: Oh no, is between boys and girls. Not the same sex

F: Besides that, what other economic factors would you say make children unsafe?

R: Ok, like giving provisions to them to go round to sell, and also ….

F: How does that make them unsafe?

R: Yes, it makes them unsafe because sometimes they leave school and be doing the selling. And so you can see that it is not safe. Some parents can even take their children out of school and let them to be engaged in selling these provisions. That is why I say, it is not safe

F: What are some of the social and cultural factors that you think make children unsafe in the district? Something of social and cultural factors that make children unsafe.

R: You see, these days it is no longer common to find those things in our district. Like promoting male education to the detriment of the female, like parents encouraging that girls would marry and leave their house, so they see them members of a different house. But they would rather encourage the males to go. But these days, with the sensitization and the education going on, parents are not just emphasising on only the boy – child education, but they now emphasise on both sexes. So culturally, I wouldn't say we are experiencing much in our district as far unsafe nature of children are concerned. But if you have one, let us discuss it and maybe if we have it, I will know

F: I do not have anything to share, I just wanted to understand whether there are some social or cultural factors that make children unsafe. Ok, I know we have already talked about children with special needs and you said stigmatization is not there, do such children feel safe?

R: I wouldn't say they do not feel safe because majority of the disabled children that I happen to be meeting are with their parents and you know it is not easy to bring up a child. When you have a child of that nature and you had fears that it is a curse and you find somebody to talk to you that, it is not a curse, it is rather a blessing. Give the person attention, maybe he will grow up to be useful to you the parent than the one we call the abled person. So they do not feel so much challenge, those that will be having challenges are the ones that have unfortunately lost their biological parents

F: The orphans?

R: Yes, the orphans. They may have issues.

F: What kind of issues?

R: Like issues of neglect. They may not be given proper attention. But in my district I have not find any of that. And I am just speaking generally

F: Tell me about your perspective on rehabilitation services for children in your district.

R: Rehabilitation

F: I know earlier you told me that you don't have any rehabilitation centre in your district, what you normally do is refer to Tamale

R: Yes

F: Ok, but is there something that you do when it comes to the rehabilitation?

R: Yeah. We do. When we see that someone is having that issue, what we can do is to assign an officer to do the referral in order to facilitate the process. This is because the community members find it difficult to go to Tamale, because of their illiteracy, they feel they would not be able to present themselves well at the referred centre. What we do then is to assign an officer to send them to the regional level

F: Do you have probation services?

R: At the district, no

F: Where do you access that?

R: You know, because Tolon happens not to be very far from Tamale, we do all that there

F: You find care homes too in Tamale

R: Yeah

F: How about access to therapist

R: I have to find out because cases that I know that our district can handle are issues of epilepsy, issues that has to do with some mild psychosis, those are the things that they handle

F: What is your view on formal child protection services that are available in your district?

R: Formal child protection?

F: Yes

R: Can you explain

F: For instance, for settling issues of children when children are abused or anything concerning children. Issues of parents and children that needs to be resolved

R: For our district, it is fantastic, as far as the issue is concerned. I always believe in dialogue, talking to them, letting them to know

F: Do you mean using the formal way to dialogue

R: Yes, using the formal way to dialogue. You see people of Tolon are easy to understand. It is the way an officer relates with them. Sometimes I believe that things that people do not understand is the manner you approach them. The best approach is to let the person know that, how bad or good the child turns out to be, the parent has a role to play. You let the parent understand that he is there to promote the welfare of the child. The interest of the child must also be seen as supreme. So if there is a problem and we do not use dialogue to resolve the issue, can we the officers be able to provide for all that the child will need? Even if the formal system is able to provide for the financial needs, how about the care and affection that the child will require? So sometimes, it is best to talk to the parent by sitting him down, hear what he has to say, then you let him understand there are certain ages that the child is bound to be behave in a particular way. It is not the fault of the child that he behaves in that, it is just the way are

F: So I got your right, your view is that we should use the informal way to resolve issues of child abuse

R: Yes

F: What type of cases would you use the formal systems to address?

R: If they are rape cases. That one goes beyond settling it in any other way

F: Has that issue ever come to your attention?

R: No

F: It has never come to your attention?

R: It has never come

F: How long have you been working in the district?

R: For the past three years

F: And something of that has never come to your attention?

R: Yes

F: So what other cases would you use the formal system to settle?

R: Maybe capital punishment, which is no longer existing. Or a severe corporal punishment of a child. You know a severe corporal punishment may not necessarily come from the biological parent. It may be coming from a community member. A child does something and you beat the child mercilessly, that one, you can use the formal to settle it.

F: Has it ever happened?

R: No

F: Not coming to your attention, but I just want to know whether in the communities, where you find parents who are not the biological parents beating children?

R: Well, I have not some. But what I heard was that there was a case that the DOVSU officer was narrating to me where a Fulani boy stole someone's bicycle and a group of people beat him to death. And that case is still in the court. But in our outfit, we have not come across an issue like that.

F: So what type of cases would you use the informal structure to resolve?

R: Cases like child neglect, then sometimes, teenage pregnancy as well. Especially when the parents of the boy and the girl do not see it as a problem, you cannot say it is problem, so you cannot come in. and cases like 'kayaaye'

F: When a girl go to 'kayaaye'

R: Yeah. Because sometimes it the mother of the girls who encourage them to go. So when see a man and his wife quarrelling because their daughter has gone to 'Kayaaye', and maybe it is the woman who has encouraged her to go, then you can use the informal way to the mother and the father to see how best they can resolve it.

F: Thank so much for time. The last set of questions that I want to ask is, can you tell me about a case that you used the formal system to resolve?

R: For me, in Tolon, we have never used the formal sector to resolve an issue. Because we have never had a case that will require us to use the formal system to resolve. Because the people of Tolon see themselves as one. So sometimes, they will even have a case and it will not be brought to your notice.

F: Ok

R: So they will even resolve it before you get to hear about it.

F: I understand most of the issues are resolved at the chief's palace.

R: Yes, it is even recently that they have started sending issues to the police station. In Tolon. If there is a problem and it has to do with police, they have to go the chief's palace first. Because the chief is also an enlightened person, if he realizes that the police has to be involved, he will draw the attention of the police.

F: Has a case of that nature ever come to your attention?

R: Yeah. According to the DOVSU officer, like the case I mentioned earlier

F: The case about the Fulani man stealing a bicycle

R: Yes, and he was beaten to death

F: Was the Fulani a child or an adult.

R: I think he was not a child but the age of an adolescent. But I don't know the age. So according to the DOVSU officer, he says he is not even a DOVSU officer, that he is a crime officer. So according to the crime officer, he said the case was referred to them from the chief's palace.

F: I know you have given me several instances, could you give me an instance where you use the informal structure to resolve? Please tell me what happened? Just the story line

R: For instance, a girl was forced to marry, before the one I talked about

F: Are you talking of the Nyankpala one?

R: Yes. A community under Nyankapla called Kukuo Naayili. The one I am talked about happened two years ago at a community called Dingoni. A girl was forced into marriage and the man was beating the man and the girl ran away, even though she was not in school. The parents asked the girl to go back and then we came in. We even partnered with NORSAC and we talked to both parents and the case was resolved amicably and the girl was given, let me say her freedom. The girl didn't like the man and the parents forced on the man. They had a little quarrel and the way the man beat the girl was not good and she ran to her parents and they wanted the girl to go back and she refused. It was at this point that it came to our attention and we got involved. And we used the informal to solve it. We worked with the chief as well as the parents of the girl and the man. We always talk to them and see that they understand each other, but we are unable to then there is no option than to refer it to DOVSU

F: Has that happened before?

R: We normally are able to resolve it at the house. Like I said earlier, northern Ghana, we don't want to get involved in our issues. So we normally tell them that if they do not listen to what you are telling them, then it may go to DOVSU or the police station or subsequently to the law court, and they will understand

F: So what do you think motivates parents to force their children into marriage?

R: Sometimes it is poverty

F: They don't have money to take care of the children?

R: Yes, that is because they give birth anyhow and without planning. Ignorance is one and poverty too is another. Like the case I cited earlier in the Nyankpala community, when you delve much into them, it was that the man provided just One Thousand Cedis to the girl's father. When the chief was involved in the matter, the parent explained it is not that he does want the child to through school. The problem is that the money that the man gave him, he has already spent and that he cannot get the One Thousand back, that is why he had send the daughter back to the man. So you will see that it is poverty. And it is ignorance because the family is big and he does not know how to take care of them. So if someone comes to marry them, the parent sees that to be reducing his responsibility.

F: Thank so much. I really appreciate the time that you have spent with us. You have been very patient with us. And thank you for sharing your inputs with us

R: Thank you

F: Do you have any additional comments that you would like to share with us as far as the issues we have discussed with us is concerned.

R: I don’t have. Thank you (then laughs)

F: Thank you too.

M:

M: fine. Please can you describe to us in general, the work of your institution?

R: thank you. Department of social welfare is under the ministry of gender, children and social protection. And the department has three core program areas in which we operate. We have community care, child rights promotion and protection and justice administration. Child rights promotion and protection is one of the major areas that we operate to ensure that children's rights are protected [00:02:22.16 inaudible], community members to help promote child rights in the communities. Under that, we have case work with families. Case work with families, we have a number of types of cases under this heading.

M: now can you tell me your role in this institution?

R: my role specifically as the head of social welfare unit or the role of the department?

M: you as a person, your role in the institution?

R: well, as I said, I am to make sure that all these three program areas I have mentioned… I haven't gone into details to explain them, that these three core areas of the department, are well performed and reported to the regional level for onward report to the national level. But as you said...

M: your unit...your roles as the unit's head here, what role specifically are you performing?

R: well that is what I am saying, is it the role that I play towards parenting or...?

M: yes, yes, may I know your position in this office?

R: oh, I am the head. Everything concerning social welfare, I am the head of social welfare in Lamusi district.

M: thank you. In relation to children and adolescent, what roles does your department play?

R: as I mentioned earlier on, these three program areas, all have role to play in terms of child parenting and child protection. But the major one has to do with child rights promotion and protection. That is what I want to, in a way, if I am given the chance, I will explain into details for you to get the roles that we play as department.

M: ok, yes

R: under this child rights promotion and protection, as I have mentioned, we have case work with families. And case work with families you have type of cases we deal with.

M: the relationship to children and adolescents, that is what you....

R: yes, that is what I am trying to...

M: ok, so now can you tell us or tell me about some of the services you provide to parents, caretakers and children?

R: if you allow me to touch on each of the departments in each of the program areas, you will see how the department contributes to child protection or parenting in the district. I think you have been hearing this LEAP program [livelihood empowerment against poverty]. That program, we try to identify poorest households that we assist to be receiving catch ground...

M: but that is to the parents

R: to assist children in terms of provision of pens, pencils, just educational materials and also make sure that children will eat before they go to school. So we assist parents in terms of catch ground through LEAP to help take care of the children. That is under community care. And when you come to child rights promotion and protection, we also make sure that in terms of separation, we bring the two parties together. I mean the man and the woman; we try to in a way bring them together to take good care of the children. But we are not magicians so we tried and then we failed. What we do is that we try to let both parties contribute their quota in taking care of the children. For instance, if the man provides food, it is the duty of the woman to also turn it into food on table for the child to eat. If the man provides soap, it is the duty of the woman to wash the clothing very well for the child to use. So when we tried to bring them together and we failed, we tried to let the two parties contribute their quota in bringing up the child. We realized that if two elephants are fighting, it is the ground that suffers and the grounds have not done anything that is the child. We make sure that, the kind of disagreement or whatsoever will not affect the child's future. And then we have paternity cases that we deal with. Some, there may be a pregnancy and the lady intends pointing at a man that he is responsible and the man declines. So we try to make sure that, though the father of the child, and vice versa. The woman can also send the pregnancy of another man to another man and the man can also protest.

M: do you give counselling to children to return to school?

R: yes! Counselling...I am coming to talk about what we call child labor, child neglect, street children and other things. They all come under child rights promotion and protection. That is where I am coming to but as you have jumped up to this place I would like to go there.

M: yes, you know, I want us to know specifically whether some of those children staying out of school, maybe you counsel them. You have a program that you counsel...

R: before you counsel there should be a case.

M: but you have that program?

R: yes we do counsel

M: ok, what about violence prevention?

R: violence prevention they all fall under that. Our department, that is, I think some of our officers are supposed to be - we are lacking staff - supposed to be with the DOVVSU unit. Those days, people use to call WAJU; it is not WAJU. Now men have also in a way

forced their way through, that no! A woman can also hurt a man, not only man that can hurt a woman. So we have domestic violence and victim support unit and we have our officers attached to it.

M: what about rehabilitation of abused children?

R: yes! Rehabilitation of abused children is a major...the word rehabilitation is even a major program we also have in the department. We try to bring the person, in a way, back to his or her normal position; it has to do with this rehabilitation. When children are abused, probably the person has been in a way denied education or the person was in school for some time they decided to just take the child probably because he lost the parents or maybe the father is not responsible or the mother is not responsible and the person is suffering. When we identify that, we try to pull the child back to school. If the child is above school going age, we encourage the child in a way to learn a trade.

M: what about prosecution of offending children and parents?

R: yes, it is also there. If you go to the children's act, it has spell out clearly that, if you have given birth to a child and you refuse to even take care of the child and the case is reported, you come to court and you still don't want to take care of the child, you will go to prison or you will pay a fine and still go to prison. Or you will suffer one.

M: so we may want to ask, what about stigmatization of children with disabilities. How do you go into that...?

R: that one, we are all aware that in some communities, even some say they even taboo a disabled child. When they give birth to a child and the child is disabled, they do away with the child, they kill the child or they have to send the child away. Recently I went to one of the communities, I don't know how you call it but in the community, they say zuziea - when you give birth to a child and the child is hair is just reddish like albino -they said they don't accept such children in the community. And when it is like that, as we have identified those areas, we are very careful and we have people there who report to us about those cases. If it is possible, you just take the child out of the community to save the child's life. And we also sensitize communities to know that disability, they can give birth to you with disability, you can also become a disabled person at any time. And if people start to refuse children with disability and reject them from the community, if you are the chief, when you become blind or you have accident and you are amputated, will you reject yourself from the community? These are some of the things we do and actually they are accepting them. And with this disabled common fund, people now even cherish persons with disabilities even than children without disability.

M: **ok, based on your experience working in this community, please tell me about your observations on how parents/adult children relationship is in this community?**

R: I have realized that, when you give birth to a child, your child is supposed to be your friend. That is the normal way. Your child is supposed to be your friend, in the sense that when the child even has any problem, can approach you with it and you help address it. But it is rather the other way. In some families, even when children are playing and when adults are coming in, they have to stop; they don't even have the chance to talk. Children are not invited into family meetings to also bare out their views. The problems that are affecting them, it becomes difficult in our traditional communities here. We went to one community like that through this LEAP program and a name of somebody was [00:16:37.04 unintelligible] something like that, so I was asking them, "So children don't have rights?"

M: **so which means their relationship is not cordial.**

R: yes, it is very poor.

M: **so, now, what are the interventions do you have in streamlining or aligning parents/guardian child relationship that are inconsistent with child welfare environment?**

R: I think UNICEF equally run a program with us. It is child protection program. And one of them is also local government states, is also helping us implement a...

M: **please the UNICEF program is what?**

R: child protection program; and local government studies, child and family welfare program, and we also have this Action Aid child marriage program that we run but it has finished somewhere 2017. With these programs we actually educate parents on importance of child parent relationship. You have to make your child your friend, if not; your child will look for friends outside. But whatever the friend intends educating the child is the problem. Most at times it is the negative aspect. So you the adult have to make the child your friend. Let your child be your friend, then you educate your child on the good and the bad.

M: **thank you. So do you have intervention for disabled children that are also inconsistent in this environment? You mentioned something like, "now some parents now cherish children with disabilities"**

R: I think with the help of the disabled common fund, they thought these disabled children; persons with disabilities were just burdens on them. Now, the assembly

through our department assists to pays their fees, to buy assisting devices for them like wheelchair, white sticks and other things for them. Now they have even realized that, we have children that are supposed to be in the school for the deaf, school for the blind and how the parents can organize and even send them there is a problem. And with this disabled common fund, I think last year, we prepared and sent four of them to school for the deaf.

M: oh, that is a good intervention.

R: yes, we are organizing for three mental retarded children to Wa School for Mentally Retarded. Now, parents do come to report when their children are having disability and cannot be in the mainstream school. And I think very soon, this inclusive education would be on recess and we will not even be sending children to special schools. I think we had a workshop in later parts of 2018 with Ghana Persons with Disability Association. The regional and the national executives came down and then we organized such a meeting on this inclusive education. And now parents are aware that, they are no more burden as they used to think.

M: thank you. So how do you assess the type of coordination that exists among child and adolescent related institution? Eg. Department of social welfare, ministry of gender and social protection, division of family health of Ghana health service.

R: I don’t know why you didn’t mention GES.

M: oh, I am not done. We have DOVVSU of Ghana police service, ministry of education of Ghana education service. How do you assess the coordination between them?

R: very good. I think we have a very good relation with Ghana education service, Ghana health service, the police...unfortunately, the district here, we don’t have DOVVSU but the police service; we have a very good relationship with them. And then we partner protect children. Last year, Ghana education service referred to my office, 8 cases on teenage pregnancies and then I sat on those cases and then the children are well maintained now. The Ghana police service we have a very good relation with them. When there is a resistance on just this [00:24:40.15 inaudible] management system, we invite somebody, then we invite a party and then if the person is resisting, we fall on them to bring the person.

M: that is the police?

R: yes! The court is one of them, family tribunal court, the office happens to be a panel member of family tribunal court and juvenile court in Nandom. We have a very good

relation with them. We refer our cases to them and then we are part of the case sitting and then, they are doing a lot. Ghana health service, I think, recently we are even meeting on this malnutrition program and look at how we can also assist to get this baby for children with special cases in terms of health for us to help. I think Monday; I will be meeting the nutrition officer for us to have a meeting.

M: **so it means you have a cordial relationship with them too. Could you tell me something about the core issues in monitoring, supervision, child related organization in terms of compliance to national, international regulations and rules?**

R: the issue here is, there are rules and regulations from the national level but our challenge here is the resources, both human resource and material. Before you can get to the communities, you can't walk to the communities. You need fuel; you need motor bikes in a very good condition. And even, the department even needed a pickup. There are departments that don't do a quarter of our work and they have cars, pickups they do run their programs with. As at now I am telling you, you can see this motor bike here, in case it's stolen, how do you identify it as this is supposed to be a government department motor bike. You can easily get it in the market and buy, even in north here.

M: **so it means monitoring and supervision is a problem here.**

R: it is a problem. There are no funds to do that. We don't have anything that is sent down for us to manage. It is this LEAP program that when we are going there to do something we take that opportunity to do our supervision. Meanwhile, all these departments are supposed to be there but there are no resources for us to do that supervision. And the little we get from LEAP...

M **why is it so? Do you find out from, should I say maybe the DCE, why is it that there is no funding or you are saying you have scarce resources, why?**

R: well, it is not that we are not trying to approach our parents, we do. But district assembly also falls sometimes on revenue mobilization and other things. If there is no allocation from the national level for us, whatever they generated from the district will not be enough. You go; they will tell you there are no funds. And this 'there are no funds' have been an issue since I joined this department for more than 10years now.

M: **no allocation or no funding.**

R: that is it. This GoG which is also not forth coming.

M: **please describe what makes children unsafe in this community. Like economic, social and cultural factors that are making children unsafe in our community?**

R: wow! Let me say safe in terms of what? Health education...

M: like economic activities that are making them unsafe. Like some places they have galamseys...

R: fortunately, we don't have galamsey here. But the issue is, parenting is a serious problem here because most of our children are under the care of their grandparents. Their real parents are outside the district, even outside the region. So we have a case that, in most cases we have children that have parents and they don't have parents in the district. And looking at it, your grandmother or your grandfather, even traditionally is [00:31:36.28 inaudible] and they are also very weak. So when the child gets up and says "ok, I won't go to school today", what can the grandmother or grandfather do? They don't even know the importance of education. They won't bother themselves to be chasing the child to go to school. And even sometimes in the morning, how to get food to even eat before they go to school is a problem because their grandparents are so weak that they cannot even provide. So they go round and even be picking leftover food. When there is a workshop in Lamusi, you will realize that schooling hours, the children will be there competing for leftover food. And in this case, even if a food is poisoned in a way and thrown somewhere, they can even pick it and eat. It is unsafe. So as they are roaming about, as at yesterday, I met some of them, they are in between Lamusi and Nandom, they were there just in the bush. So if a madman in a way jump on them, there is trouble. Probably their parents don't even know at that time where they are and they don't care. We don't have issues like galamsey, stone quarry and other things that can even [00:33:09.03 inaudible] but we are not caring for our children.

M: the social vices like if you go to some places, we have this, is it motorist where children go or jamborees where they leave school and go. They don't go to schools, they only attend these places.

R: for Lamusi district we don't have this problem.

M: what about these Chinese machines?

R: for that one, it is there and especially it is in the remote areas...I realized that, that time I was sitting in the car with him and then he played this game twice. I asked the one monitoring it, "so do you allow children to also play this game?". He said, "yes, if you have your money how can I prevent you?". You don't question, where this child has gotten the money from to come and then do this. If it is the money of any adult person and they trace it here, definitely you will receive beatings and don't know the outcome.

M: so it means they allow them to play the games.

R: they play the games.

M: so what do we do? Was it during schooling hours that you met the child?

R: yes! Schooling hours. I think I even devoted time, 20 to 40minutes to educate those people around. What you sit down and allow the child to do and you are happy, your child will do that tomorrow. And when it happens, will you feel bad or you will feel good?

M: so about cultural practices. Like these our funerals...

R: it is very risky. Let me just give you an example. There was this girl, the father and the mother who are self-acclaimed funeral contractors. One will be here and the other will be there. And there is a young man who happens to be a youth employment teacher, he does come to the family there and have sex with the girl in front of her siblings and this girl became pregnant. The case was reported to the office. When I wanted to find out when and where they do have their sexual affairs, and the lady mentioned to me that, three times in the father's house when the mother was away and the father was also away for funeral.

M: but do we allow our children to attend these funerals?

R: they attend. When both parents can leave the family, leaving the children alone in the family, why can't you allow them to go to the funeral, especially when the funeral is in the same community or just a nearby community? Even not only the funeral, when we close from work in the evening, what I realize is that the mother will go to the friend's house to be chatting there. The father will also be with the friend somewhere chatting and the house is for the children alone. They can also decide to leave and do their own thing. So for that one, it will take a very hard and maybe let me say a difficult fasting to raise a child because children easily emulate or copy from adults.

M: so economically in lLamusi we don't have that one but socially and culture we have a major this thing.

R: yes!

M: what about disabilities. Children with disabilities feeling unsafe, do we have it in this community?

R: yes, some of the communities, it is unsafe. When you have community that doesn't entertain disabled children, you know automatically they are not safe. If a child is identified as a disabled child, quickly they have to do away with the child. This common

fund national team came here last year and we were organizing persons with disabilities, the district capital for some kind of registration. I went to a particular community just to inform them that they should let all persons with disabilities come to the district for that registration; and about three young guys told me that they don't have such people in their community. So I was wondering, is it that people don't give birth to persons with disabilities in the community or when they give birth to persons with disability they do away with them? And I did my own investigation and I realized that, that is the practice in that community. When they give birth to persons with disability...as I am telling you now, I have identified about 3 or 4 there now and they know now, persons with disability have support from district assembly, now some of them try to give up on the culture but some are not safe at all in some communities.

M: so what do we do?

R: it is all about sensitization. To let them know, we are not saying culture is not good but we have to weight it, the aspect that is not good we take it off. The aspect that is good too we encourage it. And taking the life of people, and I even questioned them, in your own community there are persons with disability who are now teachers and nurses and now they come to treat you and you receive the treatment. If they were also killed, will they have that kind of talent to come and contribute to the development of our communities? So it is all about sensitization.

M: sir, tell me about your prospective on rehabilitation services for children in this district.

R: probation services that is what I wanted to go to. I serve as a probation officer as well. When a child come into conflict with the law or contact with the law - we have conflict with the law and then we have contact with the law - conflict with the law is when a child is an offender, and contact with the law is when the child is going to court to fight his or her rights. When somebody offends that child, the child has come into contact with the law. And conflict with the law has to do with the child being an offender so for that matter, they don't treat the child as he or she is an adult. The case will be in a juvenile court.

M: so your perspective is what under that? Is it good, the probation services?

R: yes, probation services, court always asks us to do our invsetigation and come out with the report. We call that one SER [social enquiry report]. The social enquiry report, we find out what happened and the child committed that crime? Is it through peer group influence? Is that kind of attitude from the parents that he has copied from? Or it is just by accident that the child has committed the crime? And we will recommend and put

the person on probation and monitor as well. We visit the family, counsel the child, as the child is on probation, mostly for 6months...

M: **so that is the care home?**

R: yes! Care home is different from this probation. When they say probation, then there is a case. And when you are talking about care homes, we have different, different homes. We have children homes, we have orphanages. Children homes are different from orphanages. Orphanages they receive children who have lost both parents or one of them and then children home doesn't necessarily mean, when a child is in need of care and protection and supposed to be taken out of the community to be placed in a different environment, just temporarily, that is where we have the children home. And then we have the remand homes. And the remand homes also, they are for juvenile offenders. When they look at the case at it is that, the person has to be taken to a home. It is just like children's prison. Are you getting it?

M: **yes**

R: they don't go to the adult prison. I think we have senior girl's correctional home, we have senior boy's correctional homes, junior girl's correctional homes and junior boys correctional homes. They are all there. They are all homes for children to rehabilitate them. For instance, if there is child prostitution and they report the case to us and it gets to the court level, and then you see that the child is not trying to stop and other things, we recommend that the child should be taken from that environment so that she will not have access to that for some time. Pastors will be there, mallams will be there to be praying for this person and then the person will reform and they will bring her back to the community. I think they are all rehabilitation services.

M: **alright. What is your view on formal and informal child protection services available in this community?**

R: formal, I think I mentioned a lot of it. Let me go to the informal. We encourage informal child protection bill. Those days, the family system that we had, we all join hands to take care of children. You will sit down and realize that, a child is doing something bad. It is not your biological child but the child belongs to the whole community. You have to contribute your part to bring up the child. I don't know where you are coming from but today there is a medical doctor in Lamusi district here that is coming from your community, he will let everybody know that that doctor is coming from my community. Even he will tell other people that, that is my brother. But when the child is a defiant, even if the person is from your family, you don't want to mention that that child is from your family. That is why there is a need for the whole community, the whole family, the

whole district, the whole region and the nation at large to help raise the child. We have to all help when the child is in need of something. If the parents cannot provide and any other person can help, you help. I think communities used to contribute for children to go to the university. Somebody will have the admission and cannot get money, the parents cannot get money. The whole community does contribute. So we are bringing that system back. We cherish it and we are bringing it back. Let's team up and take care of the children. That is the informal aspect. But when you go to the formal aspect that is what I have just mentioned. The NGOs are coming in, the child protection policies and laws, juvenile act, act 560 they all help in a way protect the rights of children, this probation services we have just mentioned, a lot of things.

M: **so what type of case or case will you use formal system and what are those you would use informal system?**

R: well, there are some cases that actually you have to use the formal system. We are very careful here.

M: **the first one, may I know the formal one.**

R: yes, when the child is abused up to some level just like defilement cases and what and what. We don't even handle it in our department here. We do referral. You have to refer it to a court and the court will take its action. Maintenance cases like this, they have to report it in the office there; paternity cases but we try...all these cases you can try both the formal and informal system. People might have witnessed certain things, certain movements of the two parties.

M: **paternity cases.**

R: paternity cases, child custody cases

M: **and you said that one can be both.**

R: yes! But mostly you can't just say they should handle it over there. So the informal sector is the issue of school dropout, child delinquency. So such cases we join hands; because if I am the parent, now, today I am travelling to somewhere and the child decides not to go to school, other members of the family, they are there, they are not trying to intervene. So what happens? You cannot be there all the time. So we make sure that we bring families together and so that they will encourage and talk to the child to go to school. Sometimes, the school itself involves them, they are all informal collaborators.

M: **what about female elopement?**

R: yes, child elopement, we have dealt with some before. As I said you need to bring the formal and the informal sector together; because this child and family welfare policy that we run actually, we are encouraged to in a way bring both formal and informal together to do that. If you elope the child now, somebody has to...they will try over there. But when the child is attending school, when the person refused number of times and the person refused, definitely you have to report. When they report, we will also go to the community, we try to invite them and talk to them. Probably they don't know the consequences and what and what and what. We have that and about three girls as at now, they are in school. They eloped them to some communities and then, even though we had challenges with one of them, we tried a number of times and we were able to succeed.

M: on the part of the man or the girl?

R: the girl! Because when it is like that, you know the girl child's mind is not matured at a level that probably, most at times the man is matured than the girl; and you know these teenagers, when they are in love, trouble. They don't just go through the man, the way you persevere and now the person is used to be…it becomes difficult sometimes for the man to even separate the girl from himself.

M: so it means you are still having a case struggling with.

R: yes! But just for the past three months but it is solved now.

M: is she pregnant?

R: no; and some of them too they just force. They take the person forcefully there and then they sit them down and then...

M: alright. Can you tell me a case you use the formal system to resolve? What was the outcome?

R: yes, there is this case of a man and he was actually with a lady...

M: we are talking of children...

R: children are involved. For us when there is any case and children are involved, it is a case that concerns us.

M: alright.

R: so this man came down here to look for job and used another woman just to call the first wife and tell her that "we are no more so please pack your things". They have three

children. So they came actually and I called the whole family and then actually they told me that they have struggled with the case in the chief's palace a number of times. But when the lady came down, the man picked the children forcefully to the new wife and then asked the lady to go away. They tried solving it in Fiamochi palace and then they couldn't succeed. They reported the case to my office. I sat the young man down, talked to him and then he told me that, he is a Christian and cannot marry two wives. He has to divorce this person and marry the other one. I tried, tried, tried and I said okay, you can go. We talked about maintenance and the amount that he was supposed to pay was also too much, he can't pay that amount so I referred the case to Nandom court. When the man came to court, he denied in front of us that he didn't say he doesn't want the lady, that the lady is his wife and he doesn't have any problem with the wife.

M: so it means the outcome was good?

R: it was good.

M: we also want to know more about another case that your outfit adopted informal scheme to resolve.

R: yes, I think one of them was a child abuse case. A teacher from the community called. I went, I tried and then if I went there with police, I may not achieve my goal; for them seeing the uniform, it might be a different thing altogether. So I picked my motor bike got to the community and I went to the chief straight. We invited the teacher, he came around and then he even narrated the whole thing. It is like the child's biological father was the one she was staying with. The mother's first husband is a brother to the current man so just like inheritance; you are my brother, you died, I married your wife and I gave birth to a child, then this gentleman sitting down is also our younger brother. So after giving birth to the child, I neglected you my late brother's wife, then he said he will not sit down there and allow me to just neglect our late brother's wife and the children and they will be suffering so he also came in and also ended up by impregnating the same woman and also neglected the woman. So now, when I realized he impregnated that same woman, and they are brothers, I became annoyed and I picked the one that have given birth to forcefully to a community I have settled and I was there with my new wife and the child and they were actually maltreating this child to an extent that, the man broke one of the hands and then the woman too broke one. So they use their local ways to tie both hands and the child was just like about 5years or so. So they will lock this child inside the room and go to work. They go to farm. So this child was able to slip out and then went to the head teacher's farm where he also met a group of students during holidays to assist him. So they brought food and they were eating, so the guy came and then he was begging for food. So the teacher called me because I do go to

schools, basic schools to even do my sensitization so my numbers are with them. So they called me, I went there, when I went, I went to the chief's palace, we invited the head teacher and he narrated the story. So myself, I asked the head teacher not to come with us. The assemblyman, the unit committee members, I moved with them to the farm settlement. The place is isolated. Even if they are killing you, nobody can save you. So we went there and then we asked the wife; the wife said is like the husband had the hint and he run away. Then I asked of the child and she said the child is not there. I told the assembly people that the child is inside. And when you look at the way they build the local homes, you will enter the first room, is it the hall; then the bedroom is also there and another room, very dark. Not knowing that is where they always hid this child, he doesn't even see the sunshine. So I entered. I didn't see the child in the first place. The assemblyman by then was convinced that the child was not there and I said the child is there. I entered the second, the third; I used my phones light and saw the child. When I brought him out, it is like he was going to die instantly. Very, very, very weak so I asked the woman, "you said this child is not there", and the woman was just there shivering and I said, "no, I am not going to do anything to you but let's send the child to the hospital". She prepared, we took the child to Lamusi here and then we sent the child to the hospital and I just allowed them to jail the step mother small before the brother came and then bailed the step mother. But when we picked the child, we went to the chief's palace again and the chief said he will make sure that...the man is not from his community, the man has to go back to his community. So with the help of the chief, we were able to send the child - I think 3 months or so I called the mother from down south. I think the mother run to Techiman for kayayoo, so I called her, she came. So when they heard about it, many family members from the mother's side they were coming but I was also very careful that a wrong person will come and pick the child and we will not know. So when they come I don't give the child until the mother came from down south, took care of the child, I assisted, took care of the child for 3 months and then they discharged the child and then they went home. I followed up a number of times and I think the child is doing well.

M:

03/05/2019

R: Good evening.

M: Please can you describe to me the general work of your institution?

R: We take the lead in integrating the vulnerable, the disadvantage and the excluded in the mainstream of development at the district level. We do justice administration, community care, adult education and many more. So we work with people in their communities to make sure that the vulnerable is mainstream is integrated into the development agenda.

M: Ok. Please what about your role, you personally, what is your role as director?

R: Well, I have officers under me that report to me. Then I also report to the DCE through the coordinating director. I make the input into the composite budget of the district assembly and like I said, I take critical decision on the vulnerable and anything on government policies concerning gender, women, children and then those who are not privileged in the community of society we support them. Then we also do adult education.

M: Please what about in relation to children and.... what role does your department play?

R: I think I just mentioned that anything concerning children we are responsible. For example, abuse like child marriage, element, child neglect and the rest, we are responsible if there is any case. Even if the child is in conflict with the law we have to go to the court and defend the child. So that the child is not mixe3d with adults in the cells or prison. So anything concerning children, even if you send them to the cell or prison, he will be referred to our office.

M: Alright, please can you take about some of the services you provide to children, care givers and children?

R: We provide many services. If a father is not responsible, and he is brought, you know a mother too can be irresponsible but you know but mostly it is the men that are brought to our office so we make sure that we make them responsible.

M: How do you do that?

R: For example, normally, it happens when there is divorce or separation, then the woman is supposed to meet the man, so when that is not done, they come to our office and we are able to

calculate based on the man’s income, the portion that he is supposed to give to the woman every month and we are even to compel them to do that.

M: What about counselling for dropout students? Do you offer services like that?

R: You know we do guidance and we do counselling and when I say we do guidance and we do counselling normally guidance is done in a group or a focus group discussion or mass interaction. Like you have a bigger crowd, so you tell them generally the dangers that a person or a group of people can face or the dangers that they are exposed to and how they can face it. But when you come to counselling that one is one on one, that one you cannot do group counselling. Even though maybe there is a term you can use but when it is group, it is guidance but when it is one on one that one it is counselling. So that one it means the person has a peculiar problem and you have to deal with that one specifically and that one there must be some confidentiality... so...

M: So can we go on?

R: Yes.

M: What about rehabilitation of abused children?

R: We do it but we dont have a rehabilitation center.

M: In the district?

R: No.

M: Ok.

R: What we do is that we have child protection committees in some communities and then when a child is abused, we have care givers in the communities that the child is supposed to be taken from the situation that the child finds himself or herself to the care giver. Normally the care giver is somebody who is trusted or who has a track record of good behaviour and respect in the community. So the child is taken to the care giver temporarily before the case is reported to us and then we go into the case. The reason is that we are not physically present in all the communities and some of these issues can happen at midnight, weekends, so that should be the firts point of call before we come inside.

M: Ok. What about counselling for violent parents?

R: Yeah, we do.

M: How do you do that?

R: When we hear the issue or the issue is reported to us. We invite them through a letter. When the person comes, we schedule a date with the person. When we finish the first session we schedule another date for the person, until such a time that you see that the person is getting ok, then we can stop.

M: So do you sometimes prosecute offending parents?

R: We do. But that is the last resort. Normally when they come, unless it is a criminal case, we want to resolve it. For example, if it is a paternity case, the man is trying not to accept the pregnancy that one we try as much as possible to let him accept it and then we end it there. But if he is not trying to accept it then it means you have to take the person through all the processes. And you are likely to end up even doing a DNA to confirm. But if the person accepts, as much as possible we want to make sure that the person accepts and then you will just end it there and then see how best the woman can cope with life if she delivers, is she going back to school, what work is she doing and so on.

M: You do all that?

R: Yes.

M: What about stigmatization of children with disabilities?

R: That one we do general education. Mass education in the community.

M: Alright, so based on your experience working in this community, can you tell me about your observation on how issues concerning adults and child are resolved, or parent's adults or child-child relationship in this community?

R: Like what kind of relationship? I dont understand.

M: What I mean is that you have been in this community for some time. So how have you observed the relationship between children and adults?

R: Well it is... if you make a generalisation, some parents gave a good relationship with their children. But majority will like to take decision for their children. So normally children are not involved in decision making but we also have few cases where children are involved in decision making.

M: Can you give an example?

R: An example is if you want to buy a Christmas or Salah dress for your child, some parents will ask the child, what do you want for Christmas? What kind of dress or what shoe do you want for Christmas, but some will just buy for them.

M: So do you think the relationship is cordial or hostile?

R: That is why I said we have cases where the relationship is cordial. Then we also have some cases that are not cordial. But we have more cases that are not cordial that ones that are cordial.

M: Now what interventions do you have in streamlining or aligning parents/ guardian relationships? Especially those that is inconsistent with child welfare environments.

R: We have a district child protection committee that is responsible for child welfare. When there are issues of child abuse, they sit on those cases. Then when you go down to our communities, we also have community child protection committees. In some of the communities, we are still expanding. The community child protection committees are also to respond to abuse cases that involves children at the community level. So that before they are reported to us. So like I said, because we are not present in all the communities, we try to put up those things so that the people can immediately report those cases to them before they come to us.

M: Ok. Wat about interventions for parents guardian child relationships?

R: I think it is the same thing because of you talk of parents or guardian child relationship, a guardian may be a person who has not biologically given birth to a child but taken custody of the child so, once these systems are there, they take care of all kinds of issues.

M: The second one is talking or parents or guardian who have children with disabilities.

R: Yeah, when there are issues we intervene, when there are no issues we do general sensitization.

M: Alright, how do you access the type of coordination that exist amongst child and adolescent related institutions?

R: Well from where I sit there is a cordial relationship, there is no problem at all. However there maybe information gab. Access to information, some people are not aware of certain things and some people see it and think it is some big place. But from where I sit, our doors are open and when they come we tell them but when we get any opportunity to meet them we tell them that our doors are open so if you have any issues you can come.

M: So like the department of social welfare, ministry of gender and social protection, DOVSU, is there information sharing between you? Are there times you come together, to talk about...

R: Mostly unless an NGO brings us together. Apart from that, there are instances where DOVSU comes, we dont have DOVSU in the district in the first place but there is DOVSU in the region and sometimes they bring their activities, like sensitization and other things to the district and when they are coming that one they pass through us. Most of those activities are supported by NGOs.

M: NGOs like?

R: UNICEF, Plan Ghana...

M: So does that mean you have clearly defined roles and tasks?

R: Yes.

M: So the social welfare has its roles and tasks? DOVSU same...

R: Yes.

M: Ministry of gender, same...?

R: Yes.

M: Alright, what about reporting?

R: You know I said earlier that I report to the DCE through the coordinating director.

M: Ok.

R: And copy my regional director.

M: Ok.

R: Or my regional directors.

M: Alright, can you tell me something about the core issues in monitoring or supervising children related organisations in terms of compliance to national or international regulations and rules?

R: What happen is that this time we have many NGOs that are supposed to register with us. But some of them dont register with us.

M: Why?

R: Well, there are various reasons why they dont register. Some are doing illegal things. So they dont want you to know about their operations. Some too I dont know whether they dont know that they are supposed to register with us. Others too think that the procedure they have to pass through is difficult. Because some will come and tell you they want to register, when you just give them a list of items they have to bring, and then they will tell you it is difficult. Sometimes I ask, what is difficult in all these things because the things are not... I dont see any difficulty in all the things that we ask them to bring.

M: Ok.

R: So there are various reasons why we dont want them to register. If you want I will just tell you want they need to bring for us to register them. You just need a certificate of incorporation and commencement of business, just a photocopy. You know when you are operating as an NGO you are supposed to register with the registered general so they will give you a certificate of incorporation and commencement of business. So you just give us three copies of that, photo copies, then you give us your constitution, I dont see these things to be difficultly then you give us a brochure of your organisation, and what we expect to see in the profile or brochure is your vision, mission, objectives, goals, and program areas, board of directors, staff and any other useful information. Then when you give that to us, we will conduct a social investigation about your NGO and give you a report. Then we will also give you a form to fill, that one we call it profile form, then that is all, you just write an application, then address it to me or the director, then your application should have the logo of your NGO. Like your letter head, attached to these things.

M: So are these some of the issues that makes it difficult for you to monitor or supervise?

R: Where I am dragging to is that because some of them are not registered with us and most of them are also doing projects in relation to that, definitely we will not be able to monitor them.

M: Any other thing you want to talk about?

R: Yeah... and you know some of them too, they dont want us to collaborate because they dont want us to see inside.

M: What do you mean by the dont want you to see inside?

R: Like some of the things they are doing they dont want to share with you. So ....

M: Why do you think they dont want to share?

R: So like I Said some of them have dubious things they do, so they dont want to open up to us.

M: So, in this community, what makes children unsafe?

R: Well I am dealing with the district.

M: So in the district what makes children unsafe?

R: In some cases, elopement. Then we also have few cases of female genital mutilation. Then the last one is child marriage or early marriage.

M: Ok. So how do you handle these things?

R: As for child marriage and female genital mutilation and development, as for elopement, when we get you we stop it and we rescue the child. Then we do sensitization from time to time.

M: Can you please tell me the rate of the child marriage, the elopement a the female genital mutilations?

R: The female genital mutilations are about 3%, child marriage is around 25%, and elopement is also around 2%.

M: Now apart from these issues, can you also talk about your perspectives on rehabilitation services for children in this district?

R: When you say perspective, what exactly do you mean?

M: Do you have rehabilitation services for children in the district?

R: We have rehabilitation services, but we dont have a rehabilitation center.

M: In the district, but do you offer those services?

R: Yes.

M: How do you do that?

R: You know when we have pregnancy issues, and what we do is that the girl, we interact and council the girl to know whether she will like to go back to school or she will like to learn a trade. I even have a picture where a case we reported to us and then the girl said she doesn't want to go back to school.

M: Ok, did you ask why?

R: Definitely we investigated it and we realised that even if we send her back to school, it will not yield any results, because what we even did was that we assign her to the guidance and counselling coordinator of the GES. Because she was in school, so we bought this sewing machine and the accessories. This is the assembly woman. So we handed it over to the assembly woman to be handed over to the girl to go and learn and I think this is rehabilitation.

M: Ok.

R: So if she wanted to go back to school, we would have allowed her. The father wanted the money we took from the man. But we said no, we will not give him the money.

M: So you took money from the man who got her pregnant?

R: Yes, to buy these things.

M: What about access to therapist?

R: You know we have different types of therapy, so I dont know. Mostly, if you need any therapy, you have to go to the hospital. For example if it is stress, or depression, we cannot give any.... well we will work on it but sometimes the health people have some drugs that they will give, apart from the counselling that they will give. And then like I said we have natural therapies and then these scientific or maybe machine or drugs therapy, I dont know the term to use. But the therapy we give is counselling.

M: Do you have a care home in the district?

R: No.

M: Ok, what about probation services?

R: Yeah, we render probation services, even though we dont have a court, they are now building a court for us.

M: So how do you render those services?

R: When there is a case in Wa and it involves our district, we come to Wa to render the probation services.

M: Ok, let me ask this before we go on. Do children with disabilities feel safe in this community?

R: From where I sit, I dont see what is making them unsafe.

M: Ok, so they feel safe?

R: Yes.

M: Alright, what is your view on formal or informal child protection services available in this district?

R: It is like sometimes we are repeating the questions?

M: Is that how you feel?

R: Because as for the formal child protection, you know I told you we have a district child protection committee, I mean that is the formal ones. The office of the social welfare and community development is there. Apart from that we have a child protection committee which we are part of. Because we selected people from traditional rulers. We selected somebody from GES, health, to form that child protection, when you go down, we also have community child

protection teams. Then the informal ones, the family system is there, the extended family system is there so that is why I am saying maybe you are repeating.....

M: I am sorry if you feel that way. What type of cases would you use the formal systems and which one will you use the informal systems? Can you give us an example of a case where you use the formal system to solve and a case where you use the informal system?

R: All the cases you can use the formal system and you can use the informal system depending on the gravity of the case. For example if a husband have a problem. Sometimes the issues they raise are minor issues but it is husband and wife issue so you can refer them to go back to their home and resolve it/. But some of them too, they need some technical advice, I dont know if I have made myself clear, there was an issue that was brought to my office, and the things the woman was raising, I dont see them to be any serious issues. They are just some petty problems. The husband's father is doing that, is doing that... he goes to drink and comes to make noise, you see those things you have to handle them in the house.

M: So that is informal?

R: That is informal. But there are also some issues...

M: Like? Can you give me an example?

R: Like there was an issue like the man is not responsible and he works in a different place, so it is like he is running away from the woman and because he works somewhere he has got advantage. So when he goes there even weekends, we don't come back. So such a case, you will need to know what a woman is doing that is sacking him from the house and then you will also need t investigate whether there is something there that is also keeping him from going home. The local people may not be able to do that kind of critical investigation of the case so that you talk to the woman and talk to the man.

M: Alright, thank you very much sir, we are done with the interview is there any other thing you want to add?

R: No.

M: Alright, thank you very much.

Region
District Name
Community Name

Urban/Rural
Gender
Date of Interview
Interviewer name
Transcriber name
Interview length (minutes)

M: Can you please describe to me the general work of your institution.

R: Now it is social welfare and community development, the departments have been merged so basically what we do is in three areas, we have community care then we have child right promotion and protection then we also have what is called justice and administration. The other aspect has to do with providing residential accommodation for the vulnerable; those abandoned and those neglected then we also work with civil societies, NGO's who are into the areas we operate. Then we come to working with persons with disability, the other aspect too has to do with the aged in the society. We facilitate the livelihood employment programme to reduce poverty by the government so basically our core mandate works around we seeing to the wellbeing of the disadvantage in the society that is the vulnerable, orphans and children then the aged then the PWD's then also we come to the children, seeing to the wellbeing of children in the district. So basically that is our core mandate.

M: So under community care what precisely is it that you do?

R: That is why I said the support we give to the aged and the Persons With Disability (PWD).

M: So what kind of support is it that you provide?

R: With the aged that is why we do that through the LEAP payment and that is a government policy which we at the office facilitate when it comes to making sure that they get what they need to receive because it is a cash transfer so when it comes to payment we have to be on the field to pay them. And with the PWD, the government has a percentage of the common fund they use to support them so when those funds come we at the office see to making sure that we get tools, equipment for them to at least empower them.

M: So I heard you say you work with person with disability as well providing support for them.

R: Yes

M: What are the supports you provide at the moment?

R: At the moment what we do is that the support is not just one stop, we have that of education, PWD's who are in school who are needy, the office through what is called reporting and also going to the field to verify recommend those people for assembly to pay their fees that is right up to tertiary. Then we also have medical, if they want to want to change their clippers they have to get some assertive devices, the office has to facilitate that based on the request. That is why the district has been able to register them and we have an album and we use that to assess. And we also come to working tools, those who have learnt some skill like those who are into hairdressing, those who are into tailoring, we get them the equipment, some of them they just need the equipment to start so we provide those things for them and when we see that your condition is beyond you working or doing something then we have to at least give you something for the family to use to take care of you.

M: So what is your role in this institution?

R: My role is to facilitate all those activities.

M: Your role as?

R: The social welfare is to facilitate all these activities because we are the desk officers so if you are the desk officer you have to see to all these things because you are the middle man between them and the management. So our core mandate is to see to all these things, collaborate with other agencies to ensure that our people at least get a minimum level of sustainability and being empowered.

M: So in ration to children and adolescent, what roles that your department play?

R: When it comes to children in this area, we are to protect every child in the district, that is what we need to do so anything that will go in a way to affect the wellbeing of the child, the office has the responsibility to come in and also see to getting it resolved with the children because we have 0 – 5 years, 0 – 12 years depending the kind of situation.

M: So we are looking at it from 0 – 19.

R: Yes they fall within our mandate.

M: Can you tell me some of the services you provide to parents or care givers of children and then children?

R: With the children that is why I said it depends on the area you find yourself and the kind of issues that confront children.

M: So let's look at it to this district.

R: When you come to this district, since the district was carved out from Central Tongu in 2012, most of the problems here is child neglect.

M: Why are you saying so?

R: Because if you look at the people, the father and the mother, the lady will conceive and give birth and that is the end, the father just forgets about having even a child somewhere so it is either child neglect or single parenting that is the major issue that is why under the justice and administration we normally form the child panel whereby we look into the issues when the parents come to report but most of the cases is on child maintenance, the father not able to maintain the child because with the children's act we use here we have a point whereby it is the responsibility of the father to at least perform his parental role so when you are not doing that we have to call you to order to make sure you do it and that is the major issue here. The others which is child labour is not common even though it is being practiced but those interventions we had from "IWERM", we had from international needs and we also had from Justice mission, with the education and with the programmes we rolled out it has reduced as compared to those times this is relating to child labour and child trafficking. For child abandoning it is not common, it is once a while that we hear someone has abandoned a child somewhere and has left and we have to come in and provide residential accommodation for the child.

M: You did mention of child neglect and as well single parenting, so with these things you have mentioned, in the area of child neglect what service do you provide for the children or the parent and looking at single parenting what services do you also provide?

R: With the single parenting and the neglect, when we talk of the single parenting it means that at least one of the parent is alive or is with the child that is why I say when we look at the situation through the support that we have at the office that is the LEAP and the fund that comes for the PWD, in a way when we assess the situation and we see that this person doesn't fall in any of the category at least we can get some support from there to give to the parent who is alive to take care of the child. But with the neglect we make sure we follow up and get to the root of when the neglected started from and sometimes what happens is that we are able to trace and even get to know where the parents of those children are and if the social welfare office calls them they come and

they say it is true so they will perform their role and we at the office also make sure you do those things we assign you to do. Some of them will say they don't have any livelihood or they are not doing anything so how can they take care of the child but since you gave birth it means you are somebody who is responsible. We do all those things that is why parents who normally want to joke with the law and don't care we sometimes hand them to the police or going to court we take them there so that if you are imprison because of not taking care of your child you will learn a lesson because the office cannot have a fund that when you come and it is child neglect and you can't take care of the child we give you money because we are looking at the long term and sustainability and you know the government policy on child protection also came out with that policy that children must even grow with their parents so we in the office here we don't bring that barrier but we rather integrate and make sure there is emotional stability in the families. So the support we render as I told you through the programmes we rolled out those times were community engagement, we selected some communities which I can say for now we were able to do 15 of them that is depending on the programme we wanted to roll out so through the sensitization we made them to be aware of their roles and responsibilities to their children because most of them didn't know. When they even come and you start using the children's act and mentioning them to them they will say "I know when I give birth I give birth" how the child will grow, how the child will eat and the rest they don't mind but through that some of them I can say those that we have been successful the feedback we get is that they are now aware of what they need to do to the children so now the neglect, the maintenance and the single parenting is now reducing. Those time the numbers were large because they will come here in their numbers.

M: So what is the numbers now?

R: We rate it every quarter but if we come the last quarter of last year and the first quarter of this years, because last year we were able to administer 19 of the cases on this neglect because when they come and they start the story it is included but this quarter all those that came the 19 that we had is has gone down to 14. So the parenting aspect as we said about single parenting what we do that is what I said we don't have the money, all that we can do is to sensitize, is to advocate, talk to parents and we do that at the social services level too the subcommittee because we have women and children in that subcommittee. And even this year, before the year will end we are rolling out a programme on good parenting whereby we want to sensitize them on how you can be a good parent, so those are the interventions we can undertake because we don't have the money to give to them.

M: So in the area of violence prevention, rehabilitation of abuses children, what are the services you are providing?

R: With violence, here I can say that it is not common when it comes to the children but when it comes to the adults it is common so what we normally do is we collaborate with the police but we don't have "DOVVSU" here because I remember anytime there is any case relating to DOVVSU we have to refer to "Sogakope" or "Akuse" because we don't have that service here. So normally when those issues come because we cannot handle those issues we only tell the parties "go to the station" so upon getting there and they call us we confirm the issue then they will also refer because we don't have that power but if the violence is around the house or in the home like somebody came here and said her husband used a cooking utensil to hit her head and there was blood gushing out, we called the man and surprisingly the wife thought the man will not come but the man came and when we asked him, he said it was because of anger and we said what he did was an assault so we are sending him to the police because we are like the people who provide that soft word services or programmes because the law restrict us on where we can get to so those are the things we normally do for those victims.

M: So what about stigmatization of children with disabilities, what services do you provide?

R: That one is also one of the areas which we are still doing our possible best to orient the parents to accept the child as they are. Some of them see it as a burden but when you go round, that is during the registration, we went round and we saw some of the children with their parents, when they heard that social welfare was coming to give them support then they know that now they can lean on something to take care of the children because for most of them it is very difficult. You can go to a family and 3 of the children are paralysed but no wheelchair so how will the mother carry all of them when she is going somewhere so when you see those critical conditions, quickly we even recommend to management that let's get this for them and most of them are now doing well because at least we don't only give you the assertive device we also give you a support to start doing something like those with Intellectual Disability (ID), they can't do anything and their parents are there with them, we provide them with equipment.

M: Equipment like?

R: Some of them will tell you they want to start selling pure water, ice cream so such people we ask them what they have and they can say they need an ice-chest and a deep freezer and we provide it, we buy those things for them from their fund and it is yielding results, now they don't come worrying us with application that they need GH₵1,000.00

to take care of their children and how long will GHȻ1,000.00 last. I don't know if you heard this but last year we did those distributions and this year by the end of May or early June if all things go well, the next batch will begin, some of them their parents are seamstress so we get them industrial machines so that when people sew they will get money because we want a flow of income whereby you will not come and be begging or come and be asking, if the equipment is there and you are using it at least what comes from there can be used to take care of the child. And now when we gave them those kind of sensitization, most of them now are saying that this is better than the money we were giving them.

M: And they were saying that themselves?

R: Yes because they will tell you that if they make ice cream and put it in and go and sell they will they will make money but it you give them GhȻ500.00, GhȻ700.00 what will that money do? So now that the directive came from government that we should buy equipment for them or do this for them as I said it is far better because people are using it and they are getting the results so those are the things we do for those people.

M: So based on your experience working in this community please tell me about your observations on how parent/adult-child relationship is in this community?

R: In this community as I stated earlier on, when you look at their relationship with their children it is not cordial because they have that notion that when a child is born it is the mother who should take care of the child so it becomes a burden for the women especially if they are not doing anything. Could you believe that one man will impregnate 4 people, give birth with all of them and now will not be taking care of them but still be giving birth so when the children are also growing they grow with that mentality.

M: So why don't the men take care of the children?

R: They will tell you they are not doing any work which is not true because look at the area at least you can get something to do in a day that is why I said when they come here, the way we treat them now it is making them aware that parenting is not only for the mother, it is for the two parties who came together, the father and the mother because the child must grow with them.

M: So how do you create this awareness?

R: Through the programmes we rolled out those period and those that didn't hear, when they are found culpable and they bring them here, we inculcate it in them. it is this week

that we are not administering cases, if you were to come next week you will come and see the office packed because people have come and we have to administer so now that irresponsibility on the part of the men is reducing because the women were suffering. That is why I said now we want those things to reduce to the barest minimum, that is why we are still doing the advocacy, we are still doing the roll out of those programmes even though some of them have ended we are still using the toolkit to carry out those programmes so it will reduce but not now because still some of them are proving stubborn.

M: Stubborn in which way?

R: They don't want to even mind, when you call them to even come they don't want to come so we just hand them to the police and we are free. We want that relationship to be there because the children grow by observation, whatever they see from the beginning will be an impression in their mind so this area what I even observe also is that because some of the men refuse to be responsible when they children grow and they are well to do they don't care about their fathers, they care for only their mothers, why because they will tell you it is their mother who was always with them taking care of them because their fathers left them. Here too most of them who are staying here are staying with their grandmothers because if mother is trying and is not able to make ends meet she may go to Accra or go to Afram plains to go and work there and there too someone will see her, she will give birth and now it ends. So most of the children if you ask them where are their parents they will tell you they are over bank, they are Afram plains, they are at Gemeni and in town I am sure you might have heard most of them.

M: So when they go to those other communities what precisely do they go to do?

R: They go to work.

M: What kind of work?

R: Some of them fishing, some of them mining so when they are there the woman cannot stay there like that because someone who has given birth before, giving birth again is not a problem so when they came again they will come with another child for another man and it is common. Those are the issues, so when the children now grow, already when they were children they were on their own so that they are grown won't they also be on their own so when he gets a motor bike and is riding he picks one girl at the back and that is his girlfriend and before you realise JHS 1 or class 6 they give birth and how will they take care of the child. So irresponsible parenting brings about those immature parents and now they also give birth and that is how the chain is because when you go

into it you will realise the child didn't even stay with the father so now that he/she is on his/her own he/she can do whatever so that is the chain and it is common.

M: So what interventions do you have in aligning parent-child relationships that are inconsistent with child welfare?

R: As we identify those problems, we have at the community level child protection committees which we have formed and we use the key stakeholders in those communities to form that committee so that when there is an issue and you have been called to the headman you will know that you have been found culpable because we saw that we can't be at every place at the same time and when you train the people and they are there as your watch dog committee, they will now be giving you information as to what is peculiar. So through the community child protection committee, we also formed child right clubs in schools, so those clubs in schools also teach the children to know their rights, to know their roles and to know their responsibilities so that if in the home something is happening they can report to the committee and the committee will also report to the department then we will quickly make the follow up. Some of them are saying that is making the children to be stubborn.

M: So who are the stakeholders who constitute this committee you are talking about at the community level?

R: Most of the time when we go we look at the headman in that area and sometimes the Queen mother because of the women group and we get unit committee members and we also get opinion leaders, those that the community respects, we don't put anybody there. Through the committee we form volunteer groups, people who will volunteer to make sure that children are being protected even though they won't be paid. So they roll out the programme, some meet on Saturday or Sundays which we partake in their meetings. We have women groups who meet, the men will also meet and then the youth will also meet. We do all those things so that if everybody is aware of his/her roles and responsibilities it will not be a problem to us because at the community level people must be there to ensure these things are being carried out. So normally we do that because of sustainability because we will not be here always, we will be transferred, we will be sent to other districts but what we normally do is that when we are being sent the people are there and they too that is their community and when they go they will come back to those areas and we will just monitor through the quarterly meetings we have with them to know the challenges and the successes and it is yielding results.

M: So what results have you seen so far?

R: That is why I said there is a reduction in the number of cases.

M: You mentioned that these committee members are trained at the community level.

R: Yes.

M: And you also mentioned the formation of clubs in schools.

R: Yes.

M: Now I want to know, for the clubs that are also formed in the schools who coordinates those clubs?

R: One of the teachers.

M: Are the teachers trained in that regard?

R: They are being trained so that is why I said through the intervention we got from those NGO's, when the teachers are being trained, they now become the facilitators in the school, the children too some of them are being trained, international needs for instance, we took some of the brilliant children and those who can advocate to their training centre and they were trained so when they came they came to also tell their friends and that is what we do because if the training is just for training sake and the people don't work with the tools, it will just die off and that has been the problem with most of the programs. Because we didn't foresee that in the near future this programme is for 2 years and it will fade out so what are the things we will put in place so that when it even fades out, it will still be continued but through these things, when those things are in place even though it has faded out the people are still there. So normally those are the things we do.

M: So what interventions do you also have in place in aligning parent and child relationships who have children with disabilities that are inconsistent with child welfare environment?

R: That is you are talking of parent child relationship.

M: Yes. Especially parents who have children with disabilities that are inconsistent with child welfare environment?

R: That is why I said those parents, when we identify them we recommend what can be done for those parents.

M: But are there some interventions for such parents whose relationship with their child are inconsistent with child welfare environment?

R: One of the parents, the child is blind so when it happened like that she didn't know what to do because now it is going to be a burden, she has to be the guide, those issues are critical issues so we identify those parents, go to them, sit them down and now make them aware that even though the child's eye is not seeing, the other senses are working so don't think that child is going to be useless in life, it depends on the training. so recently one of the training we had which we are implementing is to train those parents on how they can even take care of those children who are visually impaired, how they can direct them in the house, how they can lead them here and there and how they can even take care of them because most of them will say it is a problem and some of them say it is a curse so that child should be sent somewhere and it will not be a burden to them again but we tell them it is their child because people are blind and now some of them are lawyers, some of them are able to do this social work and they will now be asking you that is it true that that child can become somebody in future and we tell them yes. Because now if you are able to train them, they can do things on their own without you the parent being there to even assist them and here too we have one of special school at the "three teens" and one too at "Adidome" where the blind unit is which when the child grows to a point and we see that the child being with the parent is not good we send them there which that one when we send them there the little support we have at the office we are able to get them the kits and their children are doing well. Some of them are even in SHS and some of them trough that have completed JHS and they are in SHS so it is not only a burden for the mother but the department also shares in that burden.

M: So you mentioned training for parents whose children are blind, what of other disabilities, anything in place for them?

R: For now, we can't do all at a go, we are starting from somewhere.

M: So your starting point, you're currently starting with those who have vision problem.

R: Yes.

M: So why are you starting from one to the other and not having a specific…..

R: For the others we do it but it is not as a training.

M: How do you it?

R: With the others like those who have difficulty in moving, those who have difficulty in hearing, you see the problem we identified is that when those children are in those categories, the parents don't accept those children.

M: So how do you align their relationship to your welfare environment?

R: That is why we talk to them, we don't sit in the office and talk to them, we go to them because some of the assembly men will call us and say they have seen a child with her mother at some place and the way they are treating that person in the house, officer it is better you come and talk to them. So when they see us there and how we are able to inculcate into their mind that these are children who can do one thing or the other, some of them when they get the idea they now change and they are able to do some things.

M: So how you assess the type of coordination that exist among child and adolescent related institution?

R: Based on our quarterly report, the visit we go to have with them and also the community around what they tell us because the people are living in an area whereby there are a lot of people around them and they see whatever is happening to those children, so when we go for monitoring we are able to get those figures and even some of them when we go the condition we find the child prior to when we talk to the parent now when you go it will be different, some of them will say "he is around so let me go and carry him and bring him to you people to see".

M: Now I want to know how you assess the type of coordination that exist among you and then other child related institutions like DOVVSU, Ministry of Gender and Social Protection, the Ministry of Education, all institutions that have something to do with children within this bracket we are talking about, 0 – 19 years, how do you see your coordination?

R: I can say for that one it is positive. Positive in the sense that we collaborate, we don't go for monitoring alone, that is why I said we have the child protection committee which all these agencies are in so like the girl child unit of the GES, when we go to commission for Human Rights (CHRAJ), they are also part, NCCE, they are also part then we have the police, we have DOVVSU and the others so when we come into those brackets we are able to collaborate because I give them information, they also give information, they will report "social welfare there is this issue here, can you go and look at it" then if I also have a problem with a girl in a school whereby the girl is not able to do one thing or the other or the girl is going through challenges here and there, I can tell Girl child, "Girl child I went to this community and there is one student there with challenges", and

when it comes to Rights, CHRAJ is also there so we work hand in hand. And that is why any time any of them have the opportunity to go to any community, they also go and say the same message about children because when we meet as a committee and we discuss we discuss about things, that is why I said with the subcommittees we all find ourselves in the various committees whereby we are able to bring issues concerning children that is 0 – 19 years because they are the age bracket. So the relationship with them with the parents is what is cordial because some of the issues concerning the disabled children, it is the special education officer in the district who feeds us with that information so when we also carry out the support to them he also goes there to see and brings us feedback that when he went they are doing well or this is the state or that is the state so we don't do it alone, we do it with all these agencies.

M: So how do you assess the coordination in terms of clearly defined roles and tasks?

R: Sometimes when it comes to the fund to do some of the work that is where the problem is but we manage to sacrifice and do our part because you can't get the funds at a go so for my team that I work with at least when duty calls they are up to the task even though they will tell you it is going to be a problem but they will tell you the officer they will go and bring you feedback which in a way is helping us because we are here for the children and it is our work so they all do their task as assigned that is where I will end, if I give it to girl child she also carries it down and where girl child will end, if it is about rights, child abuse, we bring CHRAJ in then they come and see to it then the report comes back so it is cordial, we all talk to ourselves, we all share ideas, we all bring out things that will help to promote the wellbeing of North-Tongu children.

M: So though you all come together, you share ideas and all of that, what is the frequency of your meetings with all these other institutions?

R: You see you don't get all of them at any time you want to meet them so whereby it social welfare, girl child, CHRAJ, we will go and see to that, it is not that we all coming in at a go to go and solve the issue no but when the task requires that somebody should specifically carry it out, that person is tasked to do it but whereby the person needs the support of others that one too we coordinate but on a whole at least in a year we are supposed to meet every quarter to review.

M: All the institutions?

R: Yes.

M: You are supposed to be meeting every quarter?

R: Yes.

M: But as it is you don't meet?

R: We don't meet.

M: What are the reasons?

R: That is why I said everybody and the work schedule because we were supposed to meet the last time, you call this one and he is on another activity, this person too is on another activity, that is why we always make sure the core group we meet every time when there is the need, like the special education officer for PWD, for him we meet him every quarter, the department meets him every quarter because he is basically for those children in schools so if you don't meet him at every point for him to tell us what is happening, you can't make progress, even yesterday he was here to come and find out what is the issue now about the fees of those children who are going next week. So for him he is at the GES so every quarter we meet him because any issue that is coming from there passes through him.

M: So could you tell me something about the core issues in monitoring and supervising child related organisations in terms of compliance to national regulations and rules.

R: That is those who come and grant us interventions. When you come as an agency into children you already know that we have laws of the land which your activities must conform, your objectives must conform to those laws and regulations so when they come, normally we look at their registration first, whether it has been registered because when it is registered they will have the certificate of registration to come to the district and the district also makes sure you also register because the laws of the land states that you don't just come and say you want to carry out an activity, you must register so when they do the registration now we know their objective, their operational areas so if we are monitoring, we are monitoring looking at the operational areas that they said they are going to engage in. So some of them when issues are coming out at the committee they will tell the committee members they are not in for that so go to the social welfare and discuss it with them, they will tell you theirs is up to this point because the laws must direct them as to what they need to do.

M: So aside that are there support issues in monitoring compliances or supervising?

R: For those people we have engaged or those who have come to work here and the time of their project expiring I can say most of them we didn't have any problem with compliance but it is only that some of the activities is being done by them and when

they leave the people don't know them it is we they know; do you see where the issue is? Because when you go to the community and you create the impression that you are coming to do it, when you leave they will assume you have left with it but when you include the office so that you will empower the people there to do it with them, they know that this office will always be here, that is why we always advocate and tell those agencies that when they are coming, do the activity through the people here so that when you leave the people there can resolve the issues. Because when you hear of child trafficking and you want to come and see to it that the child that is trafficked is being integrated in the family, you can't do it on your own, you can't go and catch the child and bring him and say this is your mother and this is your father so I leave you to them so I am going. The office must be part so that they will now do the back ground study about how the child even migrated from where he is to where he got to, what was the problem, what were the challenges here and there.

M: Please describe what makes children unsafe in this community?

R: When they are not with their parents they are unsafe, anything can happen to them and the kind of hazards they are being exposed to.

M: What kind of hazards? We are talking about this community so what kind of hazards are the children exposed to in this community?

R: In this community if you go to around, those cattle herd, even though we are still telling them they have not eliminated them, those children you will see some of them and they are walking with the cattle for distance it is pathetic.

M: Why are you saying it is pathetic?

R: If you see a 7-year-old child and is following herds of cattle and you see the appearance it will not tell you the child is exposed to hazard so those things we don't encourage it because as they are in the bush anything can happen to them.

M: So what of economic factors?

R: Yes those who engage in trading like today being Friday you will see a small girl let's say between 7 and 8 years selling by the road side, anything can happen, look at these riders moving here and there.

M: So are you saying it is unsafe because the child is selling and there are so many riders in town?

R: No it is unsafe for the child because at that time the child is not mature to how to cope with that situation because somebody at the other side of the road may call “come let me buy something” because you want the person to buy if you are crossing the road and you are not able to notice the oncoming vehicle it can knock you.

M: So does that mean we have many of the younger children who are selling?

R: They are not many, they are only a few.

M: So what of the social aspect, as to the kind of relations that are common in the community and how it makes children unsafe.

R: With the social aspect I can say it is these bar operators because when there are occasions they can play music to midnight and you go there and the people you will see there are teenagers. That is why often the assembly is doing everything possible to ban those things.

M: The assembly is doing everything possible to ban?

R: Some of them they have stopped them from doing that aspect.

M: And what are the reasons for banning them?

R: Because at that time...[interviewer interrupts]

M: Please at what time?

R: Midnight, between 10 and 12, at least the child should be under the care of the mother because if you don’t stop it there it is then that teenage pregnancy will come in because they are having fun with boys and that is why we took steps to stop those things.

M: What were the steps you took in stopping it?

R: At the committee level as I said social services we recommended and those areas that we saw that there were drinking spots, we had to invite the vendors and talk to them and with the noise level environmental also came in to give them the level at which they can operate and these children too we are coming with bylaws. Even though the bylaws are there, we are enforcing those bylaws because we want the children of North-Tongu to be people who are educated and tomorrow they can help the district but if those things are there, socially even though it is good in a way but if it is too much it will affect their performance, so when they committee recommended to management they invited some of them here and through engagement they told them they have to stop and now when you walk in town at least it is not eliminated but it is minimal and the parents that

we are sensitizing to make them know the value of education because most of them didn't go to school. Now even those who are persons living with disability, most of them are now going to school because they know they can do something.

M: So what cultural factors also make the children unsafe in this community?

R: In this community what I see is the cultural factors you could attribute it to the relationship between the father and mother because most of them when you talk of proper marriage it is not there. Most of them it is because in the beginning they didn't come together well.

M: They didn't come together well in which way?

R: It is not proper marriage so it in a way affects the children.

M: I still want to understand you saying it is not proper marriage.

R: You see what happens normally and what we have observed is they are just staying together, families are not away they are staying together so when pregnancy comes in and they give birth, now families can even say they don't even know the man because customarily the man didn't come to perform anything on the woman so the child is not for the man so most of the children bare the name of their mothers. It is common which when you ask you will know.

M: So this factors make it unsafe for children?

R: Yes because from the onset already the child is now having that mind that this what happened so now I grow to let's say 15, 16, 17, I don't know where to go. I want to go to my mother and she is at her boyfriend's place, I want to go to my father and he is at his girlfriend's place so now how can the two parents monitor the child because they are not together, some of them will even tell their child that when he/she goes to his/her father's place he/she shouldn't call her because she doesn't want to have anything to do with him. And when they grow like that, culturally if you look at all the problems you are having, that is the root cause because if there is proper marriage at least if the two parties are not there, the families will take care of the children because the children grow with the environment. So culturally those things in a way contribute because here we don't have any practice whereby they align children to some sort of practice and say that if you are girl this and if you are a boy that, here we don't have those things.

M: So do children with disability feel safe in this community?

R: Yes they feel safe.

M: Please why are you saying so?

R: They feel safe in the sense that we have orient the parents to know how to stay with those children and with the disability federation most them too are parents who are disabled so when they see other children they are not hostile to them, we have included them into the society, we let them voice out for us to know what we can do to help them so now they are even feeling proud.

M: And who those feeling proud?

R: The persons with disability because now they can go to every place. When we had the occasion here and we called them to come and they were displaying they are being recognised. When you include them they will also know what to do and every time we are meeting we call them so if you are a parent and you come with your child and you see other children performing, you don't neglect that child.

M: So what are some of the performances they put up?

R: That is what I said through the training they get in school such that when we have programme we will roll out a play, so now we have included them and not disintegrated them, now they are part of us and the office has made it friendly that anybody can walk in to ask for information and the relationship we have with them makes them feel that they are part of the community.

M: What of the community members themselves, how do they treat them?

R: Most of the community leaders some of them are living with disability, some of them are visually impaired but they are opinion leaders in the community so at that point they fight for their children. That is why one time we were forming some committee and the chairman of that committee in one of the communities is a visually impaired person so when the people came from Accra to monitor they were surprised and we said the community said he should be their chairman. Other people are there but they said he should be their chairman, what is the signal, it is telling you that they recognise that he is also important and he said that he is surprised that people are there who can see and they didn't pick them but they said he should be and he is performing so now it has flowed to the children. The children are not neglected like previously. Previously you will see most of them just walking at the market and be displaying but since you came you yesterday did you see anybody?

M: No I haven't.

R: Like you will see them because most of them are in school if not here then Adidome so now we have included them seriously. They are our priority.

M: Please tell me your perspectives on rehabilitation services for children in this district?

R: Here we don't have a rehab centre but we have some in Accra and we have some of our children there.

M: You have some where?

R: Accra.

M: In Accra.

R: Accra rehab, where the psychiatric school is.

M: But not in this district?

R: Not in this district, we even have a special school which in a way we can call it a rehab because we keep them there and they are being taught so here we have that facility but what I can say is that area they have challenges but they are managing. So the children we send there, some of them when they are being stabilized.

M: So you take these children to the rehab in Accra?

R: Some of them not all of them.

M: And what happens to the others?

R: That is why I said we look at your level of stability because here they will give you medication, they will try to stabilize you so if some of them we see that were they have gotten to they can learn a skill, Accra they train them in those things so we are able to recommend that this person can join them.

M: So were talking about your perspectives on rehabilitation services for children in the district.

R: That was what I was saying that we have a special school so when we stabilize and see that they can do one activity or the other, we are able to send them to where they can be trained. Here it is a school so when we keep them and they get to a level whereby some of them can be engaged in one skill or the other, we send them to where they can be developed which the office facilitates because some of them are needy and we have to provide support so we do that.

M: So what is your perspective on access to therapy or therapist for children in the district?

R: Here we have a hospital that is the Batour Catholic hospital so we send some of them there for treatment and the hospital unit has a section for psychiatric which we work with that person. so for they getting medication is not far from away because of the Batour Catholic hospital.

M: But are there therapist for them?

R: Some of them are being given medication in school because the teachers go to training so if they see a kind of problem which is beyond their ability then they send some of them to the hospital or they inform us or the appropriate quarters so we can also take them there but to tell you that they have somebody who is there catering for them for that one those are the challenges they have.

M: You mentioned that the teachers are taken through training.

R: They are taking through training on how to take care of the children.

M: Who provides these training services for the teachers?

R: Some of them are under GES, so GES special education unit.

M: The special education unit of GES trains the teachers.

R: Because they are special teachers.

M: So what is your view on formal and informal child protection services available in the community?

R: With the formal ones those are the ones we earlier discussed when I mentioned that we form those committees at the community level but before that the community leaders and the opinion leaders also had their own way of protecting the children which one of them in one of the communities is that the chief made a bylaw that no child of school going age should be seen engaging in one activity or the other. So when you go to his residence he was even taking care of most of the children.

M: No child of school going age….

R: Should be seen in carrying out anything related to child labour, going to herd cattle, going to sell when it is school hours. He inculcated into them that education was his priority so even before we went there to roll out this programme the leaders were

aware of doing that and when they hear of any issue in relation to child abuse or child neglect, because they are the community leaders they are able to trace where those parents are. Let me add that the work we do here we don't do it in isolation with the chiefs and elders in the community, when we administer the issues about child protection, most of the time we make reference to the leaders there, the opinion leaders, the assembly man, the unit committee chairman, the chief, so that whatever we say here they will also be aware to do the monitoring for us because most of the time before the case will come to us it has passed through them so when they are not able to solve it then now it comes to our level. So with the formal and informal, I can say the formal one is ahead of the informal.

M: I will like to know why you are saying the formal is ahead, any reasons?

R: Because the informal one, most of the clients, those who are engaged in that activity because of family relations at the community level, let me give you a typical example, there was a case on rape and the issue came to us so quickly we have to send the child to the hospital for examination before we call on DOVVSU to send the report to them. so the elders sent somebody to come to the office to come and talk to us so that the case will be given to them so that they solve it because they are family and I told them that no this one has to do with the law, since the person is found culpable the law must deal with the person so the parent of the victim said no they will not agree that it goes to the elders for them to solve it but the office must solve it for them so they take priority in the office solving it for them rather than the community leaders because of the relationship some of them will have with the culprits. That is why I said we have rolled out those programmes to formulize it that even though we have you there but you are not breaking the structure but when it is beyond your level refer it because they collaborate with us to get those issues and sometimes too to even get a victim they will tell you how you can even get them. that is why I have said that the formal one since the time we came here and we instituted, that one is yielding results rather than the informal one because if you don't hear you can't act. But when we carried out the education, the people now inform us directly before the chief will now call that it has come to their attention so we tell them we will collaborate with them to solve it not they solving it alone, so when they even solve it at the community level a report is sent to the office so that we monitor.

M: And how does the office monitor from them?

R: We go to the field to the people.

M: You go to the victims?

R: Yes the victims, we go, we don't stay, sometimes we don't have the logistics but we go, personally I go to even look for children, when a case is reported that they are staying somewhere and they are being abused I go in a manner to go and find out. Last week I was in town to go and look for a child because the mother run away with the child because the father says he wants to come for the child customarily and the mother heard it and left town, left the child somewhere, I went to where the child is and we were surprised to go and see the child there, I had to get a foster parent, somebody the child should stay with whiles we do the investigation then the mother called me that she has come to town and I told her the child is at a place in good condition and she said she wanted to see the child and I said she should wait till we are done. So we go to the field.

M: Which type of cases will you use the formal system in addressing?

R: Those ones boils down to law.

M: I will like to know the type of cases, specific cases.

R: Cases that come in the area of custody. The law specifies that you look at the best interest of the child when it comes to custody so that one it is not that we just sit there and say anything, we have to use the children's act to really get those things and even in the area of maintenance we normally give them the maintenance cover, the monthly payment which should be given to the child.

M: So aside child custody which other cases will you use formal systems in addressing?

R: If it is child abuse because we have the human trafficking act so any one which involves trafficking the law states it clearly so if you are found culpable we send you to the police.

M: Which cases will you use the informal structure?

R: The informal structure when they come here, sometimes it is just disagreement between the parents, like when it comes to married couples, some of them will come here and will say the woman is doing this or that, some of them will even come and say the woman doesn't respect in the house so we should talk to the woman so such things you can formalise it you have to call them because our mandate also is to provide emotional stability in families.

M: And how do you do that?

R: Like when the man brings the issue that the wife doesn't respect, the children are insulting him, they are always fighting with him here and there, we call all those families

to come to the office then through engagement, hearing from the children and the children will tell you that their father doesn't take care of them that is why they are behaving like that toward him and the mother too will say the man too doesn't give them money to feed in the house that is why she is also behaving like that so we bring all of them together and talk to the parties then after that assign that everybody should perform his role and responsibility. So when they do that and they go back and we follow up most of the time they even call us and say that if they had not brought the case here they wouldn't have known that things can become better in the house like that because an office has come in and they respect that office and whatever we say. So the informal they are so many and most of them are not structured.

M: Can we talk about some of the informal cases aside misunderstanding?

R: Sometimes too a child will bring a parent here that they asked him to herd cattle and after 3 years they will give him one cow and after the 3 years he has not gotten it so if that is the case he will leave the parents and go and stay somewhere because he is not having any asset so we will call the father and the mother and the father will say it is true that made that promise so now that he is of age they will honour it and sometimes they will even take us there to go and see the one that they have given to the child and we tell that that they should take care of it so that when he is going to school in future that cow will be sold.

M: So do you just solve it here or you refer them to the informal structures in the community?

R: When we attempt settlement and we don't get results we refer to the informal structure.

M: When you attempt settlement…

R: And we can't come to a conclusion, so when they go back and the chiefs, the elders also sit and they resolve it and they conclude, they now bring us report likewise we also give them report so it is two-way. So marriage issues and the rest in the area of who the child should stay with as I said some of them is not formal because it is not proper marriage most of them engage in so if there is some misunderstanding we come into it so those are some of the things.

M: So can you tell us a specific case you used the formal system to solve or resolve?

R: that is why I told you there was one of the case it even went to the police, it was a child custody case. The man was from Nkawkaw, they were having 2 children, a boy and a

girl, but the boy is with the father and the girl was staying with the stepmother so through the treatment the stepmother was meting to the girl, the girl went to school went to school one day and before they realise somebody came for the girl from the school, a motor rider went to the school immediately they close, just took the girl because the mother described the girl to the rider and the name so it was a Friday like this and they had closed from school then the child was sent to another community so quickly the stepmother reported the issue to us here, gave us the number of the biological mother of the child so I called and when I called she said the child was with her, so I aksed why she will go for the child when the child was in school so we went to the police.

M: At what age was the child?

R: The child is 12 years old. So we went to the police and the police called her and she brought the child so between Friday night to Monday morning the child was with the commander because the commander said until the social welfare officer makes the report the child will not be realised to any of the parties. So now the man came from Nkawkaw and had to stay in town till Monday. Then Monday morning I had to go and investigate, I went to the child personally to sit with her and ask all that is happening because it shouldn't be in the presence of the parents, it wasn't a small case, the man wanted to beat the former "wife".

M: So what was the outcome?

R: At the end of the day we concluded that looking at the welfare principle we have to look at the best interest of the child and the child's biological mother is alive, you can't tell me that biological mother will do something bad to her own child and the father too you have married another woman and we did an investigation at where she was staying formally and we got to know that the treatment wasn't good but the child was just managing so later on we wrote in our report and ordered that the child should be with the mother for the time being while we still carried out further investigation, come and look at how the man behaved that day, we nearly sent him to DOVVSU that Monday and luckily for him when they went back to the station he said all that the office has said he is going to go by it but he will not say anything but one day the child will still come back to him. It was a very difficult case but at the end of the day the law states it clearly so we gave them split custody. The child should be with the mother but the father should be given access to the child at any time he wants and also if the child wants to go to the father she can go on condition so now everyone became afraid because as the mother, if the child comes and stays with you and you maltreat her what will she do, she will report to the office because we told them since they cannot solve their issue and

the office has come in, the office is going to help them to solve it. Now if the father wants to get the child after vacation he has to call the office and the office has to say she can go and the mother too when it is time for school to resume the father must make sure he brings the child to the mother for the child to continue school. So the law is there but if you don't know it you will end up saying it is not there.

M: You mentioned that the child wasn't treated properly, what treatment were they giving the child?

R: The stepmother was beating her mercilessly.

M: What other treatments were given to the child?

R: The child is in one community and the distance from where she is to the school is so far and she gives her only GhȻ1.00, if you even take motor you pay GhȻ2.00 so the child walks to school and walks back. I know the community the child was coming from, I have been with them and I know how they are so when I got there I knew that this is what is happening so based on our recommendation the child is now with the mother and every time we go there to find out how she is doing even though she is with her biological mother.

M: Can you also talk about other cases that you used the informal structure and the outcome at the end?

R: I mentioned.

M: We want other cases.

R: Relating to children?

M: Within our bracket 0 – 19 years.

R: That is why I said the cattle issues are common, we wait when the children report then we see to that. The cases are many and I can't remember every one of them and in different groups. A man came to report that his children are not respecting him, he had 7 children and we called all of them to the office and at that time the man was building this mud house and the children said they won't help the man so we talked to them to respect their father and their mother and to go to farm and do minor works. They went back and they themselves called us that now there is peace so the father he called on the children and they went to cut the thatch because it was their own room which was leaking but they said they won't help their father but now when it was repaired they are inside and it wasn't any difficult case.

M: So during your investigation what was the reasons for which the children refused to help their father?

R: Because the man went to marry another woman and is taking care of that woman and the children and has neglected them so when they also heard it they said since he is able to live with that woman and those children with the other woman they are small and cannot do that work but they are grown up, the oldest was 21, then 15, 16 so the man came that they should make peace so that they will go and help him and they went to do that. So we are just here to integrate families we don't disintegrate families because we will leave and the families will remain.

M: With all this discussion we have had are there some other things you will like to say?

R: In relation to the children?

M: From the discussion, where we started till now, are there certain things you will like to add?

R: What I want to add is the programmes when it is being rolled out there should be follow ups.

M: The interventions?

R: The interventions because if it is just being done and we leave it like that we won't get the results because some of them are 2 years, 5 years, after that 5 years you can't get the results we need at 5 years, it may come after 10, 15 years so we are advocating that since our partners are into child welfare and development, the interventions should continue coming because change takes time and the resources because most of them are being done but because of logistical challenge you can't go everywhere so at least when the programs are coming they should include at least those components. Some of the programmes have it.

M: The logistics?

R: Yes but not all because sustainability, for mobility to those communities, to those areas, like as you come here it is not only Batour you will get the problems, the over bank is where the real issues are but you see now it is only this area you can talk to them or you will be going to over bank?

M: We've gone there already.

R: You will see that the issues are there. Here is a town so we advocate that more of the interventions should come, as government we are doing our part but come and support us, UNICEF should come and support us.

M: You mentioned that some of the programmes have logistics component.

R: Yes.

M: And other do not.

R: Yes.

M: So with those that don't have the logistics component, how are you people able to carry it out?

R: That is why I said since it is our mandate, whether they give us or they don't give us we have to do it but if they give us it will in a way be more efficient.

M: Please do you have other things to add up to our conversation?

R: This is the second time UNICEF is here to do this recording, this is my second time, generally it is good so since we have outlined those issues, I know when you go back you will work with them for us so that tomorrow we will have a better district. We are doing well but with your support we will become better. But those are the issues as we have discussed and we will also do our best to make sure that the children are well protected.

M:

Ok, so how many are you in the family?

R: we are four.

M: So how many of your children are males?

R: With those here right now, there is no male.

M: So you mean they are all females?

R: yes!

M: Can you tell us of their ages?

R: 7 and 4yrs old

M: Right now, the child we want to talk about is Theresa. So can you tell us something small about only Theresa to us?

R: ever since I gave birth to her, there is been nothing wrong.

M: Not necessarily that but the changes she has gone through over the years.

R: ok, so it is only her current state she is in now.

M: I want to ask again that, what are the major things that you have seen in her as she was growing up? What I'm trying to say is that, if a child gets to a certain age, he or she is expected to do some things at that age. So I want ask whether you see your daughter doing things of her age or not?

R: I haven't seen any problem!

M: I'm not asking of problems here. I'm asking that, what are the things that you see her doing most of the times?

R: she is now growing so I'm yet to see what she can do.

M: But even if she is one year you can still see things from her speech and other things. So what are the things you see her do at her age?

R: she can do everything all by herself—she plays with her friends, she eat her food on her own.

M: So what did you notice about her as she started crawling?

R: she crawled very well!

M: How many months old did you she start crawling?

R: she was six months old when she started crawling?

M: Did it mean to you that she has started crawling early or she has delayed?

R: that was a bit early!

M: Can she prompt you anytime she wants to visit the toilet or whenever she is hungry?

R: yes! She tells me everything.

M: How is she when she is playing with her friends? How does she relate with her friends?

R: that's also good!

M: **What are the things that you wish she could have been doing but it seems it has delayed?**

R: she has been doing everything I have always wished for her to do by herself.

M: **So this is the exact way you wanted her to grow up?**

R: yes!

M: **Looking at her age, don't you think there is anything that has delayed in her life?**

R: with her age, her eyes are only her problem.

M: **So you mean apart from her eyes everything is ok with her?**

R: yes!

M: **So is she able to go out and play on her own?**

R: yes! She goes out alone to play!

M: **How is your relationship with her?**

R: we are good. We have a very nice relationship and we play and do everything together.

M: **So apart from playing with her, what other things do you do with her that make you think you have a good relationship with her?**

R: I do send her to buy some things for me but it's within a very short distance. She is still very young and she can't go far alone so I don't send her when the distance is long.

M: **What again proves that you have a good relationship with her?**

R: we live good, wherever I send her she goes and we are fine.

M: **How do your children relate with other children?**

R: the way they play with the children in this house that is the same way they play with children outside. They feel very happy when they go out to play to the extent that they sometimes don't want to come home.

M: **Do your husband's young nieces and nephews or your young nieces and nephew come to stay with you at some times?**

R: my husband's nieces and nephews are not here.

M: **What about your sister's children?**

R: my children play with them. They either play in this house or play in one of their friend's house.

M: **Who do you think Theresa feels comfortable to be with?**

R: my elder sister. When I'm not around and she goes to my elder sister, she doesn't want to go to anyone.

M: **Why do you think it's your elder sister she feels comfortable around?**

R: my sister treat children very nice. That's why children feels comfortable around her.

M: **Can you tell me some of the things she does for them?**

R: what I can say is that when I'm not around, she will tidy up the place and prepare them for school. She does everything for them very well so it has now made them like her more.

M: **Where do your children get their playing stuffs?**

R: they get empty cans from our neighbor to play with.

M: **What about the times they want to learn? What materials do they use to learn?**

R: the children decide the times they want to learn. One among them will be the teacher and teach the rest of them.

M: **Where do they decide to go and play?**

R: they usually play around here…

M: **Where exactly are you talking about?**

R: in front of the palace

M: **Where again?**

R: they can go to one of their friend's house.

M: **Who would you prefer to seek advice from as you take care of Theresa? Concerning her upbringing and her health, who would first seek advice from?**

R: there is this one old woman I took advice from concerning my children's upbringing. She gave answers to me anytime I went to her with questions bordering my mind.

M: **Who was she to you?**

R: nothing!

M: **Was she living in this town?**

R: yes please!

M: **So in the absence of the woman, who would you go and seek advice from?**

R: I'm now taking my time to find another old woman who can give me advice on how to bring up my children. These old women are experienced in the upbringing of children.

M: **Now that the woman is no more, I want to know why you chose her as your advisor.**

R: the woman liked me very much and I also loved her. So I had that rapport with her and I easily went to her for advice.

M: **Can you tell me any special thing done for disabled children in the community?**

R: disabled children are been taken good care of. They are not troubled but instead, we show them love and accept them as they are.

M: **I'm not actually referring to one person though. I'm talking about a large number of people in the community.**

R: there is nothing like that here.

M: **So how do society see disabled children?**

R: I don't see society doing anything special for them?

M: **So society discriminate against disabled children? Do they do that to your children?**

R: no please!

M: **So they accept everyone with his or her challenges and see everybody as the same?**

R: yes please!

M: **Please can you tell us how Theresa's eyes became like this? Is it from birth or she was contacted this sickness whiles growing up?**

R: her sickness wasn't from birth. I realized it as she was growing.

M: **What is Theresa's exact problem?**

R: her problem is with her eyes. When the wind gets to her eyes she begin to blink.

M: So how do you notice again that she's having problems with her eyes?

R: so when the wind blows to her eyes, she blinks a lot.

M: Ok, continue with how you realize the sickness with her.

R: I notice her sickness as she was growing.

M: At what age did you did you realize that she is having this kind of problem?

R: she was two years when I saw it. She was taken to Asamankese and a white man took care of her

M: At Asamankese, is it hospital she was taken to?

R: yes! So over there, the white doctor told me that this is no problem. I was given some medicine to treat it.

M: So the doctor said it's no problem?

R: yes! Because I don't understand English, he told one Ghanaian doctor in Twi to tell me that.

M: Is any of your family members with this kind of sickness?

R: no!

M: What about your husband's family members?

R: It's one person I know.

M: I can see that your 7year old child is also with this sickness

R: yes!

M: So with your four children, two of them are with this sickness?

R: yes!

M: What are the things you do to help or assist them in whatever they do?

R: I cook for them because they are not of age to be preparing food for themselves.

M: So If she wants to go out, do you help her walk?

R: she does that on her own. They have been walking outside themselves.

M: Tell us what you wish to do for them but due to certain things you cannot do it?

R: I'm facing financial problem..

M: Apart from financial problems, what other things do you wish to do for them at home but you cannot do it?

R: apart from that I do everything for them.

M: So what are the things you don't do for them which is as a result of financial issues?

R: my finances are not good but I do well to do everything for them.

M: Have they started schooling?

R: yes!

M: Has Theresa started schooling?

R: yes please!

M: What are your challenges in bringing up disabled children?

R: I have not faced any challenges though but I do pray to God to do everything ask him to do for me.

M: Have you had any special training in the upbringing of your children?

R: no!

M: You mean no one has taught you how to care for a child with eye problem?

R: some old people have giving me some tips on how to handle them.

M: How many times has she reported to you that her friend are teasing her because of her problem with her eyes?

R: she has never told me such thing.

M: Have you warned the children here not to tease her?

R: none of the children her have teased my children before. It has never happened.

M: Have you assigned any household chores to Theresa?

R: No! But she baths on her own and sometimes when she feels like sweeping she does it. I don't stop her.

M: Even yesterday I saw her sweeping. Are sure you are not the one asking her to do it?

R: she actually watches everything I do. So when the place is dirty, she will just pick a broom and sweep.

M: Do you have any special way of correcting Theresa when she does the wrong thing?

R: I usually tell her to stop what she's doing but if she continues to do it, I just hit her slightly at her buttocks for her to stop.

M: Can you describe any of the days where you scolded her?

R: she misused all the water in our tanks. When I came home to see it, I had to beat her small. It wasn't anything that was meant to hurt her. This actually happened three days ago.

M: So what did she do after you have finished beating her?

R: she did nothing

M: Did she cry?

R: she cried for a while.

M: But why do you have to beat a disabled child?

R: the intension was not to hurt her but to correct her.

M: And why did you do that to her?

R: So that she doesn't repeat it again!

M: Have you had any special training on how to care for children with this kind of disability?

R: my mother ones gave me an advice on how to treat them. She actually told me to take it easy on them whenever they go wrong.

M: Apart from your mother who else?

R: there has not been anyone

M: So not even in the hospital?

R: It’s only one nurse at Agona Nsabaa who was also telling me to take good care of them because it is not easy to bring up these kind of children and that we should not worry them at all.

M: Which hospital did you meet this nurse?

R: I do remember the name of the hospital.

M: Is it a government hospital or an herbal hospital?

R: it is a very big hospital

M: In which way has the advice of the nurse helped you?

R: her advice has helped me in the sense that there are some actions I had wanted to take on them but whenever I remember what she said then I halt it.

M: Do the community help the children with special needs? Whether it’s from the Chief, MP, Assembly man or any agency?

R: ok, recently the Assembly woman did something for deformed people in the community.

M: What did she do for them?

R: they were given money. Some of them were given money to set up a business.

M: But your daughter didn’t get her share…

R: Yes! I didn’t take them there. And besides it was given to people who have extremely been maimed.

M: Would there have been a difference if your children were males?

R: I don’t think so. It would have been like this.

M: We would want to thank you for your time spent with us and accepting to be part of this interview. We are grateful! God bless you!

M: ok woman I really appreciate your time giving to me to have this conversation with you as I earlier said I want you to tell me some little about family how many are you in your family am not talking about your extended family

R: we are six (6)

M: you are six (6) how many males and females

R: Two females and four males

M: Please can you tell me something little about your child I don't know the child I so can you tell me what the child can do and what the child cannot do

R: Please anything concerning the child sickness

M: Yes something little about that just like you introducing the child something little and so kindly tell me a little about your child

R: My child (Eyaki) been the last born when I gave birth to him people in the neighbourhood were all admiring how beautiful the child was and people also complimented me for giving birth to beautiful and handsome children in the society and also people single the out to be special and so when the child got to forty (40) days and the child began to fall sick and the navel got swollen to the head and so I took the child to the hospital and they told me that they will have to do operation for the child and so when to operation was done and was left with two weeks to remove the bandage and the child starting coughing and I immediately took the child back to the hospital and upon getting there they informed me that the way the child is coughing they will have to remove the bandage and so they removed the bandage and so when we got home the child became very weak and dull and so I took the child to a herbalist and at the same time also was taking the child to the hospital and aside that also I added prayers to it by God`s grace the child can walk now and has even started schooling the only challenges

the child is having now is the speech and also the neck too is very weak. Eyaki is a child who is very hairy

M: Is ok we will get there I want to ask you a question what was some of the things that you notice that make the child very unique

R: My child is someone who is very knowledgably and also sober

M: Apart from that what else again

R: My child is also very neat and also likes learning that is whatever she sees she tries to force to read and write just that the speech is not coming

M: Ok let say for instance the child is trying to pronounce a word what do you do?

R: I say the word and the child will continue with the rest for example I will s and the child will continue to pronounce so

M: So the child cannot pronounce the word by herself

R: yes, please unless you pronounce it then the child will repeat after you or if the child picks the book on her own she points to the two letters words like the 'go and so"

M: Ok if you can recollect or remember what was the first word the child was able to pronounce

R: A lot of words like mother I want apple, give me money and I want this

M: Please tell me what are some of the thing you were expecting the child to do and as at now the child has not been able to do whiles growing

R: How to read and write

M: No please as a child is growing there are things you expect that the child can do at this of growth, like the child can do this and this as you see it has delayed?

R: how to bath and brush the teeth and when you put the paste on the brush the child tries to brush the teeth but not as very well like you want her to do

M: What else again

R: And also how to dress herself

M: And what else again as you made mention of her speech what about that one also?

R: Yea she cannot talk very well

M: So has that one also delayed?

R: Yes, please

M: And so what type of relationship does you and your child have?

R: Good one

M: You said good one and so what and what do you both do to warrant that as a good one

R: There are some mothers out there who maltreat their when they face such predicament in their lives especially they don't want the child to come closer to them but am not like that and she also one special child wherever you will send her she will go and

come and when she comes across something that I have left and forgot to cover it she will look for the lid and cover that so that It will not pour. Eyaki is very calm and I love her so much

M: And so please does your child relate to other child from your family and outside your family very well

R: Yes, please

M: How do you see that

R: Whenever my child sees any child around she forces to go and play with that child and moreover she shares whatever she is having equally among the other children and she doesn't mind to give hers to them and this has made the children to love and like her

M: And so which of your children does Eyaki relate with very well

R: The one she comes after they are both free to each and when you give Eyaki biscuit to eat and the one she comes after is not around Eyaki will always keep some for him or you have giving something to her with giving the brother`s own she will ask you were being my brother`s own

M: So your child is always with the one she comes after and how do you see that or why is it that she always wants to go with the one she comes after

R: Ok they both always do things together especially bathing and going to school together

M: Ok so please can you tell me the things they play with that are around you and also use it to learn

R: we use to have a game that they use to play and also our phones also

M: So can your child operate the phone

R: Yes, please and she can even operate the games on it or when someone calls you Eyaki can pick the call and bring it to you

M: So please apart from the phone and game what other local's things did they use to play

R: There is this wooden car that the play with and also they do make their own wooden car that the place it on the shoulders to play with

M: And so if they want to play where do they go to play?

R: we stay along the street and so they use the street to play

M: So apart from the street don't they go anywhere too

R: No please

M: Please who talk to me in case you need an advice or counselling regarding on how to care and cater for the health of your child who will you seek the help from first

R: A woman of God and she is the one I have being talking to when it comes to issues regarding my child. I do go to her place to complain to her and she tells me to keep on taking the child to the hospital

M: you said a woman of God and why her and not any other body

R: she is a person who has my child`s interest in her heart and she do sometimes come for her spend the holiday with her at her place

M: what are some of the norms and custom's you know about children with such disabilities

R: A little that I know is when you speak very ill about somebody that can happen to you and some also as I have giving birth and sickness came and met the child that can also cause that

M: Ok apart from you speaking ill about somebody don't you know any?

R: Maybe some have done something wrong or wrong a river that also do happen to them

M: how do the people in this town relate with such people in the society

R: In this our town everybody is minding their business and really do not care about others and some people can tell you this one is not a human being go and throw her or he away and people even told me also and I told them when the child was giving to me she was not like that and I have faith in God that my child will be very soon

M: You have already told me but I want you to repeat that did the sickness befall during birth or after birth

R: After birth that the sickness befalls on the child that was before the child got to forty days and the sickness attacked the child first starting from the swollen on the naval to the head and I went to the doctor who told me we need to do an operation for

the child and it was left with the two weeks for the bandage to be removed and the child got attacked with a severe cough and I took the child back to the hospital and then to inform us that they will have to remove the bandage if not the child`s life can be taken and so the stood on it and removed the bandage and so after that was

done about three day time and the neck became very weak and could not stand and so I took the child back to hospital and they gave us drugs and also gave me date to bring the child back to the hospital and so when the time was up I took the child back to the hospital and the child was admitted there for two weeks and so after the two weeks the head began to increase in size and the body to was also reducing and so the child was again admitted for another one week and a new doctor came and told us that he will discharge us home so that we will start administering herbal drugs on the child and so we were taking her to the hospital and also giving the child herbal drugs

M: And so you are administering herbals drugs to the child

R: Yes, please

M: please is there anybody in your family with such illness

R: hmmm in my family no please

M: Not from your father and mother side

R: No please and not even my husband house

M: Please can you tell me the things you have put in place in order to help your child the things you are doing well to help the child

R: Am so focus on my child`s schooling and I want to make sure that my child goes to school so she make use of her knowledge so she can become someone great in the future

M: Apart from school, am talking about the house what are the things you have putting in place to help the child and what are the things you do for the child

R: When we are in the house there is a slate that I hold the hand to write and also sings for the child and sometimes plays ludu with them at home

M: And please can you mention some of the things you would have loved to do for the child but because you don't have the strength so you can do and or you have a problem is anything like that?

R: yes please I wanted to employ a teacher at home for my child and also how my child can also become active like other children

M: Do you understand the question the things that you wanted to do for the child but because you don't have the strength or there is a problem that is what am talking about I hope you have seen that there are somethings that he cannot

do by herself if it is within your will you will help your child to do it but you don't have the knowledge these the things that I want you to tell me

R: that's why I said if I could get a teacher to be teaching my child for me to enhance her writing and reading and also the school that they use to attend was good for my child development but because of money issue I had to remove them from that school over there they were paying GH₵80.00 cedis per a term and so because of that

moreover to the feeding fee and pocket money when I add up I couldn't afford it and so I had to remove the child from there to another school

M: So you as a mother do you see this as a burden for having such a child

R: I don't see that a burden that I have gotten such a child because God has giving me the normal ones I did not complain why should this one be a burden

M: With such children you need those with special care to care for them maybe the child wants to eat or want to sleep and hitting the head again the wall has you experience that

R: No with my child if she wants to sleep she will go for the mat or mattress and sleeps on it and if she is hungry and am not at home she will go and take the money and go and buy food to eat

M: how many time has your child reported to you that people are making fun of her or abusing her

R: my child has never told me that unless someone has beating her

M: How do your child tell you when someone beat her?

R: If my child knows your name she will tell me by mentioning your name or if she doesn't know your name she will pull my shirt until we get to that person

M: In what ways have you put in place to make sure that they don't beat or make fun of your child

R: with the house we don't stay there much and with here also when we come they go to and comes back around 4:30pm and so when she comes, she will eat and then we go back home

M: Please kind you tell me the responsibilities that you have assign to her at home?

R: I have not assign any responsibility to my child

M: Your child doesn't do any work at home?

R: Yes, please unless I need something at home and then I will tell her to get me that

M: In what ways to your discipline and correct your child when she at fault?

R: I beat the hand when she is at fault to show her that what she did is wrong

M: Apart from you beating the hand what again we only don't use beating to discipline a child?

R: Or I warn my child not to repeat that again or else I will her

M: Do you recollect a day your child did something that you punish her something your child did in particular to warrant such punishment?

R: I went with my child one day to fetch water and when she was done eating she took the plate and fetch water with it in the big bowl containing water and I took her hand to the full water and beat the hand told her not to repeat such thing again

M: So why do you think it is necessary to punish a child with disabilities

R: if you don't punish the child he or she will not know what she did is not good and so that's why you need to punish the child to know is wrong to do that my child is someone who fear beating and so when she is doing something and you shout on her that I will beat you she stops

M: Please can you tell me some of the things that you have been taught to use it to take care of the child`s wellbeing

R: Yes we were taught at the hospital on how to care and cater for them and we were told that when it comes to handling them we must apply with caution because everything of there is slow and so when they are doing something that is not wrong and we want to correct them we shouldn't use beating but rather with love that you must do it like this not like this or we should be sacking or shouting on them when they want to approach you we must exercise patience in their dealings so that they will know what we want or like for us to also know what they like

M: So which are the people that taught you that

R: Room 10 and NTC

M: are they all in the hospital

R: Yes, please

M: So which the teaching that they are giving to you what can you really say about that

R: It has been of a great help to us

M: Why do you say that

R: The reason why I said that is because initially when she uses to eat she pours the drinking water into the food and I have taking my time to explain to her that we don't do that and she has stopped doing it and I also told her that if she stains her

dress with the food she will dirty the dress or she will be smelling and so because of that if she is about eating she will go in for the napkin and put it on the legs so the food particles and stain will fall on it

M: In what was as a mother has that really helped you

R: In my child`s eating habit and because of that I really monitor my child when she is eating and I do give her spoon or tell her how to eat and the way she should eat or

show her then use of napkin and other importance thing and this has improved her eat habit drastically and she can eat without soiling or staining her dress and body with food

M: Do you get help from the society or community for children with such disabilities

R: Yes there was a time ago and the education from their school called me that there is a council who want to support children with such disabilities and so we met them for the arrangement and so on the first day I was told to brief them on her condition

and they told me my child`s own is good and they don't know where to place her since then they have never called me again and I also didn't go there again

M: And so apart from the council you have never heard anything again?

R: yes please

M: what about the chiefs in this town?

R: No please

M: What about the minister or the assemblyman

R: No please

M: What about the hospital do you get special treatment or you follow the normal way

R: Yes we follow the no process

M: Please is there anything that I didn't ask you concerning the child or the things you are going that you want to share anything like that?

R: No please

M: I thank you for giving me the opportunity to talk to you.

TRANSCRIPTION SHEET

R: Okay the number of people I live here with?

M: Yes

R: Okay when my mother died, my mother's elder brothers child is who I stay here with but he also, he is the one sitting there, the one who asked us a question

M: Hmm I am talking about the people you stay in your room with

R: I stay in my room with my son

M: You stay with your son

R: Yes

M: And can you tell me something brief about your son,?

R: About what? I don't understand

M: Oh tell me something brief about the things he likes or doesn't like

R: Is it about food?

M: Yes the food he likes and those things, he is your son and you live with him

R: Yes

M: So say something small about him and let's hear you

R: Okay he likes everything

M: Meaning what?

R: Meaning he likes every food

M: So how old is?

R: 6 years

M: And where does he attend school?

R: He doesn't go to school

M: He doesn't go to school

R: Yes

M: And what food does XX like, what doesn't he like?

R: Please he likes rice and fufu but he doesn't like kenkey

M: He doesn't like kenkey

R: Yes

M: So what are the things that he can do and what can't he do?

R: Do you mean in terms of talking?

M: Everything, everything

R: Okay as I said, he is not that strong, he doesn't talk properly. When he was born there was something under his tongue and so his speech is not accurate and so he can't do anything. Because of this, I have not been able to enrol him in school. They said I should take him to the where the white people are and they will cut that part under the tongue and then I can enrol him in school so now, he can't speak well. When you speak to him he can hear you but he can't speak back to you, he can't call me Da, he can't say anything

M: And when you speak to him how many times do you have to speak before he hears you or in your mind you feel like he can hear you?

R: Yes

M: You feel like he can hear you

R: Yes

M: So what makes you think he can hear you?

R: When he is doing something and I tell him that if he doesn't stop I will beat him, he sits quietly

M: Okay

R: And if I tell him to come too he comes

M: Okay so how was he when he was growing up?

R: I don't like the way he speaks

M: Why are you saying you don't like his speech?

R: Because of the thing under this tongue he is unable to speak well

M: What about if he wants to use the toilet or do something, how is that? If he wants to go to the toilet is he able to go by himself?

R: When he removes his attire then he goes to the toilet

M: So does he tell you?

R: No, he doesn't tell me, he removes his clothes

M: He removes his clothes and what about bathing? Is he able to bath himself so does someone has to bath him?

R: I bath for him

M: **You bath for him**

R: Yes

M: **Okay and what about playing, does he play?**

R: Yes he plays nicely, he plays

M: **Where does he go to play?**

R: He plays right here in this house

M: **And why do you say he plays nice?**

R: Hmm okay erh…with the playing, you know we have playing and we have playing, he plays ball and the rest and sometimes he plays cars

M: **Okay please I am listening**

R: He plays with cars and he plays balls

M: **Okay so in which of the things he does would you say he is slow?**

R: Please I don't understand that part

M: **I asked you if XX is able to go to toilet on his own and you said no, you said you bath for him. In which of the activities would you say he is slow?**

R: When he removes his clothes and goes to toilet then he steps in the toilet and puts it on himself and all over his leg, I don't like that

M: **You don't like it**

R: Yes

M: **And what else?**

R: And he also urinates all over his clothes

M: **Okay so that is the only thing you don't like**

R: Yes

M: **So can you tell me the relationship between XX and the rest of the children here?**

R: As for that one unless the children's parents; I can't tell

M: **No, I mean how does XX relate with the children here? Maybe he doesn't play with them…he doesn't always stay at home, he goes out**

R: Yes

M: So when he goes out how does he play with the children?

R: I don't let him go far. If he is playing out he plays just here. He doesn't go to the station

M: Yes but does he walk alone or with the children?

R: He is with the children

M: And how do they relate?

R: He can't speak to them but if they are playing ball he plays with them

M: Okay so XX, who has the time to take care of him?

R: Please since I brought him from Breman I have been staying with him. No one else has time for him, I am staying with him

M: And why is it that only you have time for him and no one else?

R: Please here that we are staying, my mother is dead and my mother's brother that is here, this child is stubborn and so even if I am going to the farm and I leave him for him, he won't agree to watch over him so I take him everywhere. If I am going to the farm I go with him, if I am coming too I come with him. If you leave him, he will go on the street, if a car is coming he won't give way, he will stand there till the car knocks him so if I leave him, no one wants to watch him

M: And why is it that when the car is blowing the horn he doesn't give way?

R: He can't hear

M: He can't hear

R: Yes

M: So that means he is deaf as well

R: Yes

M: Okay so what items does he play with?

R: Sometimes he picks the car tyre or he picks the utensil and use it as drum and sometimes he pulls tyre

M: Why does he pull tyres and things? Why doesn't he pick a ball or something else to play with?

R: Sometimes he also plays ball and he also plays utensils as drum

M: So who do you go to if you want any advice on how to handle him?

R: I go to an elderly woman who is my mother's friend

M: And where does she stay?

R: She stays in Apam

M: She is in Apam

R: Yes, there is a place called Apam

M: So can you tell me the reason why you went to her and the advice she gave to you?

R: What she told me was that I should exercise patience and pray, I should also take him to chapel and tell the pastor to pray about it for me

M: So why did you go to your mother's friend and not to anyone else in this community?

R: Here I don't have any elderly person here that I can talk to about this issue

M: Why is that because you said you have stayed in this community since you were born? Why is it that you don't know anyone here that you can talk to?

R: Oh I know people here but it is not everyone that you can talk to for them to give you good counsel

M: Okay and do you have some cultural practices or traditional practices that you know about caring and raising up children like XX?

R: No there is nothing like that

M: And how do people in this community relate with XX? How do they see him?

R: They see that he is not strong

M: Is that the only thing? Aside knowing that he is not strong how do they relate to him?

R: Oh they just treat him as someone who is sick; they don't do anything to him

M: They don't do anything to him

R: Yes

M: So can you tell me a bit about XX's life when he was just born, when he was a baby.

R: When he was born, he was born here, he was very little and so when he was one month his grandmother came to take him to Breman and so I wouldn't know what happened to him. Only his grandmother would know

M: **And so at what point did you know that XX couldn't talk or that it took longer for him to understand what is said to him?**

R: With that, it is just when I went for him. I went for him about one month now and then I realised that when you speak to him, he doesn't hear

M: **So when he was growing up they didn't tell you that when you talk to him he doesn't hear**

R: No

M: **So I would like to know, in your family is there anybody like XX?**

R: No, my wife is from Breman; she is not from Agona, she is not from this community

M: **And what about your family?**

R: I am from here, my family is from here

M: **What I am asking is, XX's condition, do you have it in your family?**

R: No

M: **And is this condition in his mother's family?**

R: Yes they do have.

M: **Who in the family has this condition?**

R: My wife's' sisters child

M: **She is also like this**

R: Yes she is also like this

M: **And what are some of the things you do to take care of XX as he is?**

R: I don't know anything to him. All I do is to take him to prayers for God to change him for me so that he can speak fine.

M: **But about taking care of him at home, what do you do?**

R: At home when I wake up I bath for him then I clothe him and feed him. If his clothes are dirty then I wash them

M: **Okay so what are the things that you want to do for XX but you are unable to?**

R: Okay right now they said I should take him to the white people so that they can treat him, if it happens that they will operate him, they will operate him so that he will be able to speak again but I don't have the money to do so.

M: **And what are some of the things that you wish you knew about his condition that will enable you to stay with him well?**

R: If he was well, I would have been able to enrol him in a school

M: **But seeing that he is not well, what are some of the things that you wish you knew about his condition that will help you to take care of him better? What would you like to know?**

R: Please I don't know

M: **You don't know anything about it**

R: Hmm

M: **So staying with XX, how are you able to train him knowing that he can't talk well?**

[Respondent is silent]

M: **Is it difficult or it is not difficult?**

R: It is difficult

M: **And what makes it difficult? Can you tell me some of them?**

R: Okay his speech

M: **But we know he has speech difficulty but what makes it difficult?**

R: If he is speaking, the words don't come out well

M: **And that makes it difficult**

R: Yes

M: **And what will his speech do for you? As he doesn't hear…**

R: If he speaks well I would take him to school but because he doesn't speak well it is a worry to me

M: **Why is it a worry to you?**

R: Because he is at home and he follows me wherever I go and that is worrying

M: **That is what I am saying that apart from he following you to the farm what else?**

R: There is nothing else

M: **There is nothing else?**

R: Yes, just that he can't speak and the he goes to the farm with me is a worry

M: **Okay so that worries you. Does XX come and tell you that his friends make fun of him or laugh at him because of his condition? Let's talk about the community; do they make fun of him?**

R: Yes

M: **Okay what work does XX normally do at home?**

R: He doesn't do any work; just stubbornness

M: **So when he wakes up he doesn't do anything?**

R: No

M: **He doesn't wash bowls or anything?**

R: No, he is just stubborn at home

M: **He is just stubborn at home?**

R: Yes

M: **He doesn't wash bowls or anything?**

R: No

M: **Why doesn't he do anything?**

R: That's what I said. He himself is not strong, he is not strong and that is the reason why he doesn't do anything

M: **Why doesn't he do anything?**

R: He doesn't do anything because he doesn't have the strength and so he can't do anything

M: **Okay**

R: Only stubbornness

M: **Okay only stubbornness. So you have spoken about stubbornness. When he does something wrong what do you do?**

R: If he is doing something and I tell him to stop and he doesn't stop I beat him

M: **You beat him?**

R: Yes

M: **With what do you beat him?**

R: With a cane

M: With a cane?

R: Yes

M: And give me examples of some of the things he does that make you punish? What makes you beat him? Give me examples of some of the things he does that makes you beat him.

R: His stubbornness

M: Like what?

R: For instance, this knife he is holding can cut him so if I tell him to put it down and he doesn't listen to me

M: Then you beat him because of that

R: Yes

M: Oh okay then you beat him

R: Yes

M: And have you received any training on how best to take care of XX?

R: No

M: You haven't received any training

R: Yes

M: And I want to know also that in this community, do you get any support on how to take care of XX? Do they have anything that they do to support children like XX?

R: No, I do everything

M: You don't get any kind of help

R: No

M: Okay so I want to know, if XX wants to eat, how is his feeding like?

R: There is nothing with his feeding; I just give him what I have

M: So what do you have that you usually give to him?

R: Oh as for him, he will eat whatever you give to him; he doesn't reject any food

M: He doesn't reject any food

R: Yes

M: Okay please we have brought our discussion to an end. Thank you for having the

patience for this conversation.

END OF DISCUSSION

M: it's exactly 4:39 and in the household interviewing parents with child with disability and the interview is being accompanied. Please let me come close to you. Thank you for being part of this research.

R: Thank you!

M: I want you to tell me the state of your household—the number of people living in the house, how many rooms are here… Give me a picture of that.

How many children do you have?

R: Two!

M: You have two children! So together with yourself and your husband will be…?

R: Four!

M: What is the name of your children?

R: Florence Oduro and Albert.

M: Tell me something about Albert?

R: Albert was attack by an illness three months after I gave birth to him. It was like convulsion. He has been battling with it since and now he cannot walk again.

M: I want to know whether he was born with it or not?

R: I didn't see any sign of it right after giving birth to him but the sickness attacked him when he was three months old.

M: How did it get to him?

R: I bathed him and decided to go and throw the water away. I returned to see him very stiff on the bed. That was where I rushed him to the hospital.

M: So what did the doctors do when you got to the hospital?

R: When we got to the hospital the doctors attended to us and gave him some medicine. There wasn't any improvement so I took him to Koforidua, Kumasi—Gee, Accra, and Ankaful. Right now it's Ankaful we have gotten to. He has been taking all the medicines given to him but there is still no improvement.

M: Ok! Were the doctors of the first hospital you attended able to tell you what kind of sickness he was infected with?

R: I immediately took him to the hospital when he started feeling ill. Over there I was given a medicine for the treatment. The nurse then told me that this kind of medicine is usually given to those with convulsion for treatment but she doesn't understand why my son's situation is still like this. So she said they would have to do an operation on him. And it was all about money.

M: Did she do it?

R: They did it for him!

M: you said he got ill when he was three months…?

R: Yes! He was attacked by the sickness when he was three months.

M: Ok! So he was three months?

R: Yes!

M: So with your first visit to the hospital what was done for him?

R: There is this medicine that was given to him but he was still the same.

M: Did the doctors tell what sickness it was?

R: The doctor asked me whether I was sick when delivering and I said no. And he said this kind of sicknesses attacks the child brain. So he gave me a medicine to cure it. So it is prayers and the medicines we buy from Ankaful is what we give to him so far.

M: So that's what the doctor told you it was?

M: He said it was like convulsion.

M: So it started like convulsion?

R: Yes!

M: Ok! Right now, what things do you see about him now that you see that he is getting better?

R: I was the one that makes him sit but one day, after bathing him, I went back see that he has been able to sit.

M: How old was he?

R: He was around seven years.

M: What other things again?

R: At first, he wouldn't respond to anything but now you can play with him. And he can see what happens around him.

M: So can he do things for himself?

R: No! He cannot even hold things properly. So I do everything or him.

M: So what if he wants go visit the toilet?

R: He has been wearing pampers and I change it for him whenever time is due.

M: Ok! I want to know what things that he should be doing that he is not doing for himself at this age.

R: Because of what the sickness has made him, I really wish he would be able to talk or even fetch water for himself when he is thirsty. He hasn't even started walking.

M: So talking and walking has delayed?

R: Yes!

M: Ok! I want to know the relationship between Albert and the other children in this house?

R: They play together though but whenever he can't do certain things, they help him with it. He doesn't even talk when they are playing together. Sometimes too his father will be playing with him in the compound here.

M: But is he able to talk with them, do they play with him?

R: They play with him but it is not often.

M: Does he play with his sibling?

R: Yes! When she comes back from school, she will come to him for a while.

M: Ok! So who has been taking care of him most of the time?

R: His father. Sometimes when his father takes care of him then I will go to work. Even this morning he was the one who took care of him.

M: Why do you always have to stay close to him?

R: Sometimes his sickness becomes very terrible that's why someone has to be close to him all the time. The medicine calms it down but it's serious when we don't give him the medicine.

M: So how does he behave when the sickness becomes severe? Does he shiver?

R: Yes!

M: What do you do when he shivers?

R: He will sometimes shiver or he sometimes becomes stiff for some few hours and then he comes back to normal.

M: Is that the reason why someone must be close to him?

R: Yes!

M: does he have anything that he plays with or learn with?

R: He doesn't really have but his father bought one small musical keyboard for him. Even that one unless someone helps him to play.

M: Has he been playing with the music keyboard for a long time?

R: Yes!

M: So does he play often?

R: He cannot press on it himself. His sister or father has been playing for him. And he really loves the melody.

M: And does he understand what the melody is?

R: Yes, he does!

M: And does he see when someone is beside him?

R: Yes please!

M: So all what he cannot do is that…

R: He cannot talk, he cannot stand, and he cannot hold anything but he sucks his thumb.

M: Why is it so?

R: That's what the sickness has done to him so he behaves like a child.

M: So if he want to go out and play what does he do?

R: He cannot walk how can he go and play?

M: Maybe that day he wants to play or something?

R: He does it alone

M: So you haven't allowed him to go out and play?

R: No! He is always with me when we go out.

M: So haven't you taken him to town before?

R: I go with him to the hospital. Even yesterday I went with him.

M: So apart from the hospital, do you take him out?

R: Not really! But when his father is not home, I take him to wherever I go!

M: Where do you seek advice concerning his health and upbringing?

R: I don't have any special place I go. When we go to church, the pastor always console and encourage me and say that God is in control. Some people also do same especially those who care.

M: So concerning his health, where do you take your advice from to take very good care of him?

R: I don't go to anyone!

M: So don't you take any advice when you visit the hospital?

R: They just tell me that this kind of children that's how they are so I should take very good care of him.

M: so sometimes when something is bordering you who do you go?

R: His father sometimes call someone for advice. Sometimes he calls his friend or relative.

M: I want to also know what has been going on for children like Albert or what is been done for them?

R: Government sometimes brings money for them. So when we go, they ask of their wellbeing just like how our church do.

M: so apart from his father calling to friends and relatives for assistance, does he consult any specialist who knows much about Albert's sickness?

R: We have gone to so many places; Pastors and fetish Priests. But his father doesn't like traditional way of life. So we always went to places that people directed us to. And we paid money to them for attempting to cure the sickness.

M: so you mean there is nothing done for children with this kind of sickness in this vicinity?

R: No!

M: so what is society's reaction to Albert?

R: The sickness seems strange to some people so when they come around, they just stare at him and leave.

M: I want to know whether they accept his deformity and live with him as one of them.

R: Yes! They embrace him as one of them but I'm the one who don't usually give him out to them.

M: is there anyone in your family or your husband's family with this kind of sickness?

R: They told me that when my brother and I were young, we had convulsion but we were healed immediately we got to the hospital.

M: can you tell us what you have been doing for Albert that is good?

R: The exercise we have been doing.

M: You do exercise?

R: Yes! Wednesdays

M: where do you do it?

R: Ahotor.

M: Apart from the exercise what do you think you do for Albert that is helping him?

R: His father has been helping him to hold thing but he still cannot hold them properly. And his father says we should be conversing with him. So his father converse with him a lot. Even though he doesn't speak, his father ask him lots of questions.

M: what are the kind of things that you know you should do but you cannot do for your son? Things that you know could help him but you can't do for him?

R: I have been told to serve him with 'Kontomire' to eat but he doesn't like it. And I was told to give him 'Impua' and milk, he doesn't like it too.

M: so apart from this what else do you know that you have do but you can't do for him or very difficult for you to do for him?

R: That's all!

M: so concerning his diet, why don't he like certain foods that has been prescribed for him?

R: He likes eating one kind of food ones. He will never eat it for the second time. So it makes it difficult to feed him.

M: can Albert chew things?

R: Not really. When he puts food in his mouth, he doesn't chew it very well but he will swallow it. And when you put your finger in his mouth, he can't even bite you hard.

M: how difficult is it for you in taking care of Albert?

R: I don't really have money and also I can't leave to go to where I want to go.

M: so apart from this, what else makes caring for Albert difficult for you to do?

R: It is very stressful and also if we don't have money, we can't get the necessary things to keep him alive. So at least, there should be one person to take care of him.

M: What do you do for Albert that is different from what you do for Florence? For instance, what were you told to do for Albert.

R: That's his food and the kind of medicines that was prescribed for him. We should see to it that he takes it every day and make sure when it get finished, we buy some for him.

M: Who taught you about his diet?

R: I was taught in the hospital.

M: so what have you been able to do about this?

R: They taught me some skills that I can do to handle Albert. I even knew what they taught me already

M: so when Albert plays with the children here, do you see them teasing him or doing anything bad to him.

R: They don't tease him but what I have observed is that people stare at him a lot.

M: with that how do you feel?

R: Nothing.

M: what measures have you put in place to make Albert fit in society when he grows?

R: Nothing!

M: even though Albert is still young, he might go do something wrong. How do you correct him?

R: Whenever he is refusing to eat, I will just hit him slightly on his hands.

M: so apart from his hands where do you hit again?

R: his back

M: has any agency come to this area or to this house to teach you on how to care for Albert?

R: No one or agency has come to this house.

M: Is there a place you can go to in this area where they help you in caring for Albert?

R: That is the Ahotor School he attends.

M: where can Ahotor School be located?

R: It is behind Ankaful

M: so you take him to school every day?

R: Yes! A car picks them up for school. So on Wednesday, I will take him there for exercise.

M: So when did you start taking him to school?

R: It's quite a long time.

M: did you see any difference when he was schooling?

R: He was taught to sit and turn around by himself. I did it for him at first but he started doing it on his own.

M: Why did you stop sending him to school if the school was a good one so that he would have become what you want him to be in future?

R: It's all financial issues. And sometimes the sickness becomes very serious when he is in school. So I have made stay home to treat his sickness for a while.

M: Would you prefer that there would be an institution to give counsel to parents with children like Albert?

R: It would be good!

M: In which way will it help you?

R: As Albert can't walk, it would be a good thing for that particular institution to help him walk.

M: This institution I'm talking about will only teach these kind of children. So will it help you or not?

R: It will help me.

M: So assuming Albert was a female, would there be any difference?

R: It would have been the same but the only problem would have been at the time she will be menstruating.

M: Why are you saying it would have been a problem?

R: She might menstruating in my absence and that would have been the problem.

M: Were you educated on the kind of sickness with Albert when you visited the hospitals?

R: They said they would be giving him medicine for a long time to cure his sickness.

M: Who said that?

R: The Doctor. He said I should be patient because it will take time to treat this kind of sickness.

M: Did the doctor also educate you on how you can handle the pressure from your son's sickness?

R: Yes! They gave me some encouraging words.

M: What kind of help do you need from government, the hospital, schools, social welfare?

R: The government promised to help us buy the medicine for Albert and when it doesn't come I buy it myself.

M: Apart from the help from the district office, what help do you need again from the hospital to care for your son?

R: It's still about money. When you see the doctor, whatever medicine he prescribes for you will be bought with money.

M: So what should the hospital do to help you?

R: They should help me with the medicine.

M: Let me get it clear, what should they do?

R: From the hospital, they only write the medicine for us to buy.

M: I still want to know the kind of help you need from the hospital? For example, someone prefer that the hospital will give him the medicine for free. So what do you also want the hospital to do for you?

R: Yes, I would want them to give me the medicine for free.

M: Ok! What kind of do you need from school also?

R: That's what the Ahotor school is doing but they take fees. And the fees they take is used to pay the workers over there.

M: But why haven't you taken him to any of the schools in the area?

R: With his situation, he cannot go to any of those schools.

M: So if there is any arrangement to allow children like Albert to go to any of the schools in the area, would you send him there?

R: I would take him there but I think he wouldn't be happy because he cannot speak.

M: You know, Albert would surely grow one day and have you considered how you are going to move with him? As at now you can carry him so there is no problem. When he is old you cannot carry as you are doing now.

R: We have made his special chair for him but I still carry him to wherever I go. I asked the carpenter and he said he makes such chairs for grownups. It is Ghc400, very expensive for me.

M: Do you have any question for us?

R: I want to know why you are here asking me all of these questions.

M: We were sent by the government. What we are actually doing is to understand these kind of situation for the government to make policies that would help you. And when it happens that way, you will be added to the system so that hospitals will treat you people special.

Government does not know the number children with these kind of sickness.

R: They don't know the number of children though but they gave us Ghc500 to take care of our expenses.

M: The reason why it has not been coming regularly is that, the number is not stable. So what we are doing will make the government know the number of children with this kind sicknesses.

<u>IDI INTERVIEW WITH A MOTHER WITH A SPECIAL CHILD 1 YEARS 3 MONTHS OLD BABY GIRL</u>

R: I have 6 children and I gave birth to 4 children with his dad and we buried 3 and now left with 2. But for me I have 5 children.

M: Her dad is different. I want to know for now including her and the husband how many children are they living here?

R: We are 8 in the room.

M: How many children?

R: The children are 5.

M: then 3 adults.

R: Yes.

M: Thank you, eerr, your kid, I don't want to mention the child's name in the conversation, do you get me? So don't mention the name here. What can you say about your child?

R: Please my child, she is 1 year 3 months when I have birth to her, we couldn't eat, we couldn't sit down, we are always crying. I can't sleep when she is not asleep, that's what I will say.

M: OK, please, she means, when the child was born, she don't sit down.

R: She can't eat, she only cries.

M: She can't eat.

R: So I want you to do something about it for me.

M: She can't eat, she can't sleep.

R: Yes.

M: OK, thank you, and aside she can't eat, she has been with her for 1 year 3 months so what can she say about the child? What can you say, what type of child is she?

R: I took her to Ussher clinic and they gave her some foods to eat but they didn't help her so they referred me to Children's hospital but I have not taken her.

M: OK, thank you, when she looks at the child now, what and what does the child of 1 year 3 months do that the child hasn't done that has delayed a lot?

R: I want you to take care of her so she can walk so that I can also be a little free.

M: I understand but I want to know...I understand you

R: Ooh!

M: Sorry, this isn't your first born, you have other children already. When a child reaches a certain month there is something the child has to do but I want to know a child that is 1 year 3 months should be able to do this and that but you haven't seen anything like that with the child. That's what I want to know.

R: All my children walk early after birth but this child that I gave birth she is doing me like that. But we want to a certain place to do something but they said we have to summon her spirit before. I came to tell her father but the father hasn't paid attention to me still.

M: OK, if I'm right then she meant the child can't sit, she can't walk and she can't eat well. Have you taken her to the doctor to tell you what's exactly wrong with the child?

R: I took her to Ussher clinic and they gave her Kwashiokor foods to eat but they didn't help her so they referred me to Children's hospital but I have not taken her.

M: I want to you to tell me something small about your pregnancy? How was the pregnancy like when she was pregnant with the child?

R: When I was pregnant, I was in pain so I didn't deliver at Korlebu but I delivered at home.

M: Why the pains, why were you sick?

R: I was very sick throughout.

M: Did you go to Korle Bu when you were pregnant? Did you go and take a card?

R: I went to Ussher clinic. I even have the hospital card with me.

M: What did they tell you?

R: They told me to come later but I didn't go and came to live at the seaside so when the place was destroyed came to live here.

M: She gave birth at home. When she gave birth at home did the baby cry?

R: When I gave birth to her, she didn't cry but, some water was in her...and we called and they came to remove it but it was later we took her to our place.

M: Didn't you take her to Korle bu?

R: It was later when it became serious we took her to Ussher clinic. She wasn't able to eat.

M: OK, thank you, I want know something, eerr, is the child familiar with others? At her age does she play with others? Does she play with her siblings?

R: No, it's only me. She is always by my side. She can't sit, she cannot do anything so she is always at my back.

M: Please I want to know something, you made it known to me that, she is always with her mother.

R: Yes.

M: Please can I know something small? At her age, has she started to eat a little? Aside the breast milk, determined does she drink porridge or any other food?

R: As for the porridge, when I buy I force her to eat but she doesn't eat rice and banku.

M: What type of porridge? Is it the corn porridge or the millet?

R: The white one.

M: OK and do you add anything else to it for her?

R: Only that.

M: Only the porridge. Please I want to know something, she is very young but by this time she should be playing by herself.

R: Yes.

M: Has the mother bought anything that she use to play it the mother...

R: She doesn't play. She hasn't do such things.

M: Have you bought like toys or dolls for her to play with?

R: No, I haven't bought some for her.

M: And does she play?

R: She doesn't play.

M: Is she always sleeping just like that?

R: Yes.

M: Please I want to know aside Usher fort that..

R: Usher clinic.

M: Usher clinic, when she reported to them when she saw that the child the child wasn't well. Who else will she take an advice concerning the health of the child.

R: Apart from Usher clinic?

M: This is about your view on this. I want to know, she went to the hospital and she didn't go when she was referred. Didn't she go to any elder? I want to know if you took an advice from an elder concerning the health of the child.

R: I didn't talk to anyone.

M: Please why didn't you?

R: My mind wasn't there.

M: OK, thank you, I want to know if you have a child in Jamestown here and you have a child and she can't walk, she isn't growing well and other stuffs, how do people see the child?

In: This woman, she is not worried about the child. If you talk about it then the husband is angry. I am worried and a certain lady and a carpenter her too is worried about the issue.

M: So I want to know that for this place, do everybody wants to help?

In: Yes but we don't give that chance.

M: Everybody wants to help.

In: Yes but we don't give that chance.

M: OK, I want to know if they have some cultures at this place that help children who is like this?

In: She said she went to summon her child and she is a queen.

M: Please ask her, its as if you are answering the questions.

R: We didn't summon her spirit yet but her mother's sister said she should bring 50 cedis so she summons the soul of the child and find out what is wrong with the child. But when she told the husband he didn't care about it.

M: OK, so the people here how do they feel when they see children like this? Do they ask her where are these children from?

R: They haven't asked me about something like.

M: OK, not that someone has asked you but, please have you heard that, I didn't say what they...for her, they haven't told her anything but let's say if you are walking and you see such children, what do these people see them or what do their traditions tell them about who those children are?

R: They see that the child even cries at dawn, she doesn't sleep and I carry her at my back at dawn and walk about:. But some people said the way the child I should take her to Korle Bu but still if I tell her father, he himself sees what the child does and hears what people see and say about it but he doesn't care.

M: OK, thank you, please right now so you know what's wrong with the child?

R: I don't know.

M: So what she knows is that, the child isn't growing, she doesn't sit, she doesn't eat.

R: Yes, she doesn't do anything, she doesn't sit. When I'm doing to Bath, I go with her, when I'm going to the toilet I go with her, when I'm going to the sell I go with her, when I'm doing...helping others she will still be at my back. She doesn't do anything, when you make her sit, she will cry.

M: That's the only thing she does. But do you feel that, what's happening to her is from birth but not when she started growing that the thing came.

R: As for me I don't know.

M: OK, she don't know. I want to know that if there is someone in her family that whose child is like this?

R: No, I'm the only whose child is like that.

M: She has made me see that she is very tired, she is very tire. She is always at her back, when she is going to sell, she goes with the child. She does everything with the child. I want to know what is she doing to be able to take care of the child? What is she doing as a parent. By this time a child like this, you can make and sit down and do other things but...I want what is she doing about the up bringing of the child. What is she doing well about the child?

R: I am doing everything thing but still, that's how she is. I don't know what to do again.

M: OK, I want to know something, if the child is...the child is a special child. She is different from other children. What is she doing as a mother, not in money aspect but what is she doing about her up bringing that she can use to help the child?

R: So I want to sit at home and look after her and her father too hasn't been giving us money. I am the only one who is trying...and I have other 2 children too.

M: I want to what businesses is she doing?

R: I sell fish, I roast the fish and then sell them.

M: OK, so is the child at her back when she is roasting the fish?

R: She is at my back, I don't put her down and I alone do everything.

M: So is she the only person looking after the child? Is she with her when she is working? Is the child with her when she is going to sell?

R: Yes!

M: Thank you, I want to know something, you see this child is a special child and her up bringing needs special skills, as she said, she takes her even when she is going to take her bath because you can't put her down so you have to go with her. I want to know what makes it difficult for you in looking after this child?

R: Anything encourages me to look after her because of how she what she does. What I'm still doing is not working so I have to go everywhere but still that's how she is.

M: Thank you, I want to know, eerr, she should explain to me on...I want to bring her mind on something. She even said we should help her because she is suffering because the child is always at her back. So this makes her very tired, she is at her back even when you are

working. So I want to know as the tiredness, is there anything that is very difficult for her to bring up such a child? Is it difficult to bring up such a child? I want to know if because of the child she has learnt anything special that helps her to look after the child.

R: I haven't learned anything. But I can look after her very well. Today I didn't go to the market so I will look after her.

M: I want to know something, I want to know if her siblings or someone teases her? Do someone or her siblings teases her or bullies her?

R: No.

M: I want to know something, how is her nature's call?

R: When she wants to go to toilet, it comes very small because she hasn't been able to eat well.

M: OK, tell her that we are done with the conversation, have you seen? We are done, left with...she is very young but I don't know if she can do something things that are painful or something that she needs to be corrected or if she wants her to know she, in what ways does she make her see that...

R: I don't do anything.

M: What tone do you use.?

R: When she is crying, I shout at her to stop crying and I tell her does she want me to worry? Why, she doesn't want me to sleep at dawn but she is not the only child I gave birth to but the children I gave birth to don't do behave like this but she is the only one I gave birth to that is doing this.

M: I want to know when she went to Usher hospital was she given some teachings about how to take care of the child?

R: I wasn't taught but when we went to there, they gave us food to give her but even the food I force her to eat it.

M: What type of food was she given?

R: Groundnut, groundnut in a small container, everyday when I go they give me 6, It gets finish yon Fridays then I go back to buy again and bring it.

M: OK, I want to know if in this area there is an organisation for those having a child like this that they can help take care of her.

R: As for me I don't really know.

M: She doesn't know about something like that. OK, thank you for the conversation about your child because I was asking if you have heard about it, what did they tell you about your child but you said you haven't heard anything. When you went to Usher too, they didn't tell you anything about it. They only thing they did was to give you food for her. Didn't they teach you how to take care of her? Or they said it?

R: They didn't say anything, the only thing they gave me was the groundnut I give her at home.

M: Ok

In: They think the child is Kwashiokor, so

R: That's what they tell me.

In: So they always give the child food for Kwashiokor.

M: OK, thank you.

Good afternoon and how are you doing?

R: Am doing well.

M: Thank you for accepting to have this interview with us.

R: You are welcome.

M: The first face of the interview involves home setting and general knowledge. Can you please tell me a little bit about you home?

R: We relate well in the house, they do not liked me when I gave birth but they later realized we have children that are born like mine, so we are ok now.

M: How many are you in the family?

R: We are 5, I have 4 children and myself included.

M: Is your husband not part?

R: He do not live with us.

M: **Are all your children male?**

R: Yes, I have 2 boys and 2 girls.

M: **What can you tell us about your child?**

R: She did not cry when I gave birth to her. She could not do anything. My child could not sit, the nurse at the hospital gave him drip but to no avail. People later told me that my child was not a human but she is from the marines.

M: **Was that what you were told?**

R: Yes. I have to stop work because of my child.

M: **What happened at the hospital before your child was given a drip?**

R: She was not crying as a baby and later we realized she could not breath proper so they have to go her oxygen.

M: **Are you the one who gave birth to her?**

R: Yes am the one, they wanted to perform a surgery on me but I later gave birth by myself.

M: **How long did you spent at the hospital after given birth?**

R: We spent 3 months at the hospital before we were release. We were release when she started crying.

M: **Where you kept in the hospital because she could not cry.**

R: Yes, moreover, she could not suck breast for breast milk, the breast milk was going through a drip to her belly. Later I started feeding her and she started sucking breast a little that was when we were release.

M: **What can you telling me about your child growing up?**

R: I felt sad.

M: **Why?**

R: Because when I meet my friends and see their children, my child do not look the same.

M: **How was your child like after 3 month?**

R: My child was just laying down. She does nothing. She was looking weak. The neck was not firm. It was same after 6 months that was when I realized by child was not normal.

M: **Did she crawled?**

R: No, she rather crawled on her belly.

M: **Does she speak?**

R: No, she cannot speak with her mouth. She speaks through signs.

M: **How does she speaks through signs**

R: Assuming she wants to call me, she will make a signal. When she wants water to drink, she does signal me, same with when she wants to urinate but she do not signal when she wants to defecate..

M: **How old is she now?**

R: She is 8 ages old now, and she still wear diapers.

M: **Has she grown teeth?**

R: Yes, all her teeth are grown but she can use them to chew food however, she can to chew bottle.

M: **What kind of food does she eat?**

R: She eat all kind of foods except okro.

M: **Did she says she does not like okro?**

R: When she eat okro she gets ''drop attack'' seizures.

M: **Does it means that, children with those seizures do not take in okro?**

R: Yes, the elders said it.

M: **You said, she can say 'ma' did it took her a long time to be able to say that?**

R: Yes, it took him very long time to say that.

M: **How long did it took him to say ''ma''?**

R: It took him 7 years. She start calling ''ma'' when she started school and because she hears her sisters saying it so I think she pick it from them.

M: **Is she schooling?**

R: Yes, she schools at New Rising in Cantonment.

M: **Does she stays in the school. On the other hand, she goes to school daily from the house.**

R: She attends the school daily.

M: **What did the doctors said was wrong with your child?**

R: The doctor said, my child is suffering from autism.

M: **How can you describe your child development as a child, did her development delayed?**

R: Hmm! A normal child should start crawling at least after 7 months but I did not see any sign of that with my child. I planned killing her but her grandmother ask me not to kill her because it was for a reason God gave him to me. His grandmother encourage me for that thought to fade away.

M: **Why did your decided on killing your child?**

R: When I see my friends children going to school and mine is not, she could not talk nor do anything, she could not even walk. She started walking at the age of 7 years.,(M cuts in)

M: **So she has started walking.**

R: Yes, but not firmly. She walking side and side.

M: **Can she eat by himself?**

R: Not really, she will soil himself when she try to eat by himself. I always wants to feed her.

M: **How does your child relate with other children in the community?**

R: She relate well with but they do not play much with her as they do among themselves. She mostly plays by herself. She knocks her head against the ground.

M: **Does she plays with her siblings?**

R: They play in the room but not outside.

M: **Is she always indoors?**

R: She is mostly in the room but comes out when the light goes off.

M: **So she stays indoor when the lights are on?**

R: Yes but she destroys things in the room.

M: **Things like what does she destroys.**

R: Things such as television or picking someone mobile phone and put it her mouth. She could chew phone chargers and earpiece too, that is why she is always indoors.

M: **Whom does she spend most of his time with?**

R: Me.

M: Why you and not any other person?

R: Sometimes, I leave her with my mother or her father when he comes around.

M: Why do you want her to spend more time with you?

R: Because I know how she is.

M: How is she?

R: When she needs something, she makes a sign that I understand, another person might not understand that sign. When she is hungry, I know, and when something is wrong with him, I know.

M: Does your child has items she plays with?

R; Yes, she plays with those things and end up destroying them. She chews them.

M: Can you mentioned what she had play with and destroyed?

R: She used to have lego but she chewed it. I do not give such things to her again because I know she younger ones also need to play with such things.

M: Does she has a space or place she plays?

R: She plays in the room.

M: Whom would you see advice from on your child's health, or how to nurture your child?

R; Hmm! I do not no. We go for checkup.

M: When do you go for the checkup?

R: Every one-month, at the police hospital. Therefore, in case of any advice, I will go to the doctor.

M: **That means you will seek advice from the doctor concerning your child's health.**

R: Yes.

M: **Whom would you seek advice from on how to handle your child?**

R: Nobody.

M: **Why do you visit the doctor on his health and not any other person?**

R: because that is the doctor's job and the doctor knows the medicine to prescribe.

M: **Are there culture practice regarding people like your child in this community?**

R: None that I know of.

M: **How do the people in the community sees you, with a child with autism?**

R: I shear children like her I send off, but no one has told me that in this area.

M: **How do you send the child off?**

R: The child would be shot so she could go back to where she came from.

M: **Shot, with a gun!**

R: Yes, you will send the child to the fetish priest, then the priest will send the child off. However, no one has said or told me that in this community.

M: **How are child as yours called or refer to in this community?**

R: They say children such as my child are from the marines.

M: **How do the people in the community react or relate to your child when they see her?**

R: They just look at her but do not say anything.

M: **What about people you live with in the house?**

R: They have not said anything to me. They do not come closer to me preciously but I think an education of such children was show on the television, I could say that they stated getting closer after they watch the program on television.

M: **Was it because of your child, they weren't coming closer?**

R: Yes, but they have come close to him now.

M: **At what age did, you realized your child was an autistic child?**

R: We went for a checkup when she was 3 years old and the doctor confirmed she has autism. I was not informed, it was my husband they told, I got to know about it later.

M: **Did the doctor taught you how to handle such a child?**

R: No, I was not inform, the doctor did not say anything to me, the doctor rather rather told husband. It was later my husband informed me. The doctor thought am a woman so I might not be able to handle the news.

M: **So your child had the illness right from birth.**

R: Yes

M: **Are there traces of such illness in your family or that of your husband's family?**

R: No, there is nothing like that in any of the families. It seems am the first person to bring it to the family.

M: **No one has such illness in either of the families.**

R: Yes, my husband nearly end the marriage because of that. Because he was thinking, there is no such thing in his family. Therefore, his family had to meet with my family and they advised him that, there is no trace of such illness in either families. If he should

leave the marriage, who is he expecting to take care of such a child for him? That is why my husband stayed in the marriage.

M: Was you husband wanted to leave the marriage as soon as your gave birth or after the child had grown?

R: After the child had grown.

M: Has your husband have an understanding of such illness now?

R: Yes, that is why we did not divorced. In addition, we have 3 children afterwards.

M: How was it when you were pregnant?

R: I suffered during pregnancy. I once vomited blood.

M: Which month of the pregnancy did you started vomiting?

R: I realized I was pregnant after 3 months, that is when the vomiting started and I was rush to the hospital.

M: Did you became well afterwards?

R: Yes.

M Therefore, nothing happened to you afterward.

R: Yes.

M: What can you say you are doing well regarding catering for your child?

R: Is the father that does much, the father is the one working now. He buys diapers for the child. In addition, my siblings supports me because am not working now.

M: Do you know your child is a special child?

R: Yes.

M: What you think you are doing well in nurturing your child?

R: Feeding her and cleaning her when she defecate on herself. Sometimes she does things that get me angry but I regret after beating her then I apologize to her.

M: What are some of things your child does that angers you?

R: Sometimes she goes to destroy people properties and I will end up been insulted. Then out of anger, I will also beat her. My husband warn me that I should let the child always be in the room, so the child could only destroy things in the room. My husband said he is okay when his child destroys his things than destroying other people's property.

M: Are there things or help you would have wish to give your child but you are not able to do?

R: It is her father I would wish to support to take care of the children, if am working.

M: What support could you have given if you are employ?

R: I would have like to work but there is no one that can take care of her especial when she is on vacation. That is why I can work too.

M: Aside you not working, are there other things you would have wish to do for your child?

R: I just wish I could support my husband. He is the only one paying all the fees. However, if I should start work, there is no one to take care of the child.

M: Can you describe your difficulty in parenting a child with disabilities?

R: It is difficult because you have to feed, bath and do almost everything for her. She cannot do anything for himself, she is 8 age years now, a normal child her age your be able to at least sweep but she cannot do anything. She still wears diapers at her age.

M: Do you need a special skill to parent an autistic child?

R: Most of my friend have left me because of her. In addition, I cannot leave her behind and go out for any social gathering. Those situations has thought me a lesson in life and made me trust in God. Because I did not know, my child could walk one day.

M: Aside the signal your child gives you when she needs something, are there other means she communicates to you?

R: No.

M: What is she taught in school?

R: When they hold her hand then you will see her shaking. She can recite the alphabet and you will see her acting 'my head, knees and shoulder' when it is sand.

M: Oh! I see.

R: She was gradually picking up with how to speak before she had a stroke.

M: Does she have a stroke too?

R: Yes, half of his body is not working now. She became okay that we even wanted to send him to a normal school before she had the stroke.

M: How were you able to detect that she had a stroke?

R: She became weak.

M: Did she fall sick?

R: Yes, she fell ill and all of a sudden, we realized she had stroke. She can do anything now, she cannot even open his palm.

M: She did stayed long at the hospital?

R: Yes, for about a month.

M: Is she tease by the children around?

R: Oh no, they just look at her in a way but do not say anything to her.

M: Has your child ever reported been tease by someone?

R: Oh no, none that I know of. In addition, she cannot even speak, so she may not be able to say it.

M: Have you prepared her on how to handle hherself when been tease by the children around?

R: Oh no, have not started just.

M: Why you not started?

R: Because I do not think, I have it I have it in mind that someone will tease her. She plays by himself.

M: Does it mean she do not play with anybody?

R: Yes, she plays alone. You will see her sitting and later walk to the door.

M: Can she sit by himself now?

R: Yes. However, she cannot sit at a place for long. She plays around.

M: Does your child have any responsibility in the house or does some house chores?

R: She does nothing. I do everything for her. I feed and bath her.

M: Can she take care of her other siblings?

R: Oh no, he is a male so I cannot let her to take of her brother.

M: Why can't she take of her brother, is it because he is a male?

R: Oh no,

M: **But you claim you can see her improving, don't you trust her to take of her brother?**

R: Haha! I just cannot leave my son for his sister.

M: **How do you correct you child when he does wrong?**

R: I whip her with cane.

M: **Does she understand why you caned her.**

R: Yes, she does understand, she do not repeat the same thing again.

M: **Oh okay.**

R: When she is beating for the first and second time, she does not attempt to do the wrong thing for the third time.

M: **Besides caning her, are there other means you could correct her?**

R: I beat her with my hands sometime.

M: **Are there the only means you use?**

R: Yes.

M: **Do you talk or advise her?**

R: Yes, I do talk to her but she cannot speak back.

M: **Can you give an example of something she did wrong and you disciple her?**

R: She can give signal when she wants to urinate but do not give signal when she wants to defecate. Have been advising her that, she is grown so at least she should let me know when she wants to defecate, she will agree but still defecate on herself.

M: **Now that she is in the room the room, how would you know if she wants to urinate?**

R: I have worn her diapers.

M: Have you had some sensitization about to live with your child?

R: Yes, at Share Care.

M: Where or what is Share Care?

R: It is a center in the Osu Township, they train people on how to handle such children. I had a physio session with them.

M: How is the attendance of Share Care like?

R: We attend 3 times in a week.

M: Oh, okay.

R: Yes, is been a while I went to the meeting because same is been then for her at school.

M: Therefore, Share Care taught you how to live with him.

R: Yes, I was taught the kind of excise my child has to be doing.

M: Were you educated on the kind of meal or food you should give your child?

R: No.

M: When did she start schooling at New Rising School?

R: 2016. About 3 years ago.

M: Did Share Care encouraged you to send your child to New Rising School?

R: No, but rather the boss of by husband that made us send our child to New Rising.

M: Has your child attending New Rising helped?

R: Yes, it has helped because I did not know she could learn but when he started schooling, I realized she could learn.

M: Have you been teaching her?

R: Yes.

M: What changes have you seeing with your child since she started school?

R: Yes, she used to sing gospel song but when she started schooling, she began singing songs from school before she had the stroke.

M: So she was able to sing before?

R: Yes, when we visit the church, everybody goes like, the pastor is in then everyone laugh. All that went away when she had the stroke.

M: Does the Share Care only takes care of autism patients?

R: They take care of people like my child.

M: Is Share Care an NGO or a government organization?

R: It belongs to an individual; it has really helped because now people like me has the boldness to take our children outside without shame.

M: Do you know the owner of Share Care?

R: A woman called auntie Becky is the one in charged but it seems, it belongs to some 2 individuals who joined their effort together.

M: Are they Ghanaians?

R: Yes there are.

M: What materials or items are shared among the people at Share Care?

R: They do gives us rice sometimes, diapers and whips.

M: What can you tell us about Share Care?

R: They organizes parties for us during Xmas and shares items such as bag rice, bags and some amount of money among us but it seems, they are tight financial now.

M: Which year did you joined the group?

R: I should be between 2013 and 2014.

M: Can you say for a fact that Share Care has been of helped to you.

R: Yes, they have really helped me.

M: In what ways have, they helped you.

R: I request for money sometimes from them when I do not have.

M: Aside Share Care, are there other organization that helps children like yours in this community?

R: I do not know of any such group or organization in this community. I only know of Share Care.

M: Does the police hospital have similar program?

R: No.

M: Is there something you will like to tell us regarding nurturing someone like your child that we have not touched on?

R: It about finance because sometime what to eat is very difficult. It is only my husband that work, I would not know what would have happened if I were not staying with my mother. I sometime give my children food and go to bed on hungry bell sometimes.

M: Thank You.

MV: Thank you for agreeing to have this conversation. For my first question, I want you to tell me about your family in this house.

R: We are two people

MV: Ok, so you the mother, and who else?

R: And my husband.

MV: Who else?

R: That is all.

MV: What about the child?

R: And the child.

MV: So making three?

R: Yes please.

MV: I want you to talk about whatever I ask you. Very soon I will be asking you about how you discovered something was wrong with regards to the health of your child. So please, I want you to tell me something about the child.

R: When he was two years, we realized he couldn't speak. At the age of three years, we took him to the hospital and we were told there was nothing wrong. From three and a half years up till four years he still couldn't speak and his teachers were complaining at school. I was the only one who could hear him speak but other people complained about it. So we went to the hospital and the doctor referred us to Tamale. We were told that there was nothing in there so they washed his ears but he still has a problem in speaking; some of his words are not clear.

MV: But is he able to hear clearly?

R: Yes he is able to hear clearly but he cannot speak out well.

MV: Ok, I've heard you. You mentioned that people complain when he speaks. How do they do that?

R: That they don't hear his words. Even when he's playing with her friends or talks to anybody, they complain that they can't hear the words well.

MV: Please what does your child do best?

R: He is able to do what he is thought in school but for him to read out clearly, it is difficult for him. He can act and sing but there are mistakes in his speech while he does that.

MV: What was his condition at the crawling stages of your child?

R: He fell sick often. He crawled for a long time. He was about two years before he got up to walk.

MV: Did you suspect any ill-health?

R: Yes we did so we took him to the hospital and we were told there was nothing wrong with him. So he got up to walk after some time, and then we also discovered there was something wrong with his speech.

MV: Children are usually trained to sit on a chamber pot, how was it with him?

R: He was taught to do all of that and he did.

MV: He didn't encounter any problems with that?

R: No he didn't.

MV: What was the condition when he started talking?

R: That was a problem. It took him a long time to talk just like it took him to crawl. When he started talking, there were mistakes.

M: Which of the things did he slag in doing at the various growth stages?

R: He is not as active as she should be. According to the teachers he stays quiet throughout classes unlike other kids who play around.

M: So why do you think he doesn't do that?

R: I think it is because her speech isn't clear and he fears he will be teased in school that is why he isolates herself from his peers.

M: How does your child relate with her friends?

R: At home, he plays with his friends even though he is sometimes scared and has to run home when another child bites him. He doesn't retaliate, he just runs home crying.

M: What kind of play do they engage in?

R: Sometimes he joins them to football but he doesn't play in school. When he goes out too, he wouldn't play.

M: Does he have younger or older siblings?

R: No please he is the only one.

M: Does he have any relationships with some cousins?

R: No, they don't live close by. He goes to one lady who sells close by to play with the children there. Apart from those children, he doesn't have any friends anywhere.

M: Who does he spend most of his time with?

R: Mostly he likes his father but then he runs to me whenever he needs something because I understand his words better. He tells me to interpret whatever he is saying to his father because he doesn't understand his words so I do that for him.

M: Why does he like his dad?

R: Right from childhood he's been handling him well a lot of times so he's used to him and sometimes it's very difficult for him when his dad travels.

M: How was his dad handling him from childhood?

R: He goes with him on strolls or buys him kalyppo and other sweets if only he has money.

M: Who amongst his friends does he spend most of his time with? Does he have friends?

R: As for friends I can't pinpoint any, he doesn't really have a friend apart from children in this house. Moreover, he closes from school at 3 after which they meet. But then, to pinpoint a particular friend he goes to would be difficult.

M: Who amongst the children in this house is he really close to?

R: There's one girl here she is very close to.

M: Why do you think she is very close to that girl?

R: I think it is because of how she relates to him. Whether she hears his words or not, she still moves with him and is quite older than my child.

M: Does your child have playing toys?

R: Yes he has cartoons and books that he traces and colors with the crayons by himself.

M: How helpful has that been for him?

R: It has helped him in his writing very well. But for him to read after clearly is a problem. Some of his words are not clear.

M: So whenever he wants to play at home, he plays within the confines of the compound but stays in school quietly without playing with his mates for fear of being teased, right?

R: Yes please.

M: Where else does he go to play?

R: He doesn't go far. If he isn't at home, he is at a park nearby playing with the kids or with his father playing with the kids at his shop.

M: What kind of play do they engage in on the park take?

R: They're either playing football on the park or running.

M: From whom would you seek advice with regards to the health, school and general well-being of the child?

R: I go to my mother to tell her things about my child and she advises that we put everything in prayers.

M: Why did you choose your mother and not any other person?

R: I think she is the best person to help me. She's elderly and knows everything with regards to upbringing of children and so she is the one I go to.

M: who else will you go to?

R: I don't go to anyone apart from her.

M: So any time you need advice you will go to her right?

R: Yes.

M: Okay. What are the beliefs and culture of people in this township with regards to children with different forms of disability? Do they have any cultural beliefs?

R: For that I cannot tell. He goes to his father's hometown with him and for them, they are Christians but I do not know if they have any traditions for such people and I have not asked him anything of the sort. Over here, I haven't heard anything of the sort.

M: How do they see children like your child in this town? Do they see them to be normal or abnormal?

R: Most of them do not know that he doesn't speak clearing. When they see him, they assume he is normal. He responds well to greeting but will not speak when you engage him in a conversation.

M: Do they change the way they relate with him when they discover that he doesn't speak well or they still relate with him as though he is very normal?

R: I am someone who doesn't have friends and that is how the child is. It is difficult for people to know. My problem is with his school but I do not take him to friends.

M: With your child aside, how do members of this township relate to other children with these health disorders? Is it normal?

R: I haven't seen that before but I think it is a normal thing. For me I see it to be a normal thing because of my child and I am not perturbed when I see any of such cases.

M: I am not referring to you. I am talking about other members of this town and how they relate with other children suffering from your child's plight?

R: I haven't witnessed any of such but I think it is a normal thing.

M: What about your hometown? Would it have been any different if you lived there?

R: I believe they would have been worried. They would be discouraged from playing with him because he's not as active as they would have loved him to be.

M: Let me give this example; some children are born with very big heads, others do not walk like a normal human being. Let us also assume such a child was in this town, how would inhabitants of this town relate with him?

R: I think people would isolate themselves from him because he isn't normal.

M: Why would they not want to associate with such people?

R: I think when they compare themselves to such people, they are not same.

M: We sometimes hear on radio how such children are sometimes banished. Do you think such a thing can occur in this town or a family can do that?

R: I doubt. We won't do that.

M: I want you to tell me something about, I think you already said something about how he delayed in talking. When did you discover that the child couldn't speak well?

R: When he started talking.

M: How old was he?

R: He was about three years old. I realized if I didn't get close to him, I wouldn't her anything he said. He didn't say his words clearly.

M: Do you think he was born with it or he inherited it whiles growing up?

R: I will say he was born with it because we do everything that's supposed to be done for every child. He didn't really soupy foods so we gave him indomie. We didn't downplay his health and nutrition. I think he was born with it.

M: Is there any member of your family or your husband's family with this health condition?

R: No, I doubt.

M: What do you think you do best for him as a mother?

R: I do not downplay his academics, nutrition and bathing.

M: Why?

R: Because he cannot speak and would be difficult for him to tell anybody when he feels hungry in my absence. Because of this, he is always on my mind whenever I'm away from him. Apart from his dad, I hardly leave him with anyone and when I have to do so, I make sure all his things are well prepared so that he can eat on time.

M: Everyone has a negative side. What do you as a parent think you could have done better but isn't able to do or is lacking with regards to his upbringing?

R: I am an SHS leaver also striving to make ends meet and sometimes not able to provide some things he requests at that moment. I sometimes feel I should be able to provide them at that moment but it takes some time to get them and it's quite worrying.

M: Things like?

R: In his academics and feeding. For instance if he requests for malt or biscuit and I don't have any money on me at that moment, I wish I could get it for him at that moment.

M: With regards to his upbringing; bathing, feeding, schooling, washing and the likes, are the things you struggle to do for him?

R: Bathing and feeding isn't a problem. I bath him and take him to school. In times that I'm not able to go, his father takes him to school. For that, we do our best.

M: I want you to tell me how difficult it is for you to be a good parent to your child. You have said something about money. Is there anything aside money?

R: There's nothing aside money.

M: What about in terms of interpreting his speech, helping him to speak, making his friends accept him and the likes? Is it difficult for you to do these?

R: Sometimes yes. Sometimes I'm able to control them. There are times I find him crying when I come back from errands and it is difficult that way.

M: What about the difficulty in interpreting his speech?

R: That is not difficult for me. He is my son and we sometimes laugh over it when he makes some mistakes. It will be difficult for other people but not me. I correct him when he says things wrongly.

M: You mentioned that he is teased by his friends. Do they beat or cheat him?

R: They really beat him. He comes home to complain about such instances from school.

M: Why do you think they beat him?

R: He is not an active child.

M: Have you informed his teachers about it?

R: Yes I have informed them because his teachers complain that he isn't active in class.

M: Have you informed his teachers about the constant beatings in school?

R: They beat him in the absence of the teacher, probably during break times and the likes.

M: How many times do they beat him in a day or how many times in a week does he experience these? How frequent does this happen?

R: He always complains about being pinched, bitten or beaten. So he brings about three or four of such complains weekly.

M: What do you do when he comes home with those reports of being beaten?

R: I have nothing to do because it occurred in school. I sometimes think such things will encourage him to learn how to be active. But I say sorry and advise him not to retaliate. I calm him down till he smiles. That is because I cannot be complaining to the teachers every other time.

M: What do you do when the incident happens at home?

R: I playfully threaten the children before him and he laughs.

M: Does he perform any duties at home?

R: He doesn't do anything at home because he is not old enough to perform domestic tasks. He wakes up around 8 to 9 in the morning but wakes up at 6:30am during schooling days and leaves for school by 7am.

M: Does he bath by himself?

R: He can bath and wear his shoes but doesn't do them well.

M: But doesn't he perform any domestic chores like water fetching and the likes?

R: No he doesn't. I send him when I need to pick something from the room, which he does but he doesn't wash utensils.

M: How do you correct him when he does something wrong?

R: I don't really shout at him. I threaten him with a cane, something he really fears. So as soon as he sees the cane, he stops whatever he is doing.

M: Can you give me a situation where you had to correct him for a wrong doing?

R: I found him eating sugar that we bought. I was washing outside so when I found him doing that, I shouted at him. I realized he was really shaken by that and it makes it difficult for me to shout at him. I just use the cane to threaten him but I don't beat him.

M: Why don't you beat him? It is biblical to beat a child in order to keep him on track.

R: I get worried when I do something to make him cry because of his condition.

M: How do you motivate him when he does something good?

R: I clap for him when he brings he scores well in his school assignments. I tell him he will be a doctor and he says "Mama I want to be a doctor". And I tell him to pray to God and that he will be a doctor in future and we all laugh and he gets happy.

M: Have you received any training on how to nurture your child?

R: Home or hospital?

M: Either of them. Have you received any training on how to bring up a child; their dressing, feeding and general upkeep?

R: No please

M: What do you think about that, not receiving any form of training?

R: I think I'm doing my best to cater for my child. I don't want him to be dirty. I make sure he is neat all the time.

M: Do you think it would have been helpful to have such a training?

R: Yes, it probably would have been better than what I do for him.

M: Why do you think it would have been better?

R: Because he cannot tell us what to eat, I think if we had training on what to feed him on, it would have improved his well-being more than I provide.

M: Do you have any opportunity to acquire knowledge on upbringing of your children in this town?

R: None that I have heard or seen.

M: At all?

R: Yes please.

M: So what do you think about the absence of such opportunities?

R: I think it would be helpful if we get one in this town.

M: Why would it be helpful?

R: Because for those of us who have children with these conditions, it would be helpful if we had guidelines on what to feed them and the likes.

M: thank you for agreeing we have this discussion Please we will first talk about your home, the people in the house

R: we are two people here and we gave birth to the child on the 23rd

M: you said you are two

R: yes, myself and my husband

M: and who again

R: its finish

M: what about the child

R: yes the child is also part

M: making three

R: yes

M: please whatever question I ask you then you respond to that, now I want you to tell me something small about your child

R: when the child was 2 years, then we realise the child does not talk, so when the child was 3 we took the child to the hospital and the child was checked and they said nothing was wrong with the child. When the child was 3and half going to 4 years the condition was still the same, the teachers complain that my child cannot talk or they seem not to understand the child; I am the only one who is able to understand what the child is saying so I had to take her back to the hospital again and this time the doctor referred as to tamale and there to they check and they said there was nothing wrong with the child and they just wash the ear and they said there was nothing there also. Still the child has problem with the speech and the child mentions some words you cannot hear it well

M: is the child able to hear what you tell her

R: yes, the child hears everything but she can't respond very well, when the child speaks the speech is not clear

M: you said when she talks people complains

R: yes

M: how

R: because they can't hear what the child is saying

M: what are the things that you can say your child is able to do well?

R: whatever the child is taught in school when the child return is does it, the speech aspect is very difficult for her but she does the actions. The child sings all right but the speech is not clear and you can see mistakes in what the child is saying

M: what can you say about the crawling stage of the child?

R: the child was sick throughout the crawling stage until the child walked, the child was 2 years before she was able to walk; it took the child a long time to do that

M: do you think it could be a sickness that made her to take that long to walk

R: we were suspecting that so we took the child to the hospital and they said nothing was wrong with the child until she walked. When she walked the later we realised the child had speech problem

M: what about potty training

R: the child was able to potty trained

M: was there any problem on that

R: no there was not problem on that

M: what about the speech

R: the speech was the problem, because it took the child along time to walk, the speech also took a long time but with what the child speaks now it's not clear

M: what are the things you expected your child to have been able to do at each stage of the child's development that you think it's delayed

R: the child is not active like other children, the teachers says she will be quiet until school closes. Like other children that will be screaming and going up and down, my child will just sit quietly. Maybe because the speech is not clear and the friends might tease her that why she pull herself away from them I don't know

M: how does your child relate with her friends

R: at home she plays very well but she is afraid when they attack her she runs back to me. She does not stand to defend herself she will run crying back to me

M: What types of games to them play?

R: when they are outside like this they play ball but at school he does play, he only play with the children at home not outsiders

M: does your child has other siblings either older or younger

R: no, he is the only child

M: does he play with other children of the extended family?

R: they don't stay close to us, it only one lady who sells close to our house that he play with the lady's child and children in the house

M: who does the child spends most of the time with

R: mostly he loves his father and wants to be with him but when he needs something he runs to me because I understand his speech very well so that i can explain what he needs to his father. The father don't understand his language

M: why does he like his father most?

R: because his father carried him a lot so they bonded so when the father travels it's not easy for him at all

M: with is friend who does he spend most of his time with

R: he does not have a friend except for the children in the house he closes from school at 3pm

M: the children at home whom one does he gets closer to

R: the small girl outside

M: why

R: because that child will still play with him if she understand him or not but the child is older than him

M: does your child has items he can play with

R: yes he has book that contains cartoons that he traces and colours

M: do you think it's helping him

R: with writing I think it's helping him but if I ask him to say after me that is the problem because his speech it's not clear but the writing is good

M: you have already told me that if your child wants to play he play at home but does not play schools because he fears they might tease him

R: yes

M: apart from all these areas when he wants to play where can he go

R: my child does not go anywhere to play except in the house or at a park in front of the house he does not go far

M: what type of games do they play on the park?

R: football

M: if you need advice about your child on his health, physical or general care who do you do?

R: I go to my mother and she said we should pray about it that will rather help

M: why your mother

R: she knows how to take care of children more than myself so things in relation to the care of the child I go to her

M: Apart from her will you go to someone else

R: no

M: if you need an advice you will go to her

R: yes

M: are there any cultural practices or belief in relations to children with special needs like your child

R: I can't tell, since I gave birth to him his father goes to the village and come back and I have not ask him if there are any cultural customs we need to perform because we are Christians and here I have not heard of anything like that

M: when community members see your child and other special children how do they perceive them?

R: most of them don't know he has a speech problem, when you greet him he will respond very well but he can't make further communication with you

M: so when they realise it how do they relate to him

R: personally i don't have a friend so it my child and i think am there with him they are not able to do anything

M: lest move away from your child how do they treat other children with special need

R: I have not seen one before but I believe it will be a normal thing

M: what about if you had the child in your hometown

R: i believe it will worry them because they way they think he should act he is not doing that

M: look at this scenario, maybe in the community they gave birth to child with a big head, or mentally the child is not sound or can't walk well, and that child is in this your community. How will people relate to that child?

R: i think people will not get close to that child because the child is not normal it will be difficult for a lot of people to get close to that child

M: why do you think people will not get close to him?

R: because the people think they are not the same with that child

M: we have heard from the radio that such children their parent perform some rites and send them away

R: i don't think so

M: can some family do that

R: i don't think so

M: when did you realise the speech was not clear

R: when the child started talking

M: what age

R: 3 years going, if i don't get close to him i can't really hear what he is saying, if he want something he does not mention is clearly you will hear the sound in his nose

M: do you think he was born with it or it happened when he grew up

R: I will say maybe he was born like that because when he was born whatever a child needs he was provided. He was a child who did not like soupy meals but rather indomie and Milo and with what he likes we provided for him

M: as a parent what do you think you are able to do very well for him?

R: in his learning, feeding, bathing i think am doing very well with that

M: why

R: because i have realise he is somebody that I can't see he can't talk, if he is hungry and am not close by he can't go and tell anybody because of that when am somewhere all my attention is on him if he has eaten or not so when am going out I don't leave him for anybody except his father or prepare his meals and pack it with him before leaving him with someone

M: what are the things you wish to do for your child but you are not able to do as a caregiver

R: there a lot that am not able to do, I just completed SSS and am looking forward to continue and sure there is something that my boy will need but we will not be able to provide that very moment; it worries me a lot on that

M: what are the things that he might request for that you are not able to provide that moment

R: with his schooling and feeding example he can say mama I want malt or biscuit but I might not have at that moment to provide for him

M: let's look at his feeding, bathing, schooling, general care, are there something that we wish you could do for him but you are not able to

R: am able to do, with washing of his things everyday I wash his things, I bath him and take him to school, if am not able to take him his father takes him, we are doing our best there

M: apart from money to buy something for the child, is there anything that makes it difficult for you to play your role as a caregiver

R: there is nothing like that

M: let say ability to hear what your child is saying, help your child with his friend or accepted by his friends or bully him, do you find some difficulty in that

R: it's difficult I have to always talk to them not to tease him, if you step out to buy something before you return he is already crying

M: what about the ability to understand what your son is saying, do you find it as a challenge

R: personally i don't find any difficulty because he is my child i understands him but am sure with another they will find it difficulty

M: apart from the teasing do they beat him also

R: they do beat him a lot at school when he comes home he will tell me this and that person beat him

M: why do you think they beat him?

R: I think because he is not active type

M: have you reported it to the teacher

R: I think it happens at break time or when the teacher is not in the class

M: How frequent is the beating or bullying

R: in a week 3 or 4 times

M: when your child brings such complains what you do

R: I don't do anything because it's a school and also i feel their actions will make him active and i cant always go and give complains to the teacher, i just console him and tell him they are his friends

M: what about the home when it happens what do you do?

R: with that one I warned that child in front of my child so that he will know i also don't like what they are doing to him

M: does he has any household chores that he does

R: no, he has nothing doing because the time is not yet right for him to do any chores

M: what about bathing himself and dressing up

R: he sleeps up to 8 am on weekends by 6:30 am on week days so that by 7am he is ready for school, he can bath himself but with the dressing up he is not yet perfect I have to go and help him out

M: what about picking something for you

R: with that one he does it but after eating i don't ask him to wash his bowls

M: how do you discipline him when he goes wrong?

R: I don't discipline him, I just frightened him with the cane, he is afraid of the cane so when you raise it then he stops whatever he is doing

M: can you give me of an example and he did something and what you did

R: we bought sugar and i was washing outside and he was in the room eating the sugar so i shouted at him and used the can to frightened him

M: have you beaten him before

R: No

M: why are you not beating him because the bible says spare the rod and spoil the child?

R: with his condition I don't want to beat him when he cries it worries me

M: how do you reinforce positive behaviours in him?

R: when he comes home with good reports in his books from school I clap for him, and i will say my son will be a doctor and he will respond mummy I will be a doctor and i will say pray to God you will be a doctor in the future

M: have you received any teaches on how to take care of your child or children with special needs with regards to feeding, general care

R: No please

M: now that you have not gotten any teaches on it, how will you rate yourself?

R: I can say am doing well, I try to keep him neat

M: do you think if you had that training would it have been helpful to you

R: yes because maybe I *would* be able to do more for him than what am doing now

M: how

R: with respect to his feeding since he can't talk I would be able to give him the type of food he needs to eat that what am doing now

M: do you have community support with respect to mothers who have special children

R: there is nothing like that

M: since there are no such support in this community what do you think if you had such facility here

R: it would have been very good because parent with such children can go and be taught on how to treats, feed and care for their special children

M: thank you very much for your time end time 7:06am.

M. **All that we are going to say here will be recorded a when we get to the office we can write it out without living any conversation.**

Now can you tell anything concerning your house like the number of people you are in the house you and your husband and the children?

R. As for me an not coming from here or have not stayed here for long just that after I got pregnant for him that I decided to come and stay with him to deliver.

M. **But now that you are here with him for someday now you should have something to say, that is what you experience or witness so far in the house?**

R: My boyfriend is here with the rest of the family members and my child.

M. **can you tell something about your child and yourself?**

R. After I gave birth the child was alright can speak but for some time came and the child got sick which resulted into a lot of problems like can't speak, walk, see etc after a lot of visit to hospital it became better by God's Grace.

M. **Hope now the child can do a lot of things by himself, name them?**

R. Can see, walk and go to school, speak alright after school can prepare food to eat, bath, fetch water e.t.c.

M. **Now that the child is grown, have you seen any changes so far?**

R. Yes, like speaking, thinking and doing good things is a great change.

M. **How do your child relates to other children in the community?**

R. They play together but when she speak the find it difficult to get him clearly, she can see all they do and repeat some to the rest.

M. **The children in your house is there different way they relate to him and the outsider.**

R. They live peaceful like how he live with outsiders no difference.

M. **Your child whom is she closer to in the house, you or the father?**

R. I can say she's closer to me than any order person in the house.

M. **Why you alone in the house are you the only perform in the house?**

R. Just because after she get sick for long and am the mother and always backing him here and there for sure she should be closer to me a that I say and demonstrates she gets me right. After gave birth I stated schooling but because of his sickness she goes out to places his not suppose I have to stop to take good care of him till it becomes better.

M. **What and what can you tell me the child normally use to play with?**

R. Milk and tin tomatoes cans are some of the things plays with and sand.

M. **Where usual does she goes to play?**

R. Under the mango trees

M. **Is there any other place you observe she goes to seat and play with?**

R. Yes, for example when she gets into school compound and his happy she stays out side alone and play that she wants to cook with the sand.

M. **When things are getting difficult when do you go to for a help?**

R. I go to her father and grandfather for a help.

M. Why not any order person but only she child's father and grandfather?

R. The child belongs to them.

M. In this community if you give birth to a child what do they do for or at the child.

R. They decide for a day to shave, name the child and outdoor for the family to see.

M. What taboos are there for the child, like what to eat and what not to eat?

R. They don't eat rabbit, here is a particular day I don't go to farm and Sundays to.

M. Special children or disable children what do they do to or do for them here?

R. They always take special care for them like bathing leaves and hubs and consult oracles e.t.c.

M. Let say our parent always consult gods and say this child is our grandfather/mother who is back, he/she is a tree, gods/Fetish priest, not up to date before he/she was born etc. What taboo or how do they take care of them?

R. They will take care of the person, for example they will consult the god's and know that he/she is not good, so they take child out of the family. Others too they have to just pour libation and bath him/her with special dust and leaves. For others you only worship the person to keep him alive.

M. Now tell me how your child sickness happened up till date.

R. It was in the night it happened the child can breathe in a form of epilepsy and we seat him o hospital the kept oxygen on him for about four days before we were trying and forcing him before she grew abnormal fatness, cannot eat, see walk e.t.c. if the inject him he don't fell anything unit for some time through constant visit the hospital and our hubs and the leaves we bath him it became better. He can walk, eat, bath, see, but talking fluently is the problem.

M How do you take care of your child as a whole?

R. In the morning I will bath brush his teeth, cook for him and he will go and play, in the afternoon a give him different food till night.

M. What where you suppose to do for him which you coun't do so?

R. A lot, I would have wish o by what I know is good for him but I can't

M. **Hope is all about money you don't have, like cooking different food, buying different clothing e.t.c.**

R. Yes, because I wish he eat something different better all the times.

M. **So you what difficulty or challenge do you face just because you child is sick or disable till now?**

R. If not now first I make sure his always closer to me either than that I have to go round looking for him till I see him where I don't expect to see him, because he behaves different form order children. I don't feel like crying I think a lot some times. But for now is better in all areas of his sickness.

M. **Being with his age mate playing does he come to the house to complain either the beat him or say something bad about him?**

R. Yes, he knows some of them their names and comes sometime to say A or B beat or insult me like or that.

M. **Do you advise him that if you go out behave well or else they will beat or insult you, how do you do that?**

R. Yes, I advice when him not to take what do not belongs to you or say bad things to people you are living with.

M. **As he stays with you in the house I hope helps you to do things, like?**

R. Yes, fetching water, carrying and cleaning of cooking pots e.t.c.

M. **His just like any other child how do you displain or advise him when he do bad things.**

R. I beat him when the bad things he does are too much but sometimes advise him not to do that again.

M. **Just mention one thing he did badly**

R. Spoiling of our drinking water by dropping bad things into I

M. **You said visit hospital several times, what have they said do to or for the child I actually help the child a lot.**

R. We have traditionalist which can tell you don't let him eat this or that for some period and Christians to have theirs, like fasting and prayers.

M. **Traditionalist and Christian all the advice giving you have it help you?**

R. Yes, I can say it did help because we actually obeyed their says it's now better for the

child life. For example a man told me to cook a goat tongue for him to eat and in did it help in the speaking area.

M. In this your community do you have some where the take care of this kind of children?

R. No, I don't think so.

M. I thank you, I can see that you were busy doing work but you have listen to me and we discuss a lot.

M. Which network are using

R MTN but my phone spoiled

M. So now I want to give you MTN credit of GH₵5. what do will you do? Are going to sell it to someone?

M: My name is Seidu Latifatu and am having an interview with parent with special child age 0-6 years. Am in a district in the northern region and the district is Tolon district and the community is Tali. Today's date is 22nd April, 2019 and the time now is 9:56am.

M: Please can you tell me a little about your home? That is who are you living with, how many of you are in the house?

R: The parents of my husband are staying in Accra so is myself and my husband who are in that house

M: With your kids?

R: I have only one kid who is Sayibu

M: Can you tell me a little about Shaibu? How is he like? How does he react when he gets angry or not happy? Just tell me a little; is he still breast feeding or not and what does he like?

R: He is still breast feeding

M: So what does he like?

R: He eats TZ (Toun Zaafi) and rice

M: What are his dislikes? And what food does he not like?

R: He doesn't like TZ but rather prefer rice

M: What have you observed in terms of his developmental stages, did he crow early or any other thing you observed?

R: He did not crow early because we did not seat him early enough to pick up and that made him not to crow early. Also the delay in his ability to walk too is due to constant illness.

M: I see! What sort of illness is these?

R: He vomits and ran diarrhea always and when it happens that way his temperature will rise very high.

M: Does it occurs frequently?

R: Yes and we usually sent him to the drugs store

M: Have you ever sent him to the hospital?

R: No

M: Why? Don't you have a clinic in this community?

R: We have a clinic

M: So why don't you go the clinic?

R: (No responds)

M: Where does he normally defecate?

R: I sit him on a chamber pot to defecate

M: How did he learn how to sit on the chamber pot? How months old was he when you started sitting him on the chamber pot?

R: He was over six months when I started sitting him on it

M: Has he started speaking?

R: He can only say mma (mum)

M: Apart from the problem of his inability to walk what else is his problem?

R: His major problem is just his inability to walk but for the talking all his age mates are still not talking

M: So the major problem is the inability to walk? Ok

M: How is your relationship with Shaibu? Like; do you play with him, does he laugh? Or do you beat him?

R: We do play and he laugh's a lot

M: Does Shaibu becomes happy when he sees you?

R: Yes he is always happy when he sees me

M: Are there siblings or other children with Shaibu in the house?

R: Currently he is the only child at home and the other children are staying with their grandmother at Tamale

M: What about the children in your neighborhood? Do they associate with Shaibu?

R: Yes they always play and associate with him

M: Who does Shaibu always found with or like to be with?

R: He always like to be with me

M: When you consider the time he usually eat, breast feeding or defecates, is he always with you or with the friends?

R: Well, normally he is either with me or with the father

M: I want to know why he is always with you.

R: Because he is used to me. Even if someone try taking him from me he refuses and prefers to be with me.

M: What do Shaibu like to play with or what objects does he use for playing?

R: He uses a doll for playing

M: With what again?

R: That is the only one he uses

M: Doesn't he play with sticks or empty tins or…?

R: Yes, he play with those things too

M: You said he plays with the doll, sticks, empty tins and what else?

R: That is all

M: Can you remember any other thing?

R: No

M: Please I will like to know if you have some difficulty in handling Shaibu do you have someone to consult as what to do?

R: Yes I will consult my grandmother

M: Why will you consult your grandmother?

R: Is because she is an old woman who has given birth and groom a lot of children up to that stage she will be in a better position with that experience to advise me as to what to do.

M: What is the tradition or cultural practices in this community after a woman has given birth? What do they do from infancy till the child grows?

R: When it is a male child, the practice is that he is circumcised the third day and if it is a female child, her ears will be pierced on the third day before the naming ceremony.

M: So what happens during the naming ceremony?

R: The baby will be bath and the hair shaved

M: Who baths the baby?

R: The traditional birth attendant normally baths the baby

M: Have you ever seen how the bathing is done?

R: Yes, she use soap and a sponge with water for the bathing and also spray the water on it very well so that the baby will be strong.

M: I want to know if a child gets to the age like your child and cannot still walk how are they treated in this community?

R: They are normally sent to the traditional herbalist

M: To do what?

R: For treatment and herbal concoctions for bathing of the child

M: So have you gone for the herbal concoction for bathing of your child?

R: Yes I went for the first one and I was asked to come for the second one but am yet to go

M: How many days did you bath him?

R: He was bathed for seven days

M: Ok

M: Now if a child gets to this age like yours and can't still walk how do people perceived them in this community?

R: Pressure will be put on to send him to the traditional herbalist for the concoction for bathing so that he can walk.

M: Do people shy away with such children or say nasty things about them or see them nauseating?

R: Yes they do

M: What do they say?

R: They insult and say things like you are a cripple, all your age mate are walking and you can't walk

M: So that is all what they say?

R: Yes

M: So can you tell me how he got to this stage and can't still walk?

R: The problem is that we didn't bath him the herbal concoction early enough that is how come he got to stage and not still walk

M: So in this community when someone give birth….

R: You see the issue is that when I gave birth I went and stayed with my grandma in Tamale and there is different. So it was when we came back that I got the opportunity to bathed him the herbal concoction

M: So Unlike Tamale, here is norm that you must bath them?

R: Yes, and is two weeks after the naming ceremony which will make the baby develop very fast.

M: How many months did he start crawling?

R: He started crawling in the seventh month

M: And up to now he is not walking?

R: Yes

M: Do know or aware of any child either in your family or from the family of your husband who is over a year or more before they can walk?

R: I don't know of such a child

M: So he is the only one with such condition?

R: Yes

M: Please can you tell me about some of the things you are doing well as parent of a child who cannot walk?

R: I always pay attention to him and also visit the herbalist for more treatment just to see him walk

M: Tell me some of the things you know you supposed to do and you find it difficult doing for Shaibu?

R: I wish I could give him a tea every day but I can't do it because don't have.

M: So what does he eat?

R: The day I have some money with me I do prepare tea or "koko" for him to take

M: So I want you to tell me a day when you don't have money and your child wants to eat what do you do?

R: I will tell the father to give me money to buy and prepare for him

M: I want you to tell me how you train him and what you find difficult about him

R: I don't find anything difficult with him

M: How do you prepare him against teasing or any other thing that will not make him happy from other children in the community?

R: Because he is still little I have not experience any of these

M: Ok

M: How do you discipline him when he does something wrong?

R: I will shout at him

M: What again?

R: I will shout at him and asked him to stop what he is doing

M: Have you been sensitized about dealing with children with disability?

R: No I have never been sensitized

M: Children like Shaibu what sort of support is made available to them in the community?

R: They is no support for them in this community

M: We are done, thank you for your time

Transcription Sheet

M: I am having an interview with a child with special needs. Ok madam, we are about to start I will plead with you to plead with you to speak up. I want you to tell me about your household, who is your husband and how many children you have, that is what I want you to tell.

R: I have three children

M: You have three children, how old are you?

R: I don't know my age

M: You can't tell me your own age, ok. How old do you think you will be like, in your view?

R: I will be like 20

M: This is your husband, right, how many wives does he have?

R: One wife

M: Ok, you are the only wife

R: Yes

M: I will tell him to add someone

R: Laughed

M: So, you said you have three children?

R: Yes

M: How old is the eldest?

R: Six years

M: The next to the elder?

R: Four years

M: We are here to speak about Hikma, how old is she?

R: She is four years

M: So, if I should ask you to describe Hikma to someone who doesn't know her, how will you do it?

R: To describe her as in how?

M: Like some asks, who is Hikma, what will you say?

R: Her size or age

M: Everything about her. You said earlier that she is four years?

R: Yes

M: So, is she fair, her character, dark or how is she like?

R: She is chocolate in completion neither fair nor dark in completion).

M: How is her health condition like?

R: Truly, I can tell about how her illness is?

M: Looking at her, it is like she has challenge in walking, meaning she can't walk?

R: For the walking, she finds it difficult to walk, it is this week that is able to walk small small. You see what we have there, that is what has aided her to be walking small small. When she was a child, she was calm.

M: When she was growing up, what has been her condition before reaching this stage?

R: Truly, she was plump when young; we didn't observe any sickness with her, so, we don't know whether she was born with or she got it after birth. Truly, I can't tell when she had the sickness

M: You don't know how she got it, whether born with it or got it after birth?

R: Yes

M: Did she crawl, when growing up?

R: No, she never crawls, only move by dragging her buttons on the flour

M: How does she relate with her peers, let say playing together?

R: Truly, she doesn't often play with her peers. If she often sits at one place and observe her peers while they play.

M: We are referring most of our questions to you, Mama XY (name of respondent) because you are always home with the child than with your husband. So, please tell what happened when she was growing up, at what age your thought she would have crawled, and she didn't or at what age you thought she will walk and she didn't, that is what I want you to tell me?

R: I thought she would have walk at age one, but it didn't happen

M: And, she didn't walk up to age four?

R: Yes

M: You said, she started walking small, this week?

R: At least child should walk at age one going to two, but we didn't observe it in her

M: What about her easing or going to toilet how is it like?

R: She sits on chamber pot to go to toilet; else she finds it difficult to do it.

M: What about her speech?

R: She doesn't speak. She tries to do it, but always have difficulty in doing it. However, when you say something, she understands

M: in your view, at age four she should have been speaking, yet she isn't. So, she only murmurs?

R: Yes, she tries to say, "[*Mba* (father) or *Mma* (mother)]. She does that by pointing at things

M: What are things you think she should have been doing at her age which she isn't up till now?

R: At her age, she should be walking at her age and be in school, because most of her age groups are in school

M: I wanted to know, what your think she should be doing at her age which she isn't doing.

R: At her age, she should have been in school to learn something, at least counting

M: What of her life, at her age she cannot walk any other challenges that she has which you will like to share with us?

R: Also, we said her health is challenge which we have tried using local herds and other concoctions which she tries to stand a little.

M: You treated her the traditional way?

R: Yes

M: Never sent her to a health facility?

R: No

M: Ok.

R: It was a church group which came to visit her…for the health facility, we send her when she is ill.

M: Hmmm

R: Then, they give us some medicines to be given her at home…they also give her food being given to kids at the facility for us to feed her. Sometimes, when she is given food, which is hers, she hardly eats it, but when the kids are eating and she grabs eat, she eats it.

M: For, this question, let me direct it to the mother, how do you relate with your daughter?

R: I and the child?

M: Does she like being with you?

R: Yes

M: Or she likes to be with her peers or being with others?

R: She often sits with her grandmother than her mother, sometimes she cries to be with her friends, so they pick her to be with them outside. We only pick her from her friends when she cries. She doesn't quarrel with her friends, she only sits and get some empty things and play with it.

M: So, she prefers being with her grandmother than mother?

F: Yes

M: What of being with father?

R: The father doesn't often stay with them; he lives in his uncle's house.

M: What of his relations with his peers?

R: For now, she plays with her peers. As she sits, when they come out to play she will try and join them to play.

M: While sitting with her friends, she isn't scared about them?

R: No, she isn't scared of her peers

M: You said, she isn't speaking

R: Not at all, she speech is always father, mother and doing some gestures to accompanied it

M: You said she likes to be with the grandmother than the mother, or with everybody?

R: As I (father), sit here all of them prefer to be lying on my labs.

M: Hmmm, but she is always with the grandmother

R: This is the grandmother

M: Why does she like to be with her?

R: Because, she is always with her. She has a younger sibling, who is always with the mother.

M: That is why she is not closer to the mother?

R: Yes

M: What does she like to play with?

R: She likes to play with empty tins, and bottles

M: What type of play does she engages in besides using empty tins

R: She doesn't do anything else, ok she plays *ampe* (a local game which goes with jumping and clapping), but she does clap while sitting on the ground

M: Any other game, she plays?

R: No response

M: Apart playing in the house where else does she plays?

R: She goes out and plays with her peers

M: Does she go out by crawling?

R: Yes, and sometimes she drags on her buttons to meet her peers outside. They often play under a tree. When she gets to a place, they carry her.

M: Ok!

R: If not under the *nim* or mango tree. Sometimes, her peers will live her and later come back and play with her.

M: Assuming you need knowledge on Hikma, where or from whom will you seek for that knowledge?

R: As I sit, here my parents haven't sent me to any formal school; they only sent me to farm. I can only teach someone how to weed on a farm. In this house, all my children are in school. So, if she recovers, I will get her enroll in school and come back to my farm.

M: My question is that, if you should need knowledge on how to take care of a special child like Hikma, where or from whom, do you think you can seek knowledge from?

R: I hear people say, there is a school for physically challenge where they teach them vocational skills, but we don't have it in this neighborhood. Since, we don't have it here, the only place to seek for knowledge, is to send her to the school. If you want her to learn some skills, you enroll in sewing apprenticeship.

M: I am asking of the knowledge to take care of the child

R: That is what I am developing

M: Ok

R: Put her in sewing apprenticeship or carpentry or mechanics.

M: Hmmm

R: For this child, if we should seek for any knowledge about her, is to send her to school and when she returns, she can be enrolled in sewing apprenticeship because we don't have any other vocational skills in this neighborhood.

M: Hmmm

R: That is the only vocation in this area. It was in recent time they brought weaving in this neighborhood. Other vocations I am aware of are hairdressing, but they are no more. So, if you don't have enough vocation in your community, how do you select the appropriate vocation for her?

M: What we want to understand is, where you as an individual will go and seek knowledge on how to take care of a special child like Hikma, and not going to school to learn?

R: I you referring to me?

M: Yes

R: If I should seek for knowledge, which is a skill like, I will be able to learn it. If I should say, I will go to school and learn something; I will deceive you because my responsibilities are more. I can't under anyone.

M: Earlier you said, when your daughter wasn't able to walk at birth, you tried using traditional method on her medication

R: Yes

M: At what age was she did you start her medications and how did you go about it?

R: In this household, if a child is about three to four months and the child is healthy, I will introduce traditional medication on that child till they recover. Beginning from the child's crawling till they walk. So, with her case, I tried medication on her and never saw any improvement on her and tried elsewhere.

M: Was that place to a traditional treatment?

R: Yes, I tried several times, yet no change. I stopped because she is a girl and if she should continue with the herbs, she might develop a character which will affect her and I will receive several complains because she will be quarrelling with her peers.

M: Meaning, if you should continue treating her in the traditional way, she will end up developing different habit?

R: Yes, if you allow more herbs in her system, she will develop a character, you will never cherish.

M: It will let her become aggressive

R: Yes

M: Ok. In this community, what are your belief systems towards children with special needs, example epilepsy?

R: It is heredity, if you have it in your family; you can treat and will never find its cure. Some also get those sickness from bad breathe, emanating from evil spirit. Or someone might also get infected of epilepsy when they come in contact with the victim, but is rare. Some individual can get infected when they inhale a bad breathe from an epilepsy patient.

M: Ok. That is what you meant at the bad breathe

R: Yes

M: Some are in the blood of the family

R: Some are heredity because in is in their family and anyone in the family has it. Every child is likely to have convulsion, but the white man has done well with the introduction of

convulsion immunization which heels the illness. If a child has a polis, you have to send the child to hospital for an injection

M: Ok

R: When the child is brought back from the hospital, we also prepare some herbs to bath him.

M: Such children how are they treated?

R: If you are not affected with convulsion, you will not know how it is. In this household, there is one child who was infected with convulsion, and got treated. Every child has convulsion, but because of the treatment at the health facility, it is treated. His convulsion revealed itself when we returned from farm. After he has put his luggage at a place, he had it. So, I rushed him to hospital. We had a first aid, which is a concoction from our elders; we squeeze it into his nostrils and get some water from a calabash, and bathed him before sending him to the health facility. Now, we rely on the health facilities because most of our children don't know the herbs

M: What of if the child is not affected with convulsion, but has challenge with vision, or hearing.

R: Hmmm for hearing impairment, I haven't observed what they do about them, does it have treatment or not, that, I can't tell.

M: Hmmm

R: There sometimes people delivered with such challenges

M: What do they do with children with such challenges?

R: For now, it is those who can read, are able to tell what steps to take. Some children are likely to have challenges with hearing when they had headache after birth. They slightly hear what people say. A name was mentioned as an example. Just delivery the child had headache and it affected the hearing.

M: How do treat children with hearing impairment, traditionally?

R: Like I said, we don't have treatment for such conditions

M: You don't have any treatment for it?

R: Yes, what we have treatment for is sinusitis or tension heacache that affects the eye. We have traditional herbs which we can eat, drink and bath as well, God willing you will get well.

Sinusitis or tension headache, s like worm it may affect someone and they get the medicine and inhale it and get recovery early.

M: What of epilepsy, do you have any treatment for it

R: That is what I said, I cannot say much about it.

M: How do we care for children suffering from the illness I have mentioned?

R: Epilepsy takes away the senses of the victim. So, if the child is not in school or farm, we give the epileptic a chicken for them to send it elsewhere to perform some rituals. If in case the illness affect an individual on the farm and none is able to rescue him and the sun scorches on the victim without anyone rescuing him, it might end their life. Also, if it is in the raining season, it can end the life of the epileptic. However, the epileptic has to provide feeds for the chickens used for the rituals. But, if the epileptic goes to provide feeds to the chicken and hasn't returned, those around him have to make a follow-up.

M: Hmmm

R: For epileptic, there is a ritual they perform for them.

M: Ok!

R: If the sickness resurrects, the rituals expected to be done for them is important to comply with because their condition was ordained by God. You will not say because it is a special condition therefore you will neglect them or support them. It has not help than to adhere to the norms of the rituals.

M: Why don't we assist them?

R: If you do assist an epileptic! Everybody and their household issues, you are an individual just like I am. The epileptic was created by God as you, and put them in that condition, why are you trying to doubt God?

M: Ok

R: Even, enemies can let someone suffer from such illness or even kill them and they always try to neglect them among a crowd

M: In this community, how it is?

R: As how, what specific is, can you explain further?

M: How does the community take care of children with special needs?

R: Some are scared of children with special needs. For instant a patient suffering from epileptic can fall in a house and everybody will run away from the sick. Also, some will also take the patients when they fall, we have observed that.

M: Hmmm

R: But, I cannot speak for every structure in the community because I don't visit all structures in the community.

M: Ok.

R: To know what happens in each house and say how they treat children with special needs.

M: Meaning it household specific and how they respond to children with special needs

R: Yes, like I was saying, traditional herbs have been used in some cases to cure epileptic. Because some don't like oily food, and if you realize it and stop eating oily foods, you will be recovered.

M: Ok

R: Hmmm, not every oil, but shear butter. A disease mentioned it doesn't like she butter, so if you are infected with it and take shear butter it brings out some boils on your skin.

M: Hikma mother at the beginning said she didn't know whether she was born with her condition or not?

R: Hmmm, for that one no entity can know tell how she got her condition. Anyone trying to tell us the source of her illness will be a deceptive. Not that she was born looking sick, she was healthy at birth. All of us got to know she was ill when she reaches stages in her development and we could see any changes in her. Then, we started treating her with herbs

M: How old was she?

R: She will be around one year three to four months. Ideally, around that time period a child should start crawling, which we didn't see.

M: Hmmm

R: Then, started bathing her with herbs, which made her to start some movements till she is able to sit. Our concern is how she can walk. We travelled to a village to get some other herbs which she used and was able to crawl

M: At what age was she?

R: She was above three years

M: When she started crawling?

R: Yes, we finished eating our supper and I was contemplating when this girl will crawl, low and behold, she starting crawling. At that time, her peers started clapping for her. Since then, she started crawling small, small.

M: Things that she isn't able to do, when she start it, her peers clap for her to motivate her?

R: Yes! So when she started crawling small, small, she likes dragging herself on the flour. It is about a months when he younger siblings started standing up and that motivated her to also starts standing up. This week she started walking small, small.

M: You told me in this household, she is the only child with special need?

R: Yes, we have never had one.

M: What of Hikma's maternal and paternal family, anyone suffering from such conditions?

R: None from her paternal or and maternal father doesn't have such condition. But, I cannot tell for her entire maternal family whether they have suffered such condition.

M: Hmmm! What in your view will you say your household is doing its best in taking care of Hikma, since she is a child with special needs?

R: Hmm! You people want to know much about my house eeen! With Hikma, we are doing our best with her health condition so she remains healthy. We allow her to eat well our main food as Dagombas is Tuo Zaafi (TZ). In the morning she takes her porridge. That is what we feed her every day. It is the white people who advice her to minimize her intake of TZ and provide us with some feeds we prepare for her. Whenever, we prepare it for her she rejects it and eat our normal typical mal, TZ.

M: TZ

R: Yes, if they should serve me my TZ now I will be struggling to eat it with her.

M: What are the specifics feeds she is given?

R: I said …something in a tube and type of powered grains used for porridge. In this community, we don't often get feeds. If you go to the health facility, they only give us some small amount of food. In this community, you cannot have access to feeds for your children.

M: Like Hikma, with her condition, what do you think is necessary to be doing for her, towards her upbringing?

R: Truly, at the health facility whenever we sent her for weighing, they teach us what we should do to make health. But, if we try to heed to their advice, after three days, we are unable to continue.

M: What specifics are you asked to do?

R: They advice us to feed her with green vegetables, and bones and soupy foods for her to eat. Also, she should be fed in hygienic conditions. Wash her hands before they eat. On daily, basis she should be thoroughly cleaned. We receive those advices from the health facility. However, when we come home and start doing it, owing to financial constraints, we aren't able to continue with it.

M: Which ones do you have challenge to continue with?

R: Don't have challenge with cleaning her, but the diet to feed her is the challenge because I have to buy. If I try to do it, after three days I am unable to continue. In the north, if say we have a challenge; it is because we don't have work to do

M: If you have a child with special needs, what challenges do you have in bringing them up?

R: The main challenge is finances to get her needs. If you have the finances to adhere to her needs for a speedy recovery

M: Tell me some specifics of those needs?

R: That is what I have enumerated to you. You have to buy this and that. I don't have any guinea fowl to sell, what I have is corn and that is what we are fed on

M: Ok. Earlier I asked about how she relate with her friends. Also in this house I think they don't mock at her because you are one people

R: No

M: Do they mock at her outside?

R: For outsides, they don't mock at her, rather play with her. Those who do that are her grandparents

M: What of her friends

R: No at all

M: Apart from the house and outside, where else does she play?

R: She doesn't go anywhere, unless her friends want to carry her to play. Her grandmother used to sell along the roadside and she carried her along.

M: Around that place none was teasing at her

R: No, just playing with her and those who do that are her grandparents

M: How do they play with her?

R: Do you have grandparents and uncles

M: Yes, I do

R: Don't you play with them?

M: I do, as you know playing with someone is in different categories

R: In our Dagomba culture, we have playing mates among kinship and it is always encouraged. God created the world and added playing to be part us. So, with such kinship play, it you misconstrued it and become angry because you are playing, one might die.

M: What of using my nature to mock at me and me become angry?

R: Oh! In our culture, it is normal if someone tease at you with it. It isn't sinful because it is part of you relations. God asked you to play among yourself and never said, you should quarrel while playing, if you quarrel while playing, then you argued with God.

M: Ok

R: If you are angry anytime they play with you, will any of your relations play with you again, your uncle's child or grandparents will not play with you again. If that persists, it breaks the family. Hope you have understood my explanation?

M: Yes, I do. Hikma is still young, do you allow her to do any chores?

R: Her chores are to bring this and that is all. As we sit here and her mother's mobile phone rings, she will rush and bring it. She drag herself and bring it, saying Mother, mother.

M: Is she able to carry her younger sibling?

R: What! How can someone who is not strong carry a baby? But, when they are seated, they play together.

M: She doesn't do any chores at home?

R: Not at all, maybe moving forward

M: How is Hikma counseled, if she does something wrong?

R: If she does something wrong, we advice her not to repeat it, else she will be lashed, hence she will not repeat it

M: Hmmm!

R: If a child does something and you caution them that if they should repeat it you will lash them, they understand what they did was wrong.

M: You said she doesn't speak

R: Yes

M: But…

R: She is able to pronounce mother and father. She even request things by pointing

M: Meaning, you advice her by speaking with her

R: Yes

M: She listens while you speak with her

R: Yes, whatever you say, she listens. He challenge is speaking

M: What does she do, for her to be beaten with the hands?

R: Like I said, if she eases on herself without telling the mother, she is sometime beaten. Also, if she throws things kept for the household about, she receives some beaten

M: Besides, the beaten, and advice given her, another form of upbringing

R: No

M: Apart from the advice received from health facility, have you received any advice elsewhere on how to bring up your child?

R: Nobody will invite you. We only receive the advice at the health facility when she is ill and we visit it.

M: Unless you visit the facility

R: Yes

M: in this community, do you have any support given to children with special needs?

R: Unless, what I told you, if a child unhealthy, they give them some feed in a tube to feed them.

M: Hmmm.

R: They do that often on Mondays. Even with daughter doesn't like eating that food .She will never eat it when we come home. If she should eat small portion of it, she will be having running stomach. She will be having continuous toilet, making me have some fear in me.

M: Ok.

R: So, I have got her some medication to cease the toilet.

M: Thinking about the advice you received at the health facility, is it helping you?

R: Yes, it helps

M: How?

R: When you adhere to their advice for the few days, you observe some changes

M: Why do you think so, or what specifics have you learned at the facility which you didn't know?

R: They will teach you how to take care of your child to be health. You don't need to leave your child to be in filth. They teach us about hygiene. It isn't everybody who heed to their advice .I always follow their advice and whatever I am ask to buy and feed her I do it and if I don't have I ask the father for his assistance .

M: But, in this community, they don't have assistance for children with special needs?

R: Not at all.

M: Don't you have any school in this community for children with special needs?

R: No, we don't have a school in this community. What I am aware we have in this community is a health centre which does well with immunizations children against polio and CMS. They will travel in all structures to immunize. They announce it to the public to it. That is what we have in this community. But, if it comes to food for children with special needs, when you go for it today, the next day you will not get it.

M: Ok. I really appreciate your time. So this has been an interview with a parent of a child with a disability. The interviewees were the child's mother and father.

M: Please Sir good morning, please can you tell me your name and a little about your family or your home here?

M: Please tell me a little about your child the one who is not well?

R: She was 10 months she could even call me Dada, when she was10 months she holds you, gets up she crawls and she use to disturb a lot, in the morning she always wake us up, she will disturb she will go and touch this you will tell her to stop she will go back to it she was very stubborn, then there was a day I was not at home, then according to my wife she had temperature, and then she was sent to the polyclinic, they tested her and she didn't have malaria, they prescribed parcetamol syrup and some other drug, so she called me on phone and told me this so I asked someone to buy the drug because the polyclinic did not have those drugs, so I asked someone to buy the drugs for her, then when I came later, I came and saw the child she was.

M: please what are the competencies you observed with her development?

R: Because she is still a little girl, her competencies will be much of a problem because even though she is down but she is still very active.

M: So does she crawl?

R: No, she makes a lot of noise.

M: But no noise comes out?

R: No.

M: Just noise but you don't know what she is trying to say?

R: Yes, just noise but you can't hear what she is trying to say.

M: What about toilet training?

R: Toilet training, no.

M: She is not been able to be on her own to toilet?

R: No, she just does it without our knowledge.

M: Please what other development competence do you expect but has been slow?

R: We think or we want her to at least talk because she hears, as for the hearing when you call he she turns, but we want her to talk we want her to sit, and get up and walk that is what we are expecting from her.

M: Please can you describe how you and your child relate with one another?

R: I call her my Sweety, before she was sick she was my Sweety, then she fell sick she is still my Sweety. Any time she is at home and I am around, I am always with her.

M: How do other children, how do your children get along with other siblings maybe her nephew and nieces?

R: When they come around they all like her, they call her they gp to her play with her.

M: So who does she spend most of her time with and why?

R: The mother.

M: Why does she spend most of her time with her mother?

R: It's because the nature of my work I don't sit at one place, but the mother from home she goes to her shop, so that's why she spends more time but anytime I am at home, I am always with her.

M: Can you tell me some of the resource and items your child has to play with?

R: For now most of the time, it's just the phone, music, because sometimes when the mother is also busy it's the music that she play for her to sit or to lye at one place and enjoy her music.

M: So where does she go when she wants to play?

R: The girl?

M: Yes.

R: She is always at home here.

M: Please if you need advice from someone relating to your child who will you take advice from and tell us your reason?

R: May be, should I say a Doctor or because they are trained in it, that is their field so whatever they tell you, you will have to take it.

M: What are the traditional or cultural practices you know around children with such disabilities?

R: This question is difficult for me.

M: How does the community relate to her type of caliber and her colleagues you might know of?

R: The way she is, because she is still a little girl, I have not observed the way community is treating her, I have not observed it. But they do, people do say that I have a sick child, I have a sick child and that this sick child is worrying me., that are some of the things the community members do say.

M: Tell me a little about the kind if disability your child has? Is it at birth or after birth?

R: It was after birth, she was 10 months when she was down by severe malaria, and that lead her to be deformed, because now she can sit unless you hold her, when you hold her sometimes she sits a little, then she becomes tired if you are not there to hold he she falls off, she can't walk she can't stand, she can't talk but she hears when you call her, she turns and look at you.

M: Does anybody in your family both sides have a similar disability?

R: No.

M: Tell me some of the things you are doing well parenting your child with disability?

R: You know she is my child, so how would I put it, I do my best for her.

M: Please which are?

R: If I am idling I go to sit by her, play with her, sometimes if she is hungry I feed her, when she goes to toilet I clean her up.

M: please tell me some of the things that you know you ar supposed to do but find it difficult to do for your child?

R: It's just the care, I would have wished to have much time to always care for her, but I am the type that doesn't sit at one place, so whenever I am away I call the virtually every 30 minutes to one hour asking of her.

M: How often does the child report being teased or bullied has she ever been bullied even though you said she doesn't go out and all that.

R: No.

M: So how prepared are you to protect her from bullying from the community?

R: That one I am prepared very prepared because I wouldn't give you the chance to come and bully her, I will not take her to you that you should keep her for me, I will do it myself. If I don't have the time my wife will have time for her.

M: Can you describe some of the responsibilities your child has at home?

R: She has no responsibility, her only responsibility is to eat, drink, bath that sort of things then lie down.

M: Tell me about how you discipline your child with disability?

R: The only discipline is talking to her, when you want to urinate tell me or your mother before you urinate why do you go to toilet without telling me? She never reply me.

M: Can you tell me some of the things you have been sensitized about dealing with a child with disability?

R: No, but I will take the responsibility of having patience with her because I know she is not as normal as I am so whatever she does wrong I have to accommodate it, if she was normal she might be okay but because of the illness may be that is why she is doing it so you have to accommodate it.

M: Has any organization done sensitization to you?

R: No.

M: Please what support services are there for children with disability in your community?

R: I am not aware of any support like that. There was a time someone asked me to go to social welfare so I went to them and they said I should put it into writing, so I wrote an application asking for funds to give her further medication, so after giving the letter out for some time they gave me one thousand to take care of further treatment which I did and that is the only support that I got and that is I think a year ago, since then there is not any support again

M: Mr. Nwodio thank you very much for your time. Thank you for granting me this interview.

R: You are welcome.

M: I have ended an interview with Mr. Nwodio the father of Davina at Lambusi at 00:7:50 thank you.

M: I’m here to interview a parent with disable child.

R: You are welcome.

M: Good morning sir

R: Good morning. How are you?

M: I’m fine

M: Please can you tell the number of people living in this house?

R: Right now us we are seated these are my children and as you can see this is my first one born, when he was born he used to hear.

But one day my wife told me that when she call him he doesn’t respond then I also tried calling him but he never responded so we sent to hospital. But they told us that there is nothing wrong with him.

When we left the hospital we went to the herbalist for local treatment so right now we don’t even know what to do. We have 5 children in this house with me and my wife that means we are seven (7) in the household.

M: So as your own child who can’t hear nor talk (deaf)a mean your disable child can you tell me something small about him?

R: Okay what is there right now is, as I don’t know, he can’t hear that means he can’t help me do some of my work. Sometimes you have work to do and you need a helping hand but he can’t help you because he can’t talk.

M: With this your disable child what have you seen that he can do to help himself?

R: Okay what I see he can do is when he goes to school, then government knows that he is a disable.

M: Sorry what I’m saying is right now, does he goes to nature call on his own?

R: Yes we thought him how do it on his own even when he goes anywhere alone. But in the house to I taught him how to do things that I want him to do it.

M: Has he ever spoken for you to hear?

R: NO

M: Have you ever heard him speak?

R: I heard him speak once, when we gave birth to him. By then He was a toddler

M: Like as your own child, what do you think he could do for himself which he is not able to do it and you are not happy about it?

R: Okay right now when he is with his colleagues and they go out when he returned I always want to tell him what he doing I'm not happy but I can't .

M: Can you tell me how he and his colleagues do things, like when he is with his peers?

R: Yeah Like I said it earlier on when they are together and he does thing which is not good I always called him and tell what he is doing I'm not happy so he should stop it.

M: So when he does that are you always able to tell him.

R: Yes yeah.

M: Arrh! what about his other colleagues, like his uncles children, your sister's children, your wife's brother children how do they relates to him?

R: Okay when he is with them and they are doing something which he finds it difficult to do he always run away from them , and when that thing happens thus where I know that he doesn't want to play with them or there is something wrong with him.

M: At what time do you always be with him, or like what time everyday do you have time to chat with him?

R: Okay anytime I wake up in the morning or when I'm to go to work I will let him go to his mother.

M: Why do you that, but you yourself what time you do or what time are always with him?

R: Okay right now that I'm seated here when I'm going anywhere I go with him.

M: Why do you go out with him?

R: So when I go out with him and I see his colleagues always let he joined them to play which he learn new things from them.

M: So that's means you are always with him any time?

R: Yes.

M: Why always so?

R: Yes because he has a hearing problem that why. But if he could hear he would have been roaming freely with his peers without my guidance.

M: Please can you tell me what things he used in playing, what type of things does he used in playing and he is able to learn songs from them?

R: Okay sometimes I see him playing with colleague's football or balloon and sometimes too he will joined them to play with their empty cans of milk (konko) where they put sand in it and be pulling around on the ground. That's how he plays with them

M: When he wants to play where does he goes to?

R: When wants to play? Yeah he always goes to them under shed of this three, but you know children they are together without fighting, but when there is a fight he runs back to me.

(Child screaming at the background)

M: Like this your disable child when you need an advice, where would you seek it from is it from the health service organizations or the elders in your town that you will seek an advice from or those who knows much like the psychology , Which of them would you seek an advice from?

R: Okay right now that I'm as I'm seated as a father of sick person whoever is ready to help I wouldn't stop the person. I will be much grateful to the person for the support provided

M: Like the old traditional method of treatment of this sickness which one of them do you know about that you could use to treat your child?

R: Okay those days we used go out for the local treatment medicines but to no avail right now we don't even know where to go again?

M: I'm asking you about your grandparent and your ancestors what they used in treatment of such ailment of a child.

R: Okay our grandparent used to have it but because of education and right now we don't do such treatment here again.

M: You don't have it here to help you again.

R: Yeah.

M: **Hmm! what about these village people where you are staying right now how are they coexisting with the child?**

R: Okay anyone that sees him always advice me to send him to school. They always tell me to send him to school for proper education.

M: **But do they like him or they dislike?**

R: No they like him even when they see someone who wants to disturb him they chase the person away.

M: **Is it the village people who protect him when anyone wants to disturb him?**

R: Yeah.

M: **Right now can you tell us, was he born like this or he fell sick after his birth?**

R: When the mother gave birth to him erm! ,erm! he used to talk and any time I come back home I will feed him, I even received gifts from my relations like my senior brothers wife and others who give him items like eggs, meat but no knowing he was sick hmm is not easy.

M: **So you mean he was born normal before the sickness?**

R: Yeah.

M: **So like within your father's family do you have someone who also have such sickness or your mother's side do have someone like?**

R: No my father side no one and my mother side too no one.

M: **Can you tell me as a parent what you do with the child is it he is always happy or what do you do right that the child get excited? What and what do you do?**

R: Well when he was born and he fell sick and I thought he would have died but since he survived I'm so excited you are here to support?

M: **Erm! what I'm asking right now is, as a father of the child what are you doing that you know you are doing it well to help the child?**

R: Well what I do to help the child is that the work that I'm doing as he can't hear; I rear animals so he always go to the bush for pastures to feed the animals.

M: So thus that means you trained him on how to rear animals and also you guide him on what to do?

R: Yeah aahaa! when he was born I was excited because I knew my suffering has ended I will get a helping hand on the farm but I didn't know he will fall sick

M: So what you train him on or what do you think you are helping is this?

R: Yeah ermm!

M: So what and what do you think that it would have been very difficult for you right now that he can't talk?

R: Right now from my family there is no one like that and from wife side too no one like that too

M: I'm not talking about your family what I'm asking you is that is there anything like your responsibility which you are finding it difficult to do or something's like providing his food the clothing's or some others needs you wanted to do but you don't have the resource, what is it ?

R: Aaah ok all this things the work I'm doing like the rearing of the animal is all for his upbringing and I'm keeping them for him in future

M: Like, you as a parent what do you think that you can actually do for him but you are not able to do it?

R: Aaahh! (Laughing haahaa! Errm!)

M: Like taking care of his upbringing his education and a lot of responsibilities which you could have been doing but you aren't able to do it?

R: His education? yes the school he goes, but what I found difficult to provide is items like food and provision for the school I swear to God and I can't tell whether he is happy or not.

M: But you know him very well, that is your child?

R: Yeah.

M: But you know as his your child you are suppose to do this thing for him, I'm supposed to do this thing for him but I'm not able to do it but you have been able to some what are those things ?

R: When we wake up early in the morning I prepared egg with tea for him but you know I can't be doing that every day for him alone, how about his other siblings?

M: And as the father of this disable what do you think is very difficult responsibility as a father what really preventing you in taking care of him aside what you told earlier?

R: I had wanted him to understand in future when I'm not around him would be able to take care of himself.

(Child crying at the background)

M: Okay what I'm asking you is that what actually is your responsibility as a father, is it to take care of this disable which you have been trying all your possible best but you are still not able to do it?

R: Well taking care of him is very difficult for me alone, sometimes when he wants to go to school the responsibility as a father is a big burden.

M: And as you living in this village when he goes out does he ever complained that people teased him around or someone has punished him just because he is a disable?

R: Yeah when such thing happens he will run to me and point his finger to the person who is disturbing him. He will pull my hand to the person

M: Does is it mean they teased him because his disable?

R: ohh! Yeah when that happens he will come and take me to see the person who punish him.

M: Is it that they do that to him because he is a disable child or they do that any other child?

R: No sometime when he goes out with his colleagues to climb trees and the owners are come chasing them his other colleagues always managed to run, leaving him alone to be caught that's why.

M: So because of this would you lock him up because they teased him or what his peers push him to do , which his not suppose to do?

R: That is why right now I don't want him to be roaming with his peers again because they are teaching him bad habits as such I always prefers him to follow me out .

M: So are you ready to fight with any other person who try beating him up or?

R: I always tell them when you see them doing anything bad practice discipline all of them but don't single out him alone for punishment which they all did.

M: So as you living within the house what duties do you put him to do? it is by taking care of junior brothers or any house chores. There some small small duties which you assigned to?

R: Well when he wake up early the morning he normal goes to checked on the animals Pent to see whether there is food for them, if there none he will go to grinding meal with maize to process the animal feed for them.

M: So as you're the father when he does something wrong how do you correct him?

R: Toor,, when he does something wrong and I'm not around when I come back and they tell me I always call him to warned him from doing such a thing again.

M: So do you have an experience with him where you have punish him for indiscipline?

R: Yeah he ever did that some time ago, but when he does that for the first I always warned him but when is his second time then I would beat him to stop it.

M: What did he do that got you angry to discipline him, what exactly did he do which deserved you raising your hand on him?

R: First he threw a stone on the head of his junior brother intentionally which he got injured on that day I beat him up but on the second time when he unintentional stepped and killed one of the animal but I didn't beat him.

M: Is it because he threw the stone on his junior brother's head that was why you beat him?

R: Yeah that's why I beat him.

M: So okay as you have been living here, has there been instance that some people will come to talk you about how to handle or take care of your disable child?

R: No,, you are the first person to come to us nobody has ever been here.

M: Uhmm nobody has ever come? Uhhm errrm,,, apart from me nobody have ever been here?

R: Uhmm uhmmm nobody apart from you nobody ever came here.

(Child disturbing on the background)

M: **So what have I'm telling you , do you see the relevance of our conversation, is it helpful in your life ?**

R: Yeah, yeah, uhhmm.

M: **With this place as in such "Pinaa" do have a place where people come to help the needy in the community or support them with disable items?**

R: Toor,,, I have never seen a place like that but first, first when I was a kid, I used to see people who will come to our village here "Pinaa" to give our old ladies cloths and some small, small things but since then I have never seen them here again.

(Child disturbing on the background)

M: **Not old ladies only, how about the needy and the disable in your community?**

R: Yeah they have never been here uhhh uhhhm.

M: **Nobody has ever been here to distribute items to you? Or you don't know any place within this district that you can go for an advice?**

R: No uhhhm uhhmm there is nothing like this in the district here.

M: **There is no place like that? uhhhm?**

M: **Toor,,, I will like to thank you very much for giving me the opportunity to have a conversation interview with you?**

R: Toor,,, I am also grateful for the time shared with me.

(Child disturbing at the background)

M: **Thank you very much!!!**

R: You are welcome.

This is where the interview got to an end with a disable child parent Mr. Charles Kouw.

The interview ended at 10:49 am

Thank YOU!!!!

M: **We are about to begin our conversation so I will like to know the people you are living with.**

R:

M: **Can you tell me something about your son? Like what can you say about his sickness since you are his mother and you are staying with him?**

R: He has been having seizure since childhood until now. I have been consulting many people for medicine but as for now I can say he is getting better.

M: **What exactly do think is wrong with him?**

R: I can't really tell.

M: **Concerning your son Baalayele how is his behavior?**

R: Generally he is a good boy but sometimes he acts in a stubborn manner.

M: **What can you say about his growth and general well-being? I want to find out from you whether he is growing more than his, less than his age or according to his age?**

R: He is growing in the normal way.

M: **Why did you say that?**

R: I can't really tell.

M: **Do you expect him to have been doing better than this? Like to react to situations better than he is doing now.**

R: He was supposed to have been able to understand things better than this but he is unable to do so.

M: What do you think is responsible for that?

R: I will say it because of his condition.

M: How are you able to relate to him? I mean whether you are to cope with his condition or you are facing some challenges living with him.

R: No, we are living in a peaceful manner.

M: Is he able to interact with his friends?

R: I will say somehow because he is not able to play with them as expected. Because of his condition the children fear him and they don't want to play with him.

M: Whom does he normally want to be with?

R: The children of this house. They are closer to him and they the ones he knows better.

M: What are some of the materials he likes to play with?

R: He likes to play with football.

M: Apart from football what other materials?

R: He also likes to play with worn out tyres.

M: Where does he get these items from?

R: His father gives them to him.

M: In case you wish to seek knowledge concerning how well to take care of your children, who do you go to?

R: I always go to my aunt.

M: Why your aunt and not any other person?

R: She is my aunt so she knows what is good for me.

M: Do the people of this community have any tradition concerning children with this kind of sickness?

R: No, I can't think of any.

M: How do the people of this community treat children with this kind of sickness?

R: They treat them well.

M: You said your son has been having seizures, when do you start to experience this from him. It is from birth or it happened at a particular point in his life.

R: It is not from birth. He fell sick at some time which has left him in this state.

M: Does any of his father family members ever have this kind of sickness?

R: No, none of them.

M: What of your own family members?

R: I was suffering from it but God has helped me and have recovered.

M: Can you tell me some of the things you are doing to help me under this condition?

R: I am taking him round looking for medicine. Whenever I get money I take him to the hospital also.

M: Apart from spending money on him what else?

R: Apart from that I don't allow do any hard work.

M: What do you think you would love to do but you are not able to it because of his condition?

R: I would have loved to look for money and take him to better health centre for treatment but because of his condition am not able to do it. I can't leave and go anywhere. So I am constrained.

M: Apart from the medication what other thing?

R: I would have loved to give better food and better clothes.

M: What are some of the challenges you are facing with regard to taking care of him?

R: Yes please, am not able to go to the farm and also do something to help ourselves but because of his condition I can't leave him behind.

M: Do you have any knowledge concerning this kind of sickness? Like if a child is suffering from this kind of sickness what food do you have to give, what measures will you put on place to make sure he is fine?

R: Yes I have.

M: Where did you get this knowledge from?

R: I just use my common senses.

M: Do his colleagues isolate themselves from him?

R: No they don't.

M: What are you doing by yourself to make sure he is fine?

R: I don't allow him to perform any house chores.

M: Apart from not allowing him to perform house chores, does he go to the farm?

R: Yes he does go some times.

M: How do you discipline him as his is under this condition?

R: I always warm him to stop.

M: Apart from warning him what else?

R: Apart from that I don't do anything because I don't want to beat him.

M: So you have never beaten him?

R: Not at all.

M: Have you ever received any special training from anyone concerning how well you can cater for him?

R: No I haven't.

M: Is there any special hospital in this community or elsewhere that takes care of children like him?

R: Yes, we do go to a village called Lassie to collect some drugs for him.

M: Are these drugs really helping him in anyway?

R: Yes they are helping him.

M: How do you know they are helping him?

R: It has reduced the number of times the seizures do occur. It used to be more often but it has reduced drastically.

M: I will say we have come to the end of our conversation. I will that you for the time you have spent for the purpose of the conversation should we need any further information we shall to you. Thank you so much.

M: Mother please can you tell me something concerning your house. I mean I would like to know the people you are living with?

R: Am living with my daughter Am my four grandchildren.

R: All that I can say is about his sickness and that I have been consulting a lot of medical practitioners but there is little improvement.

M: What can say about his growth and development when he was a child?

R: Everything was going on normal until he fell ill which has made him hearing impaired and he is unable to be controlled?

M: Is he able to speak?

R: No he is unable to speak.

M: How is his behaviour in terms of anger?

R: He is not hot tampered because he is still a child.

M: Since Muazu is your grandson, were you expecting him to have seen something different from him as at his age?

R: I was expecting him to be in school so that he could learn but because of his sickness he is still her with me. All his colleagues are in school but he is not.

M: How is your interaction with Muazu is like? I mean do you play with him to make him happy or otherwise?

R: Yes I do play with him and am excited to have him as a grandson. Even if am not happy with him who else will I give him to?

M: Has he been able to interact with his colleagues outside?

R: Sometimes, but they seem to fear him since he can't hear and can't talk. He likes to play with them but they don't give him the chance.

M: Who is closer to him in this family?

R: Myself.

M: Why does he like to be with you more than the other people?

R: Am living with him under the same roof so I have to try everything possible to develop love between us so that we become excited.

M: Can you tell me some if the things he likes to play with?

R: He likes to play with cans.

M: Where does he get these things from?

R: He picks them from outside the house.

M: In case wish to seek advice from some one concerning how to take care of children concerning their growth and well-being?

R: I would consult the teachers in this town.

M: Why the teachers and not any other person?

R: Because they are more educated than me and they teach people a lot of things.

M: Do you think there were, even if it was in the past they would have been able to offer advice to me concerning what to do on it?

M: How do people of this community behave towards children with this kind of sickness?

R: They only sympathize with me but they don't really offer any advice on well to treat the sickness.

M: Can you tell me something about his sickness? I mean what kind of sickness do you think it is?

R: I can't really so much, all that I can say is that he was a normal person until the grandmother took his only for him to fall I'll and she came and gave him to me.

M: Can you tell the time the sickness attacked him?

R: When he was two years old, so I can say two years because he is now four year.

M: Do you know anybody with the same sickness in this community?

R: No, I don't know.

M: In what way are trying to help him as he is under this condition?

R: Am able to play with him, bath him, cook for him and when his clothes are dirty I was them.

M: Can you tell me some of the things you would have like to be doing but you are not able to do because of his condition?

R: Yes, I would have love to go to the and pick Shea fruits but because of his condition I can't go because there is no one helping me to take care of him.

M: What are some the challenges people who have these kind of children face in the society?

R: What I can say is that, they are left entirely to them and you do what you think is good for them.

M: Is he in the position to tell if anyone does anything does something unpleasant to him?

R: No, he only cries but he can't really tell me anything.

M: What have you done to put him in the position to tell if anyone does something unpleasant to him?

R: I haven't, because of his condition; he barely reacts to situations of that nature.

M: **Are you able to correct him when he dies something wrong?**

R: Yes am able to correct him.

M: **How do you do that?**

R: I console him and sometime I do strap at my back.

M: **Apart from that what else?**

R: Apart from that nothing else because he is not a normal person even if I beat him he would not realize what he has done.

M: **What kind of advice do people give you concerning his condition?**

R: They tell me to be extra patient otherwise I can't really handle him.

M: **Apart from the advice have you received any special training from anywhere?**

R: No, I have not received any special training from anyone or anywhere?

M: **Madam please we have come to the end of our discussion. I will say thank you for the time you have given to me. Should we need any information or assistance we shall get untouched with you.**

Region
District Name
Community Name
Urban/Rural
Age of Child
Gender
Date of Interview
Interviewer name
Transcriber name
Interview length
Wealth Quintiles

M:

R: We are three; my husband, myself and our child.

M: Can you tell me a little about your child?

R: It's very difficult for me to talk about my child; my eyes are usually filled with tears when I talk about it. She is my first born and I love her. When I was pregnant, I was asked to abort her but I insisted to deliver her for her to become whatever she ought to be. I have finally given birth and it's 1yr 7months yet still my child could not sit nor do anything. I am a young lady of only 28yrs. I have to work to cater for her and her education. Who do I have to take her for me whiles I go to work? It is really burdensome for me; anytime I think about it, tears fill my eyes and I don't want to even talk about it because it's giving me headaches. She cant see and she cannot talk

M: I am very sorry we are here this morning reminding you of those things you don't want to talk about but just as I said we are here to find out the kinds of aid available to you, how the community perceives children with special needs and to help improbve communication. We really appreciate your interest in trying to talk to us; I must say that.

R: Thank you.

M: Are there some other things you'd love to say about your little girl?

R: I am hoping and praying to God that one day she'll rise to her feet and walk

M: At the moment, what are they things she's able to do?

R: She can try to chat with you but you can't hear clear to understand what she's saying. She at times say daddy but it's not clear; she can turn from her back unto her stomach. Those are the things she's able to do.

M: So what are the things that you think she should be able to do by now but she's slow in doing them?

R: I was expecting that she may be sitting, crawling or walking

M: Do you specifically know the reasons why she's unable to do such things?

R: For that one, I am not God; I do not know the reasons why she's like that. Only God can know but by the education the doctors have given me, I may say it is a fault from the midwives who helped in delivering her. There may be other reasons but from what I witnessed, I know that they are the cause this trouble I am going through. I was pregnant for 49 weeks which is more than the usual 9months until delivery. So when I visited the doctor, he told me that they'll take me through a ceasarian section. When I returned, I met a different doctor who has never attended or done any examination for me ever. This doctor knowing very well that I cannot deliver the baby, asked me to for medicine to aid me deliever the baby. I was desperate to deliver and I was tired carrying the baby because I have carried her too long a time! My stomach was very big; when the baby was 7months old, they advised and wanted to remove the baby from me because I was falling sick too quick and they said that the baby was not getting enough breadth. I was really suffering and it was very difficult for me to carry her for over 11 months. So when the doctor said I should take some medicines to enhance me deliver, I had no choice than to do that. When I went for the medicine, on the second day, I went into labour. They fixed oxygen on me because they said the baby was not having sufficient breath. I was tired, I couldn't deliver the baby lying on my back unless I lie on my side. How can I deliver a baby in that state? I passed through a lot! The midwife who was supposed to assist me deliver left me that she cannot deliver for me so if I cannot do it by myself, then I should just be there. It was her assistant on that night, that helped me to deliver that night. Was it not for that lady, I may have even died with the baby; this is why I say that it is the doctors and the midwife on duty that night who are responsible for the situation in which I am today. If they were to operate me and removed the baby, I don't think I'll be here today being burdened in this way.

M: Can you describe for me a little how you relate with your child?

R: I am well related to her. If I don't see her around, I don't feel comfortable and if I am looking at her, I don't want anything to happen to her. Of course, this is my first child at 28yrs; almost 29yrs. When I conceived her, they wanted me to abort her but I refused. Sometimes, she can really do somethings to provoke you to anger! Sometimes when I am angry, I just feel like dropping her in a toilet pit so that I would be free; when she was very small, I get feelings of just hitting her down so that she'll die and I can also be free but because of the love I have for her, I will never do that! If God takes her life, fine but if God gave her to me by himself, then he should rather change her. The enemy is using every means to make her evil but I am also praying that if she's a good thing that God has given to me, let him give her to me but if it's not a good thing, then he should take her back.

M: You said you were asked to abort when you were pregnant but you refused. Who asked you to abort and die?

R: My husband's parents. As at that time, he's just graduated from the nursing school and has not yet started working. He was about starting his national service so when I was pregnant for him, they told him to tell me to abort because he is not yet working. I only completed SHS before I got pregnant but I refused to abort because I don't know what she'll give me after this one so I'll not abort.

M: So how does your child relate with her other siblings in the house?

R: When I was with my parents, she used to joke with them but since I returned to my husband, it is not so because we are only three at home.

M: So how do the neighbours relate with her?

R: They like her; when they see her, they say she's beautiful so they like to carry her except for the fact that she lacks the ability to sit or do what the others are doing.

M: So with whom does she spend most of her time?

R: Either myself or her daddy but she loves to be with her daddy more than me. Anyutime her daddy goes out and come, she will be shouting "daddy" but it's not that clear

M: Can you please tell why she spends most of her time with her daddy ?

R: She loves her daddy more than me

M: So if I got you right, she spends most of her time with her daddy?

R: Yes

M: Can you tell me some resources or materials that you have in the house that she plays with or she can use to study?

R: I buy toys for her. Toys and some tambourines

M: Why do you buy those things for her?

R: I want her attention to be on it and also so that she can be playing with it on her own.

M: Are there some other resources you've in the house for her to learn with?

R: No

M: Why?

R: There's no money

M: If it becomes necessary for you to sick advise from someone, from whom would that be?

R: My mum

M: Why?

R: Because I trust her most; people can talk to you but they may not be sincere. They may later mock you or tell everyone about whatever you discussed with them. I would like to take advise from anyone but I won't tell my problems to anyone!

M: So when it's health related, who will you talk to?

R: Somebody trustworthy.

M: Let's say a health related need arises right now, who would you talk to?

R: Just like I said, I would look for somebody trustworthy; someone who can help me solve the issue. I cannot talk to anyone about my problems

M: Do you know why those people you talk to, do nothing about it?

R: Selfishness; some would think it is not even a need for you.

M: Do you know of any traditional practices that are done in this community related to special children like her?

R: No

M: Have you also heard of any done in this community either for the child or the mother?

R: No. There are just some people who would tell you that they can heal your child but I've tried for a number of them and it yielded nothing; I just lost money.

M: Can you please tell me a little about those things that you've heard regarding how to handle the children?

R: Oh, they'll say they can treat her and her condition wouldn't be so hard for them to resolve.

M: What can they do, please?

R: They'll say they can cure her from such ailment. Some people have attributed it to witches who have changed the destiny of the child so they can help me. I made attempts by visiting them with the child but it yielded nothing; I didn't see the result I wanted to see.

M: So who are these people who are saying they can do it?

R: The traditional people; they use herbs to try to cure her.

M: How does the community react to such situations?

R: It depends on who you are. If you're a traditionalist, things would be easier for you but if you're a Christian, it'll take you a lot of energy before you can go to them for help

M: Kindly speak a bit louder for me. In this community, how do they see children like her and her parents as well?

R: I don't know because none of them comes to me to tell me anything about the child so I wouldn't know.

M: Maybe no one has come to speak to you about the child but how do they react when they see the baby with you in town or at your work place?

R: Some people pretend they love the baby but I cannot tell the sincerity of their hearts. Some people show their love for the baby but others don't want to even come close to her. I've heard people say that when I was pregnant, I had sex with another man who isn't my husband and the result is what has happened to my child; but I know the truth by myself and I know that the truth would set me free. There are some people whose comments can even make you commit suicide because those words are not supposed to be coming from a friend but they do it anyway! Sometimes, it is even from parents; it is not easy but we will be through successfully by God's grace.

M: Do you know why they say those things?

R: I don't know but it may be because of the child's condition; I am not the first to be experiencing this. When this happened, I was sent to Tema general hospital. I delivered at Keta and I was referred to Battor catholic hospital who also referred me to Korle Bu so I have seen a lot of disabled children; excuse me to say my child's condition is better as compared to others that I've seen. If you've not experienced like this before, you may say anything that's appeasing to you. So they say all kinds of things they wish to say but I know that it happens in life but God can help you to be through it; if He doesn't too, we'll take it like that.

M: **Can you tell me a little about your child's ailment?**

R: Some other children, immediately you see them, you'll realise that something is wrong with them but for my child, if I don't tell you, you may never know. I carry her to several places and I am not shy about her because it's God who has given her to me. I believe God didn't make her like this but it's some evil people somewhere who are trying to change her destiny as well as mine.

M: **So at what point did this situation start?**

R: Right from the day of delivery.

M: **Do you have a family member who is disabled?**

R: No, not even one

M: **How about your husband's family?**

R: No

M: **So what are some of the things you are doing parenting your child?**

R: I was a seamstress apprentice but her condition made me dropout so I am currently selling items to cater for her.

M: **Aside the trade, what other thing are you doing to care for her?**

R: That's the only thing I am able to do.

M: **Can you tell me some of the things you know you are supposed to do but you find it difficult to do for your child?**

R: Yes a lot of things. I intended to go to the nursing training college before I got pregnant. I decided that after I deliver, I can give the child to my parents and return to school but it's two years already and I am still home because of the child's condition.

M: **Okay. I want to know the things you know you are supposed to do for her but you find it difficult to do for your child?**

R: I am expecting that at around one and a half or two years, she should go to school but because of her condition, she cannot go to school; and all these things are problems for me.

M: **Okay. I want to know the things you know you are supposed to do as a mother for her but you find it difficult to do for your child?**

R: That's what I said earlier that I should've taken her to school when she's of age to go to school as a mother should do.

M: Aside this, what else must you do that is difficult for you to do?

R: It is quite difficult feeding her because I expected her to be able to eat or even try to be bathing by now but that's not the case.

M: So what are your challenges in parenting the child?

R: There are many challenges. If she were healthy, she could've helped me in selling so that we can get money to buy her needs for her but because she is not healthy, she's always at my back and I am able to achieve little with her behind me

M: So if I get you right, your challenge is that you have not time on your side because the child is unable to do anything?

R: Yes

M: What special skills do you have that enhances you in taking care of the child?

R: There are some chairs specially designed for such children to be sitting in but it is expensive so I couldn't afford to buy it for her. If I needed to buy something for her, I don't have money; neither my husband nor myself are working. My husband has finished his national service and awaiting posting; so he is unable to give me enough money as he should for us to be able to cater for the girl.

M: Is the child able to tell you whether they mock or bully her?

R: She cannot say it sometimes if anything saddens her, she'll only cry but cannot say anything.

M: But do you see people mock or bully her?

R: Yes

M: How do you put things in place so that people won't bully her?

R: I have to take care of her; I must keep her neat and give her food so that people won't laugh at her

M: So how are you preparing her so that others won't mock her in future?

R: I must take care of her and teach her the right things

M: **Is there anything she does at home?**

R: Things like what?

M: **Like household chores**

R: No

M: **Okay. How do you discipline the child?**

R: She's not of age so that she'll be doing wrong things to even deserve punishment except that she won't like to sleep after you've bathed her so that you'll also get a little space to do something by yourself. And if she behaves like that, I only beat her to stop.

M: **If I get you right, you mean that you discipline her by caning her? Can you explain that part to me?**

R: I don't usually beat her; in fact I don't beat her because I don't want her to feel lonely at all.

M: **Can you give me an example of an instance where you punished her?**

R: Usually, when you bath a child, you expect her to sleep after taking her bath but she's not like that. She'll refuse to sleep and allow you a space to do something or some work at least; so I sometimes leave her to cry. After she finish crying, she'll then sleep and I can also be a bit free.

M: **If she disobeys, you only leave her to cry?**

R: Yes

M: **Is there anything else that you do to discipline her?**

R: No

M: **What have you been taught in reference to how to parent the child?**

R: No

M: **Perhaps, maybe at the hospital when you delivered**

R: Oh yes! They taught us how to take care of the child's foods to avoid her from contracting cholera

M: **Were you taught anything in reference to how to take care of her in her disability?**

R: No, we were taught nothing

M: You were taught nothing?

R: Yes

M: What kind of support come from the family with regards to children like this?

R: I don't know of any

M: Has anyone approached you or have you heard of any aid that is available for children with disabilities?

R: No one has approached me neither have I heard of any

M: Thank you. We shall bring our discussion to a close. But do you have anything else to add to all what we've discussed?

R: I have nothing else

M: Thank you very much.

M:

R: We are about 5 in the family but I am here with my Grandson,

M: **This implies that it's you are currently only two staying here, yourself?**

R: Yes pls

M: **Okay. Pls is your Grandson male or female?**

R: He's a boy

M: **Pls tell us a little about your grandson**

R: Okay. I would say right from his conception to his birth, his mother was staying with me so I took care of her and the pregnancy as well. Even after the birth of XX, I am still the one taking care of him, even now. He seems to be having eye problems right from infancy but it has not been serious like it is now. His eyes itch him so you'll find him scratching that area and you may see his eyes to be as though there are wounds on them. But I cannot tell if he can see well or not because he's young. He's not yet attending school so that a teacher may complain whether he can see and read well or not

M: **So considering the activities he engages in at home, can you say he can see well at times or not?**

R: Oh on some days, I can see that he sees very well especially the days where his eyes itch him not. But the days he experiences the irritations in his eyes, you can even observe that his eyes seem to be foggy and he experiences blare visions. You can also notice this in his walking, because, he usually doesn't walk well on the days he cannot see well

M: **So if I get you right, you're implying that if his eyes become red from the scratching, he doesn't see well and that influences his walking as well?**

R: Yes

M: **What are the qualities or consistent behaviours that kingdom who is now four years has exhibited since infancy?**

R: The number one thing is that he doesn't love to sit at home. When you ask him to sit and do anything for you and that you'll be back in jiffy, before you return, he'll not be there. That's one of the main reasons I lash him!

M: **He doesn't love to sit home?**

R: Yes

M: **With reference to his learning and moral training, how exactly do you see him doing in this regard?**

R: It's just like I said, He doesn't love to sit at one place or home! That's why I say he's not trying to show any qualities of a humble child. And that's why I sincerely lash him! I want to raise him in a good mora way that he'll learn to take instructions from the elderly.

M: Please I want to know which things he is able to do as from his infancy till now

R: He can't do anything. Even if he goes to town and his friends beat him up, he only cries and do nothing else!

M: He can't even do anything at home?

R: In exception of asking him to fetch water for you; which he can do, there's nothing else he can do

M: Does he know how to use the toilet?

R: No

M: What are some the things that you had wish he is able to do when he was growing up but he couldn't do or perhaps, he did but did very lately in his growing stages?

R: It's just like I said, he doesn't love to sit at a place. I don't want him to be leaving home so that other children would beat him up but he won't listen. That is what worries me a lot.

M: What other qualities do you wish to see but he isn't responding to?

R: I usually fetch water for him to bath by himself or maybe even to just begin to bath before I help him with it. But he's not able to bath himself. If I ask him to sit and watch over something, before I realise, he's gone! These are my worries. So I cannot see any real development in his growing years.

M: Did you say you can see improvement in his relevance?

R: He's got problem with his eyes from childhood, sometimes, you can see wounds on his eyes as his scratches them. And you can notice that he's not able to see well in the way he even walks. If it's not itchy, you'll observe him see and walk very well.

M: What do you think are the causes of his irresponsiveness to the training you're giving him?

R: I can see he's very stubborn! A child learns how to be respectful from childhood. You can see this when that child is able to heed to your voice or call in terms of any instructions given him. But in the case of this child, I do not think he's willing to learn anything because

he doesn’t heed to any instruction that you may give him! He will readily disregard your command in your absence.

M: How’s the relationship between your grandson, Kingdom and yourself?

R: we are very okay except for the fact that he’s very stubborn. Stubbornness is his problem

M: I want to know how the relationship you have with him is like

R: I always go anywhere I want to go with Kingdom. When I bath him and lay him to bed, he decides when to sleep or when not to. Sometimes, he does sleeps and other times he can also run away from me when I sleep off. That’s why I strongly think Stubbornness is his problem. Because he’s not becoming the child I am raising him to be.

M: How is the relationship between Kingdom and His other friends?

R: He visits his friends and usually, they beat him up. He’s older than them but he doesn’t know how to fight so when they beat him, he only cries and that’s all. Even as he cries, he still stays with them until I come for him.

M: Why is the relationship between him and his friends so?

R: Usually, children do fight. So if he goes out of home and there’s a fight, they usually beat him up because he doesn’t know how to fight

M: Who does Kingdom usually spend most of his time with?

R: He stays at Yawodie’s house

M: Please with who exactly?

R: Oh they are a lot!

M: What’s the relation of Kingdom with the people of that house?

R: We are a family

M: Why does Kingdom spend most of his time in that house?

R: That’s because there are no children in this house where we are staying so he’ll love to go to his age mates and friends. That’s why he usually goes there

M: Please tell me about some of the things that you have in this house with which the child can learn or even play with.

R: There's nothing please. There's nothing to play with

M: I am not referring to those things that are foreign made only. It could be indigenous ones designed by someone for him to play with

R: There's nothing. That's why he goes to his friends to play

M: Where does he go usually to play?

R: He goes everywhere. He can follow his friends to our other family house or anywhere in town

M: Do they go to town to play?

R: Yes

M: So is there anything he can use to learn at home?

R: No, there's nothing. He's not yet started school so there's nothing as such

M: How about some items to allow him learn at home; as in for household knowledge sake?

R: He wouldn't stay at home so that you may say he will be learning anything by you. He doesn't stay home so I cannot tell if he's learning anything right now

M: If you were asked to seek advice from someone about how to raise your children, from whom would you take such advice?

R: I cannot tell from whom I can take such an advice! Some people know how to teach very well so if those people come to teach about parenting, you have to learn

M: I meant if you were to take an advice from someone right now concerning how to raise your child, from whom would it be?

R: I don't have anybody who can advise me right now. I go by what I know

M: Concerning the child's relationship, in terms of how he plays and relate with people around here, who can you seek an advice from?

R: I don't have anyone. There are people but I don't ask people advice about how to raise my children or anything so I go by what I know

M: Please why don't you seek or take advice from others?

R: I don’t like to be close with people. There are some people who can play with your words to give a different meaning before others once you’ve discussed anything with them. That’s why I just trust what I know

M: If I get you right, you mean that if there’s the need that you seek an advice for someone, you won’t do that because you don’t like to be close with people

R: Yes. Even if I am close with people, I don’t know what kind of advice they’ll give me that I would be pleased with

M: Please, I would be asking questions concerning cultures or rites that are concerned with child upbringing in this community.

R: Over here, taking care of a child means knowing what he eats daily, ensuring he baths each day and giving him medicine to recover from an ailment.

M: Please what kind of rites are performed for children with disabilities in this community?

R: Which kinds of disabilities?

M: It could be the blind, the lame, the dumb and the deaf

R: They haven’t performed any rites for any of them that I have seen

M: It could be that you’ve not seen any before but have you heard about any of such rites performed for the disabled children in this community?

R: No, I haven’t heard of anything like that. There are so many children with disabilities but they don’t do anything to any of them. I haven’t heard of anything like that before.

M: You haven’t seen or heard of anything like that?

R: Yes

M: Okay. Please can you tell me a little about some disability traits that are worrying Kingdom right now?

R: The only thing I can say Sincerely, about Kingdom is that his father’s house is full of children with disabilities

M: So can you tell me about Kingdom’s?

R: I do not really see any disability with Kingdom apart from his eye problem

M: Can you tell me a little about his eye? From What time did it begin to worry him?

R: Like I said, it started from birth. His eyes were red as though there was blood on his eyes from birth. So we used to drop breast milk on his eyes and as we do that, the blood disappears. It is up to this age that we are realising that he may probably be suffering from an eye problem.

M: Did you say, there are many children with disabilities from Kingdom's father's lineage?

R: Yes

M: Is Kingdom's father with any disability?

R: No but his siblings are

M: Are there some other disabilities in his family?

R: Yes, there are a lot from his father's lineage

M: Can you please mention a few?

R: Epilepsy, "dwarfs" (people with disproportionate short stature)

M: Tell me a little about some of the things you do in taking care of Kingdom

R: Like I said, right from his conception, I took care of his mother until she delivers. After she gave birth, my husband came for them before they later return here

M: Can you talk about some good things that you believe you do in taking care of Kingdom?

R: It's basically providing food for him and ensuring his healthy living. Sometimes, because I don't have money, I am unable to take him to the hospital so I only take him to the pharmacy to buy some drugs for him to take.

M: Please tell me some of the things you think you need to do but you are unable to do or perhaps, it is very difficult for you to do in the child upbringing?

R: Financial problems!

M: In which way has your financial problems hinder you from doing the things you ought to do in raising the child?

R: Like I said, it is because of the financial problems that I usually take him to the pharmacy or drug store instead of the hospital when he is not well. I feel like I am not able to do exactly what I have to do to him because financial problems.

M: Is there any other thing that hinders you from doing the things you have to do for the child in his upbringing?

R: I don't have anything else apart from the financial problems. I really intend several times to take him to the hospital when he's ill but we have never been able to because of the financial challenges, therefore we only buy those drugs from the drug store for him.

M: Please can you tell me about your sufferings or challenges you face in raising someone like Kingdom?

R: I go through a lot. It has made even me almost like a patient. I am facing a lot of sicknesses; this has made it difficult for me to be active in working like before.

M: Are there teachings on how to take care of children with disabilities or how do you find it taking care of a child with a disability like Kingdom?

R: Are you referring to taking care of other children?

M: There is difference in the way a normal child and the child with a disability are being taken care of. In this vein, what are your challenges in raising this child?

R: The only challenge is trying to work hard to take care of him

M: Please what kind of work?

R: Currently, I am working with the Zoomlion but they don't pay us regularly. So if we are faced with sicknesses, it really worries me and I struggle to take care of him.

M: How many times has the child come to tell you about others mocking or beating him up?

R: He never opens his mouth to say any of that but when I hear him cry, I go to wherever he is by myself then I would be told who beat him up and I'll take him home.

M: How are you taking care or preparing him that other children or people who live around here may not mock or beat him up?

R: I don't do anything else apart from bathing him and keeping him neat. I usually ask him to stay at home but he decides to go out of home, he bears the consequences by himself. So, I don't do anything else apart from bringing him home when I hear him cry.

M: Are you saying that you have not put anything in place so that they will not beat him up when he goes to town?

R: Like I said, I go and bring him back home. Sometimes, we both sleep but by the time I wake up, he has run away to town.

M: But I want to know which measures you're putting in place so that others would not always mock or beat him up when he goes to town.

R: Who will mock him? No one can mock him because there's nothing to mock him for. They can't mock him for being hungry or being dirty because I take care of all those. I never like to see him go hungry!

M: Do people mock him for his eyes when he visits the town?

R: I don't follow him so I can't tell

M: When they beat him up in town, why does he cry?

R: Your questions are becoming difficult for me; I cannot answer them again. It's even late and time for me to eat. I don't know which answer to give you again. What I have is what I have said to you

M: We'll be done soon please. What kind of work does Kingdom do at home?

R: He does no work

M: He doesn't perform any household chores like sweeping etc.

R: He does no work

M: Does he perform any small function or ran any errand for you?

R: He may fetch water for you or bring you a chair to sit. Aside these, he does nothing!

M: That means, he has no morning chores in the house?

R: It'll be fetching water for him to watch his face and giving him pepsodent to brush his teeth. That's all

M: Are those his chores?

R: He's not old enough for me to send on errand so that's all

M: Please can you tell me about how you punish Kingdom?

R: I have a cane specially for him. When he's proving stubborn and leaves for town where he's been beating up, I would also beat him with the cane before he'll come home. Did you see how I have to lash him before he left here when he came to us here making noise?

M: Yeah

R: That's how I train him. If I don't threaten him with the cane, he'll never respect you.

M: What other punishment other than caning him do you mend out to him?

R: That's all. There's no other. I can't starve him to punish him. Anytime he proves stubborn, I lash him

M: Please let us continue

R: So wouldn't I eat today? I am very hungry. I don't eat late into the night; I may not be able to eat food containing pepper.

M: We shall be done soon please

R: I am extremely hungry

M: Can you tell me about some of the lessons you learnt from raising children with disabilities?

R: I have not taken care of any child with disabilities before

M: I am referring to people with eye problems like Kingdom, people with epilepsy and the rest. Have learnt any lessons from raising any of them?

R: I have never learnt about them!

M: Has anyone ever come to this area or community to teach about how to take care of children like kingdom?

R: No. no one has ever come here to ask us to come with our children to be taught on how to raise our children well

M: Please what kind of assistance is there for children like Kingdom in this community?

R: No one has ever given us any assistance

M: Concerning health care, safety and feeding, what kind of assistance has ever been given to children like kingdom?

R: Is the person intending to do it for us or it is actually in session now?

M: I mean that is currently in session

R: No one has ever come to help children here. I have never seen any

M: Oh okay. Thank you. Please shall end our discussion here.
